# Iodine Nanoparticles (Niodx^™^) for Radiotherapy Enhancement of Glioblastoma and Other Cancers: An NCI Nanotechnology Characterization Laboratory Study

**DOI:** 10.3390/pharmaceutics14030508

**Published:** 2022-02-25

**Authors:** James F. Hainfeld, Sharif M. Ridwan, Yaroslav Stanishevskiy, Henry M. Smilowitz

**Affiliations:** 1 Nanoprobes, Inc., Yaphank, NY 11980, USA; yaroslav.stanishevskiy@nanoprobes.com; 2 Department of Cell Biology, School of Medicine, University of Connecticut Health Center, Farmington, CT 06030, USA; ridwan@uchc.edu (S.M.R.); smilowitz@uchc.edu (H.M.S.)

**Keywords:** brain tumors, nanoparticles, iodine nanoparticles, glioma, brain metastases, radiotherapy, cancer, iodine, breast cancer, dose enhancement

## Abstract

Effective and durable treatment of glioblastoma is an urgent unmet medical need. In this article, we summarize a novel approach of a physical method that enhances the effectiveness of radiotherapy. High atomic number nanoparticles that target brain tumors are intravenously administered. Upon irradiation, the nanoparticles absorb X-rays creating free radicals, increasing the tumor dose several fold. Radiotherapy of mice with orthotopic human gliomas and human triple negative breast cancers growing in the brain showed significant life extensions when the nanoparticles were included. An extensive study of the properties of the iodine-containing nanoparticle (Niodx) by the Nanotechnology Characterization Laboratory, including sterility, physicochemical characterization, in vitro cytotoxicity, in vivo immunological characterization, and in vivo toxicology, is presented. In summary, the iodine nanoparticle Niodx appears safe and effective for translational studies toward human use.

## 1. Introduction

### 1.1. Mechanism

Glioblastoma multiforme (GBM) is a brain cancer with a poor prognosis. The standard of care is surgical resection followed by radiotherapy (RT) and chemotherapy, but the five-year survival is only 5.6% [1] due to its location in the brain, rapid onset, high recurrence rate and resistance to currently available therapies [2]. New effective treatment methods are desperately needed.

**Figure 1:**
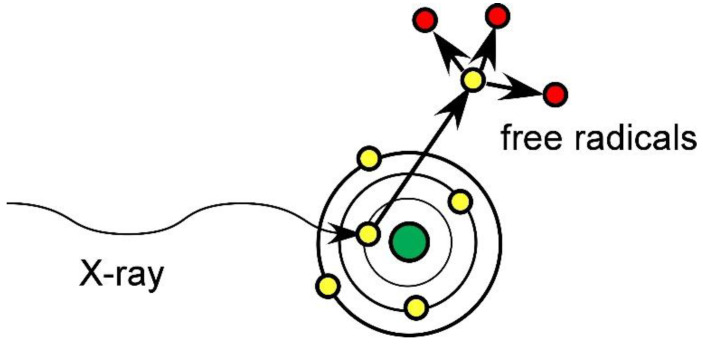
High-Z atoms absorb X-rays and eject electrons creating tissue-damaging free radicals.

One such new method that appears very promising is the loading of high atomic number (high-Z) atoms that highly absorb X-rays to the tumors followed by RT [3,4,5,6,7,8,9,10]. The absorbed X-rays eject inner shell electrons (the photoelectric effect) which then create tissue-damaging free radicals (Figure 1).

**Figure 2:**
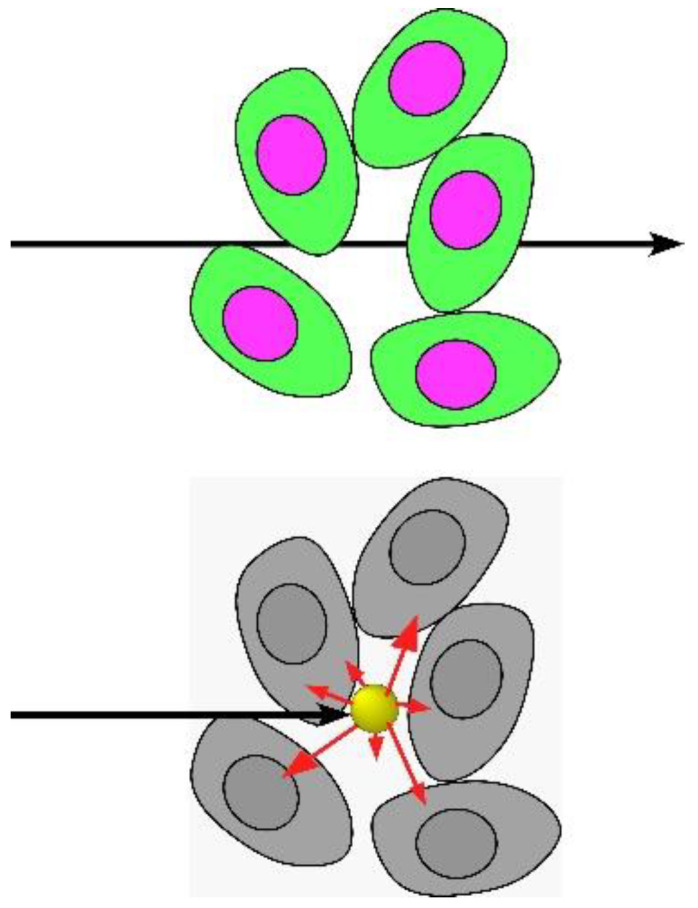
X-rays that would normally pass through tissue are instead absorbed by high atomic number nanoparticles and the energy deposited locally, boosting local dose [11].

This in effect increases the local radiation dose (Figure 2), potentially overcoming the main limitation of standard radiotherapy (RT): to deliver a high enough dose to the tumor without overly damaging normal tissue.

**Figure 3:**
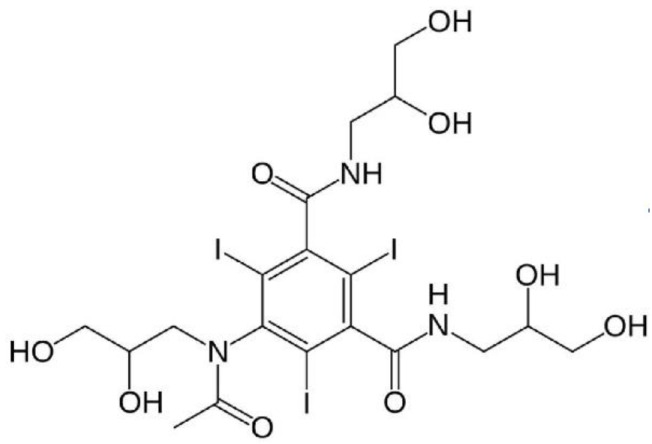
Iohexol (Omnipaque®). Molecular weight 821, 46.4% iodine by weight.

This local radiation dose enhancement of high-Z X-ray-absorbing atoms has been known for many years [12]. Extensive work using standard iodine X-ray contrast media was pioneered by Norman and coworkers who showed significant tumor regressions in test animals [13]. A phase 1 trial using a modified CT scanner with brain tumor patients and IV injected iodine contrast media showed the method to be safe and promising [14]. More recently, a synchrotron was used to irradiate glioma patients also after IV injected iodine contrast media [15]. The irradiation was timed to maximize the iodine in the tumor, which averaged 0.19% iodine by weight (average of 12 patients [16]). This concentration was expected to yield a dose enhancement of 17%. This benefit is largely limited due to the use of standard iodine contrast media which clears the system very rapidly through the kidneys, thereby reducing tumor uptake. For example, iohexol (Figure 3) has a blood half-life of 45 s, followed by a slower phase half-life of ~13 min [17]. At maximal tumor uptake times, the vascular level in the normal brain parenchyma was ~0.5% which could also cause non-tumor effects. The pharmacokinetics of standard iodine contrast media then poses restrictions in its ability to deliver high levels of iodine to tumors while clearing normal tissue.

For effectiveness and specificity, the goal is to have high tumor uptake, which takes many passes, and also good non-tumor clearance.

### 1.2. Gold Nanoparticle Imaging and Radiotherapy

**Figure 4:**
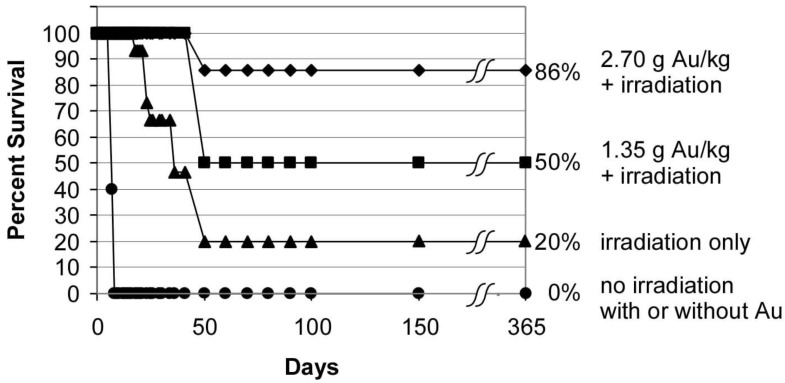
Survival graph showing 86% survival at 1 year after tumor loading with intravenous gold nanoparticles (AuNPs).

To overcome this delivery and clearance problem, we developed high-Z gold nanoparticles (AuNPs) with longer blood half-lives that have more time to infiltrate tumors and clear normal tissues. In the first mouse trial using a subcutaneous mammary tumor, intravenous AuNPs followed by X-ray irradiation resulted in up to 86% long-term survival, with no tumors histologically detected after 1 year (Figure 4 [18]).

Tumor to non-tumor ratios were ~8:1, with tumor concentrations of 7 mg Au/g body weight. Nanoparticles were 1.9 nm in size, and irradiation was 26 Gy with a 250 kVp clinical X-ray therapy machine.

**Figure 5:**
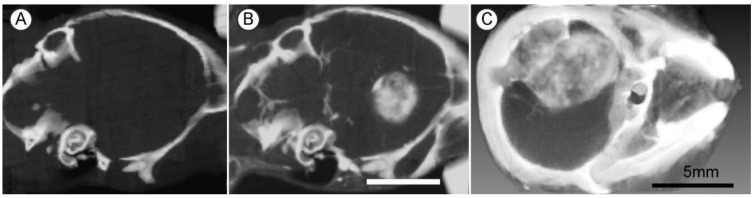
Live mouse microCT images of gliomas 9 days post implantation. (**A**,**B**) Same mouse before (**A**) and 15 h after (**B**) intravenous (IV) injection (4 g Au/kg); (**C**) Larger tumor imaged 15 h after IV injection of 1.7 g Au/kg. X-ray source was 45 kVp. Bars (**B**,**C**) = 5 mm.

To achieve an even longer blood half-life to obtain better tumor to non-tumor effects, 11 nm AuNPs were constructed with a PEG coating (AuroVist^TM^), having a blood half-life of 24 h, and used to treat a challenging orthotopic highly malignant advanced glioma, (Tu2449), syngeneic for B6C3F1 mice. Fifteen hours after IV injection of 4 g Au/kg, the tumor-to-normal brain ratio was 18.8:1, and tumor concentration was 1.5% w/w gold. This level of uptake and normal brain tissue clearance produced clear X-ray images of the tumors with low non-tumor levels (Figure 5).

**Figure 6:**
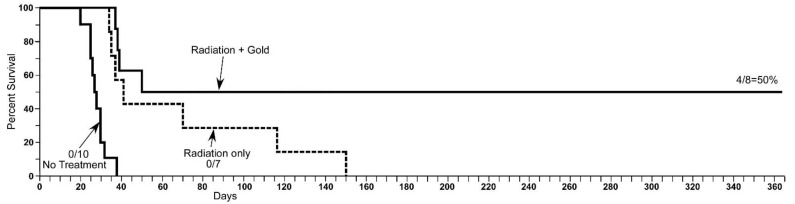
Survival graph of mice bearing invasive orthotopic gliomas showing rapid demise with no treatment, no benefit from AuNPs only, some life extension with radiotherapy (RT) only, and 50% long-term survival (no tumor detectable after 1 year) when RT was combined with a prior IV injection of AuNPs. Durable responses seen here may have been due, in part, to a functional immune system.

Perhaps one of the most exciting aspects of tumor painting with high-Z nanoparticles is their use to enhance radiotherapy (RT). RT is used in about 70% of all cancer treatments [19]. Tumor brain regions with AuNPs were irradiated with a single 30 Gy 100kVp dose. Results are shown in Figure 6 [20].

### 1.3. Iodine Nanoparticles

**Figure 7:**
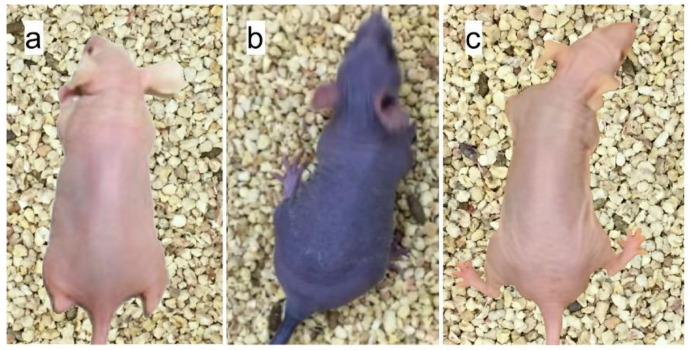
(**a**) Normal nude mouse; (**b**) nude mouse 24 h after tail vein injection of 1 g/kg 15nm PEG coated AuNPs. Skin color change was almost immediate after AuNP injection and showed little change even after 1 year; (**c**) nude mouse 24 h after intravenous injection of 4 g iodine/kg iodine nanoparticles.

Although gold nanoparticles worked quite well for this application, they are intensely dark and cause long-term skin discoloration (Figure 7 [21]).

**Figure 8:**
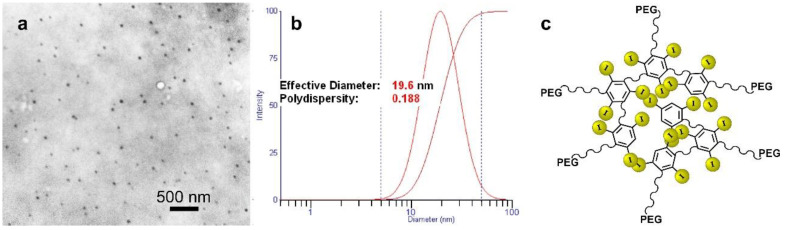
(**a**) Electron micrograph; (**b**) dynamic light scattering; and (**c**) schematic of the iodine nanoparticles (Niodx).

Hyperpigmentation after parenteral gold therapy was first described in 1928 and termed chrysiasis, when gold therapy was widely used for several common conditions such as tuberculosis. The discoloration was found to occur even after only 1 g of gold administration [22]. Skin pigmentation is also seen with silver and silver nanoparticles, called argyria [23]. To overcome this drawback, an iodine nanoparticle (INP, “Niodx”, a Nano-iodine-X-ray absorber) was developed by Nanoprobes, Inc. (Yaphank, NY, USA) which is colorless and shows no skin discoloration (Figure 7 [21]). With a size of 20 nm, it does not clear via the kidneys (which filter molecules less than about 5 nm). An electron micrograph, dynamic light scattering, and diagram of its structure are shown in Figure 8. The mean hydrodynamic diameter was found to be 19.6 nm with a polydispersity of 0.188 (19.8 ± 8.6 nm).

**Figure 9:**
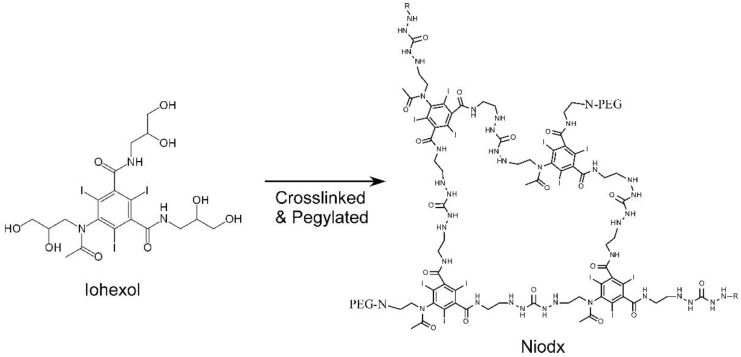
Niodx is made from crosslinked iohexol with a PEG coating.

Chemically, Niodx is prepared by crosslinking iohexol and covalently attaching polyethylene glycol (PEG) to the outer surface (Figure 9). Further structural and synthetic details are described in reference [21], and extensive physicochemical characterization is reported in the Supplementary Information.

Niodx is well-tolerated at 7 g iodine/kg in mice, the highest tested. This level is somewhat surprising, as a clinically used iodine contrast agent, diatrizoate, has an LD50 of 7.5 g iodine/kg, producing acute death in 50% of mice at that dose [24].

### 1.4. Iodine Nanoparticle Imaging

**Figure 10:**
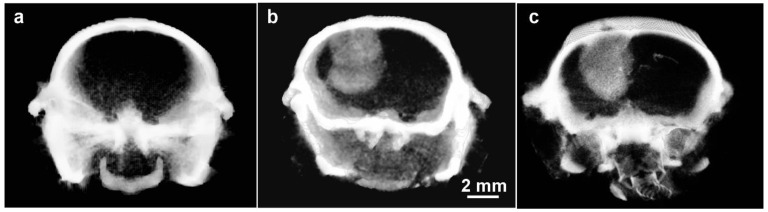
MicroCT images of orthotopic gliomas growing in brains of athymic mice. (**a**) Before Niodx injection; (**b**) same mouse 24 h after intravenous injection (Niodx, 3.5 g iodine/kg); (**c**) Different mouse but taken 3 days after IV Niodx injection. X-ray source was 70 kVp.

Cancers stimulate new blood vessel formation to support growth, and this leaky endothelium can accumulate NPs. An example of a glioma imaged with Niodx is shown in Figure 10.

The tumor to non-tumor ratio was measured to be ~20:1. It was also found that excellent tumor imaging remained after 3 days (Figure 10c), and likely longer (not tested). Such high resolution tumor painting could be used to accurately align and develop an optimal IMRT (intensity modulated radiotherapy) treatment plan. As the tumor painting is stable over days, additional contrast agent injections would not be needed for every RT fraction, especially for hypofractionated regimens.

It should be noted that the favorable tumor to non-tumor loading with Niodx in these glioma studies (human U87 in mice) was achieved without active targeting, presumably due to the Enhanced Permeability and Retention (EPR) effect [25]. Tumor blood vessels are, in many cases, more leaky towards nanoparticles. Some brain tumors have an intact, tight blood–brain barrier (BBB) blocking nanoparticle entry, but most GBM tumors in humans have an altered or disrupted BBB [26].

### 1.5. Iodine Nanoparticle Glioma Therapy

**Figure 11:**
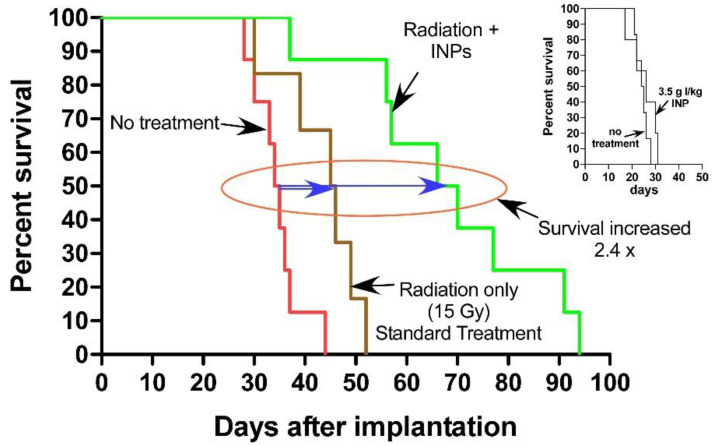
Kaplan–Meier survival graph showing survival vs. days after implantation of orthotopic U87 tumors. RT only (15 Gy, n = 6) extended life on average (at the 50% level, small blue arrow) by 16 days (compared to no treatment, n = 8), but 7 g I/kg Niodx + 15 Gy (n = 8) extended life by 38 days (2.4-fold). Inset shows the Niodx without radiation had no effect on survival. X-ray source was 100 kVp.

Niodx was then tested for efficacy when treating human U87 gliomas implanted in the brains of athymic immunocompromised mice lacking the adaptive immune system. Once again, the NPs themselves, without irradiation, had no effect on survival (Figure 11, inset). However, when combined with RT, they were found to provide more than double median survival (Figure 11 [27]).

**Figure 12:**
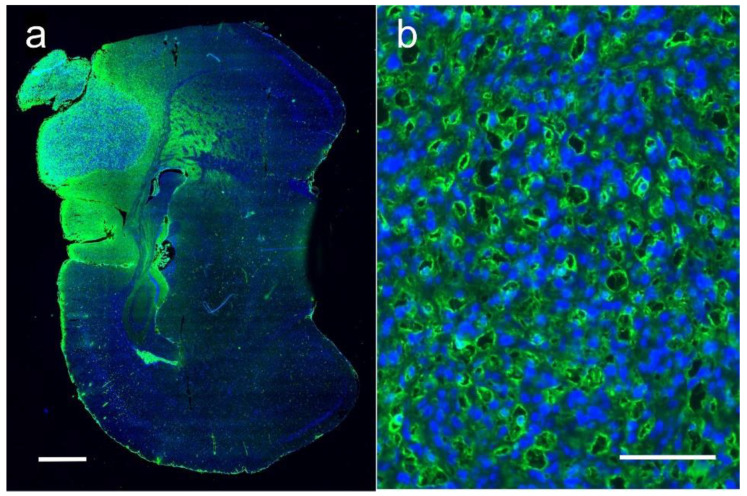
Histological section of an orthotopic human U87 glioma growing in the mouse brain. (**a**) is a coronal section of the whole brain with tumor in upper left. Green is a fluorescent secondary antibody immunolabeling an anti-PEG antibody, specific for the PEGylated iodine nanoparticle. The Niodx targets the tumor and the surrounding edematous growing region. Blue is DAPI, staining nuclei. (**b**) is a higher magnification of the tumor region. Niodx highly stains endothelium surrounding all tumor capillaries (round-like “holes” in image). Bars: left image 1 mm, right image 50 μm.

Histological examination of experimental orthotopic gliomas showed that Niodx targeting after IV injection was quite specific (Figure 12). Niodx was found localized to both the tumor and the surrounding edematous region.

### 1.6. Iodine Nanoparticle Radiotherapy Synergy with Drugs

**Figure 13:**
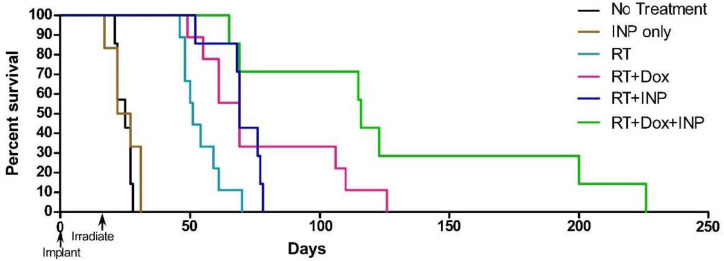
Survival graph showing results with Doxil, Niodx (INP), and radiotherapy (RT, 15 Gy). Groups: no treatment (n = 7), INP only (n = 6), RT (n = 9), RT + INP (n = 7), RT+Dox (n = 9), and RT + Dox + INP (n = 7). X-ray source was 100 kVp.

Upon irradiation, the high concentration of Niodx in and around tumor endothelium would be expected to selectively damage the blood–brain barrier (BBB) and blood–tumor barrier (BTB) in the tumor region. We hypothesized this could break down one of the barriers to drugs that generally are not effective against brain tumors as they do not penetrate well into the tumors. To test this, intravenous Doxil (liposomal doxorubicin) was administered to mice after RT performed in the presence and absence of INPs (Niodx). Results are shown in Figure 13.

These results showed RT+Niodx and Doxil were synergistic with one another and potentially confirmed that the breakdown of the BBB/endothelial barrier greatly enhanced drug delivery and increased efficacy. This may provide a way to enhance the effectiveness of chemotherapy, not only for brain tumors, but other cancers as well. Another independent study imaged better liposomal penetration and Doxil treatment after irradiation of subcutaneous tumors in the presence of AuNPs [28].

### 1.7. Iodine Nanoparticle Targeting

**Figure 14:**
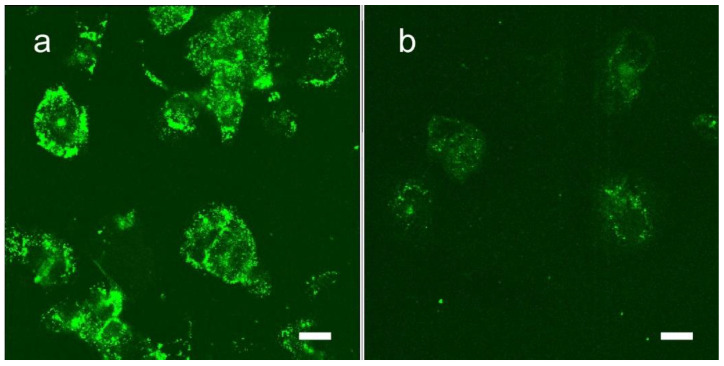
Uptake of Niodx targeted with transferrin (**a**) and non-targeted Niodx (**b**) 16 h after in vitro incubation with U87 human glioma cells. The uptake of iodine is about 5 times with the transferrin targeting in this study. Bars = 20 μm.

Next steps in improving tumor delivery, tumor cell uptake, pharmacokinetics, pharmacodynamics, and clearance may include: (a) active targeting and (b) fine tuning the nanoparticle properties (size, coating, and use of degradable bonds). A commonly used nanoparticle targeting method relies on the Enhanced Permeability and Retention (EPR) effect. Angiogenic endothelium of growing tumors is leaky to 5–200 nm particles which then exit the blood compartment and are retained in the tumor. For more active targeting and cell uptake, adding a receptor ligand or cell penetrating peptide are common approaches. The chemical structure of Niodx supports its linking to amino groups and covalent binding to proteins, peptides, or other molecules. In preliminary tests, we attached transferrin, chosen because it enables nanoparticle penetration across the BBB/BTB and enhances uptake into tumor cells due to their upregulated TfR (transferrin receptors) [29,30,31]. “In vivo study suggested only NPs with Tf conjugation are able to cross through BBB and penetrate into the brain tissues via receptor-mediated endocytosis” [32]. Rapidly dividing cells (e.g., tumor cells) require more iron and typically have upregulated TfR. Transferrin targeting has been shown to be effective in many tumor targeting studies using nanoparticles [29,31,32,33,34,35]. Preliminary tests of Niodx conjugated to Tf indicated 3–5 times greater U87 tumor cell binding and uptake of Niodx in vitro (Figure 14).

### 1.8. Iodine-Enhanced Radiotherapy of Brain Metastases

**Figure 15:**
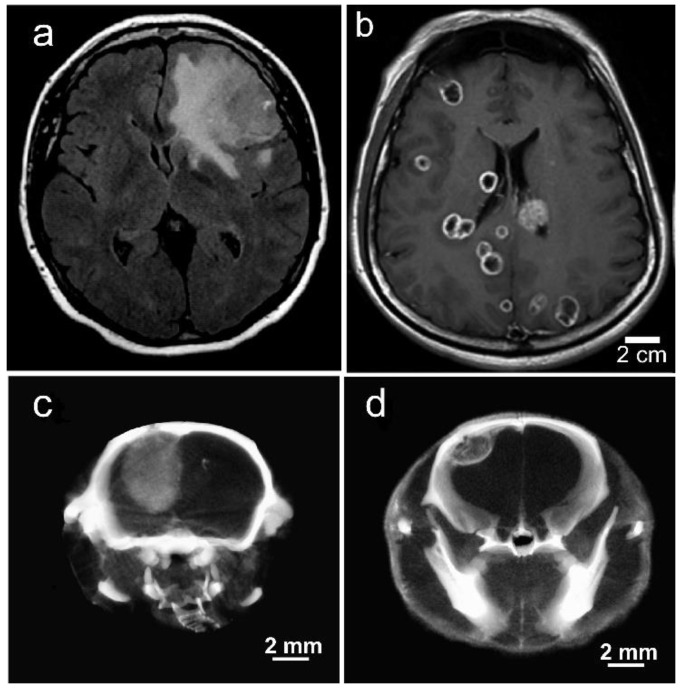
Gliomas and metastatic tumors grow differently in the brain as shown by (**a**) MRI scan of patient with GBM, (**b**) MRI scan of patient with triple negative breast cancer that has metastasized to the brain, (**c**) U87 glioma growing in the brain of an athymic mouse, and (**d**) human triple negative breast cancer growing in the brain of an athymic mouse. (**c**,**d**) are microCT X-ray images (70 kVp) 24 h after IV Niodx injection.

Tumors that metastasize to the brain actually comprise 90% of brain tumors. Metastatic tumors (e.g., lung, breast, and melanoma) grow very differently in the brain compared to gliomas (Figure 15a,b).

Gliomas are highly invasive, expanding and emanating from the origin site. In contrast, metastatic tumors often have multiple contained lesions with well-defined growing edges (Figure 15a,b). Such tumors grown intracranially (IC) in mice showed similar morphologies (Figure 15c,d). They also accumulate Niodx after IV injection and displayed a higher concentration at the growing edge, similar to human brain mets (Figure 15b,d). The intensities seen in the X-ray images are proportional to the iodine concentration. Iodine concentrations were quantified by calibrating the microCT images using iodine standards. The growing edge of the metastatic lesion, such as those seen in Figure 15d, showed an average iodine concentration of 2.9% (*w*/*w*) with many peaks at 4.5%. Monte Carlo calculations indicated this could amplify the radiation dose there approximately 5 to 8 times (Table 1).

**Figure 16:**
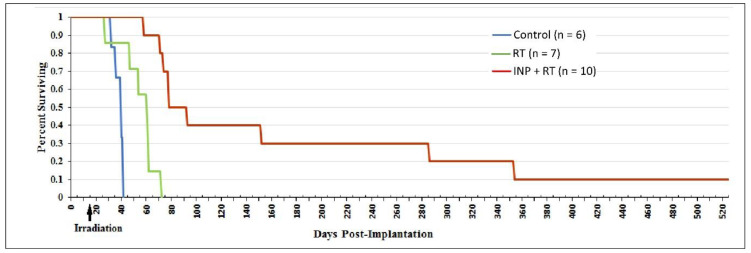
Survival graph showing no treatment (blue), RT only (15 Gy, 100 kVp, green), and INP + RT (red). n denotes number of animals per group. Arrow indicates day of irradiation.

This level of in vivo tumor concentration and dose enhancement has never before been achieved by IV injection. To test the resultant therapeutic benefit, brains were irradiated one day after IV injection. Results are shown in Figure 16 [11].

With RT alone, all animals died within 72 days; Niodx pretreatment resulted in extraordinary longer-term remissions, with 40% of mice surviving 150 days and 30% surviving > 280 days.

### 1.9. Iodine Nanoparticle Targeting to Brain Metastases

**Figure 17:**
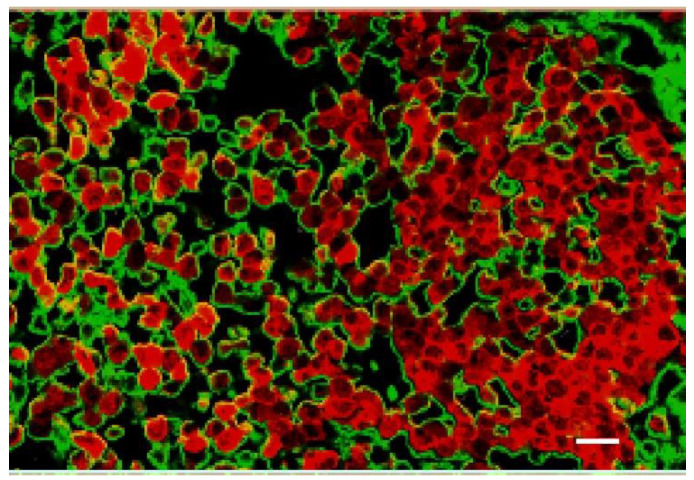
Confocal cryosection image of advanced human triple negative breast cancer tumor (MDA-MB-231) growing in athymic mouse brain 24 h after IV administration of Niodx. Tumor cells have been transduced with red fluorescent protein (td-tomato, red), and labeled for Niodx using an anti-PEG antibody (Alexa-488, green). No endothelial cells are present, and black spaces surrounded by tumor cells (red) are vascular channels formed by the tumor cells mimicking endothelial cells. Niodx shows strong binding to these channels [37]. Bar = 20 μm.

Active targeting can significantly improve tumor uptake. For example, coating nanoparticles with gallic acid (probably self-polymerized) was recently shown to increase binding to orthotopic GL261 gliomas in mice from 3.6% ID/g to 13.5% (3.75-fold) compared to PEG coated NPs [36]. For Niodx, an interesting interaction was discovered where the iodine nanoparticle specifically bound to human triple negative breast cancer (TNBC) tumors growing in the brains of athymic mice [37]. This specific binding increased iodine loading concentrations from about 1% iodine for U87 gliomas to 3% (average tumor concentration at the growing edge) for the 231 TNBC growing in the mouse brain [11,27]. The nature of this molecular binding is still under investigation. It appears that the PEG on the Niodx binds specifically to vascular mimicry surfaces expressing collagen-1, which are not found in the rest of the brain (Figure 17).

Interestingly, in this breast to brain metastasis model, there was an abundance of vascular mimicry typified by irregularly shaped spaces (ISS [37]) instead of normal, more circular endothelial cell-lined tumor vessels (cf. Figure 12). This may partially explain why anti-angiogenic therapies, such as bevacizumab, a vascular endothelial growth factor inhibitor targeting endothelial cells, are poorly effective when used to treat some tumors.

### 1.10. Optimal X-ray Energy

**Figure 18:**
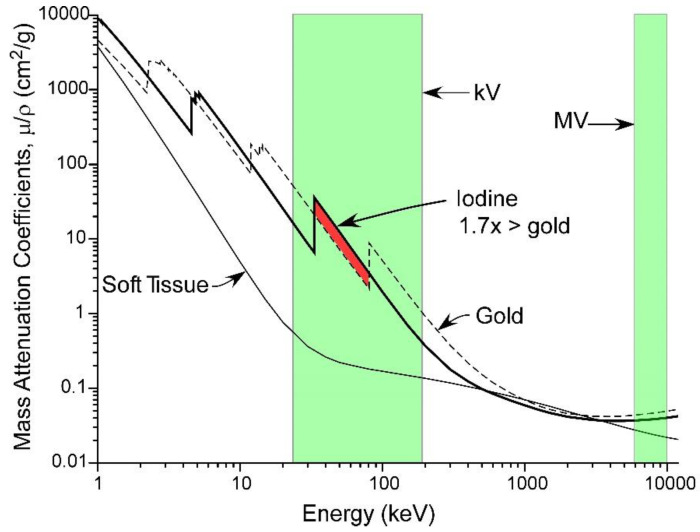
Absorption of iodine vs. gold and soft tissue. In a useful range, iodine absorbs 1.7 times more than gold and at iodine’s K-edge, it absorbs ~100 times more than tissue (on a weight basis). Absorption of high-Z elements compared to tissue is better in the kV range rather than the MV range.

It should be noted that the best absorption of X-rays for iodine compared to tissue occurs in the ‘orthovoltage’ range (<400 kV, Figure 18). In a very useful region, iodine absorbs better than gold.

All hospital X-ray imaging uses orthovoltage, such as CT scanners, imaging for heart stenting or bypasses, vascular surgery, orthopedic operations, chest X-rays, and mammography. However, most radiotherapy is performed with 6–10 million volt (MV) machines that minimize skin entrance dose absorption and have better body penetration. From Figure 18 it can be seen that high-Z elements have little advantage over soft tissue at MV energies. Nevertheless, a number of studies have shown radioenhancement of heavy element nanoparticles at these high energies, perhaps due to more complex physical, chemical, or biological interactions [9]. This points to the difficulty in comparing radiation enhancers, as effects depend on many parameters that can be optimized.

## 2. Materials and Methods

The Nanotechnology Characterization Laboratory has developed a standardized analytical cascade that performs physicochemical characterization as well as preclinical testing of the immunology, pharmacology, and toxicology properties of nanoparticles. Protocols, with explicit materials and methods for each of the tests reported here, are available online [38].

## 3. Results and Discussion

### 3.1. National Cancer Institute Nanotechnology Characterization Laboratory Study of Niodx

For clinical use, the iodine nanoparticles must be well-characterized and shown to be safe at useful doses. To provide these data for promising nanoparticles, the National Cancer Institute created the Nanotechnology Characterization Laboratory (NCL, https://ncl.cancer.gov/ (accessed on 28 December 2021)). Niodx was submitted to them for testing. Over a period of about 3 years, comprehensive tests were performed; some of these studies overlapped with safety studies that we performed previously [21]. The complete report (193 pages) of the Iodine nanoparticles (Niodx, also designated NCL388 in the NCL report) is included in the Supplementary Materials. The nanoparticles were characterized by physicochemical properties, in vitro toxicity in several cell lines, in vitro hematological compatibility, and in vivo pharmacokinetic properties. The most significant findings from these studies are summarized below.

#### 3.1.1. Sterility, Endotoxin, and Beta-Glucans

Niodx was evaluated for sterility and endotoxin contamination before other biological assays were conducted. Samples for testing had no bacterial contamination. Endotoxin levels were initially at approximately 1 EU/mg of iodine as detected by the turbidity and chromogenic Limulus Amebocyte Lysate (LAL) assays. Filtration using Mustang E-filters was able to reduce these levels by about 10-fold. Despite this reduction, however, the current levels were still above the calculated allowable endotoxin limit due to the high dose intended for clinical use (7 g/kg). Adjusting the dose to 291.7 mg/kg/h and increasing the infusion time to 24 h would allow for delivery of the intended 7g/kg dose to humans without exceeding the threshold pyrogenic dose of 5 EU/kg/h, provided the endotoxin level in the formulation does not exceed 0.017 EU/mg.

#### 3.1.2. Physicochemical Characterization

The physicochemical properties of Niodx, including size and size distribution, molecular weight, zeta potential, iodine concentration, iohexol concentration, and PEG concentration, were evaluated using a variety of techniques.

The hydrodynamic size was measured by dynamic light scattering (DLS). The volume-weighted peak sizes were in agreement with the reported values and were in the range of 11–22 nm. There was good batch-to-batch consistency noted for most lots with respect to size. Particle size and molecular weight for Niodx were also measured by asymmetric-flow fractionation (AF4) and size exclusion chromatography coupled with multiple angle light scattering (MALS) and dynamic light scattering (DLS) detectors. The AF4-DLS measured average size was 21 nm, consistent with batch-mode DLS measurements. The molar mass based on the refractive index signal was 142 kDa and 163 kDa based on the AF4 and SEC results, respectively.

The iodine concentration of Niodx was determined by inductively coupled plasma mass spectrometry (ICP-MS). Iodine concentrations were in agreement with the sponsor’s reported values. Importantly, a microwave step is required for complete digestion and accurate quantitation of the iodine in the Niodx samples. The PEG+linker and iohexol concentrations were determined using thermogravimetric analysis (TGA). The iohexol and PEG+linker concentrations were 68.7% (*w*/*w*) and 28.7% (*w*/*w*), respectively. The zeta potential was neutral under the measurement conditions utilized. This was expected due to the composition of the formulation.

#### 3.1.3. In Vitro Toxicity Studies

The toxicity of Niodx was evaluated in vitro using two cell lines, porcine renal proximal tubule (LLC-PK1), and human hepatocarcinoma (Hep G2) cells. The formulation showed greater toxicity to the LLC-PK1 cells than the Hep G2 cells, with estimated IC50 values of 0.89 and 5.03 mg/mL, respectively. In addition, Niodx induced autophagic dysfunction in the LLC-PK1 cells at cytotoxic concentrations, and this is a potential mechanism of cell death. However, in vivo tests did not reveal such toxicities.

#### 3.1.4. In Vitro Immunological Characterization

The hematotoxicity of Niodx was assessed in vitro using freshly drawn human blood. In brief, Niodx was not hemolytic, did not induce complement activation or platelet aggregation, and did not affect collagen-mediated platelet aggregation at the tested concentrations. The formulation did exhibit prolongation of plasma coagulation times in both the thrombin and activated partial thromboplastin time assays. This finding is consistent with the known effects of iohexol on blood coagulation [39].

Interestingly, NCL388 induced chemokine response in human PBMC. As chemokines function to recruit immune cells, the data suggest a potential utility of NCL388 to improve the efficacy of traditional immunotherapeutics (e.g., anti-PD1 and anti-CTLA4). The role of these chemokines in the safety of NCL388 is currently unknown. Aas IL-8 is known as one of pyrogenic markers, an elevation of body temperature in sensitive individuals may be observed after the administration of NCL388. However, the risk of pyrogenicity does not appear high, because other cytokines with more prominent roles in the fever response (TNFα, IL-1β, and IL-6) were not induced by NCL388. These findings warrant additional investigation.

#### 3.1.5. Multidose Toxicity Study (ADME-Tox)

The intravenous multidose toxicity study compared two dose levels, administered for four consecutive daily doses (qdx4) (cumulative dose of 855 and 1715 mg I/kg, respectively), and a saline control. Each dose level and saline control contained a main group (assayed 1 day after the last dose) and a recovery group (assayed 14 days after the last dose). The administration of Niodx resulted in multisystemic histiocytosis of all organs as confirmed by immunohistochemistry (IHC). Histiocytic infiltrate was dose-dependent, and progressed in recovery groups. The histiocytosis noted is most likely the result of iodine nanoparticle accumulation in tissue macrophages, as has been shown for other biopersistent polymers such as PEGylated therapeutics (Irizarry et al., 2018). Other histopathological changes were not observed, nor were alterations in hematological or clinical chemistry parameters that would indicate toxicity.

Spleen and liver weights were increased compared to the control in both treated main and recovery groups, with weights greater in the recovery groups as compared to the main groups. In the liver, TEM analysis showed intracytoplasmic vacuoles present within Kupffer cells and sinusoidal endothelial cells, consistent with iodine polymer uptake. The increase in organ weights was associated with histiocytosis in the absence of other significant findings. Mild neutrophilia was also present in all treated groups, with the main groups being less severe than the recovery groups. There were no other findings of biological significance noted for this study.

## 4. Conclusions

A novel iodine nanoparticle (Niodx) is described with unique properties. In particular, it is a 20 nm nanoparticle with a long blood half-life of 40 h and is well-tolerated at an intravenous dose of 7 g iodine/kg (in mice). It accumulates in gliomas and brain metastases and provides robust tumor imaging, potentially useful for diagnostic purposes and alignment for surgeries or radiotherapy. High tumor loading with iodine results in absorbing more X-rays during radiotherapy, thus boosting the local dose at the tumor several-fold, which is shown to provide significant life extension compared to radiotherapy alone. It is a promising new treatment for gliomas and other cancers. Extensive pharmacological testing has shown that Niodx appears safe for in vivo use.

## Figures and Tables

**Table 1:** Iodine uptake and calculated Dose Enhancement Factor (DEF) at 72 h after IV injection of 7 g I/kg Niodx for triple negative breast cancer (TNBC) growing in brains of mice. Regions measured: tumor center, tumor growing edge, peak regions of growing edge, expressed as mean ± standard deviation.

	Tumor Center (Average)	Growing Edge (Average)	Peaks at Growing Edge (Average)
%Iodine	1.13 ± 0.35	2.9 ± 0.54	4.52 ± 0.07
DEF	2.77	5.48	8.01

## Data Availability

Data are contained within the article or supplementary material.

## Supplementary Materials

The following is available online at https://www.mdpi.com/article/10.3390/pharmaceutics14030508/s1, NCL Niodx study report.
