# Supplementary material for: Iodine Nanoparticles (Niodx™) for Radiotherapy Enhancement of Glioblastoma and Other Cancers: An NCI Nanotechnology Characterization Laboratory Study
Source: Pharmaceutics. 2022 Feb 25;14(3):508. doi: 10.3390/pharmaceutics14030508 (PMC8955506; doi:10.3390/pharmaceutics14030508)
Supplement: Supplementary file 1 [file pharmaceutics-14-00508-s001.zip › pharmaceutics-1553895-supplementary.pdf]

NCL202105A

Niodx™

prepared for

Nanoprobes

Nanotechnology Characterization Laboratory
Frederick National Laboratory for Cancer Research
Leidos Biomedical Research, Inc.
Frederick, MD 21702
(301) 846-6939 • ncl@mail.nih.gov
http://ncl.cancer.gov

This report contains proprietary and confidential information. Any use, dissemination, distribution, or copying of this report or its contents requires written permission from the National Cancer Institute.

TABLE OF CONTENTS

Objective 3

Executive Summary 5

I. Sterility, Endotoxin and Beta-Glucans 7

Sterility (STE-2.4) 8
Endotoxin (STE-1) 9
Beta-Glucans 13

II. Physicochemical Characterization 14

Hydrodynamic Size/Size Distribution via Dynamic Light Scattering (PCC-1) 15
Zeta Potential (PCC-2) 32
Polymer Molecular Weight by SEC-MALS 39
Size Distribution and Molecular Weight by Asymmetric-Flow Field Flow Fractionation 42
Total Iodine Concentration by Inductively Coupled Plasma-Mass Spectrometry 45
Total Iodine Concentration by UV-Vis Spectroscopy 50
Iohexol and PEG Concentration by Thermogravimetric Analysis 52

III. In Vitro Cytotoxicity 58

LLC-PK1 Cytotoxicity Assays, MTT and LDH Release (GTA-1) 59
Hep G2 Cytotoxicity Assay, MTT and LDH Release (GTA-2) 61
Autophagic Dysfunction in LLC-PK1 Cells 63

IV. In Vitro Immunological Characterization 67

Selection of Concentrations 68
Hemolysis (ITA-1) 69
Platelet Aggregation (ITA-2.1) 70
Plasma Coagulation Times (ITA-12) 72
Complement Activation (ITA-5.2) 74
Cytokines (ITA-10) 75

V. Multidose In Vivo Toxicology Study in CD-1 Mice (ADME Tox 193) 78

Design and Methods 80
In-Life Results 82
Necropsy Results 84

Appendix 124

Appendix A. Molecular Histopathology Laboratory Pathology Narrative (ADME-Tox 193) 124
Appendix B. Individual Animal Data (ADME-Tox 193) 134
Appendix C. Randomization Report (ADME-Tox 193) 155
Appendix D. Study Protocol (ADME-Tox 193) 160
Appendix E. EDX Analysis of Tissue Samples 163
Appendix F. ICP-MS Analysis of Tissue Samples 165
Appendix G. ICP-MS Analysis of Tissue Samples 178

References 187

Abbreviations 189

Contributors and Acknowledgements 191

OBJECTIVE

The objective of the NCL–Nanoprobes collaboration was characterization of their iodine nanoparticle formulation, Niodx™. Niodx is comprised of an existing FDA approved contrast agent, Omnipaque™ (iohexol), which has been chemically crosslinked and PEGylated to form a nanoparticle. This novel iodine nanoparticle formulation, termed NCL388 herein, is being developed as a radiosensitizer to increase tumor radiation dosage in tumors in glioma therapy.

The sample details for each batch received, as provided by Nanoprobes, are listed below.

NCL388: Iodine Nanoparticles (Niodx™)

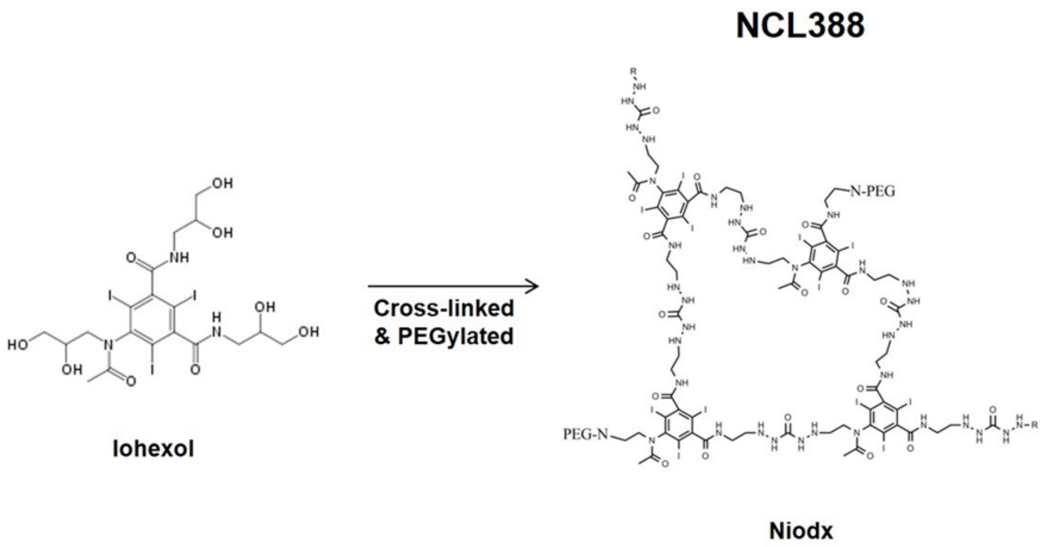

Image adapted from sponsor's application materials.

NCL388-1: Lot 4/15/18-2
Quantity/Concentration received: 1 vial @ ~4.5 mL (in PBS), 65 mg I/mL
Received 05/09/2018

NCL388-2:
Quantity/Concentration received: 1 vial @ ~2.2 mL (in PBS)
Received 07/31/2018

NCL388-3:
Quantity/Concentration received: 1 vial @ ~2.5 mL and 3 vials @ 3.75 mL (in PBS), 70 mg I/mL
Received 12/11/2018

NCL388-4:
Quantity/Concentration received: 2 vials @ ~5 mL, 3 vials @ ~4.5 mL and 5 vials @ 3.75 mL (in PBS), 70.02 mg I/mL
Received 03/06/2020

NCL388-5:
Quantity/Concentration received: 1 vial @ ~2.5 mL, 1 vial @ ~3.75 mL, 5 vials @ ~4 mL and 5 vials @ ~4.25 mL (in PBS), 73.58 mg I/mL
Received 03/06/2020

NCL388-6:
Quantity/Concentration received: 1 vial @ ~27.5 mL (in PBS), 68.26 mg I/mL
Received 08/18/2020

NCL388-7:
Quantity/Concentration received: 1 vial @ ~3.8 mL (in PBS), 70.3 mg I/mL
Received 08/18/2020

NCL388-8:
Quantity/Concentration received: 1 vial @ ~19 mL (in PBS), 70 mg I/mL
Received 09/22/2020

NCL388-9:
Quantity/Concentration received: 1 vial @ ~21 mL (in PBS), 68.5 mg I/mL
Received 09/22/2020

NCL388-10:
Quantity/Concentration received: 1 vial @ ~19.5 mL (in PBS), 69 mg I/mL
Received 09/22/2020

NCL388-11:
Quantity/Concentration received: 2 vials @ ~15 mL (in PBS), 76 mg I/mL
Received 09/22/2020

All samples were stored at -20°C upon receipt.

NCL388-12:
Several samples were combined to form batch NCL388-12; these lots were NCL388-4, NCL388-5, NCL388-6, NCL388-9, NCL388-10, NCL388-11. This sample was used for the in vivo tox study as well as the in vitro cytokine analysis.

The Niodx nanoparticles (NCL388) were characterized for physicochemical properties, in vitro toxicity in several cell lines, in vitro hematological compatibility, as well as in vivo pharmacokinetic properties. The most significant findings from these studies are summarized below.

Sterility, Endotoxin and beta-glucans

NCL388 was evaluated for sterility and endotoxin contamination before other biological assays were conducted. Of the nine batches screened, only one sample was found to have bacterial contamination, NCL388-8. This sample was excluded from further analyses. Endotoxin levels were initially at approximately 1 EU/mg of iodine as detected by the turbidity and chromogenic Limulus Amebocyte Lysate (LAL) assays. Filtration using Mustang E-filters was able to reduce these levels by about 10-fold. Despite this reduction, however, the current levels are still above the calculated allowable endotoxin limit due to the high dose intended for clinical use (7g/kg). Adjusting the dose to 291.7 mg/kg/hr and increasing the infusion time to 24 hr would allow for delivery of the intended 7g/kg dose without exceeding the threshold pyrogenic dose of 5 EU/kg/h, provided the endotoxin level in the formulation does not exceed 0.017 EU/mg. It is suggested that the starting materials be filtered through the Mustang-E filters prior to particle synthesis under pyrogen-free conditions to help control endotoxin levels in the formulation. Beta-glucan levels were also tested in select lots. The levels of beta-glucans are not regulated by the US FDA; NCL performs this analysis for informational purposes only.

Physicochemical Characterization

The physicochemical properties of NCL388, including size and size distribution, molecular weight, zeta potential, iodine concentration, iohexol concentration, and PEG concentration, were evaluated using a variety of techniques.

The hydrodynamic size was measured by dynamic light scattering (DLS). The volume-weighted peak sizes were in agreement with the reported values and were in the range of 11-22 nm (Tables II-1, II-4 to II-5, II-7 to II-15). Of note, several batches were measured, and there was good batch-to-batch consistency noted for most lots with respect to size. Particle size and molecular weight for NCL388 were also measured by asymmetric-flow field flow fractionation (AF4) and size exclusion chromatography coupled with multiple angle light scattering (MALS) and dynamic light scattering (DLS) detectors. The AF4-DLS measured average size was 21 nm (Figure II-23B), consistent with batch-mode DLS measurements. The molar mass based on the refractive index signal was 142 kDa (Figure II-23) and 163 kDa (Figure II-22) based on the AF4 and SEC results, respectively.

The iodine concentration of NCL388 was determined by inductively coupled plasma mass spectrometry (ICP-MS). Iodine concentrations were in agreement with the sponsor's reported values (Tables II-24 and II-25). Importantly, a microwave step is required for complete digestion and accurate quantitation of the iodine in the NCL388 samples. The PEG+linker and iohexol concentrations were determined using thermogravimetric analysis (TGA). The iohexol and PEG+linker concentrations were 68.7 %(w/w) and 28.7 %(w/w), respectively (Table II-27). The zeta potential was neutral (Figures II-16 to II-21) under the measurement conditions utilized. This was expected due to the composition of the formulation.

In Vitro Toxicity Studies

The toxicity of NCL388 was evaluated in vitro using two cell lines, porcine renal proximal tubule (LLC-PK1) and human hepatocarcinoma (Hep G2) cells. The formulation showed greater toxicity to the LLC-PK1 cells than the Hep G2 cells, with estimated IC_{50} values of 0.89 and 5.03 mg/mL, respectively (Figures III-1 and III-2). In addition, NCL388 induced autophagic dysfunction in the LLC-PK1 cells (Figures III-3 to III-5) at cytotoxic concentrations, and this is a potential mechanism of cell death.

In Vitro Immunological Characterization

The hematotoxicity of NCL388 was assessed in vitro using freshly drawn human blood. In brief, NCL388 was not hemolytic, did not induce complement activation or platelet aggregation, and did not affect collagen-mediated platelet aggregation at the tested concentrations. The formulation did exhibit prolongation of plasma coagulation times in both the thrombin and activated partial thromboplastin time assays. This finding is consistent with the known effects of iohexol on blood coagulation [4].

Interestingly, NCL388 induced chemokine response in human PBMC. Since chemokines function to recruit immune cells, the data suggest a potential utility of NCL388 to improve the efficacy of traditional immunotherapeutics (e.g., anti-PD1 and anti-CTLA4). The role of these chemokines in safety of NCL388 is unknown at the moment. Since IL-8 is known as one of pyrogenic markers, an elevation of body temperature in sensitive individuals may be observed after the administration of NCL388. However, the risk of pyrogenicity does not appear high because other cytokines with more prominent role in the fever response (TNFα, IL-1β and IL-6) were not induced by NCL388. These findings warrant additional investigation.

Multidose Toxicity Study (ADME-Tox 193)

The intravenous multidose toxicity study compared two dose levels, administered for 4 consecutive daily doses (qdx4) (cumulative dose of 855 and 1715 mg I/kg, respectively), and a saline control. Each dose level and saline control contained a main group and a recovery group. Administration of NCL388 resulted in immunohistochemistry (IHC)-confirmed multisystemic histiocytosis in all organs. Histiocytic infiltrate ranged from mild to severe, was dose-dependent, and progressed in recovery groups.

Spleen and liver weights were also increased over control in both treated main and recovery groups, with weights greater in the recovery groups as compared to the main groups. In the liver, TEM analysis showed intracytoplasmic vacuoles present within Kupffer cells and sinusoidal endothelial cells, consistent with iodine polymer uptake. The increase in organ weights was associated with severe histiocytosis in the absence of other significant findings. Mild neutrophilia was also present in all treated groups, with the main groups being less severe than the recovery groups. There were no other findings of biological significance noted for this study.

I. Sterility, Endotoxin and Beta-Glucans

Section Summary

The purpose of these studies was to screen NCL388 for potential contamination with bacteria, endotoxin and beta-glucans. Sterility testing was performed by plating the samples on agar plates. Of the nine batches screened, only one sample was found to have bacterial contamination, NCL388-8. This sample was excluded from further analyses.

Two assays were used for the detection and quantification of endotoxin, kinetic turbidity and chromogenic Limulus Amebocyte Lysate (LAL) assays. Analysis of the first batch, NCL388-1, showed approximately 1 EU/mg iodine; the amounts in second and third batches were lower, while in the fourth and fifth batches were higher than in the first batch. It is unknown what synthesis conditions or starting materials contributed to the increased contamination in NCL388-4 and NCL388-5. Subsequent batches showed endotoxin levels in the range of approx. 0.01-0.3 EU/mg iodine (Table I-1).

Despite the relatively low endotoxin level in the NCL388-1 formulation, it represented a concern due to the high dose of the formulation. Adjusting the dose to 291.7 mg/kg/hr and increasing the infusion time to 24 hr would allow for delivery of the intended 7g/kg dose without exceeding the threshold pyrogenic dose of 5 EU/kg/hr, provided the endotoxin level in the formulation does not exceed 0.017 EU/mg.

In an attempt to reduce endotoxin levels, Nanoprobes prepared two additional batches which were filtered using Mustang E-filters (NCL388-2 and NCL388-3). NCL also performed additional filtrations on NCL388-2, as well as filtered NCL388-1. The data demonstrated that one pass though the filtration apparatus resulted in a significant (~10-30 fold) reduction of endotoxin, while subsequent rounds of filtration did not provide additional benefit. Since filtration may also affect the particle's physicochemical properties, it is not known whether the filtration of the final formulation would be beneficial for this product. It may be more practical, safer and reasonable to first screen the starting materials for endotoxin, filter those that contain endotoxin through the Mustang-E filters, then proceed to particle assembly using all currently known pyrogen-free precautions.

The amounts of beta-glucans were measured in batches NCL388-4 to NCL388-11 (excluding NCL388-8), for informational purposes only. With the current levels of beta-glucans in the formulation, injecting NCL388 at a dose of 7 g of iodine per kg would result in blood levels of beta-glucan above those normally present in the blood from dietary sources. To understand potential effects of the beta-glucans on the safety of NCL388, more studies are necessary. Monitoring beta-glucan levels would also be helpful in assessing batch-to-batch consistency of the formulation.

Sterility (STE-2.4)

Design and Methods

Sterility, i.e. bacterial contamination, was assessed using NCL protocol STE-2.4 (https://ncl.cancer.gov/resources/assay-cascade-protocols). In brief, samples were plated onto TS agar plates at several dilutions (stock, 1:10, 1:100, and 1:1000) and allowed to incubate at 37°C for 72 hours. The plates were then visually inspected for colony formation. In addition, 100 µL of the stock was spiked into 5 mL of RPMI (no FBS) media and incubated for 7 days at 37ºC. The flasks were then visually inspected for turbidity/bacterial growth.

Results & Conclusions

NCL388-1 and lots NCL388-4 through NCL388-11 were screened for microbial contamination. One of the nine tested lots showed bacterial contamination, NCL388-8. The contamination was estimated at 3980 CFU/mL (Figure I-1).

None of the other eight lots showed visible colony formation on the agar plates at the tested dilutions after three days. The RPMI flasks also did not show any signs of turbidity/bacterial growth.

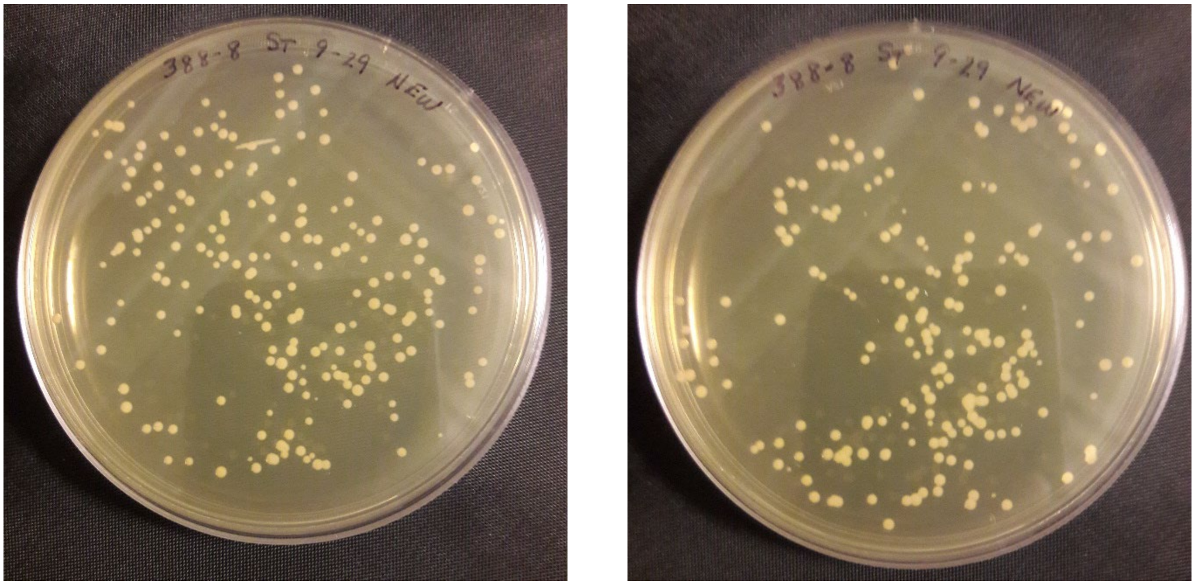

Figure I-1. Bacterial Contamination in NCL388-8. NCL388-8 was plated on TS agar plates and incubated at 37°C for 72 hours. The contamination was estimated at 3980 CFU/mL.

Endotoxin (STE-1)

Design and Methods

The objective of this experiment was to evaluate potential endotoxin contamination in NCL388. NCL protocols for kinetic chromogenic Limulus Amebocyte Lysate (LAL) and turbidity LAL (STE-1.4 and STE-1.2 respectively, https://ncl.cancer.gov/resources/assay-cascade-protocols) were used.

United States Pharmacopeia (USP) standard BET85 was followed to calculate the endotoxin limit (EL) and maximum valid dilution (MVD) [1]. The EL was calculated according to the formula:

$$EL = K/M$$

where K is threshold pyrogenic dose of endotoxin and M is the maximum dose administered per kilogram of body weight per hour. The K-value for all routes of administration, except intrathecal, is 5 EU/kg/hr. The maximum dose (M) for NCL388 was calculated using the efficacious dose of 7 g/kg, as reported by the sponsor. This maximum tested mouse dose was converted into a human equivalent dose (HED) using the formula shown below, to provide the maximum dose (M) of 569 mg/kg [2].

$$HED = \text{Mouse Dose} \div 12.3$$

$$M = 7000 \text{ mg/kg} \div 12.3 = 569 \text{ mg/kg}$$

Therefore,

$$EL = K/M$$

EL = 5 EU/kg/hr ÷ 569 mg/kg = **0.01 EU/mg/hr at an HED of 569 mg/kg**

Although the 7 g/kg dose was the maximum tested in a mouse model, Nanoprobes indicated they may also test as high as 7 g/kg in humans. Therefore, we also calculated the endotoxin limit using 7 g/kg:

Therefore,

$$EL = K/M$$

EL = 5 EU/kg/hr ÷ 7000 mg/kg = **0.0007 EU/mg/hr at an HED of 7000 mg/kg**

This high dosage may pose problems for endotoxin testing using traditional LAL assays, as the most sensitive LAL assay will only detect as low as 0.001 EU/mg. Nanoprobes may have to utilize other methods for assessing pyrogenicity, such as the rabbit pyrogen test. Alternatively, the formulation may be administered over a longer time period (e.g., 24 hr). Using a 24 hours infusion would allow administering the intended dose of 7g/kg without exceeding the threshold pyrogenic dose of 5 EU/kg/hr provided the endotoxin in the formulation does not exceed 0.017 EU/mg.

The maximum valid dilution (MVD) was then calculated for the LAL assay, using the endotoxin limit from the scaled mouse dose. The MVD applicable for this formulation was calculated according to the formula:

$$MVD = (EL \times \text{sample concentration})/\lambda$$

where λ is the sensitivity of the LAL assay (0.001 EU/mL for kinetic turbidity and chromogenic LAL and 0.03 EU/mL for the gel-clot LAL). Here, the theoretical API concentration as provided by the sponsor was used in calculation of the MVD (70 mg I/mL). Samples were tested at the MVD and several concentrations lower than the MVD.

Therefore,

MVD = (0.01 EU/mg * 70 mg/mL) ÷ 0.001 EU/mL = 700-fold for turbidity and chromogenic LAL

MVD = (0.01 EU/mg * 70 mg/mL) ÷ 0.03 EU/mL = 23.3-fold for gel clot LAL

Using the endotoxin limit from the non-scaled mouse dose provided maximum valid dilutions of:

MVD = (0.0007 EU/mg * 70 mg/mL) ÷ 0.001 EU/mL = 49-fold for turbidity and chromogenic LAL

MVD = (0.0007 EU/mg * 70 mg/mL) ÷ 0.03 EU/mL = 1.6-fold for gel clot LAL

Here too, Nanoprobes may encounter issues using the LAL assay for endotoxin testing. The particles were found to interfere with the turbidity and chromogenic assays at dilutions lower than 1:60. This finding also suggests that an alternate means of assessing pyrogenicity or extending infusion time may be required at the currently intended human dose of 7 g/kg.

Results and Discussion

Endotoxin levels in NCL388-1 and NCL388-3 through NCL 388-11 were measured using the turbidity and chromogenic LAL assays. Analysis of NCL388-2 used the turbidity LAL only.

Both assays detected approximately 1 EU/mg iodine in NCL388-1 (Table I-1). Endotoxin in subsequent batches were all lower than this initial batch, with values ranging 0.008-0.83 EU/mg iodine. Since the intended clinical dose of the formulation is so high, even at 1 EU/mg, the endotoxin in the formulation is orders of magnitude greater than the acceptable limit for clinical use. Using a human equivalent dose of 569 mg/kg, the calculated allowable EL is 0.01 EU/mg. Using this EL value, only batch NCL388-10 has endotoxin levels below this limit. Using a human dose of 7000 mg/kg, the calculated allowable EL is 0.0007 EU/mg.

As an alternate approach, increasing the infusion time may permit delivery of a larger dose without exceeding the allowable threshold pyrogenic dose. For example, adjusting the dose to 291.7 mg/kg/hr and increasing the infusion time to 24 hr would permit delivery of a 7g/kg dose during the 24 hr time period, provided that the amount in NCL388 does not exceed 0.017 EU/kg.

In an attempt to reduce endotoxin levels, Nanoprobes tested the use of Mustang E-filters (batch NCL388-2 was newly synthesized and filtered prior to shipping to NCL). The result was about a 30-fold reduction in the amount of detectable endotoxin in the sample as compared to the first batch. However, since this was a new batch of material, it was not clear whether the Mustang filters were responsible for the reduced endotoxin levels or whether the preparation procedure was improved. Therefore, the NCL performed additional passes of NCL388-2 through Mustang E-filters. There was no significant change in endotoxin levels as compared to the original batch sent. NCL also performed one additional test, namely Mustang E-filtration of NCL388-1. This showed about a 10-fold reduction in endotoxin levels. A third batch, NCL388-3 which was also filtered using the Mustang E-filters, had 0.08 EU/mg. This batch was used for physicochemical and in vitro biological studies. Batches NCL388-4, NCL388-5, NCL388-6, NCL388-9, NCL388-10 and NCL388-11 were pooled together for the in vivo toxicology and in vitro cytokine studies. This combined batch is referred to as NCL388-12.

In summary, filtration of the samples using Mustang E-filters was shown to reduce endotoxin levels in the formulation. Furthermore, it appears that one pass through the filtration device was sufficient and that multiple passes did not further improve (reduce) the endotoxin levels. However, it is unclear whether introducing Mustang E-filtration into a manufacturing process would provide a benefit. It appears to be a more practical, safer and reasonable approach to first screen starting materials for endotoxin, filtering those that contain endotoxin before proceeding to the particle assembly using all currently known pyrogen-free precautions.

Table I-1. Endotoxin levels detected by the LAL assay. Results are shown as endotoxin units (EU) per mg of iodine. NT = not tested. EL = 0.017 EU/mg I, applicable at a human equivalent dose of 7 g/kg/ 24 hr.

Sample	Turbidity LAL (% Spike Recovery)	Chromogenic LAL (% Spike Recovery)
NCL388-1	1.01 (116%)	1.04 (87%)
NCL388-1 (Mustang filtered at NCL)	0.13 (122%)	NT
NCL388-2	0.036 (102%)	NT
NCL388-2 (Mustang filtered at NCL)	0.027 (183%)	NT
NCL388-3	0.08 (170)	0.06 (100)
NCL388-4	1.95 (121)	4.5 (82)
NCL388-5	0.83 (150)	1.94 (135)
NCL388-6	0.12 (160)	0.05 (84)
NCL388-7	0.04 (172)	0.33 (97)
NCL388-8	0.12 (125)	< 0.3 (103)
NCL388-9	0.3 (113)	0.8 (154)
NCL388-10	0.008 (73)	< 0.03 (65)
NCL388-11	0.15 (124)	< 0.3 (77)

Beta-Glucans

Design and Methods

The objective of this experiment was to evaluate the levels of beta glucans in NCL388-4 and NCL388-5. NCL Protocol STE-4.1 (https://ncl.cancer.gov/resources/assay-cascade-protocols) was performed on multiple dilutions (1:5, 1:50, 1:500) of the stock concentration.

Results and Discussion

Beta-glucan levels were measured in lots NCL388-4 through NCL388-11. For batches NCL388-4 through NCL388-7, levels were detected at values ranging 17.6-33.1pg/mg of iodine. In subsequent batches, beta-glucan levels were higher. NCL388-10 and NCL388-11 had beta glucans levels roughly double those in the earlier batches, 47.8 and 61.3 pg/mg iodine, respectively. NCL388-9 had the highest detected level, at 126.3 pg/mg iodine.

Beta-glucan levels are of interest for biotechnology therapeutics and for complex formulations containing components with potential immunogenicity concerns. While there is no formally mandated threshold pyrogenic dose for beta-glucans, this contaminant may affect nanoparticle safety and efficacy profiles. Therefore, NCL performs the analysis of beta-glucans along with detection of endotoxin contamination. Monitoring beta-glucan levels is also helpful in assessing batch-to-batch consistency. Due to the high dose of NCL388, the detected levels of beta-glucan would result in blood concentrations exceeding that present in the blood from dietary sources. More studies are needed to understand the potential effects of this contaminant on the safety of NCL388.

Table I-2. Beta glucan levels. Results are shown as pg of beta glucan per mg of iodine. NT = not tested.

Sample	Beta Glucans, pg/mg iodine (% spike recovery)
NCL388-4	21.9 (59)
NCL388-5	17.6 (50)
NCL388-6	23.7 (50)
NCL388-7	33.1 (53)
NCL388-8	NT
NCL388-9	126.3 (84)
NCL388-10	47.8 (57)
NCL388-11	61.3 (52)

II. Physicochemical Characterization

Section Summary

Twelve batches of the iodine nanoparticle formulations (NCL388), Niodx™, were characterized for hydrodynamic size (diameter) by dynamic light scattering (DLS), size and molecular weight by asymmetric-flow field flow fractionation (AF4), molecular weight by size exclusion chromatography and multiple angle light scattering (SEC-MALS), zeta potential, iodine concentration by inductively coupled plasma mass spectrometry (ICP-MS) and UV-Vis spectroscopy, and ioxehol and PEG+linker concentrations by thermogravimetric analysis (TGA).

The hydrodynamic size was measured in 10 mM NaCl (zeta potential conditions) and PBS (native dispersing medium and to mimic physiological ionic strength). Generally, the intensity-weighted peak sizes were larger than the sponsor-reported sizes. However, the volume-weighted peak sizes were in-line with the reported values. The volume-weighted sizes for all lots, except NCL388-2, were in the range of 11-22 nm (Tables II-1, II-4, II-5, II-7 to II-15), consistent with the sponsor's reported values. The outlier, NCL388-2, was larger compared to all the other lots, ~31 nm.

The zeta potentials were measured in 10 mM NaCl at their native pH and after adjustment to pH 7. All samples, when dispersed in 10 mM NaCl and at both native and neutral pH, exhibit neutral zeta potentials (Figures II-16 to II-21). Zeta potentials ranging +10 to -10 mV are generally considered neutral. This was expected based on the composition of the formulation.

Particle size distribution and molecular weight for NCL388-3 were measured using asymmetric-flow field flow fractionation (AF4) coupled with MALS and DLS detectors. The measured flow-mode DLS size ranged from 15 to 75 nm, with an average size of 21 nm (Figure II-23B). This was consistent with the measured batch-mode DLS results. The molar mass based on the refractive index signal, and hence most abundant species, was 163 kDa. The molecular weight was also determined by size exclusion chromatography and multiple angle light scattering (SEC-MALS). The molar mass based on the refractive index signal, and hence most abundant species, was 142 kDa (Figure II-22C), very consistent with the AF4 results.

The iodine concentration of NCL388 was determined by inductively coupled plasma mass spectrometry (ICP-MS) and UV-Vis spectroscopy. The NCL388-1 iodine concentration (Table II-24) was 65.3 ± 0.3 mg/mL, in very good agreement with the sponsor's theoretical value of 65 mg/mL. Filtration through Mustang filters did not significantly reduce the iodine content (Table II-24). The iodine concentration for NCL338-12, a pooled sample of NCL388-4, NCL388-5, NCL388-6, NCL388-9, NCL388-10, and NCL388-11, was 71.4 ± 10.8 and 65.8 ± 6.0 mg/mL based off of two runs of the two dosing solutions used in the in vivo toxicology study (Table II-25). These results were in the range of the sponsor's reported iodine concentrations for these pooled lots. For NCL388-3 only, the total iodine was measured using UV-vis detection at 240 nm, against a calibration standard of NCL388-1 which was measured by ICP-MS. The total iodine concentration in NCL388-3 was 50.9 ± 2.2 mg/mL (n=5) (Figure II-27), lower than the client reported value of 70 mg/mL

The PEG+linker and iohexol concentrations in NCL388-3 was determined using thermogravimetric analysis (TGA). The iohexol concentration was 68.7 %(w/w) or 163.6 mg/mL (Table II-27). The PEG+linker concentration was 28.7 %(w/w).

Hydrodynamic Size/Size Distribution via Dynamic Light Scattering (PCC-1)

Design and Methods

A Malvern Zetasizer Nano ZS instrument (Southborough, MA) with back scattering detector (173°) was used for measuring the hydrodynamic size (diameter) in batch mode. NIST-NCL joint protocol PCC-1 was followed (https://ncl.cancer.gov/resources/assay-cascade-protocols). Stock samples were diluted 100- and 1000-fold in 10 mM NaCl (zeta potential measurement conditions) and PBS (to mimic physiological ionic strength). Samples were measured at 25°C in a quartz microcuvette. Traces in the figures represent the average of at least twelve measurements, unless otherwise stated.

Hydrodynamic diameters are reported as the intensity-weighted average and as the volume-weighted average over a particular range of size populations corresponding to the most prominent peak. The Int-Peak value is used as the hydrodynamic diameter of a particular species. The Vol-Peak and %Vol values are used to approximate relative amounts of various species in the formulation. Z-Avg values are generally used to assess batch-to-batch size variability of a sample.

Results and Discussion

The hydrodynamic size distributions of NCL388-1 are shown in Figures II-1. When measured in 10 mM NaCl, the intensity-peak diameters were larger than the sponsor's reported size (29 nm vs. 15 nm reported; Figure II-1). The volume-peak diameters, however, more closely matched the reported size, with NCL388-1 having a volume diameter of 12 nm. The sample showed polydispersity (PdI>0.2) at both 100- and 1000-fold dilutions, but was significantly more polydisperse at 1000-fold dilution, possibly suggesting instability at higher dilution. NCL388-1 showed the same general trend when measured in PBS. Although overall, the sample was more polydisperse in PBS than in 10 mM NaCl.

NCL388-1 was also re-measured 4 months later, both pre- and post-filtration using the Mustang E-filters (Figures II-2 and II-3). These measurements provide an assessment of the stability over time, as well as evaluate for effects on the size distribution due to filtration of the sample. In 10 mM NaCl, there was a shift in the intensity distribution with the repeat measurement as compared to the original measurement (63 nm vs. 29 nm), suggesting there may be stability issues over time. This effect was not seen in the volume distribution, however. The primary population remained approx. 12 nm. There was no effect upon filtration of the sample in either the intensity- or volume-distribution curves. In PBS, the trend was similar. There was an increase in size noted upon storage in the intensity distribution (albeit not as large as with 10 mM NaCl), with minimal change noted in the volume distribution curves.

NCL388-2 showed a significantly larger size by intensity as compared to NCL388-1 (Figure II-4). The sizes were 80-110 nm for the second batch, as compared to 25-30 nm for the first batch. The volume distribution traces were also broader and suggestive of multiple populations with a shoulder visibly evident in the curves. This batch was not used for in vitro studies due to the size discrepancy.

The size distribution for NCL388-3 more closely matched that of the initial batch and the sponsor's expected values (Figure II-5). The intensity peak ranged 25-35 nm and the volume peak ranged 15-20 nm. There was some size difference noted upon dilution, most evident in 10

mM NaCl. There was an emergence of a population >1000 nm and a slight shift in the volume distribution from 15 to 20 nm.

Figure II-6 provides an overlay of all three batches, at 100-fold dilution in PBS. From this figure, it is evident that NCL388-2 was an outlier with regards to size distribution. NCL388-1 and NCL388-3 were very similar. NCL388-3 was therefore used in most in vitro biological studies (see Sections III and IV).

The next batches of material, NCL388-4 and NCL388-5, were similar to the first batch. Both samples were measured at 100-fold dilution in both PBS and 10 mM NaCl, and both batches showed a PdI of approximately 0.2, a volume distribution size of about 15 nm, and an intensity distribution size of about 20 nm (Figures II-7 and II-8, respectively). Overall, there was a good batch-to-batch agreement with respect to size distribution between these samples. In addition, the measured sizes were in good agreement with the sponsor's reported sizes of 22 nm and 23 nm, respectively.

Batches NCL388-6 and NCL388-7 were larger than earlier batches. NCL388-6 (Figure II-9) was about double the diameter of earlier batches, with an intensity-peak diameter of approximately 45 nm and a volume-peak diameter of approximately 35 nm. NCL388-7 (Figure II-10) was significantly larger, with an intensity-peak diameter of approximately 80 nm and a volume-peak diameter of approximately 60 nm. Due to this significantly larger size, batch NCL388-7 was not used for additional studies.

The set of lots supplied, NCL388-8 through NCL388-11, were again similar to the first batch and in agreement with the expected size. All four batches had intensity-peak and volume-peak diameters of approximately 15-20 nm (Figure II-11 to II-14). NCL388-8, however, was not used in subsequent studies due to bacterial contamination in the sample (Figure I-1).

For the in vivo toxicology and in vitro cytokine studies, several batches were pooled together, namely NCL388-4, NCL388-5, NCL388-6, NCL388-9, NCL388-10 and NCL388-11. This combined batch is referred to as NCL388-12. The properties (DLS and iodine concentration) were assessed again after pooling. The DLS trace of this sample is shown in Figure II-15. The intensity-peak diameter was approximately 50 nm and the volume-peak diameter was approximately 35 nm. The peak shapes are broader than in individual batches, but is as expected due to the size distribution of the various individual batches.

It should be noted that all samples, when made, were ~20 nm (Int-Pk) when initially measured at Nanoprobes. Nanoprobes later discovered that there was no change in size with storage for 1.5 months at -20°C, but that the size increased with longer storage times. However, samples stored at -80°C appeared to retain their original size indefinitely (tested by Nanoprobes monthly to 6 months with no change). The early batches, samples NCL388-1 to NCL388-7, were stored at -20°C at the NCL, and, depending on when the measurements were made, could explain the size discrepancies. The later batches, samples NCL388-8, NCL388-9, NCL388-10, and NCL388-11, were stored at -80°C at NCL and showed good consistency. NCL388-12 was a combination of batches that were stored at both -20°C and -80°C, likely explaining the larger observed size.

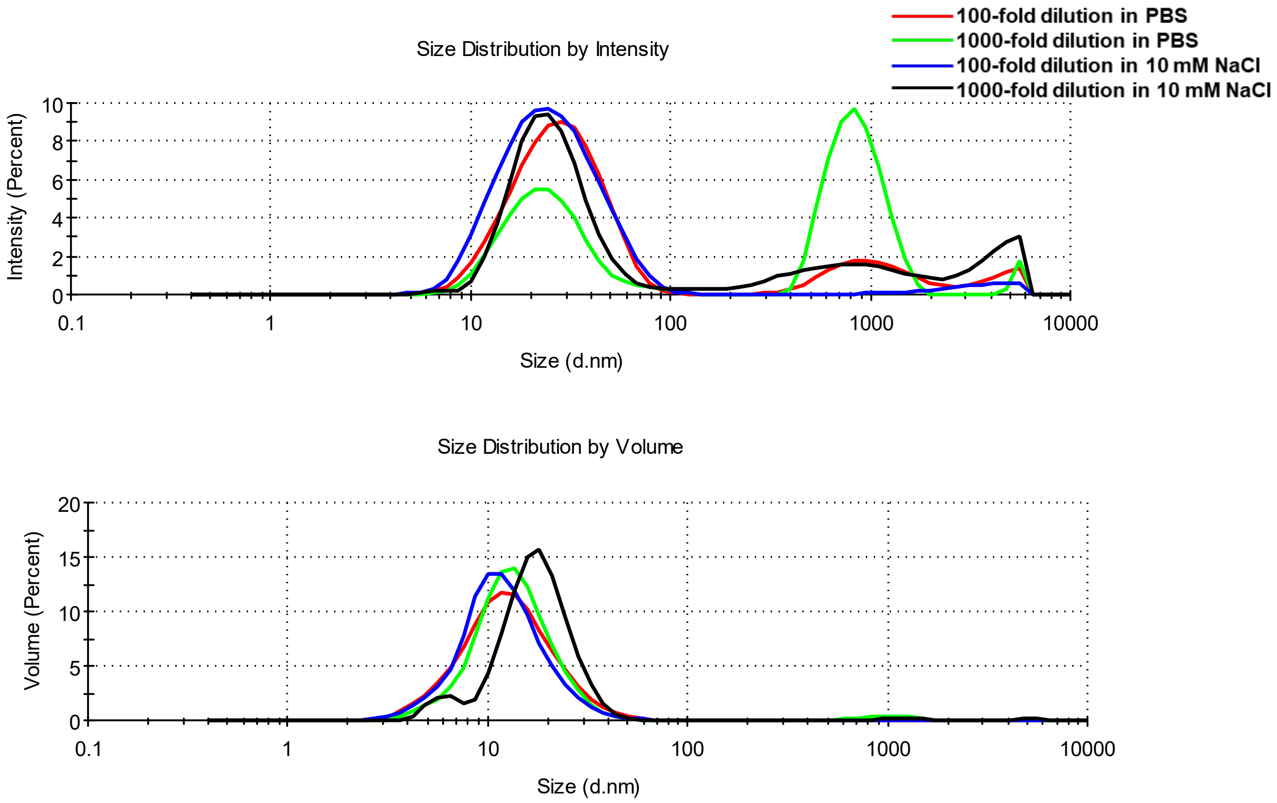

Figure II-1. The averaged intensity and volume distribution plots for NCL388-1 in 10 mM NaCl and PBS.

Table II-1. Summary of the hydrodynamic diameters for NCL388-1 in 10 mM NaCl and PBS.

Dispersing medium	Dilution	Z-Avg nm	PdI	Int-Peak nm	% Int	Vol-Peak nm	% Vol
10 mM NaCl*	100-fold	23 ± 1	0.260 ± 0.021	29 ± 3	96 ± 2	12 ± 4	84 ± 31
10 mM NaCl	1000-fold	65 ± 48	0.375 ± 0.114	25 ± 5	63 ± 12	18 ± 5	96 ± 7
PBS	100-fold	31 ± 3	0.450 ± 0.024	30 ± 4	80 ± 3	13 ± 4	92 ± 14
PBS	1000-fold	215 ± 44	0.553 ± 0.096	24 ± 1	42 ± 5	14 ± 3	93 ± 17

**Results are based on an average of 9 measurements.*

Note: Results are the average of 12 measurements unless stated otherwise. Size is shown as diameter in nanometers (d.nm). Z-Avg is the intensity-weighted average. PdI is the polydispersity index. Int-Peak is the intensity-weighted average over the primary peak. % Int is the percentage of the intensity spectra occupied by the primary peak. Vol-Peak is the volume-weighted average over the primary peak. % Vol is the percentage of the volume spectra occupied by the primary peak.

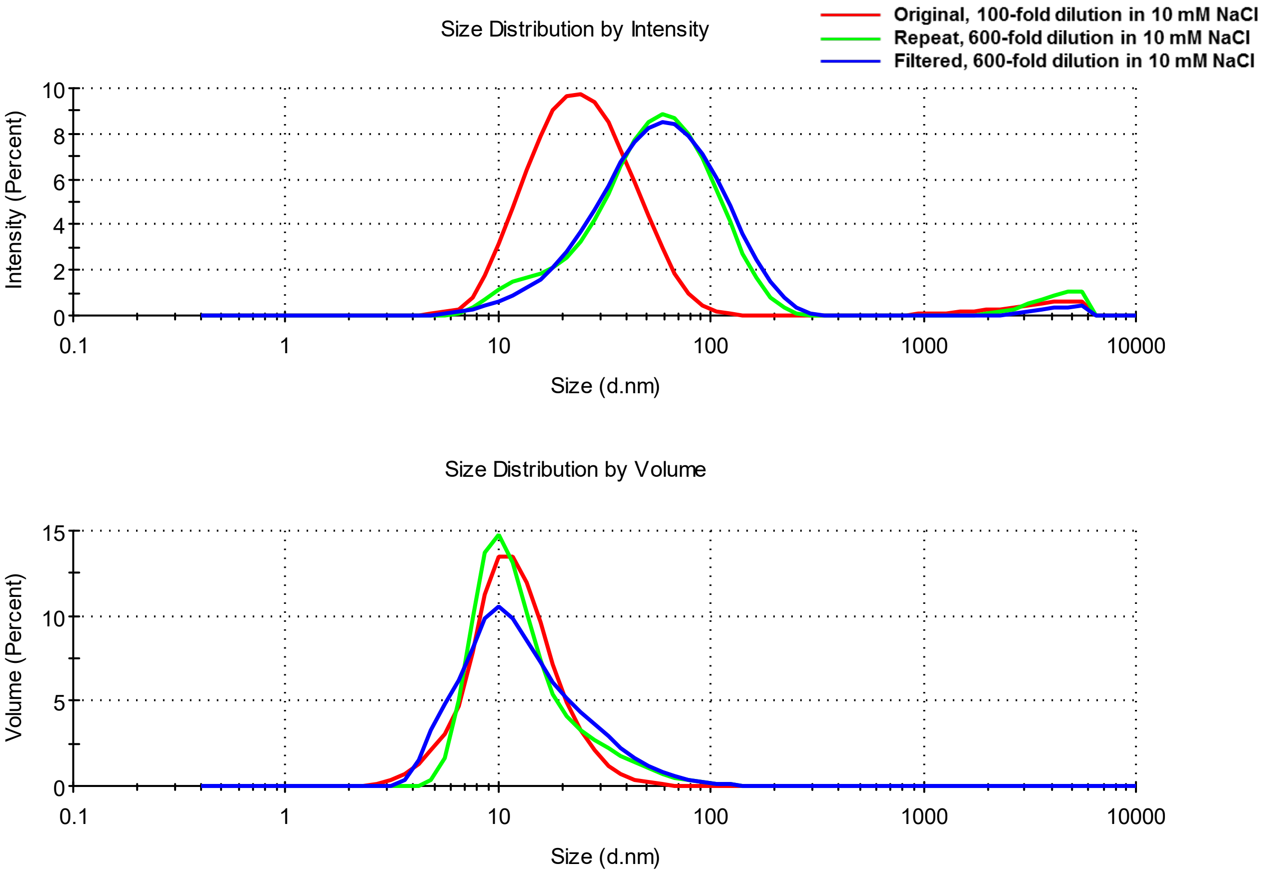

Figure II-2. The averaged intensity and volume distribution plots for NCL388-1 in 10 mM NaCl pre- and post-filtration using the Mustang E-filters.

Table II-2. Summary of the hydrodynamic diameters for NCL388-1 in 10 mM NaCl pre- and post-filtration using the Mustang E-filters.

Sample	Dilution	Z-Avg nm	PdI	Int-Peak nm	% Int	Vol-Peak nm	% Vol
NCL388-1 original	100-fold	23 ± 1	0.260 ± 0.021	29 ± 3	96 ± 2	12 ± 4	84 ± 31
NCL388-1 repeat	600-fold	46 ± 2	0.387 ± 0.031	63 ± 5	94 ± 5	15 ± 2	99 ± 5
NCL388-1 filtered	600-fold	47 ± 2	0.321 ± 0.021	68 ± 4	98 ± 2	16 ± 4	96 ± 13

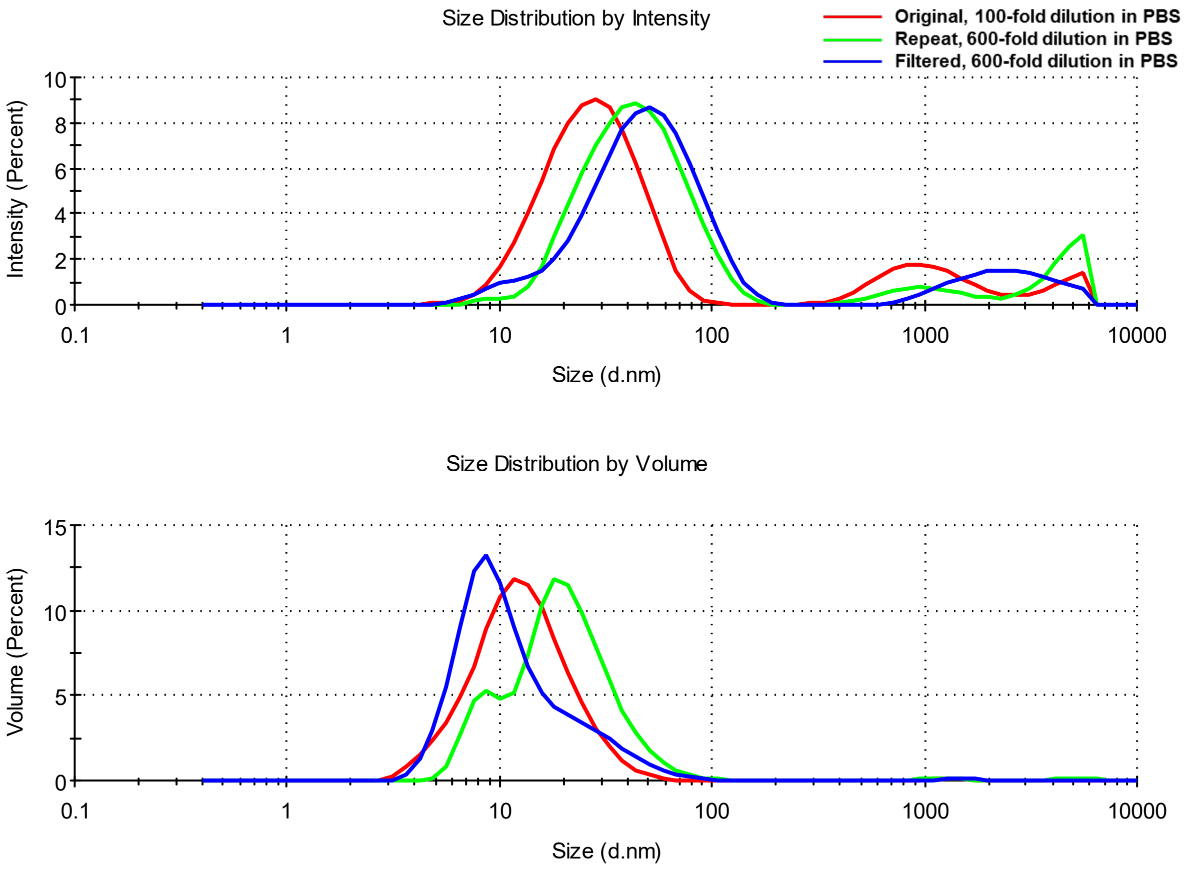

Figure II-3. The averaged intensity and volume distribution plots for NCL388-1 in PBS pre- and post-filtration using the Mustang E-filters.

Table II-3. Summary of the hydrodynamic diameters for NCL388-1 in PBS pre- and post-filtration using the Mustang E-filters.

Sample	Dilution	Z-Avg nm	PdI	Int-Peak nm	% Int	Vol-Peak nm	% Vol
NCL388-1 original	100-fold	31 ± 3	0.450 ± 0.024	30 ± 4	80 ± 3	13 ± 3	92 ± 14
NCL388-1 repeat	600-fold	62 ± 15	0.275 ± 0.121	48 ± 6	84 ± 5	18 ± 7	83 ± 27
NCL388-1 filtered	600-fold	45 ± 3	0.464 ± 0.016	53 ± 5	84 ± 5	13 ± 4	95 ± 9

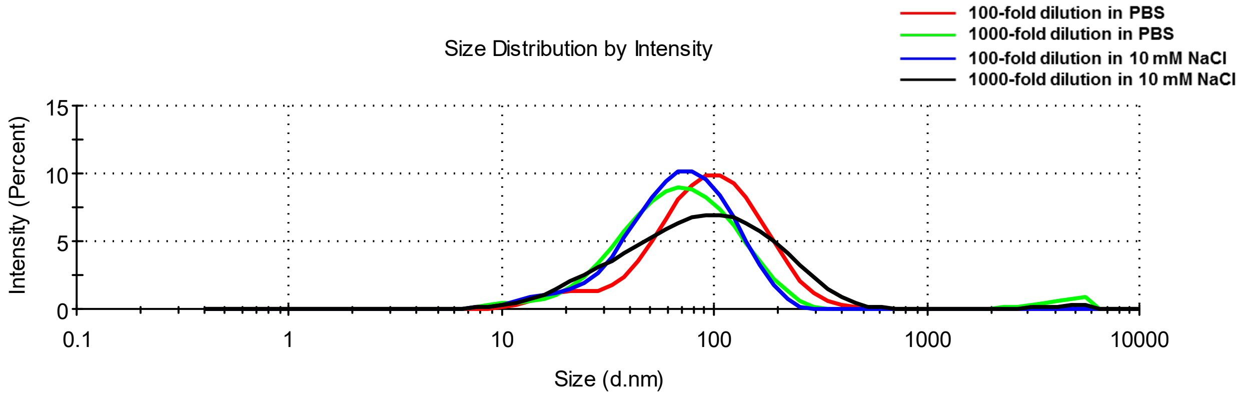

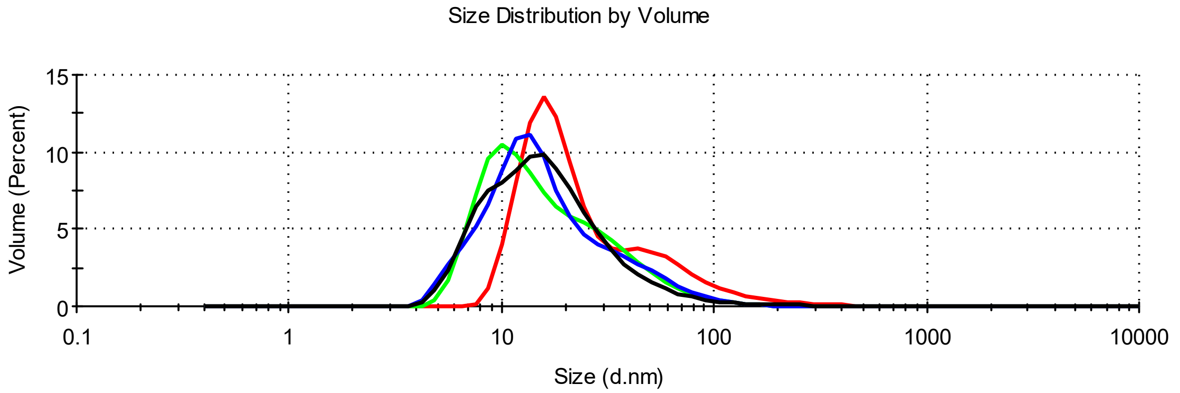

Figure II-4. The averaged intensity and volume distribution plots for NCL388-2 in 10 mM NaCl and PBS.

Table II-4. Summary of the hydrodynamic diameters for NCL388-2 in 10 mM NaCl and PBS.

Dispersing medium	Dilution	Z-Avg nm	PdI	Int-Peak nm	% Int	Vol-Peak nm	% Vol
PBS	100-fold	76 ± 11	0.277 ± 0.017	110 ± 22	96 ± 4	31 ± 22	97 ± 6
PBS	1000-fold	59 ± 4	0.333 ± 0.032	79 ± 6	95 ± 3	16 ± 8	83 ± 19
10 mM NaCl	100-fold	56 ± 4	0.255 ± 0.005	78 ± 6	97 ± 3	17 ± 8	87 ± 15
10 mM NaCl	1000-fold	65 ± 4	0.409 ± 0.011	111 ± 12	98 ± 3	19 ± 6	93 ± 17

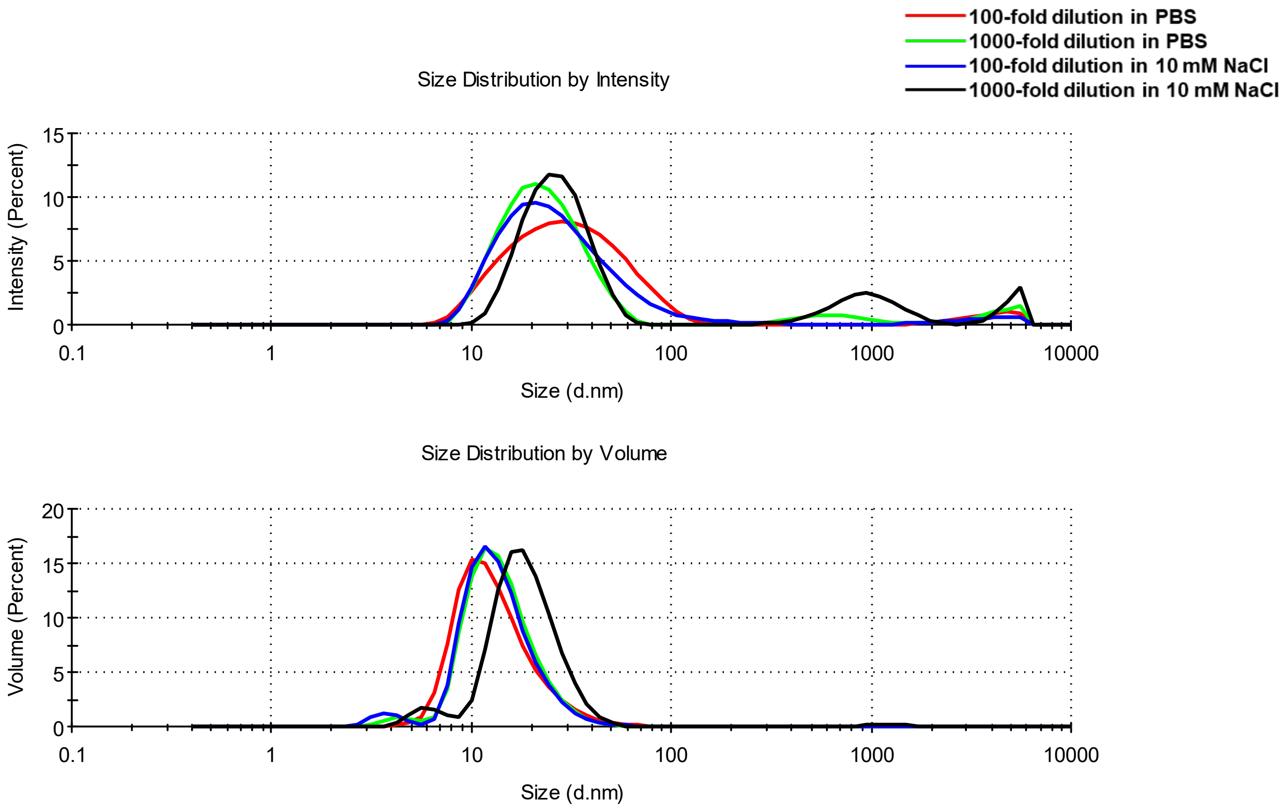

Figure II-5. The averaged intensity and volume distribution plots for NCL388-3 in 10 mM NaCl and PBS.

Table II-5. Summary of the hydrodynamic diameters for NCL388-3 in 10 mM NaCl and PBS.

Dispersing medium	Dilution	Z-Avg nm	PdI	Int-Peak nm	% Int	Vol-Peak nm	% Vol
PBS	100-fold	27 ± 1	0.331 ± 0.011	35 ± 2	95 ± 1	14 ± 1	100 ± 0
PBS	1000-fold	24 ± 2	0.342 ± 0.040	24 ± 1	89 ± 3	15 ± 1	97 ± 9
10 mM NaCl	100-fold	24 ± 0	0.268 ± 0.017	31 ± 3	96 ± 2	15 ± 1	96 ± 14
10 mM NaCl	1000-fold	81 ± 37	0.250 ± 0.074	27 ± 2	77 ± 7	20 ± 1	93 ± 15

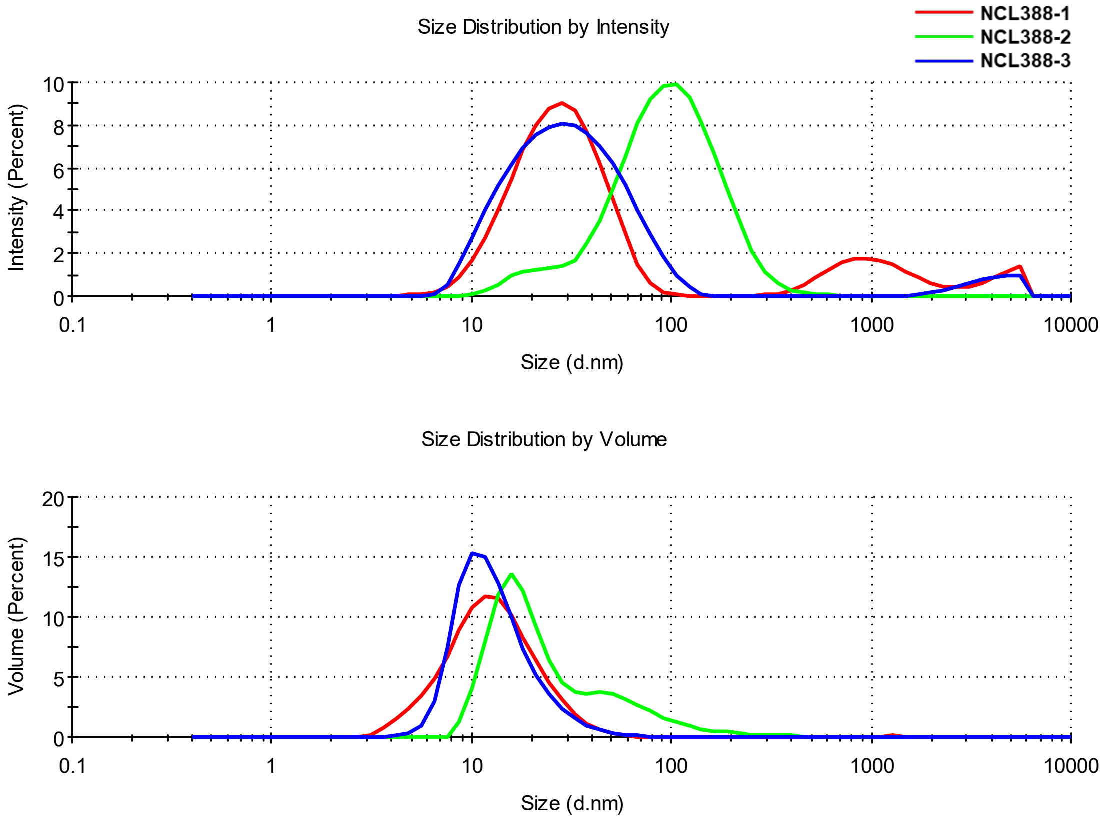

Figure II-6. The averaged intensity and volume distribution plots for NCL388-1, NCL388-2 and NCL388-3.

Table II-6. Summary of the hydrodynamic diameters for NCL388-1, NCL388-2 and NCL388-3.

Sample	Dilution	Z-Avg nm	PdI	Int-Peak nm	% Int	Vol-Peak nm	% Vol
NCL388-1	100-fold in PBS	31 ± 3	0.450 ± 0.024	30 ± 4	80 ± 3	13 ± 4	92 ± 14
NCL388-2	100-fold in PBS	76 ± 11	0.277 ± 0.017	110 ± 22	96 ± 4	31 ± 22	97 ± 6
NCL388-3	100-fold in PBS	27 ± 1	0.331 ± 0.011	35 ± 2	95 ± 1	14 ± 1	100 ± 0

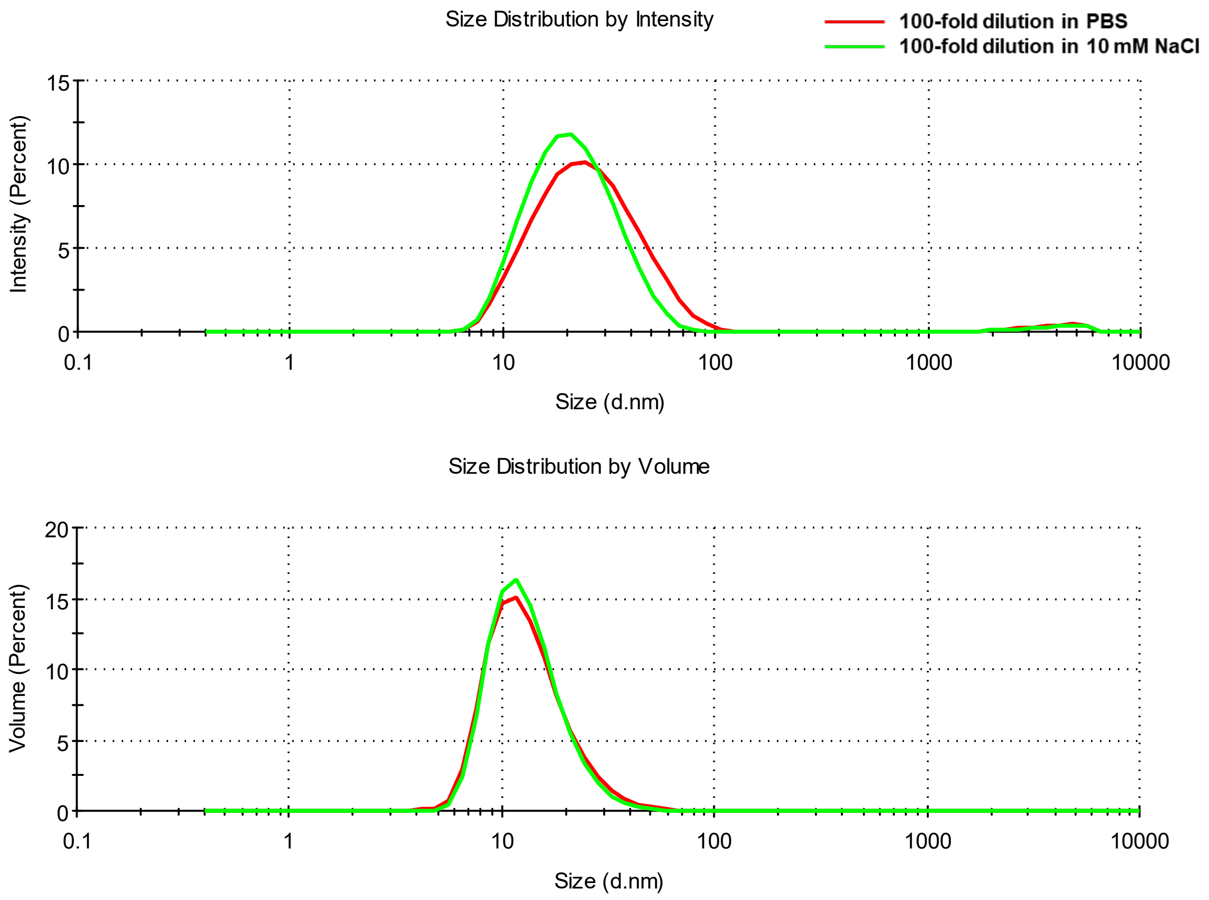

Figure II-7. The averaged intensity and volume distribution plots for NCL388-4.

Table II-7. Summary of the hydrodynamic diameters for NCL388-4.

Sample	Dilution	Z-Avg nm	PdI	Int-Peak nm	% Int	Vol-Peak nm	% Vol
NCL388-4	100-fold in PBS	22 ± 1	0.231 ± 0.015	28 ± 2	98 ± 1	14 ± 1	100 ± 0
NCL388-4	100-fold in 10 mM NaCl	20 ± 0	0.198 ± 0.012	23 ± 1	9 ± 1	14 ± 1	100 ± 0

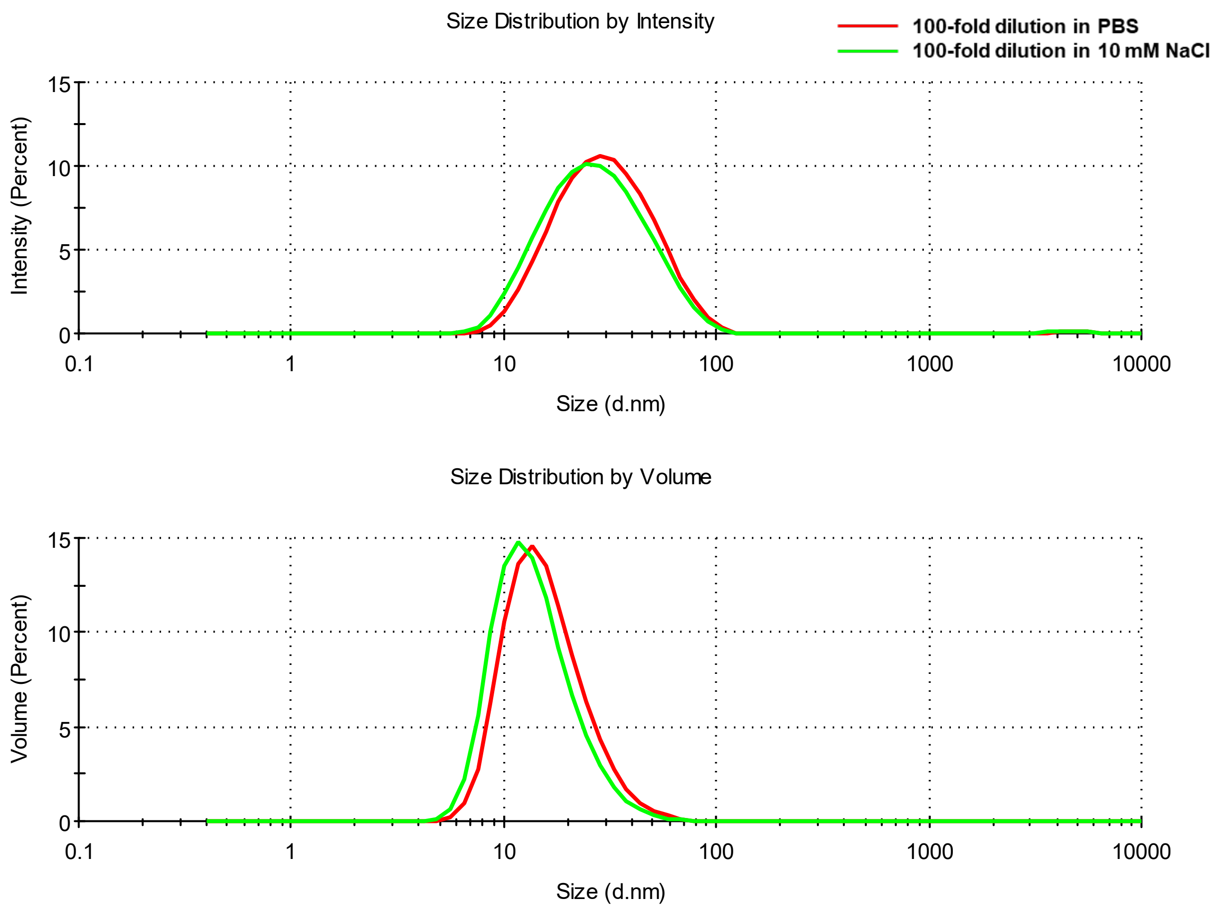

Figure II-8. The averaged intensity and volume distribution plots for NCL388-5.

Table II-8. Summary of the hydrodynamic diameters for NCL388-5.

Sample	Dilution	Z-Avg nm	PdI	Int-Peak nm	% Int	Vol-Peak nm	% Vol
NCL388-5	100-fold in PBS	26 ± 1	0.202 ± 0.010	33 ± 1	100 ± 1	17 ± 1	100 ± 0
NCL388-5	100-fold in 10 mM NaCl	24 ± 0	0.217 ± 0.011	31 ± 1	99 ± 1	15 ± 1	100 ± 0

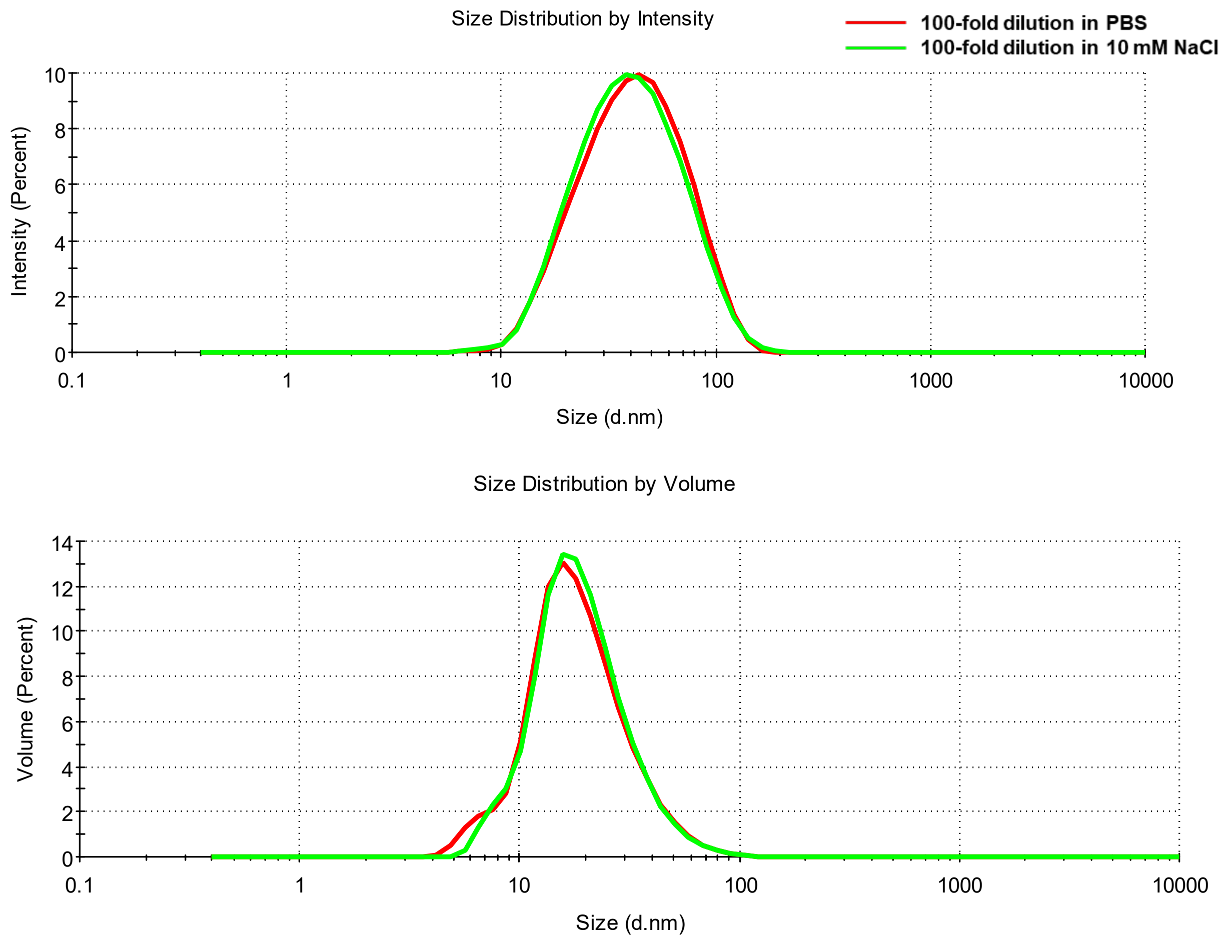

Figure II-9. The averaged intensity and volume distribution plots for NCL388-6.

Table II-9. Summary of the hydrodynamic diameters for NCL388-6.

Sample	Dilution	Z-Avg nm	PdI	Int-Peak nm	% Int	Vol-Peak nm	% Vol
NCL388-6	100-fold in PBS	36 ± 1	0.216 ± 0.007	47 ± 2	100 ± 0	19 ± 6	89 ± 27
NCL388-6	100-fold in 10 mM NaCl	34.3 ± 0.9	0.215 ± 0.007	45 ± 2	100 ± 0	20 ± 4	97 ± 11

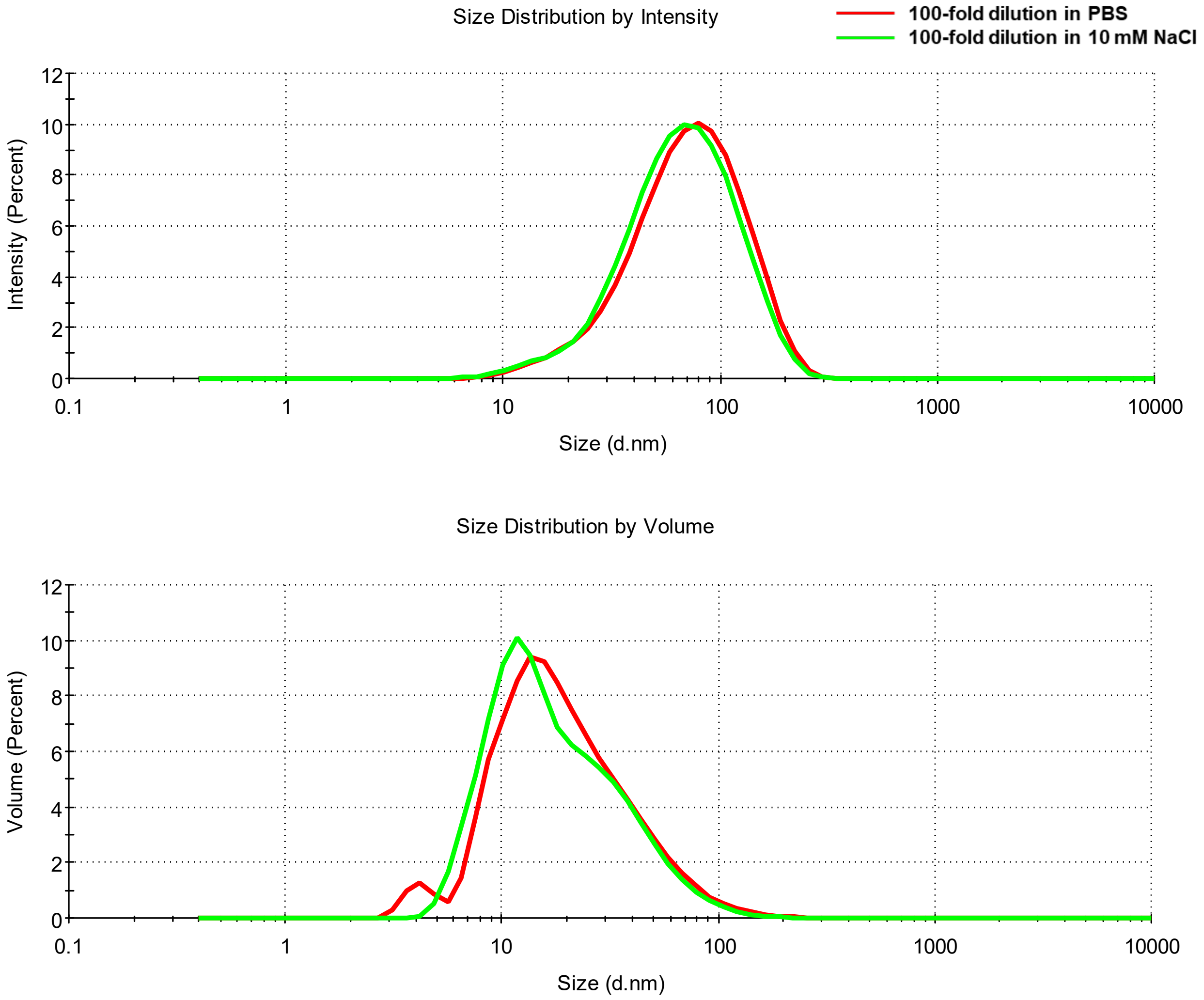

Figure II-10. The averaged intensity and volume distribution plots for NCL388-7.

Table II-10. Summary of the hydrodynamic diameters for NCL388-7.

Sample	Dilution	Z-Avg nm	PdI	Int-Peak nm	% Int	Vol-Peak nm	% Vol
NCL388-7	100-fold in PBS	59 ± 1	0.257 ± 0.008	82 ± 2	99 ± 3	22 ± 10	91 ± 16
NCL388-7	100-fold in 10 mM NaCl	55.1 ± 0.7	0.253 ± 0.006	76 ± 2	98 ± 3	20 ± 9	92 ± 12

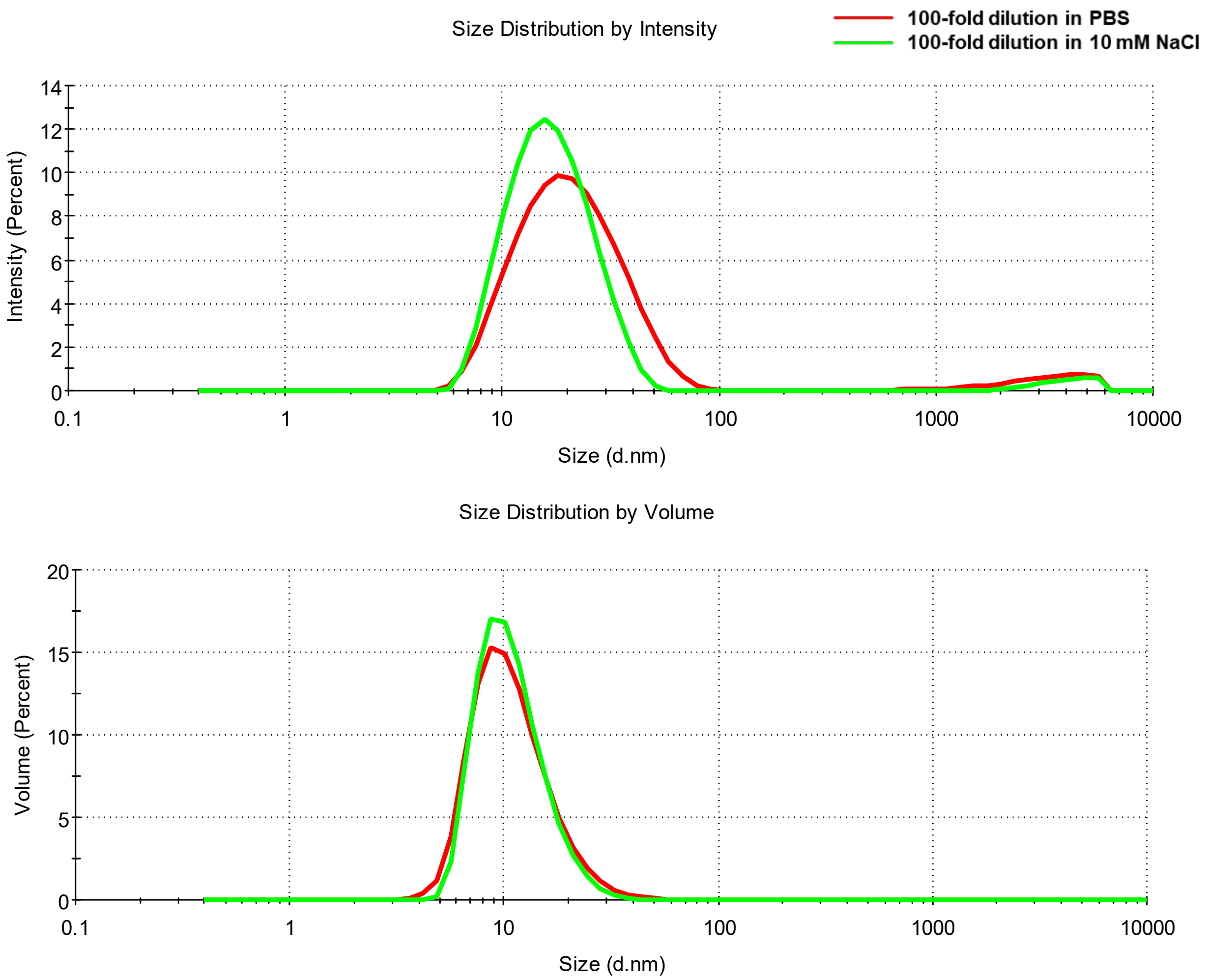

Figure II-11. The averaged intensity and volume distribution plots for NCL388-8.

Table II-11. Summary of the hydrodynamic diameters for NCL388-8.

Sample	Dilution	Z-Avg nm	PdI	Int-Peak nm	% Int	Vol-Peak nm	% Vol
NCL388-8	100-fold, PBS	19.1 ± 0.2	0.267 ± 0.023	22.6 ± 0.9	94.3 ± 1.5	11.5 ± 0.7	100 ± 0
NCL388-8	100-fold, 10 mM NaCl	15.7 ± 0.3	0.200 ± 0.016	17.9 ± 0.4	97.1 ± 1.2	11.3 ± 0.3	100 ± 0

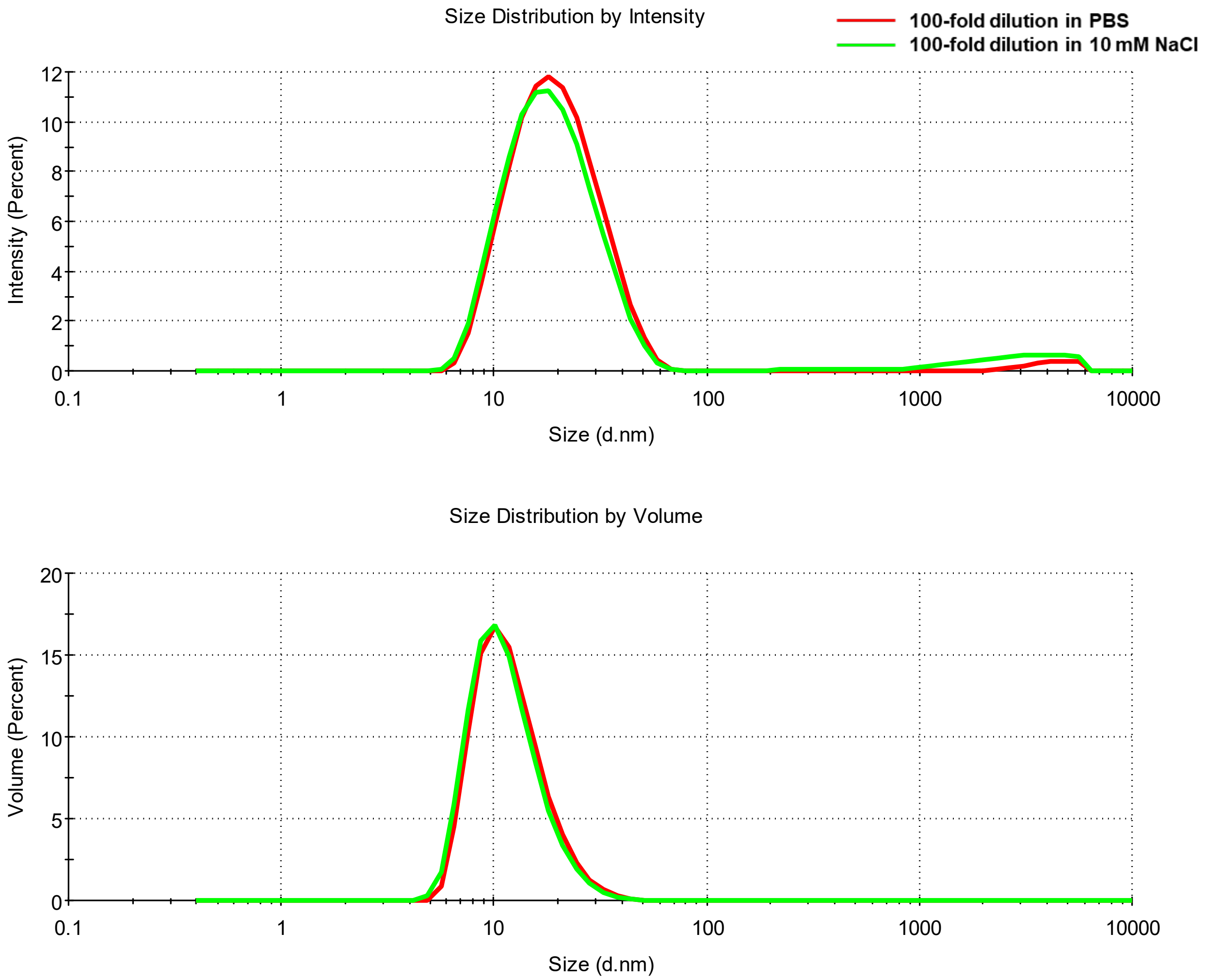

Figure II-12. The averaged intensity and volume distribution plots for NCL388-9.

Table II-12. Summary of the hydrodynamic diameters for NCL388-9.

Sample	Dilution	Z-Avg nm	PdI	Int-Peak nm	% Int	Vol-Peak nm	% Vol
NCL388-9	100-fold, PBS	17.7 ± 0.3	0.195 ± 0.013	20.9 ± 0.6	98.1 ± 0.9	12.4 ± 0.3	100 ± 0
NCL388-9	100-fold, 10 mM NaCl	17.9 ± 0.3	0.244 ± 0.017	19.9 ± 0.7	92.6 ± 2.0	11.9 ± 0.4	100 ± 0

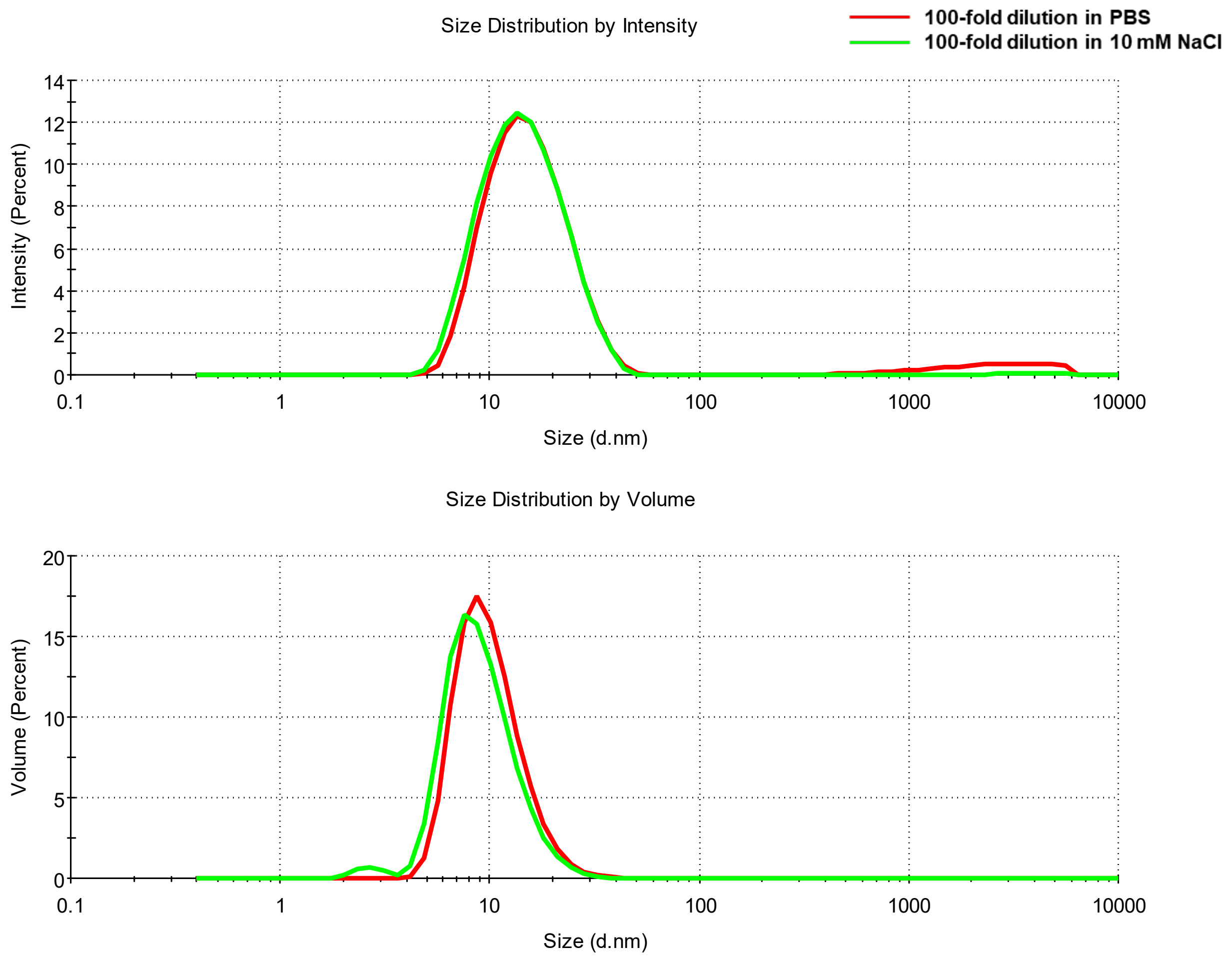

Figure II-13. The averaged intensity and volume distribution plots for NCL388-10.

Table II-13. Summary of the hydrodynamic diameters for NCL388-10.

Sample	Dilution	Z-Avg nm	PdI	Int-Peak nm	% Int	Vol-Peak nm	% Vol
NCL388-10	100-fold, PBS	14.7 ± 0.3	0.230 ± 0.011	16.1 ± 0.6	94 ± 2	10.3 ± 0.4	100 ± 0
NCL388-10	100-fold, 10 mM NaCl	13.1 ± 0.1	0.165 ± 0.010	15.6 ± 0.4	99.5 ± 0.9	9.5 ± 0.3	98 ± 5

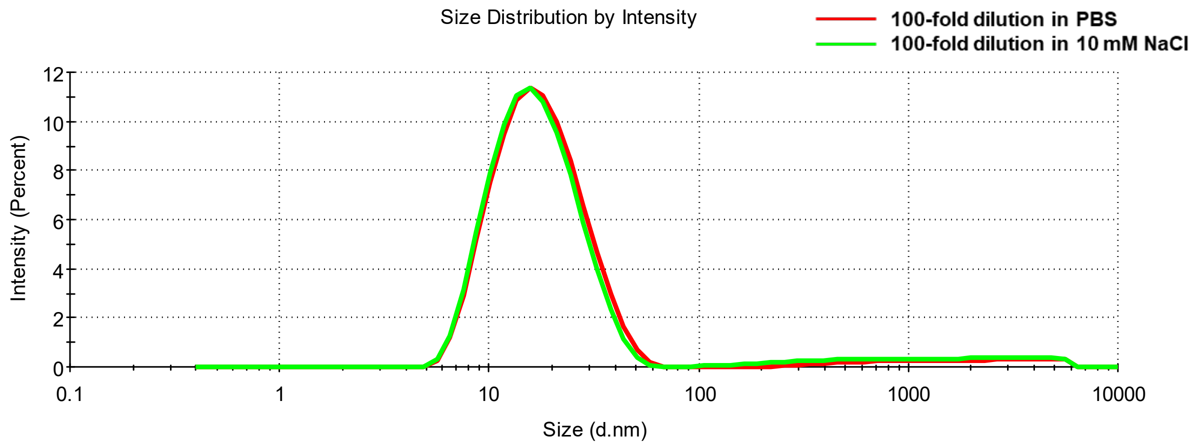

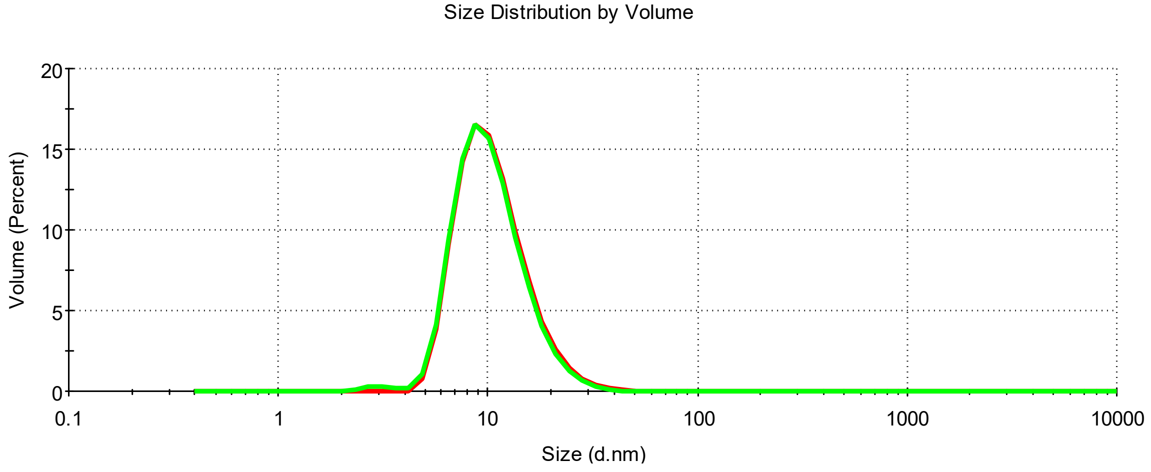

Figure II-14. The averaged intensity and volume distribution plots for NCL388-11.

Table II-14. Summary of the hydrodynamic diameters for NCL388-11.

Sample	Dilution	Z-Avg nm	PdI	Int-Peak nm	% Int	Vol-Peak nm	% Vol
NCL388-11	100-fold, PBS	16.3 ± 0.3	0.229 ± 0.012	18.7 ± 0.7	94.8 ± 2.1	11.0 ± 0.4	100 ± 0
NCL388-11	100-fold, 10 mM NaCl	16.2 ± 0.3	0.253 ± 0.022	17.8 ± 0.5	92.3 ± 2.0	10.8 ± 0.4	98.9 ± 3.7

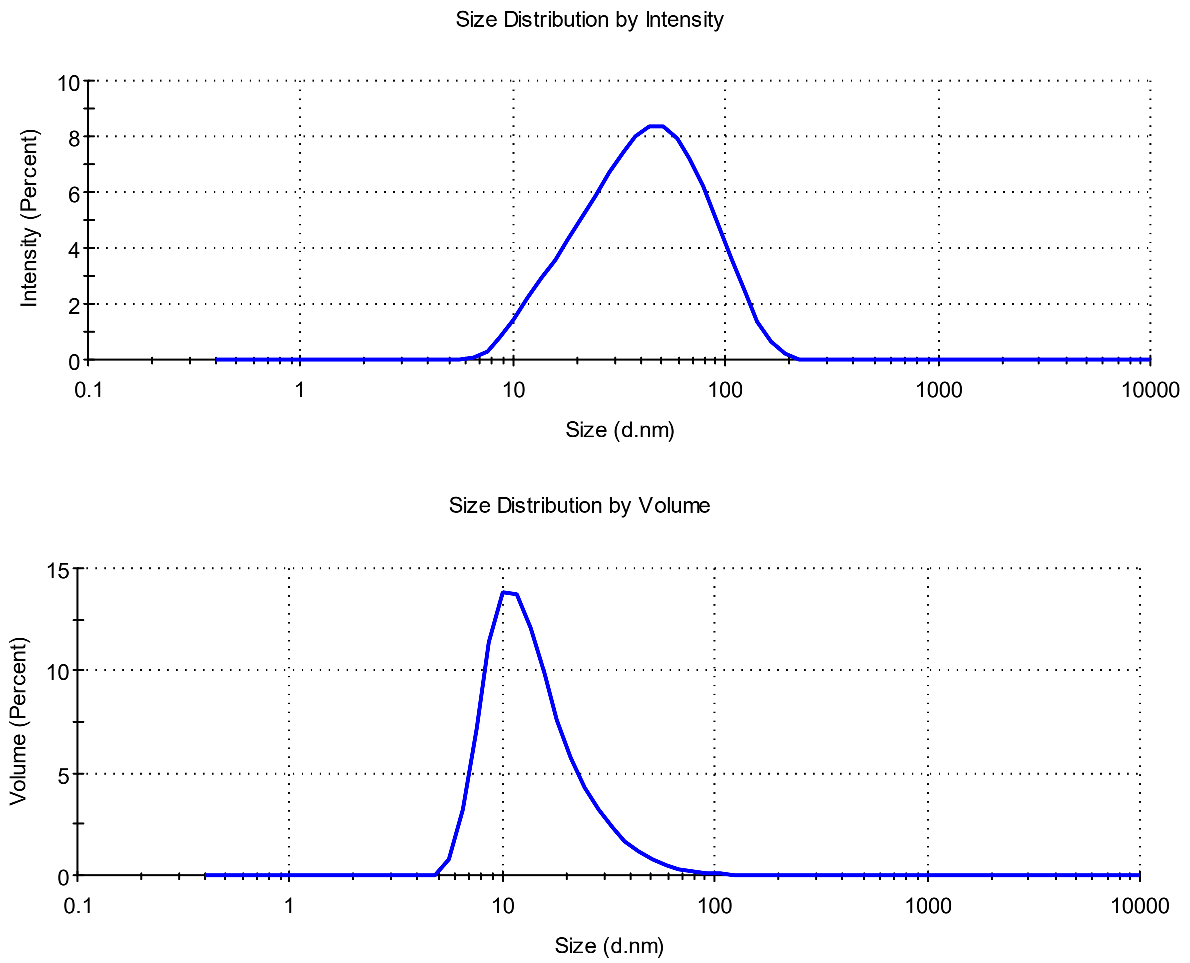

Figure II-15. The averaged intensity and volume distribution plots for NCL388-12.

Table II-15. Summary of the hydrodynamic diameters for NCL388-12.

Sample	Dilution	Z-Avg nm	PdI	Int-Peak nm	% Int	Vol-Peak nm	% Vol
NCL388-12	100-fold	34 ± 2	0.268 ± 0.005	49.1 ± 3	100 ± 0	16 ± 1	100 ± 0

Zeta Potential (PCC-2)

Design and Methods

A Malvern Zetasizer Nano ZS instrument was used to measure zeta potential at 25°C for all samples. NCL protocol PCC-2 was followed (https://ncl.cancer.gov/resources/assay-cascade-protocols). Samples were diluted 100-fold in 10 mM NaCl. Sample pH was measured and/or adjusted before loading into a pre-rinsed folded capillary cell. Measurements were made at both native pH and after adjustment to neutral pH using 1 N standardized HCl. An applied voltage of 150 V was used. Traces in the figures represent the average of three measurements.

The instrument was validated by running an appropriate standard (Zeta Potential Transfer Standard, DTS0050, zeta potential value of -42 ± 4 mV at 25˚C, Malvern Instruments) before all zeta potential measurements.

Results and Discussion

The zeta potential distributions for NCL388 are shown in Figures II-16 to II-21 and summarized in Tables II-16 to II-21. All eleven batches of the formulation were analyzed, to provide the sponsor with a measure of batch-to-batch variability. All batches showed neutral zeta potential values at both native and neutral pH ranges. Zeta potentials ranging +10 to -10 mV are generally considered neutral. No theoretical zeta potential value was provided by the sponsor; however, this was in agreement with the expected range given the PEGylated nature of the formulation.

Filtration of the sample through the Mustang E-filters did not affect the zeta potential value (Figure II-16).

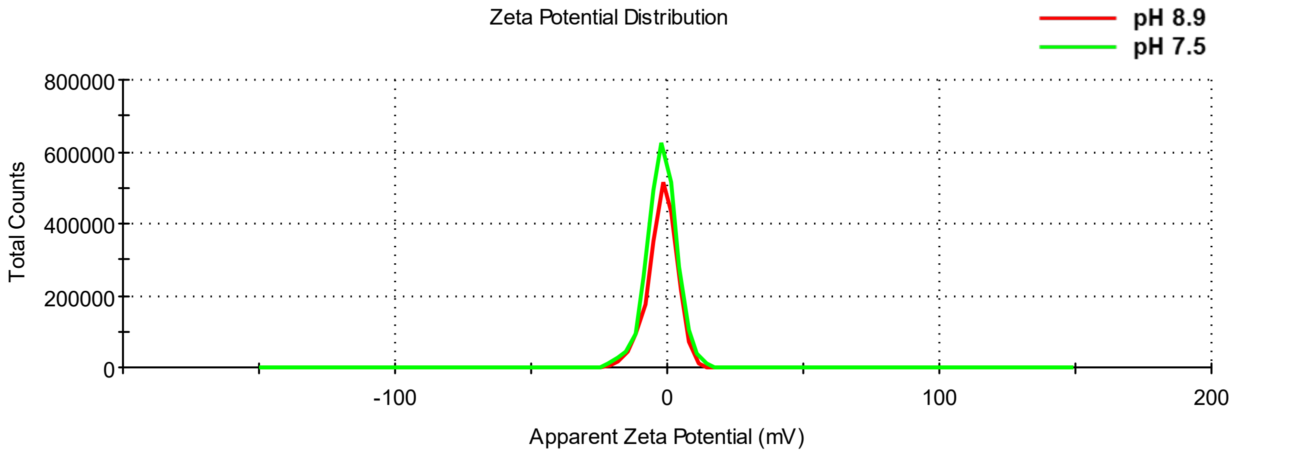

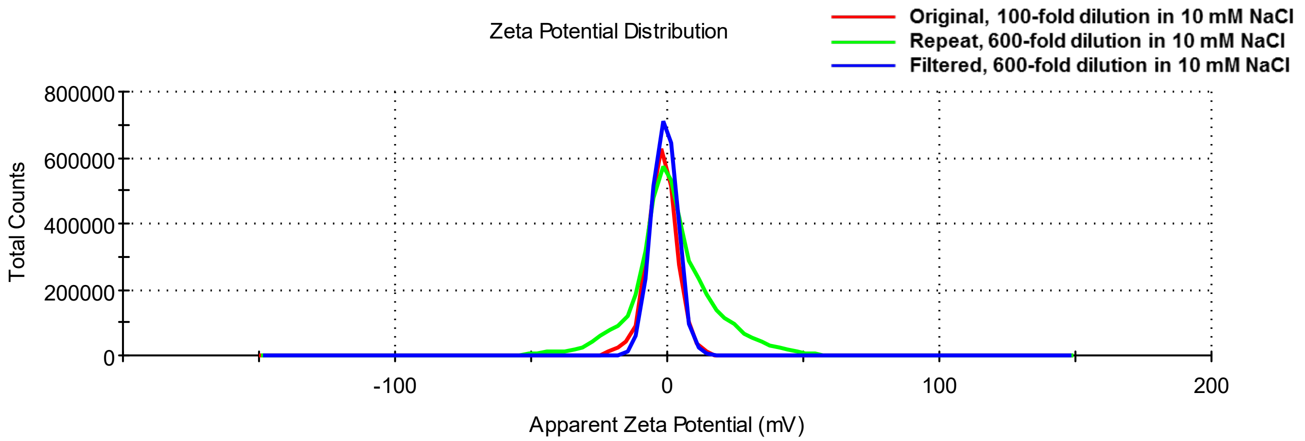

Figure II-16. The averaged zeta potential distribution for NCL388-1.

Table II-16. Summary of the zeta potential for NCL388-1.

Sample	pH	Zeta Potential mV
NCL388-1	8.9	-2.0 ± 0.4
NCL388-1	7.5	-2.1 ± 0.3
NCL388-1, repeat	7.5	1 ± 2
NCL388-1, filtered	7.3	-1 ± 1

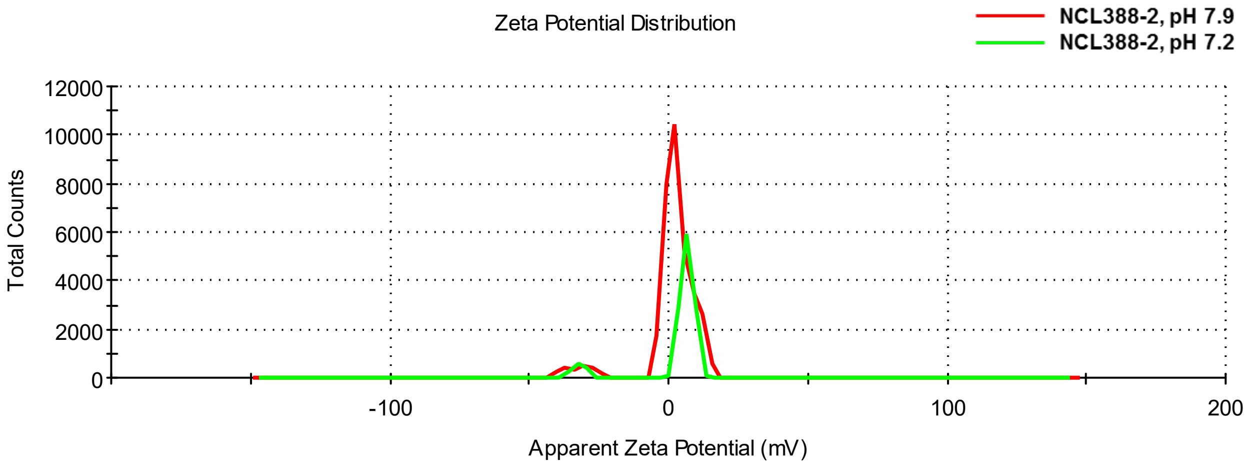

Figure II-17. The averaged zeta potential distribution for NCL388-2.

Table II-17. Summary of the zeta potential for NCL388-2.

Sample	pH	Zeta Potential mV
NCL388-2	7.9	0.01 ± 0.002
NCL388-2	7.2	0.02 ± 0.04

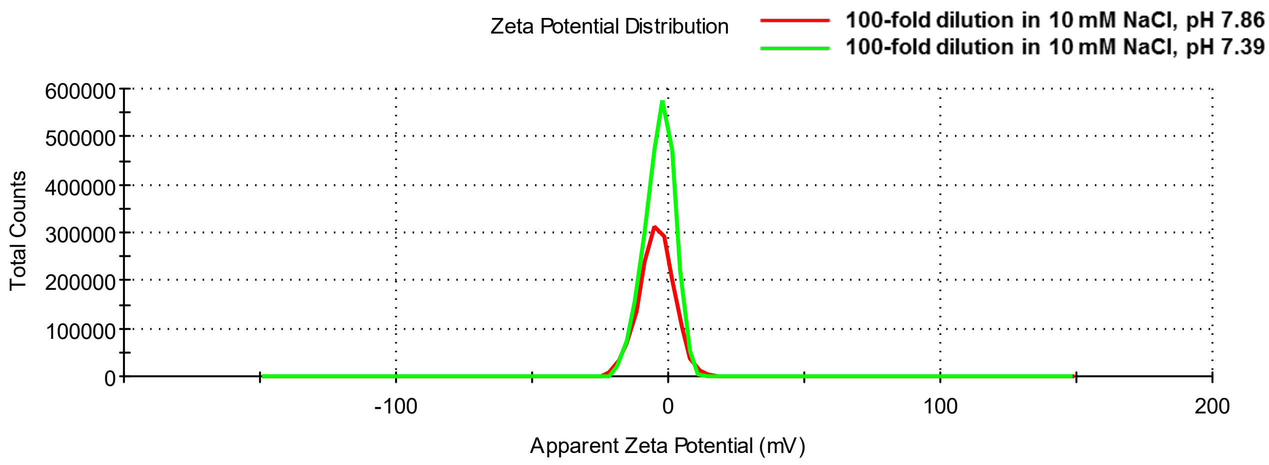

Figure II-18. The averaged zeta potential distribution for NCL388-3.

Table II-18. Summary of the zeta potential for NCL388-3.

Sample	pH	Zeta Potential mV
NCL388-3	7.9	0.4 ± 0.1
NCL388-3	7.4	-3.4 ± 0.2

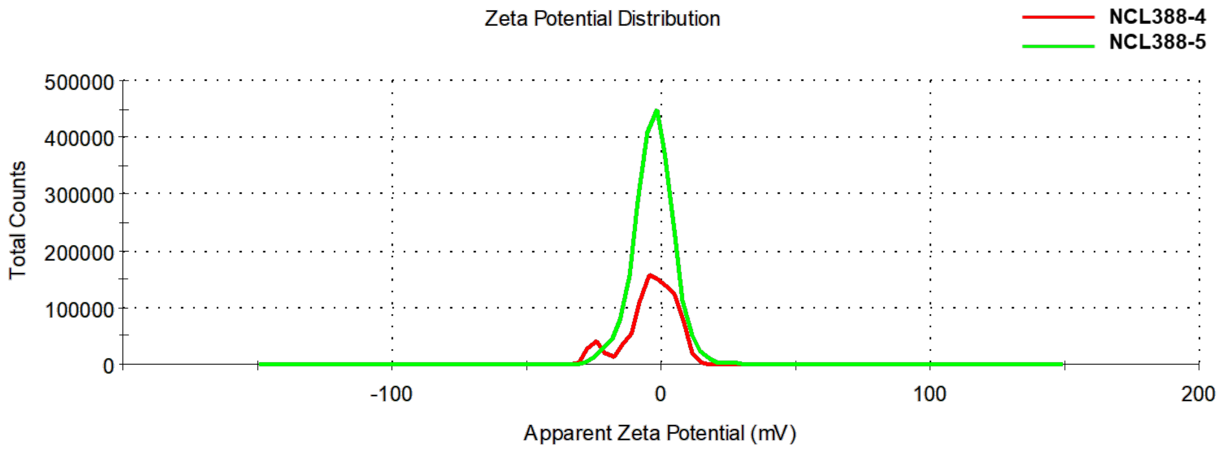

Figure II-19. The averaged zeta potential distribution for NCL388-4 and NCL388-5.

Table II-19. Summary of the zeta potential for NCL388-4 and NCL388-5.

Sample	pH	Zeta Potential mV
NCL388-4	7.63	-4 ± 1
NCL388-5	7.69	-3 ± 1

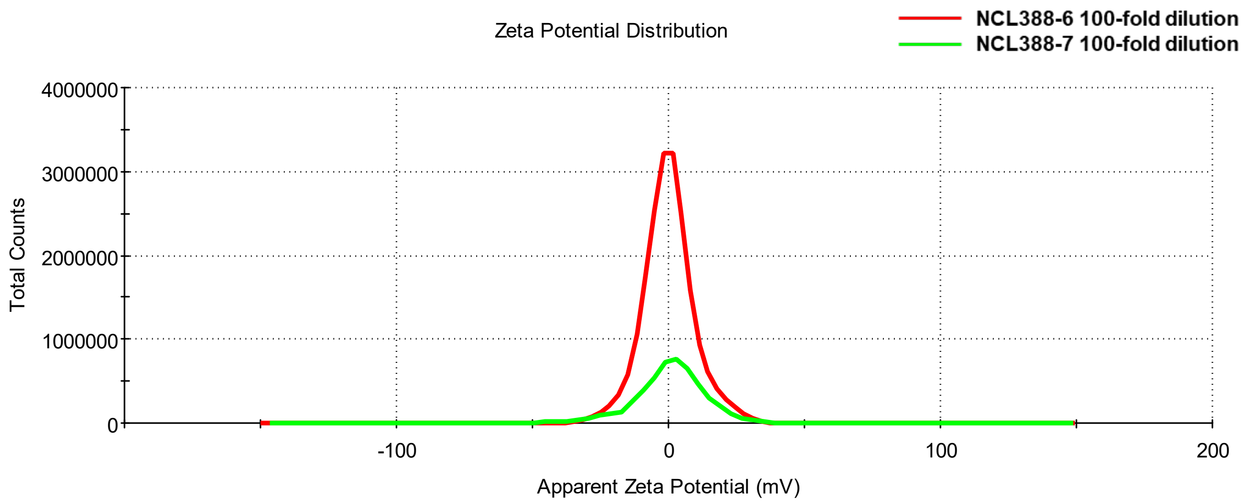

Figure II-20. The averaged zeta potential distribution for NCL388-6 and NCL388-7.

Table II-20. Summary of the zeta potential for NCL388-6 and NCL388-7.

Sample	pH	Zeta Potential mV
NCL388-6	7.87	-0.4 ± 1.6
NCL388-7	7.72	-0.8 ± 1.2

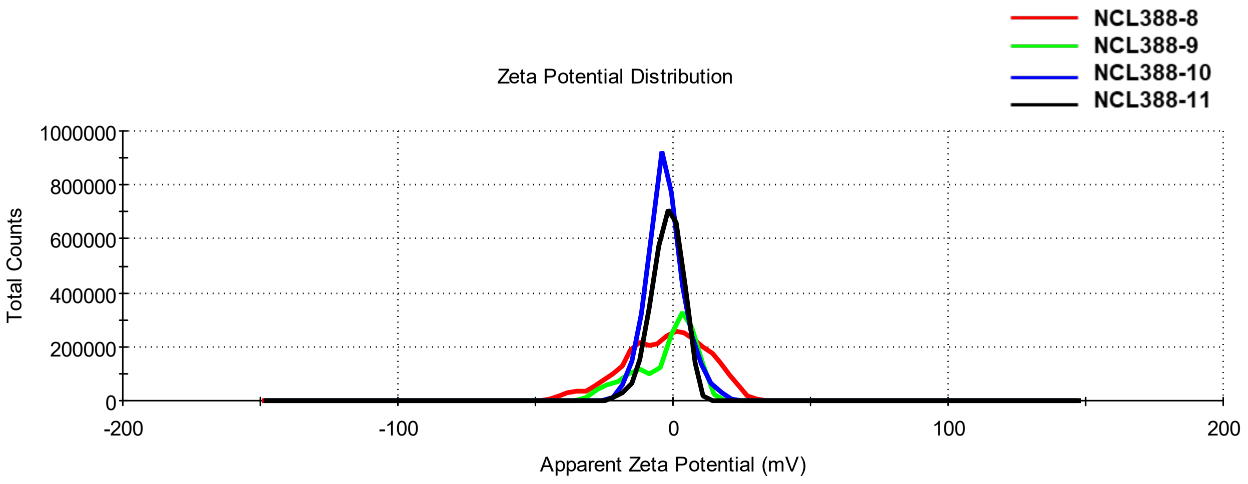

Figure II-21. The averaged zeta potential distribution for NCL388-8, NCL388-9, NCL388-10 and NCL388-11.

Table II-21. Summary of the zeta potential for NCL388-8, NCL388-9, NCL388-10 and NCL388-11.

Sample	pH	Zeta Potential mV
NCL388-8	7.74	-2.4 ± 1.1
NCL388-9	7.71	-2.3 ± 0.7
NCL388-10	7.93	-2.7 ± 0.5
NCL388-11	7.96	-2.4 ± 0.5

Polymer Molecular Weight by SEC-MALS

Design and Methods

The polymer molecular weight of NCL388-3 was determined using size-exclusion chromatography (SEC) coupled with a multiple angle light scattering (MALS) apparatus. When refractive index (RI, concentration detector) is used in conjunction with size exclusion chromatography and multiple angle light scattering (SEC-MALS), the value of dn/dc is necessary for a molecular weight determination. The dn/dc value is the change in the refractive index in response to a change in concentration.

Measurement of dn/dc begins with a manual injection of 1 mL pure solvent (PBS in this case), using a 1 mL disposable syringe, into an RI detector (Optilab T-rEX, 660 nm laser, Wyatt Technology, Santa Barbara, CA). This is used to produce the pure solvent baseline. Next, each sample (starting with the lowest concentration) was manually injected (800 µL) with a new 1 mL disposable syringe. Calibration standards of NCL388-3 were prepared in PBS at 0.1, 0.25, 0.5, 1.0, and 2.5 mg/mL. After all samples had been injected, pure PBS was injected again for a second baseline determination. ASTRA (v6.1.2.84, Wyatt Technology) was used to calculate dn/dc.

The chromatographic system for SEC-MALS consisted of an isocratic pump (Agilent G1310B, Palo Alto, CA), well-plate autosampler (Agilent G1329A), and TSK G6000PW (7.8 mm ID x 30 cm, 7 µm) column (Tosoh Bioscience LLC, King of Prussia, PA). The size exclusion column was connected in-line to a light scattering (MALS) detector (DAWN HELEOS-II, 690 nm laser, Wyatt Technology, Santa Barbara, CA) and a refractive index (RI) detector (Optilab T-rEX, 660 nm laser, Wyatt Technology, Santa Barbara, CA). The isocratic mobile phase was PBS at a flow rate of 1 mL/min. The sample was diluted 100-fold in PBS and 100 µL was injected into the chromatographic system.

MALS normalization constants were determined by running PEO 21.2 kDa (Pressure Chemical Co. PEG 5000 Mw 5160) at 10 mg/mL in PBS. The measured dn/dc value for NCL388-3 was used for the molecular weight calculations.

Results and Discussion

The dn/dc value for NCL388-3 was 0.7401 ± 0.0089 mL/g (Figure II-22B). This value was used for molecular weight determination by multiple angle light scattering (MALS).

The subsequent SEC-MALS chromatogram for NCL388-3 is shown in Figure II-22C. NCL388-3 showed a broad distribution. Using both the light scattering (solid trace) and refractive index (dashed trace) signals and the measured dn/dc value, the molar mass distribution across the peaks were calculated. From Figure II-22C, the light scattering and refractive index signals do not overlay. The reason for this is because the sample consists of several size populations with varying molar masses and in different amounts. Because of this, the molar mass was determined based on each of these signals along with the overall molar mass across both signals. The molar mass based on the light scattering signal was 351 ± 26 kDa. The molar mass based on the refractive index signal was 142 ± 6 kDa. The light scattering signal is biased towards larger particles, hence its measured molar mass, as expected, was larger than the refractive index derived molar mass. However, refractive index is a concentration detector, thus the majority of the sample had a molar mass of 142 kDa. The overall molecular weight across the entire peak (which takes into account contributions from both signals) was 205 ± 20 kDa.

A. NCL388-3 Calibration for dn/dc

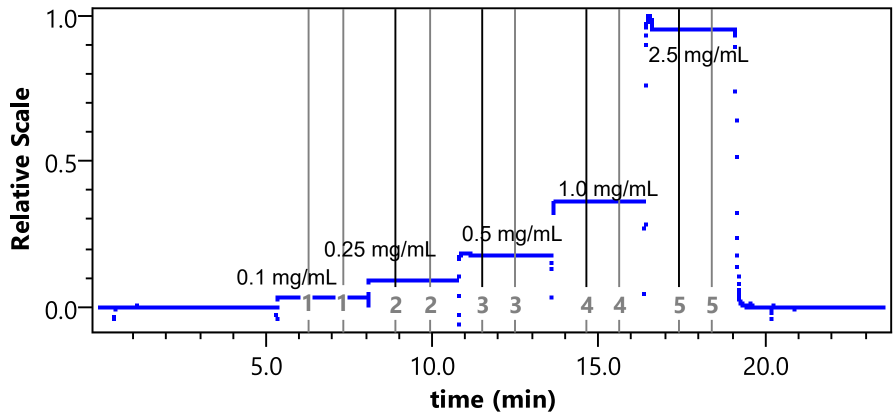

B. dn/dc Plot

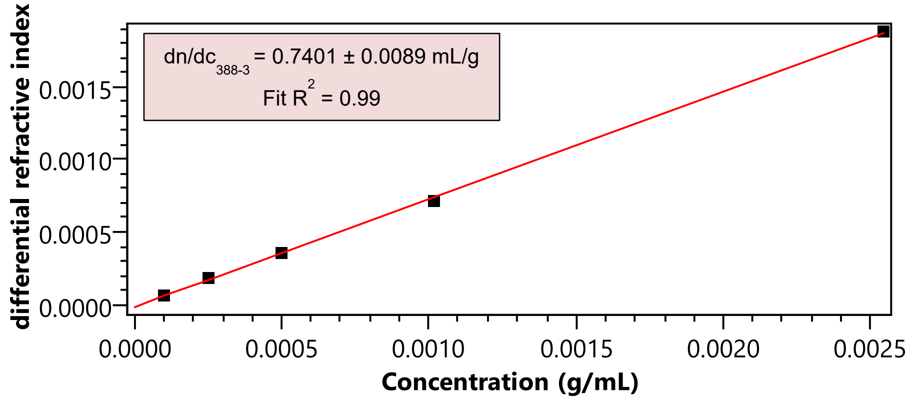

C. Molecular Weight Distribution

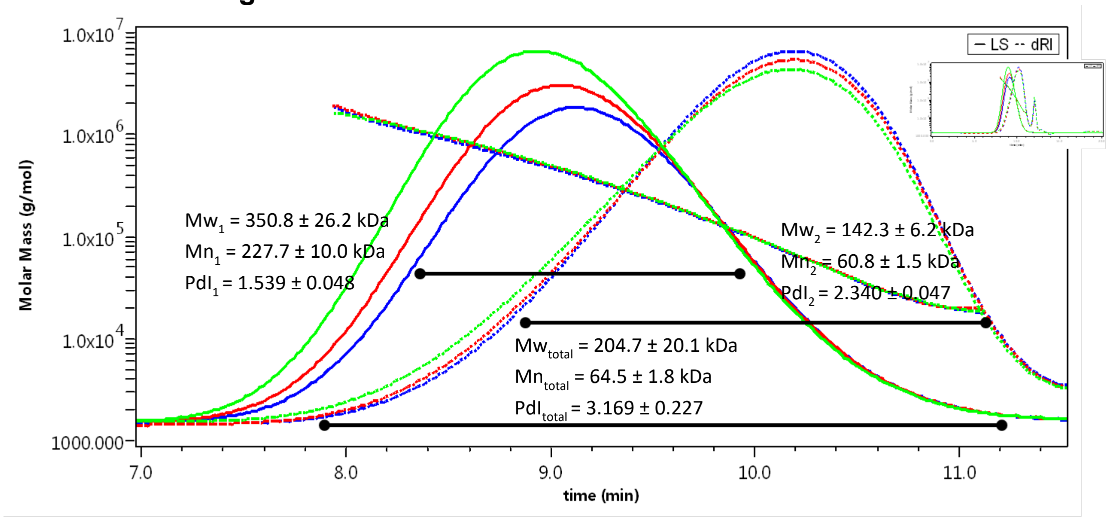

Figure II-22. Molecular weight of NCL388-3. (A) RI response for five different concentrations of NCL388-3. **(B)** Plots of the change in refractive index (dn) versus the change in concentration (dc) used for dn/dc determination. **(C)** SEC chromatograms for NCL388-3. The solid and dashed lines represent the light scattering and RI signals, respectively. The molar mass distribution is shown and was calculated using the measured dn/dc values.

Size Distribution and Molecular Weight by Asymmetric-Flow Field Flow Fractionation

Design and Methods

Particle size distribution for NCL388-3 was also measured using asymmetric-flow field flow fractionation (AF4) coupled with a DLS detector. The AF4 system consisted of an isocratic pump (Agilent G1310A, Palo Alto, CA), well-plate autosampler (Agilent G1329A), UV-Vis detector (Agilent G1315B), AF4 separation channel (Eclipse 2, Wyatt Technology, Santa Barbara, CA), MALS detector (Wyatt HELEOS II), refractive index (RI) detector (Wyatt OptiLab T-rEX), and a dynamic light scattering (Malvern Zetasizer Nano ZS) instrument. The separation channel had a length of 275 mm and a 350 µm spacer. A regenerated cellulose membrane was used for particle separation. The elution profile is given in Table II-22. The cross flow was controlled by an Eclipse 2 flow controller. The detector flow was 1 mL/min, and the injection volume was 100 µL for all samples.

The mobile phase was PBS (Hyclone) which was filtered through a 0.2 µm regenerated cellulose membrane prior to use. Samples of NCL388-3 were diluted 100-fold in PBS prior to injection. The chromatographic traces were monitored by RI, MALS and DLS detection. A Malvern Zetasizer Nano ZS instrument (Southborough, MA) with back scattering detector (173°) was used for measuring the hydrodynamic diameter in flow-mode. Measurements were made in a quartz flow cell (Malvern ZEN0023) and data was collected using Malvern Zetasizer software (v7.11). The intensity threshold was manually adjusted after each experiment. MALS normalization constants were determined by running BSA at 5 mg/mL in PBS.

Table II-22. AF4 elution profile.

Step	delta t [min]	Mode	Cross-Flow @ start [mL/min]	Cross-Flow @ end [mL/min]	Focus Flow [mL/min]
1	2	Elu.	0	0.5	-
2	2	Focus	0.5	0.5	1
3	5	Foc+Inj	0.5	0.5	1
4	5	Focus	0.5	0.5	1
5	30	Elu.	0.5	0.5	-
6	2	Elu.	0.5	0	-
7	7	Elu.	0	0	-
8	2	Elu+Inj	0	0	-
9	5	Elu	0	0	-

Results and Discussion

The resulting fractograms for AF4 separation based on MALS and DLS detection of NCL388-3 are shown in Figures II-23 and II-24, respectively. The NCL388-3 showed a single peak. Using both the light scattering (solid trace) and refractive index (dashed trace) signals and the measured dn/dc value (Figure II-22B), the molar mass distribution across the peak was calculated (Figure II-23). Similar to the SEC-MALS results, the light scattering and refractive index signals do not overlay, Hence, the molar mass was determined based on each of these signals along with the overall molar mass across both signals. The molar mass based on the light scattering signal was 362 kDa. The molar mass based on the refractive index signal was 163 kDa. The overall molecular weight across the entire peak (which takes into account contributions from both signals) was 268 kDa. These molar masses were consistent with the results from the SEC-MALS experiments.

In addition to the molecular weight determination, the hydrodynamic size was measured across the peak (Figure II-24). The measured size ranged from 15 to 75 nm, with an average size of 21 nm. This was consistent with the measured batch-mode DLS results.

A. AF4-MALS

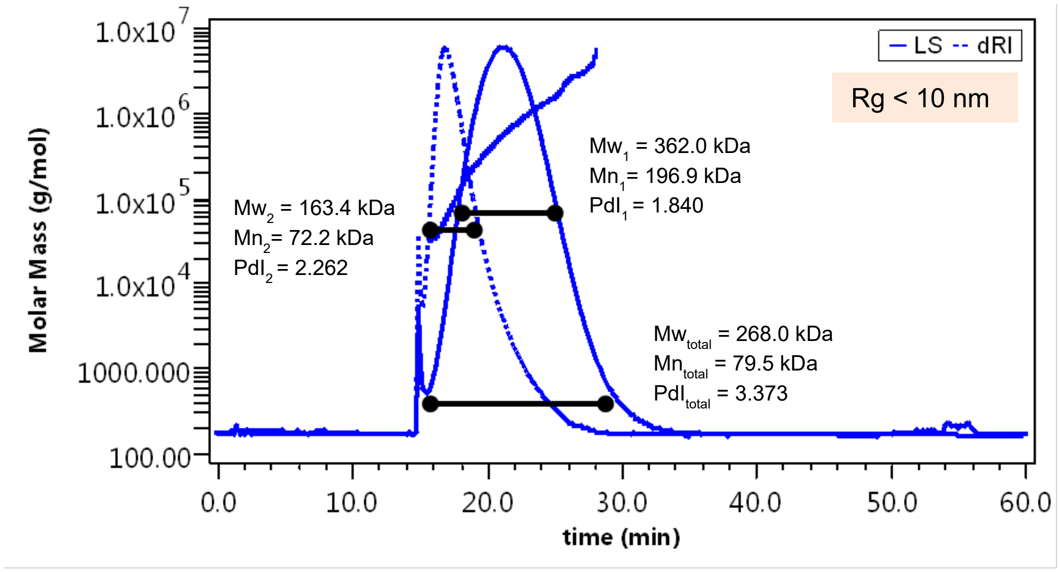

B. AF4-DLS

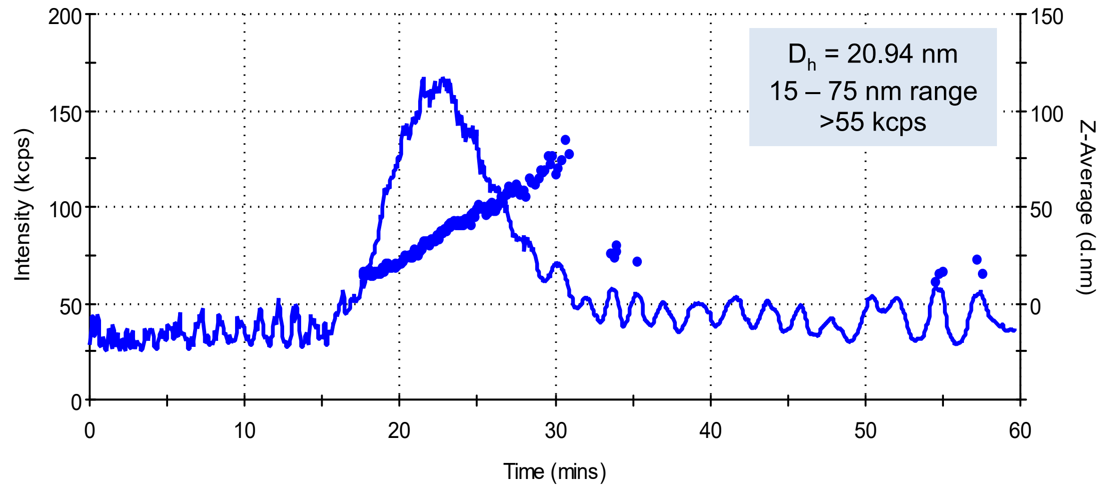

Figure II-23. Flow-mode diameter (AF4 separation with in-line MALS and DLS). (A) The radius of gyration was measured across the peak using a MALS detector. MW_1 is the molecular weight from the light scattering distribution. MW_2 is the molecular weight from the RI distribution (concentration). **(B)** The hydrodynamic size was measured using a Malvern Zetasizer and was based on an intensity threshold of >48 kcps. The size ranges for the major peak are given in the figure.

Total Iodine Concentration by Inductively Coupled Plasma-Mass Spectrometry

Design and Methods

The iodine concentration of NCL388 was determined by inductively coupled plasma mass spectrometry (ICP-MS) using a Perkin-Elmer NexION 2000B equipped with a micro-mist nebulizer, standard sample introduction system, and integrated auto-sampler, operated in standard mode. Tuning of the instrument was performed daily prior to sample testing. Masses analyzed included I-127,In-115, and Te 130 (internal standards). Data was analyzed using the Syngistics software.

NCL388-1 & NCL388-2

Calibration standards were prepared using NaI (Sigma Aldrich, 409286, 99.9999% purity). Calibrants were made at final dilutions of 0, 1, 2, 5, 10, and 25 ng/g using 1% tetramethylammonium hydroxide (TMAH, Sigma Aldrich, 331635-250ML). NCL388 samples (15 µL) were diluted using 4.5 mL distilled water and 0.5 mL 25% TMAH. Samples were then placed in a microwave according to the program outlined in Table II-23 below. After being microwaved, samples were diluted using 1% TMAH to fall within the calibration range, estimating the dilution factor based upon the sponsor's reported sample concentration. A minimum of two independent samples were prepared and measured in triplicate. ^{130}Te was used as an internal standard and was added using a sample T.

Table II-23. Microwave digestion program. NCL388-1 and NCL388-2 were microwave-digested prior to ICP-MS analysis according to the following protocol.

Step	Power (W)	Power Setting (%)	Hold Time (min)
1	400	100	05:00
2	800	100	03:00
3	400	100	05:00

NCL388-4 through NCL388-11

Samples of NCL388-4 through NCL388-11 were analyzed similarly to the methods described above except that the microwave step was omitted. Without the microwave step, complete release of iodine from the nanoparticles was not possible and hence resulted in much lower iodine concentrations compared to the client-reported values. For this reason, these data are omitted.

NCL388-12

Samples were prepared by adding 50 µL NCL388-12 (labeled as high dose #1 and #2; the same as used in the in vivo toxicology study) to 15 mL Falcon centrifuge tubes. The solutions were diluted to 10 mL using 1% tetramethylammonium hydroxide (TMAH, Sigma Aldrich, diluted from 25% by weight solution), and weights were measured at each step before addition of sample, after addition of sample, and after dilution (Dilution #1). Two (2) x 1 mL of Dilution #1 was added to each of 6 5 mL PFA microwave tubes. The tubes were sealed and microwaved according to the procedure in Table II-24. The samples were then diluted by diluting 100 µL of microwaved Dilution #1 to 10 mL in Falcon centrifuge tubes using 1% TMAH (Dilution #2). Again, weights were recorded as in Dilution #1. Finally, the solutions were diluted a final time by diluting 300 µL to 10 mL using 1% TMAH (Dilution #3). The weights were recorded as in Dilution #1.

Calibration standards were prepared from NaI (99.999% trace metal basis, Sigma Aldrich) and serial dilution with 1% TMAH to achieve the final concentrations necessary for iodine quantitation. Calibration standards had concentrations of 0, 20.550, 40.677, 62.637, 81.488, 124.226, and 205.684 ng/g.

Iodine concentration was determined using an external calibration constructed using the standards above. Three points per mass were measured with an integration time of 0.06 seconds. Ten replicates per measurement were averaged to give the final iodine counts. A 20 ppb solution of telluriumin, 1% TMAH, was used as an internal standard and added via a sample T to analyte solution.

Table II-24. Microwave digestion program. NCL388-12 was microwave-digested prior to ICP-MS analysis according to the following protocol.

Step	Power (W)	Power Setting (%)	Hold Time (min)
1	400	100	05:00
2	800	100	05:00
3	400	100	05:00

Results and Discussion

The iodine concentrations in NCL388 were measured using ICP-MS. The averaged iodine concentration for NCL388-1 is summarized in Table II-24 below. The calibration curves used to extrapolate the concentrations are shown in Figure II-24. The NCL388-1 iodine concentration was 65.3 ± 0.3 mg/mL, in very good agreement with the sponsor's theoretical value of 65 mg/mL.

Samples of NCL388-1 were filtered through Mustang filters to help reduce the endotoxin contamination. This filtered sample was designated as NCL388-2. For comparison, NCL388-1 was run in parallel with NCL388-2. The calibration curves used to extrapolate the concentrations are shown in Figure II-25 and summarized in Table II-24. The repeated NCL388-1 iodine concentration was 88.8 ± 0.3 mg/mL. The iodine concentration for the NCL388-2 was 82.8 ± 0.3 mg/mL. This was repeated again and resulted in an iodine concentration of 88 ± 4 mg/mL. While these values are higher than the initial analysis (results included in the table), the difference between unfiltered (NCL388-1) and filtered (NCL388-2) was ~7% suggesting that the filtration did not remove any iodine.

Samples of NCL388-4, NCL388-5, NCL388-6, NCL388-9, NCL388-10, and NCL388-11 were pooled together and designated as NCL388-12. The calibration curves used to extrapolate the concentrations are shown in Figure II-26 and summarized in Table II-25. Two samples, labeled as high dose #1 and high dose #2, were measured. These samples correspond to the high dosing solutions used in the in vivo toxicology study. The iodine concentration for high dose #1 and high dose #2 were 71.4 ± 10.8 and 65.8 ± 6.0 mg/mL, respectively. These values are in the range of the sponsor's reported concentration for the individual NCL388 lots that were pooled.

Original Run

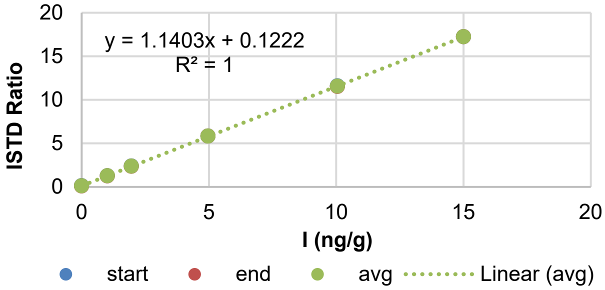

Standard Concentration	Starting Signal	Ending Signal	Average Signal
0	0.12089	0.098696	0.109793
1.01208912	1.274512	1.245026	1.259769
1.955189653	2.380301	2.35119	2.365746
4.968526664	5.822392	5.825193	5.823793
10.0481904	11.59496	11.51634	11.55565
15.00138151	17.23658	17.22924	17.23291

Repeat & Filtered Runs

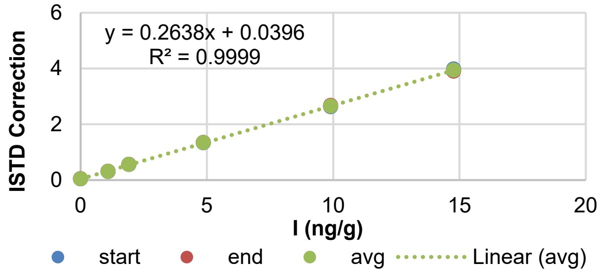

Standard Concentration	Starting Signal	Ending Signal	Average Signal
0	0.04058	0.043061	0.04182
1.092668	0.302516	0.309334	0.305925
1.913352	0.556034	0.556767	0.5564
4.860223	1.330401	1.343121	1.336761
9.904097	2.623249	2.668746	2.645998
14.77081	3.973125	3.899359	3.936242

Figure II-24. Calibration Curves. The calibration curves (corrected counts (normalized by internal std) versus concentration) used for the determination of I concentration in NCL388-1.

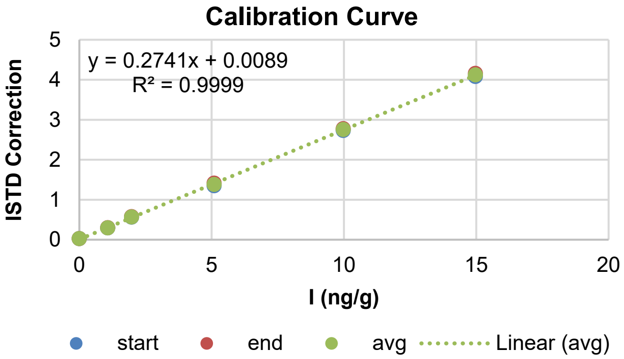

Standard Concentration	Starting Signal	Ending Signal	Average Signal
**0**	**0.02464**	**0.023717**	**0.024179**
1.07891	0.290852	0.295166	0.293009
1.978309	0.55798892	0.572114	0.565052
5.096391	1.34481743	1.409608	1.377213
9.979739	2.7296626	2.773379	2.751521
14.96865	4.07799168	4.154579	4.116285

Figure II-25. calibration curve. The calibration curve (corrected counts (normalized by internal standard) versus concentration) used for the determination of I concentration in NCL388-2.

Table II-24. Iodine concentration in NCL388-1 and NCL388-2 as determined by ICP-MS.

Sample	[Iodine] (mg/mL)
NCL388-1 (Initial)	65.3 ± 0.9 (n=6)
NCL388-1 (Repeat)	88.8 ± 0.3 (n=9)
NCL388-1 (Filtered)	82.8 ± 0.3 (n=9)
NCL388-2	88 ± 4 (n=9)

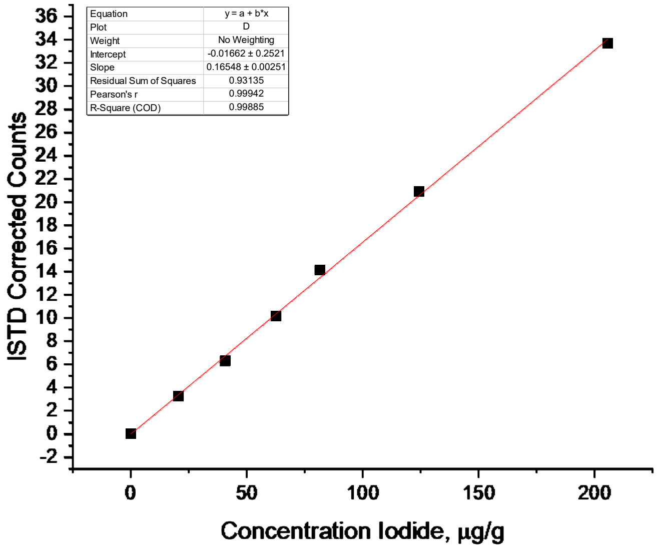

Figure II-26. calibration curve. The calibration curve (corrected counts (normalized by internal standard) versus concentration) used for the determination of I concentration in NCL388-12.

Table II-25. Iodine concentration in NCL388-12 as determined by ICP-MS.

Sample	[Iodine] (mg/mL)
High Dose #1	71.4 ± 10.8 (n=6)
High Dose #2	65.8 ± 6.0 (n=6)

Total Iodine Concentration by UV-Vis Spectroscopy

Design and Methods

As an alternative to ICP-MS, iodine concentration was also measured using UV-vis spectroscopy. The total iodine concentration in NCL388-3 was measured using UV-vis with detection at 240 nm (Perkin Elmer Lambda 35). Calibration standards were prepared from NCL388-1, whose iodine concentration was measured using the more precise technique of ICP-MS (64 mg/mL; see Table II-24). Calibration standards were prepared at 1, 2, 4, 8,16 µg/mL iodine in PBS.

For analysis of NCL388-3, the sample was diluted 16,000-fold in PBS as follows:

Step I: 10 µL stock + 990 µL PBS
Step II: 50 µL Step 1 + 950 µL PBS
Step III: 100 µL Step II + 700 µL PBS

All samples were measured in quartz microcuvettes (path length, b = 10 mm, QS105.250, Hellma, Plainview, NY). Spectra were collected from 200-800 nm at 480 nm/min in 1 nm steps against PBS as the reference.

Results and Discussion

The total iodine in NCL388-3 was measured using UV-vis detection at 240 nm, against a calibration standard of NCL388-1, whose iodine concentration was previously confirmed by ICP-MS. The total iodine concentration in NCL388-3 was 50.9 ± 2.2 mg/mL (n=5) (Figure II-27), approximately 25% lower than the theoretical concentration reported by the sponsor. The sponsor reported a concentration of 70 mg/mL.

Visual inspection of the sample also supported a lower sample concentration for NCL388-3 as compared to NCL388-1. NCL388-3 was less viscous and was lighter in color as compared to the more concentrated NCL388-1 sample.

NCL388-1 Calibration Standards

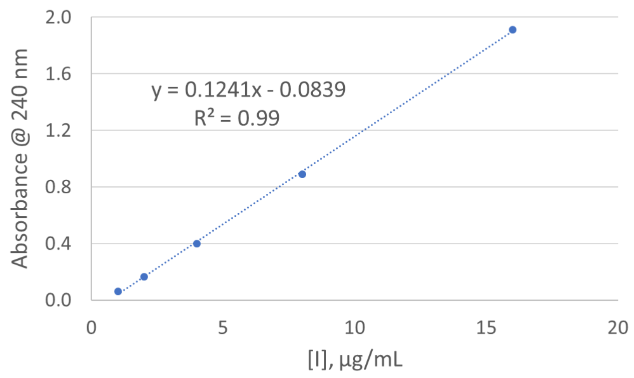

[I] µg/mL	Abs. (240 nm)
1	0.0616
2	0.1651
4	0.3965
8	0.8916
16	1.9130

UV traces for NCL388-3 and the NCL388-1 Calibration Standards

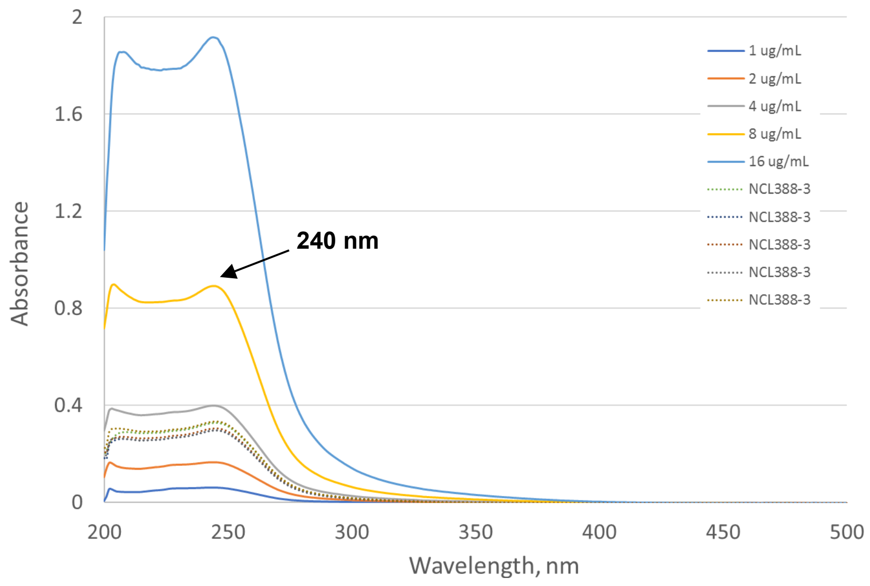

Run	Abs. (240nm)	[I] mg/mL
#1	0.3034	49.9
#2	0.2973	49.1
#3	0.3327	53.7
#4	0.3267	52.9
#5	0.2964	49.0
**Average**		**50.9 ± 2.2**

Figure II-27. Total Iodine in NCL388-3. Total iodine in the sample was measured by UV-vis at 240 nm. Five replicates were analyzed. The NCL388-3 traces are overlaid with the NCL388-1 calibration standards. The values shown in the table have been corrected for dilution.

Iohexol and PEG Concentration by Thermogravimetric Analysis

Design and Methods

The objective of this experiment was to determine the PEG+linker and iohexol concentrations in NCL388-3 using thermogravimetric analysis (TGA). Samples of free iohexol and PBS (to correct for residual salts) were run as controls. For analysis of PBS, 35 µL of sample was used. Iohexol was supplied as a powder and used approx. 7.5 mg. NCL388-3 was first lyophilized to remove/reduce the water peak which overlaid with the PEG+linker decomposition. Approximately 9 mg of lyophilized material was used for the TGA run. Two samples each were run for iohexol and NCL388-3.

Samples were transferred to an aluminum oxide crucible (150 µL crucible with lid, Mettler Toledo) for TGA measurements (TGA/DSC 1, Mettler Toledo). Samples were held at 25˚C for 5 min, and then ramped to 1100˚C at a heating rate of 20˚C/min under nitrogen gas. For each sample, a new crucible was used. The empty crucible was subjected to the TGA method prior to loading the sample to serve as the background correction.

Results and Discussion

The PEG+linker and iohexol concentrations in NCL388-3 was determined using thermogravimetric analysis (TGA). NCL388-3 was supplied as a dispersion in PBS. Initial TGA analysis of NCL388-3 as is, i.e. in solution, resulted in a huge water peak which dominated the thermogram and made analysis difficult (data not shown), Thus, NCL388-3 was lyophilized and the resulting powder was subjected to TGA analysis. Since NCL388-3 was dispersed in PBS, the contribution of the salts and any other buffer components was determined by performing TGA on the PBS solution alone. The resulting thermogram for 35 µL PBS is shown in Figure II-28 and resulted in 0.071 mg residual mass. This corresponds to approximately 2 mg/mL total buffer component mass in the stock sample; this was used to correct for the residual buffer components in the final residual mass of NCL388-3.

Free iohexol (solid) was also run as a control and its thermogram is shown in Figure II-29. Based on the first derivative plot (lower panel in Figure II-29), the major decomposition event occurs at ~300°C. Iohexol did not completely decompose; based on two runs (only one run is shown in Figure II-29), 78% of iohexol decomposes over the temperature range examined. This corresponds to ~22% residual iohexol and was used along with the final residual mass of NCL388-3 to determine the amount of iohexol in NCL388-3.

The thermogram for lyophilized NCL388-3 is shown in Figure II-30. Note, two independent lyophilized samples were run (only one data set is shown in Figure II-30). Two decomposition events are observed in the thermogram; the first occurring at 50-150°C which is assumed to be the decomposition of PEG + linker, and the second at ~ 300°C corresponding to the decomposition of iohexol. The starting (green) and residual (red) masses after each decomposition event are boxed in figure and were used for calculating the amount of iohexol and PEG + linker in NCL388-3. The calculations that follow assume that the linker decomposes with the PEG (free linker control was not available to confirm this) and that all of the PEG and linker decompose in the first decomposition event. It also assumes that the residual mass at the end of the run is due to incomplete iohexol decomposition and the residual buffer components from PBS.

Gravimetric analysis of two samples of NCL388-3 (30 µL) was performed to determine the total construct concentration. The masses before and after lyophilization are given in Table II-26. The average of the two runs gave a total construct + PBS concentration of 240 mg/mL or 238 mg/mL total construct after correcting for buffer components (determined as 2 mg/mL from TGA analysis described earlier).

Calculations are described for aliquot 1, and the results are given in Tables II-27A-C. The final residual mass of ioxehol is calculated by subtracting the residual buffer components correction (0.0742 mg) from the TGA residual mass (1.3813 mg from Figure II-28) to give 1.3071 mg. This value is then back-calculated to determine the amount of starting iohexol based on the free ioxehol run, or 1.3071 / 0.22 = 5.9904 mg iohexol. Dividing this by the starting mass (8.7574 mg) gives 68.7% (w/w) iohexol or 163.6 mg/mL iohexol (calculated by multiplying 238 mg/mL total construct by 68.7%). Similar analysis of the second run resulted in 69.0 %(w/w) iohexol, very consistent with the first run. The amount of PEG+linker is calculated by subtracting the mass at 200°C (6.3007 mg from Figure II-30) from the starting mass (8.7574 mg) and adding the PBS contribution (0.0742 mg) to give 2.5309 mg PEG+linker. This corresponds to 28.9% (normalized to the starting mass). Similar analysis of the second run resulted in a 28.6 %(w/w) PEG+linker, very consistent with the first run.

Table II-26. Gravimetric analysis of NCL388-3.

NCL388-3 Gravimetric Analysis	Vial Mass (mg)	Mass after sample lyophilization (mg)	Lyophilized Sample Mass (mg)	Sample concentration (mg/mL)
Aliquot 1	1098.93	1105.72	6.79	226
Aliquot 2	1095.96	1103.59	7.63	254
				Average: 240 mg/mL total construct + PBS<br>Corrected: **238 mg/mL** total construct

Table II-27. Quantitation of PEG + linker concentration in NCL388-3. Shown are the values used to calculate the PEG + linker concentration of NCL388-3 based off of the TGA analyses.

A.

Lyophilized NCL388-3 samples	Residual buffer component correction (mg)	Starting mass (mg)	Mass at 200° (mg) (loss of PEG + linker)	Final Mass (mg) (partial decomposition of iohexol)
Aliquot 1	0.0742	8.7574	6.2265	**1.3071**
Aliquot 2	0.0870	10.2632	7.3314	**1.5462**

B.

	Final mass corrected for buffer components (mg)	Theoretical IH in original sample (mg)	Percent IH in original sample (w/w)%	Average percent IH (w/w)%<br>-----<br>Calculated IH concentration (mg/mL)
Aliquot 1	1.3071	5.9904	68.4%	**68.7%**
Aliquot 2	1.5462	7.0862	69.0%	**163.6 mg/mL**

C.

	Calculated Mass of PEG + linker (mg)	Percent PEG + Linker in original sample (w/w)%	Average percent PEG + linker (w/w)%
Aliquot 1	2.5309	28.9%	**28.7%**
Aliquot 2	2.9318	28.6%	

PBS

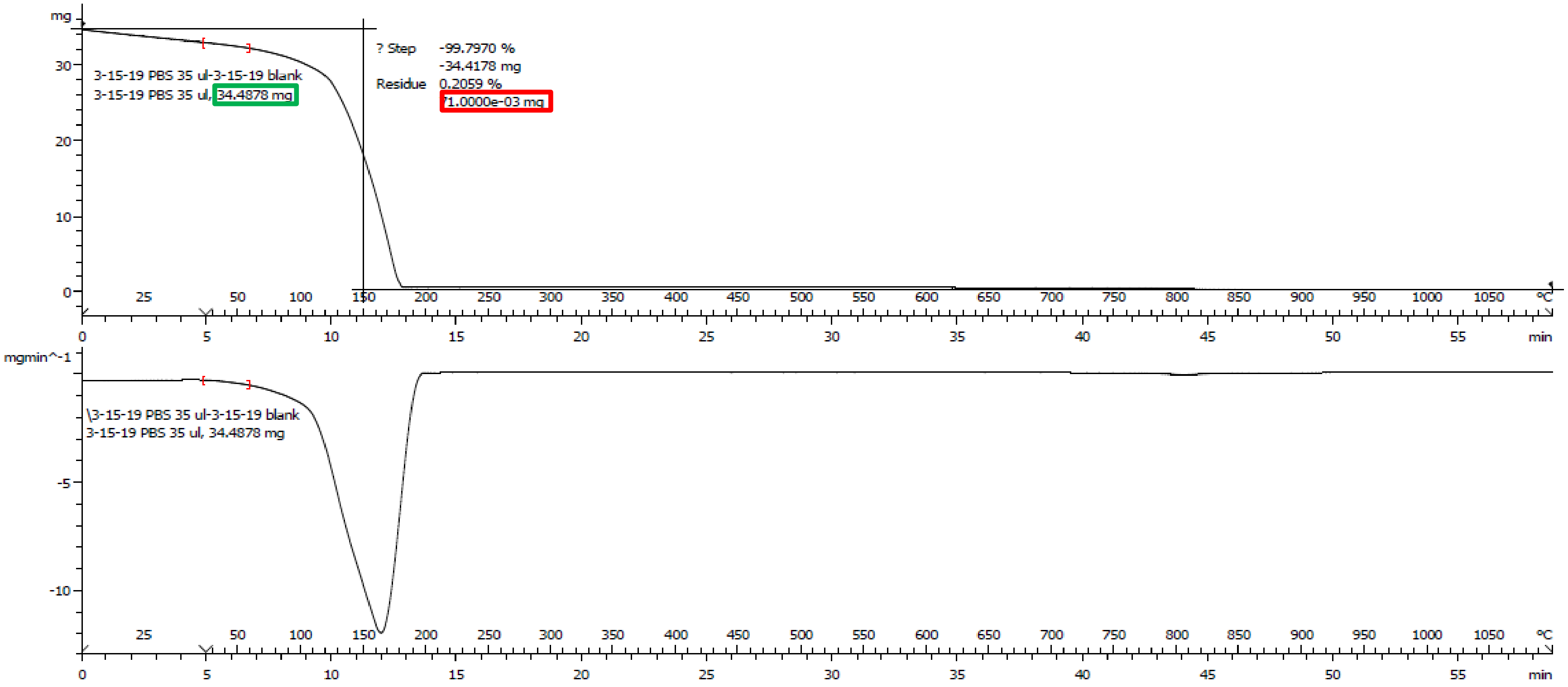

Figure II-28. TGA thermogram of PBS. The top panel is the weight loss versus temperature (and time) curve. The bottom panel is the first derivative of that curve. The starting (green) and residual (red) masses are boxed; 2 mg/mL residual buffer component mass remained at the end of the TGA run. This amount was used for corrections in the final calculations of PEG+linker and iohexol concentrations.

Iohexol

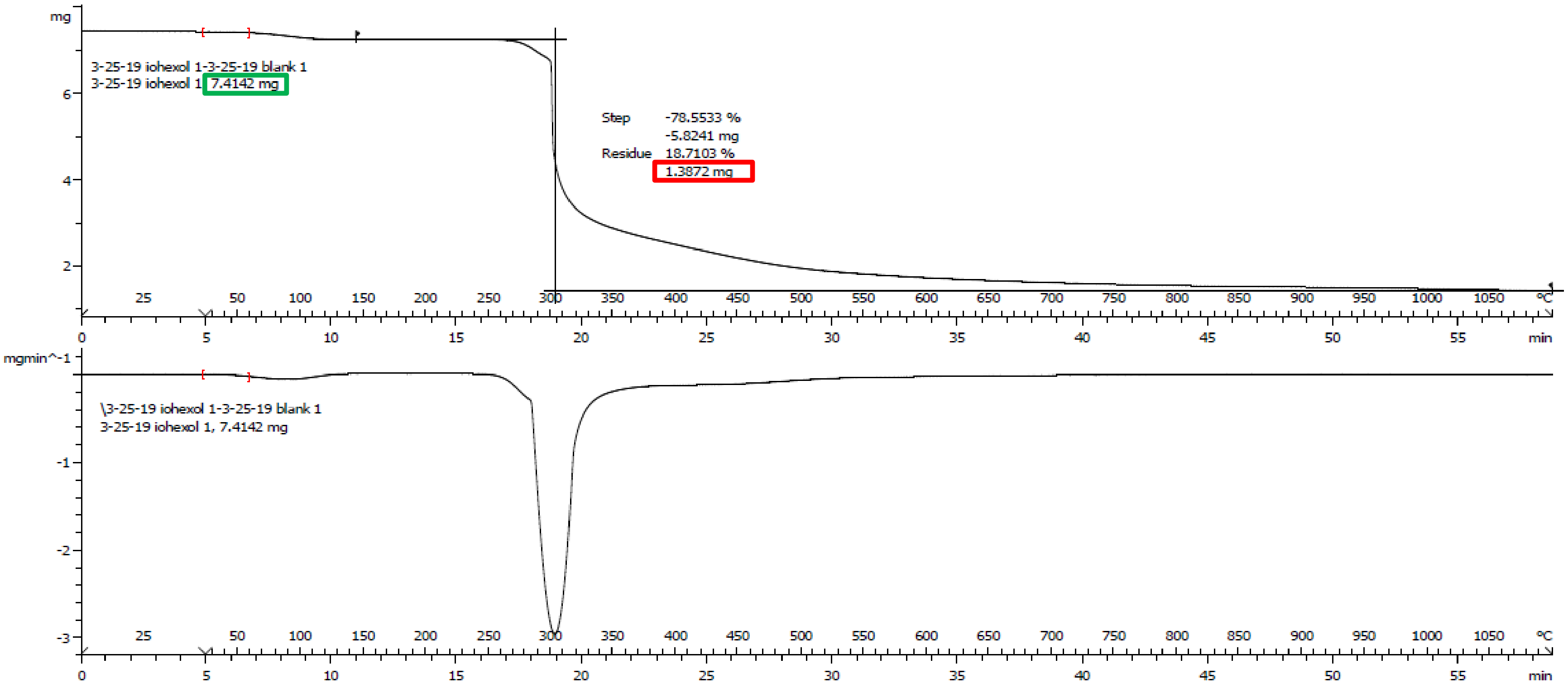

Figure II-29. TGA thermogram of iohexol. The top panel is the weight loss versus temperature (and time) curve. The bottom panel is the first derivative of that curve. The starting (green) and residual (red) masses are boxed; ~22% mass remained at the end of the TGA run.

NCL388-3

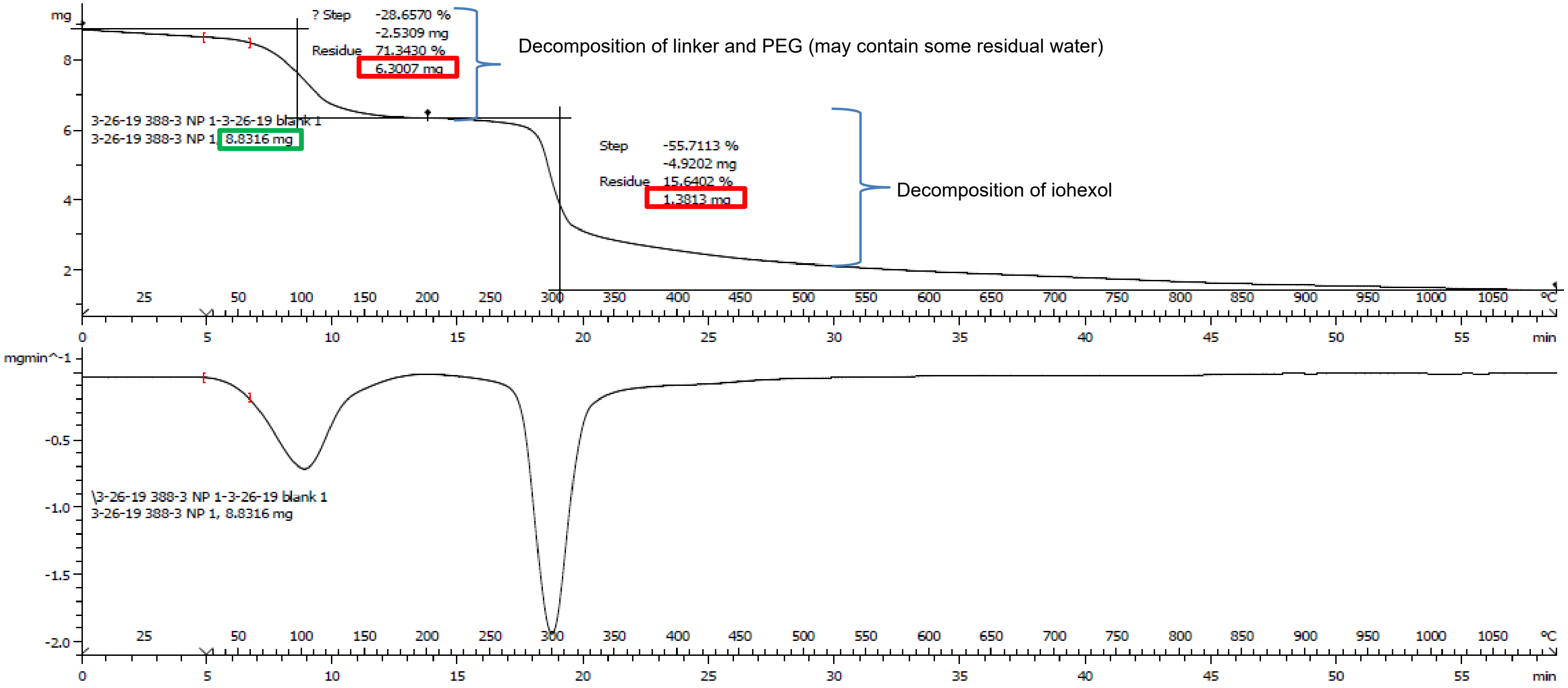

Figure II-30. TGA thermogram of NCL388-3. The top panel is the weight loss versus temperature (and time) curve. The bottom panel is the first derivative of that curve. The starting (green) and residual (red) masses after each decomposition event are boxed; ~16% mass remained at the end of the TGA run.

III. In Vitro Cytotoxicity

Section Summary

The in vitro cytotoxicity of NCL388-3 was evaluated in two cell lines, porcine renal proximal tubule (LLC-PK1) and human hepatocarcinoma (Hep G2) cells. The formulation was tested over a concentration range of $2x10^{-2}$ - 5.09 mg/mL iodine. NCL388-3 showed greater toxicity to the LLC-PK1 cells than the Hep G2 cells. The estimated IC_{50} values for NCL388-3 in LLC-PK1 and Hep G2 cells were 0.89 and 5.03 mg/mL, respectively.

Lysotracker Red signal followed the same concentration and time profile as NCL388-3 cytotoxicity in LLC-PK1 cells, and is consistent with autophagy dysfunction that has been described as a toxic mechanism for many biopersistent nanomaterials [3]. Autophagy dysfunction is a potential mechanism of NCL388-3-induced cell death.

Table III-1. IC_{50} values of NCL388-3. The estimated IC_{50} values determined by log-probit analysis of the 48 hr MTT cytotoxicity dose-response curves are summarized.

Samples	IC_{50} mg/mL iodine	
	LLC-PK1	Hep G2
NCL388-3	0.89	5.03

LLC-PK1 Cytotoxicity Assays, MTT and LDH Release (GTA-1)

Design and Methods

The objective of this study was to evaluate the in vitro cytotoxicity of NCL388-3 in porcine renal proximal tubule (LLC-PK1) cells. Cytotoxicity was determined as described in NCL protocol GTA-1, LLC-PK1 Kidney Cytotoxicity Assay (https://ncl.cancer.gov/resources/assay-cascade-protocols). Briefly, test materials were diluted to the desired assay concentrations in cell culture media ($2x10^{-2}$ - 5.09 mg/mL iodine). Taxol (125 mM) was used as a positive control and media was used as a negative control. Cells were plated in a 96-well, microtiter plate format. Cells ($2.5x10^{5}$ cells/mL) were preincubated for 24 hr prior to test material addition, reaching an approximate confluence of 80%. Cells were then exposed to test material in triplicate for 4, 24, and 48 hr in the dark, and cytotoxicity was determined using the 3-(4,5-dimethylthiazol-2-yl)-2,5-diphenyltetrazolium bromide (MTT) cell viability and lactate dehydrogenase (LDH) membrane integrity assays. The IC_{50} values were estimated directly from the 48 hr MTT cytotoxicity assay dose-response data using log-probit analysis (Finney method; BioStat Professional 5.8.4, AnalystSoft, Inc., Vancouver, Canada).

Results and Discussion

NCL388-3 showed concentration-dependent toxicity in the LLC-PK1 cells at concentrations >0.5 mg/mL iodine at the 24 and 48 hr time points. The MTT data concentration and time profile correlated with the LDH leakage assay (Figure III-1), although the magnitude of the responses in the two assays were different most likely due to the loss of LDH activity over time in the cell culture media. The estimated IC_{50} value for NCL388-3 was 0.89 mg/mL using the 48 hr MTT data.

A. NCL388-3 MTT Assay

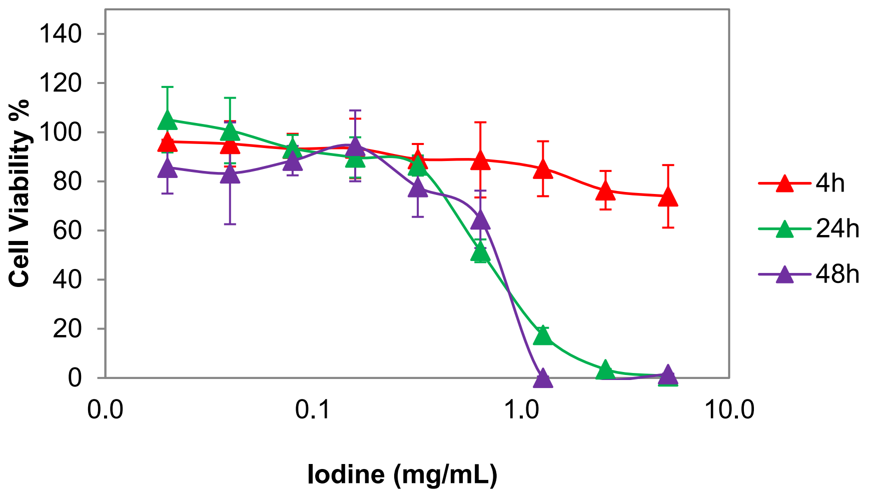

B. NCL388-3 LDH Assay

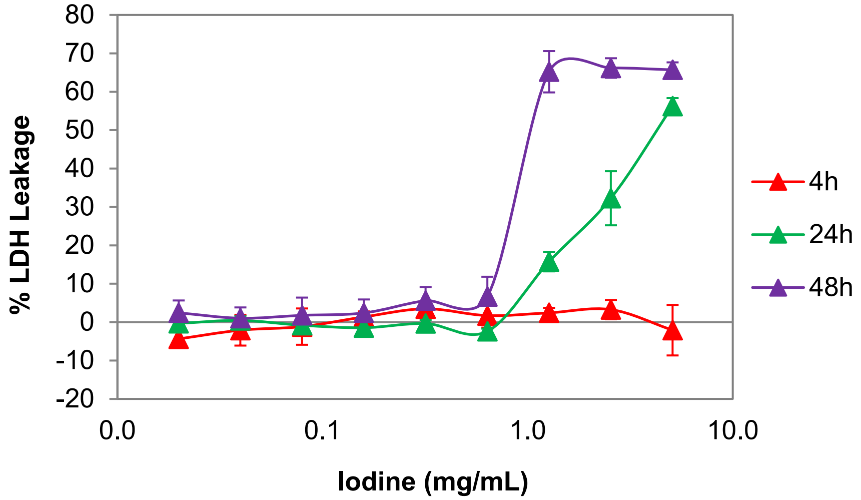

Figure III-1. NCL388-3 cytotoxicity assay in LLC-PK1 cells. Porcine renal proximal tubule cells were treated for 4, 24, and 48 hr with NCL388-3. Cytotoxicity was determined at each time point by the (**A**) MTT and (**B**) LDH assays, as described in the LLC-PK1 Kidney Cytotoxicity Assay (GTA-1). Data represents the mean ± SE, *N*=3.

Hep G2 Cytotoxicity Assay, MTT and LDH Release (GTA-2)

Design and Methods

The objective of this study was to evaluate the in vitro cytotoxicity of NCL388-3 in human hepatocarcinoma (Hep G2) cells. Cytotoxicity was determined as described in NCL protocol GTA-2, Hep G2 Human Hepatocarcinoma Cytotoxicity Assay (https://ncl.cancer.gov/resources/assay-cascade-protocols). Briefly, test materials were diluted to the desired assay concentrations in cell culture media ($2x10^{-2}$ - 5.09 mg/mL iodine). Acetaminophen (20 mM) was used as a positive control, and media was used as a negative control. Cells were plated in 96-well, microtiter plate format. Cells ($5x10^{5}$ cells/mL) were preincubated for 24 hr prior to test material addition, reaching an approximate confluence of 80%. Cells were then exposed to test material in triplicate for 4, 24, and 48 hr in the dark, and cytotoxicity was determined using the MTT cell viability and LDH membrane integrity assays. The IC_{50} values were estimated directly from the 48 hr MTT cytotoxicity assay dose-response data using log-probit analysis (Finney method; BioStat Professional 5.8.4, AnalystSoft, Inc., Vancouver, Canada).

Results and Discussion

NCL388-3 was toxic to the Hep G2 cells the highest tested concentration at the 24 and 48 hr time points. The MTT data correlated with the LDH leakage assay for the 48 hr time point (Figure III-2). The estimated IC_{50} value for NCL388-3 was 5.03 mg/mL using the 48 hr MTT data.

A. NCL388-3 MTT Assay

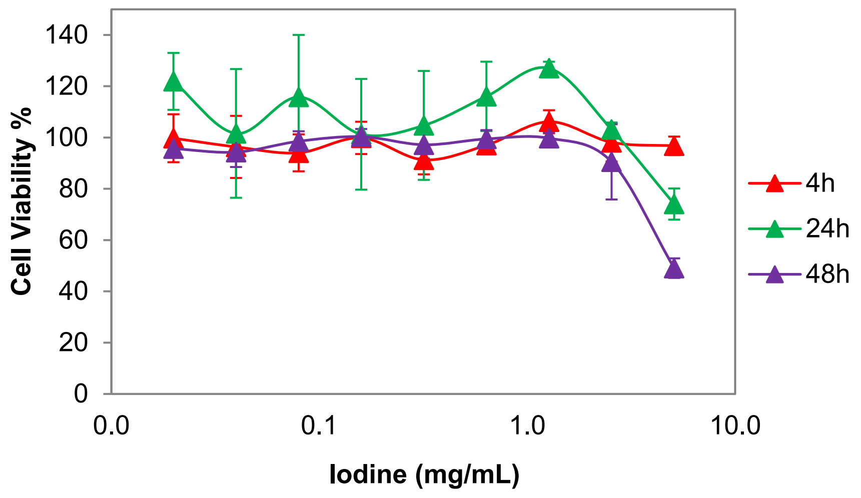

B. NCL388-3 LDH Assay

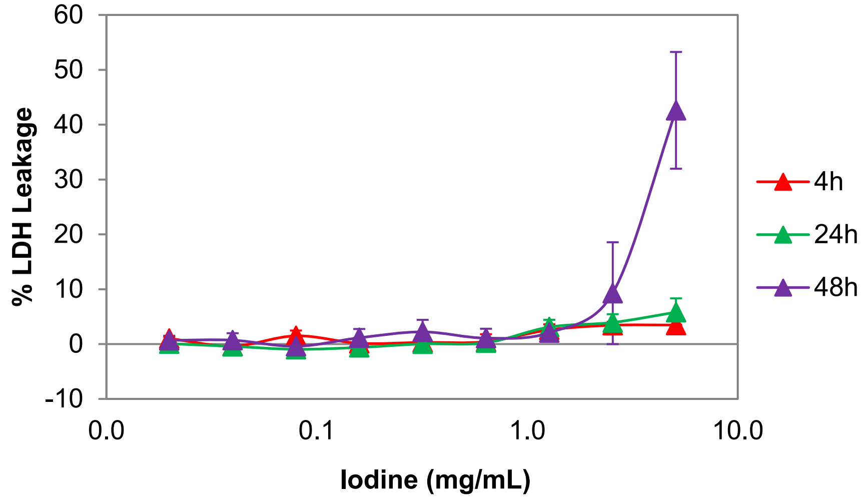

Figure III-2. NCL388-3 cytotoxicity assay in Hep G2 cells. Human hepatocarcinoma cells were treated for 4, 24, and 48 hr with NCL388-3. Cytotoxicity was determined at each time point by the (**A**) MTT and (**B**) LDH assays, as described in the Hep G2 Hepatocarcinoma Cytotoxicity Assay (GTA-2). Data represents the mean ± SE, *N*=3.

Autophagic Dysfunction in LLC-PK1 Cells

Design and Methods

Autophagic dysfunction was investigated as a mechanism of toxicity for NCL388-3 using LLC-PK1 cells as a model cell line, as described in NCL protocol GTA-12, Autophagic dysfunction in LLC-PK1 cells (https://ncl.cancer.gov/resources/assay-cascade-protocols). Briefly, LLC-PK1 cells were counted using a hemocytometer and diluted in media to achieve 1x 10^5 cells/mL. Cells (100 µL) were then added to each well in rows A-C and F-H of three 96-well plates, while rows E and F remained cell-free background control that was subtracted from the A-C and F-H fluorescence, respectively. The cells were allowed to grow for 24 hr. Following this incubation period, the media was removed, and cells washed twice with phenol red-free media. Next, 100 µL of phenol red-free RPMI media containing NCL388-3 (0-2.5 µM Iodine, 9 dilutions) was added to each well of a column, with one column treated with media alone. One plate each was incubated for 4, 24, and 48 hr. At the end of each time point, the plate was washed twice with phenol red-free media (200 µL), and 100 µL of working solution (50 nM Lysotracker Red/ 10 µM Celltracker Green phenol red-free media) was added to each well. The plates were incubated for 1 hr at 37°C, then washed twice with phenol red-free media (200 µL). Lastly, 100 µL of fresh phenol red-free media was added and the fluorescence was measured (Ex_1=492nm, Em_1=517nm, Ex_2=544nm, Em_2=-590nm). To verify results of this microplate assay, cells were grown on 8-well glass microscope slides, treated identically as in the microplate assay, and imaged by confocal microscopy in red and green channels.

Results and Discussion

The Lysotracker Red signal (normalized by the Cell Tracker Green signal) increased concentration-dependently at the 24 hr time point, and followed a hyperbolic concentration profile at the 48 hr time point. The increase in Lysotracker Red signal followed the same concentration and time profile as NCL388-3 cytotoxicity in this cell line, and is consistent with autolysosome/lysosome accumulation that can result from either induction or blockade autophagy pathway. Autophagy dysfunction has been described as a toxic mechanism for many biopersistent nanomaterials [3]. The decrease in normalized signal at the highest concentrations at 48 hr was likely the result of severe cytotoxicity that could not be accurately normalized with the Cell Tracker Green dye, used to account for viable cell number. This apparent increase in lysosome vesicles was verified by confocal microscopy, which demonstrated a concentration-dependent increase in Lysotracker Red labeled vesicles at the 24 and 48 hr time points, with loss of Cell Tracker Green labeled viable cells at the highest concentrations at 48 hr. Autophagy dysfunction is a potential mechanism of NCL388-3 cell death.

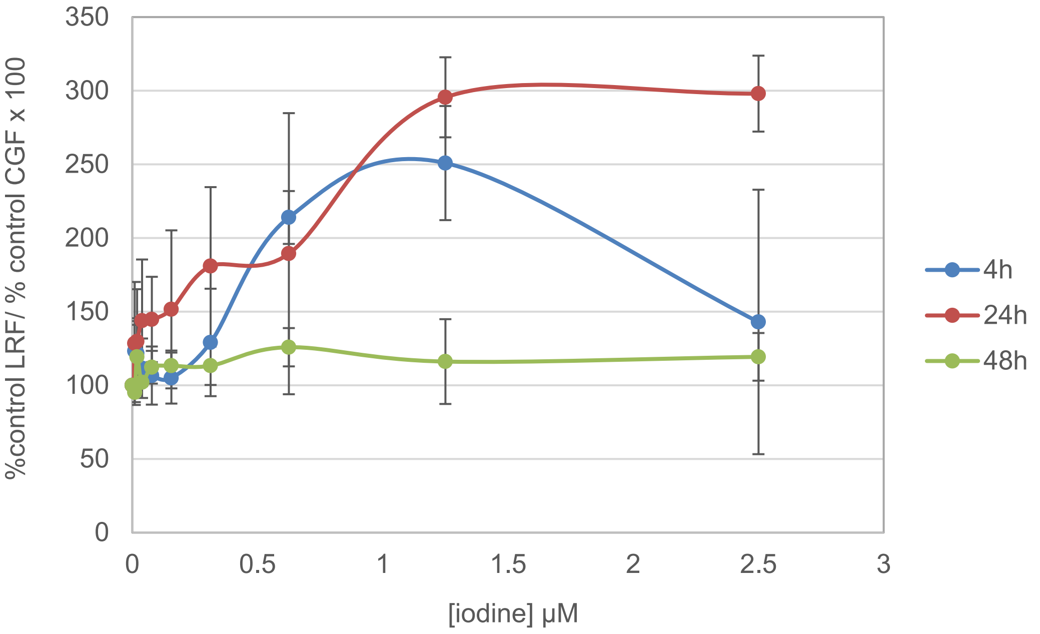

Figure III-3. Autophagy dysfunction assay in LLC-PK1 cells. Displayed is the Lysotracker Red fluorescence normalized to Cell Tracker Green fluorescence (mean + SE). LLC-PK1 cells were treated in six replicate wells of 0-2.5 µM iodine equivalents of NCL388-3, for 4, 24, or 48 hours, then read on a microplate reader (Ex_1=492 nm/Em_1=517 nm, Ex_2=544 nm/Em_2=-590 nm).

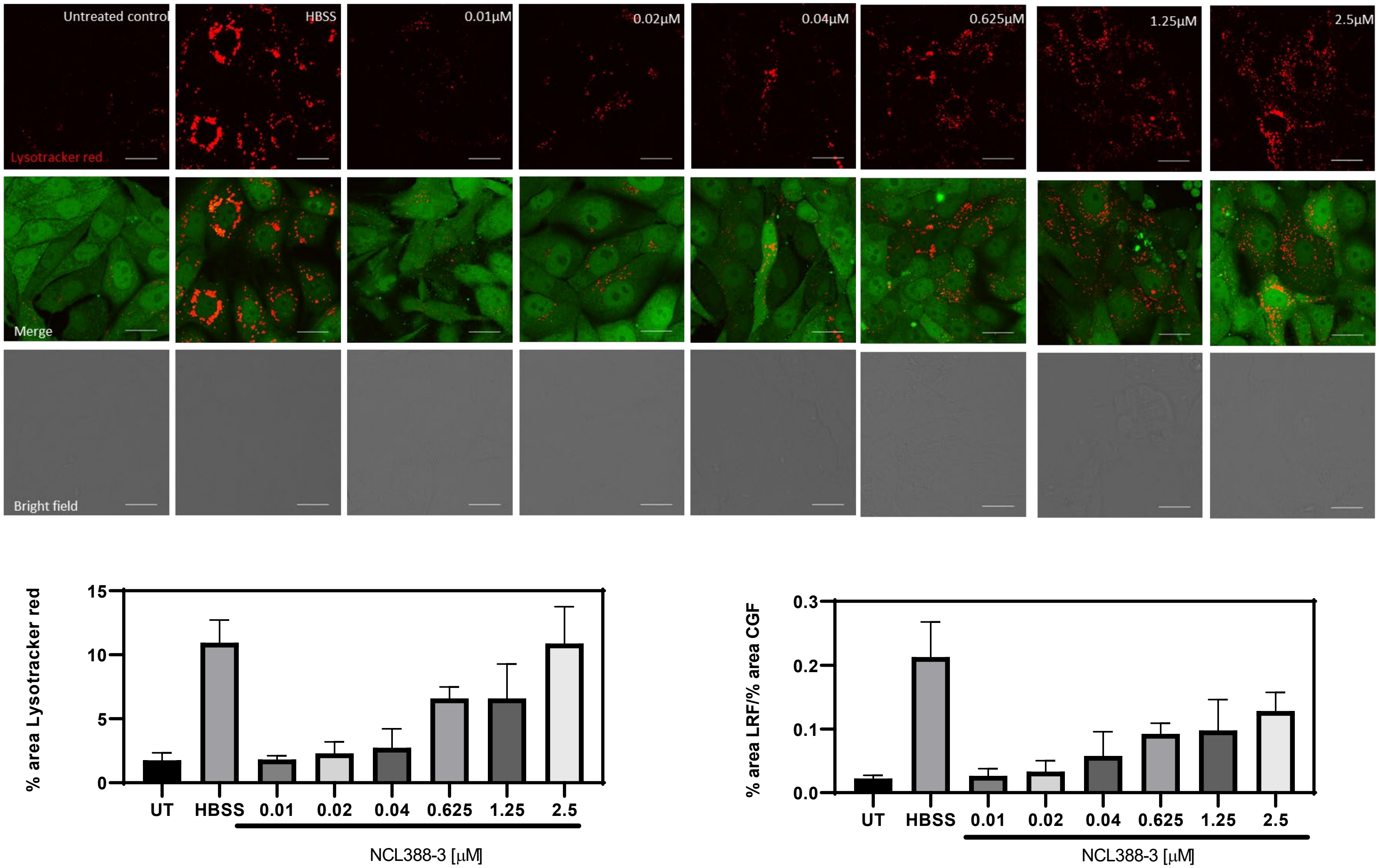

Figure III-4. Confocal Microscopy at 24 hr. LLC-PK1 cells were treated in duplicate in 8-well glass microscopy plates with 0-2.5 µM Iodine equivalents of NCL388-3 for 24 hrs, and then imaged in with a confocal microscope in red and green channels. The bar graphs show the percent area fluorescence determined by Image J. Scale bar=20 µm, UT=untreated control.

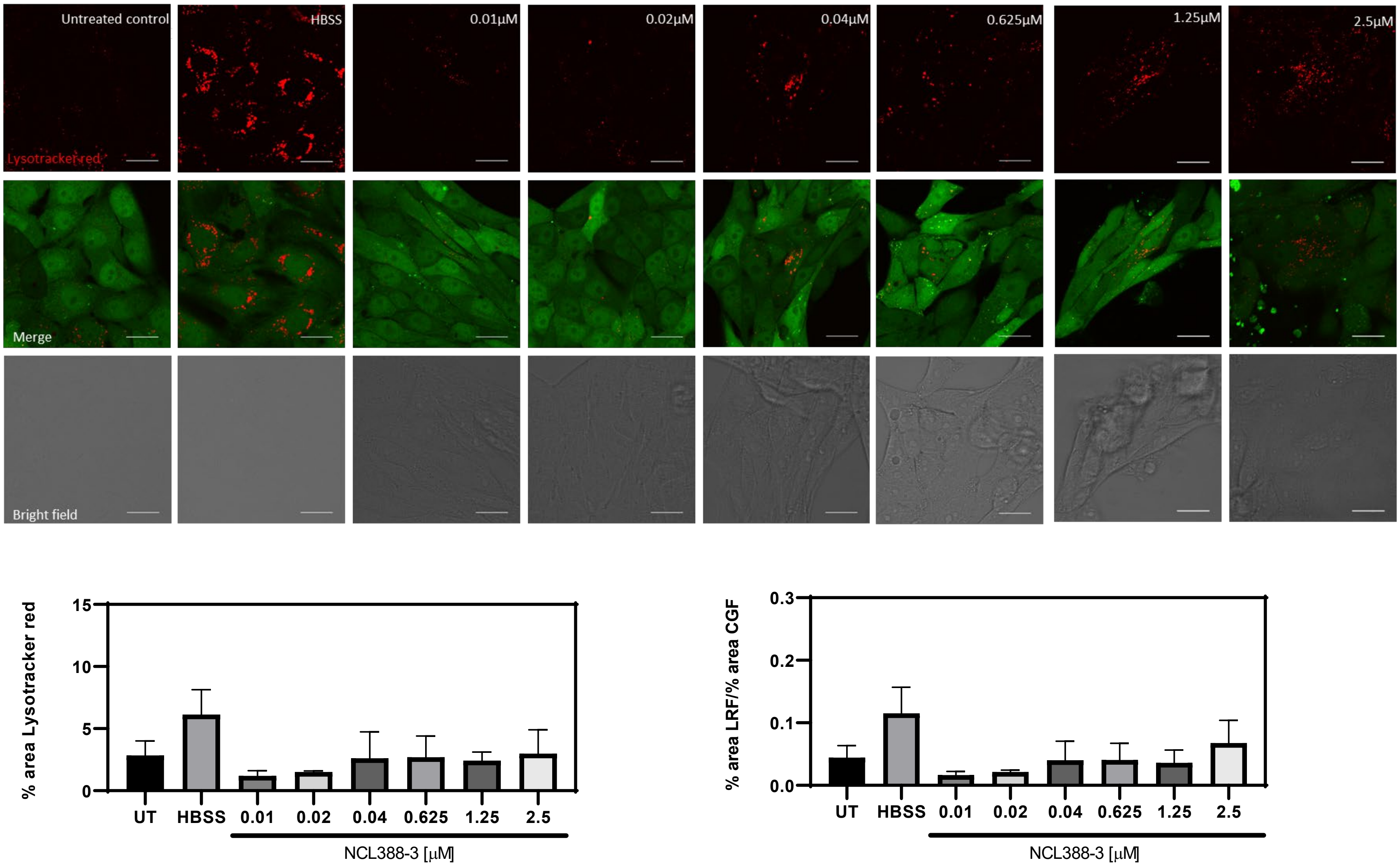

Figure III-5. Confocal Microscopy at 48 hr. LLC-PK1 cells were treated in duplicate in 8-well glass microscopy plates with 0-2.5 μM Iodine equivalents of NCL388-3 for 48 hrs, and then imaged in with a confocal microscope in red and green channels. The bar graphs show the percent area fluorescence determined by Image J. Scale bar=20 μm, UT=untreated control.

IV. In Vitro Immunological Characterization

Section Summary

The purpose of these studies was to analyze the compatibility of NCL388 with blood components. The hematotoxicity of NCL388 was assessed in vitro using hemolysis, platelet aggregation, plasma coagulation and complement activation assays. These analyses were performed using the third batch of NCL388. Batch NCL388-12 was used for the in vitro analysis of cytokine responses in human peripheral blood mononuclear cells (PBMCs). Other batches were not evaluated in these in vitro immunology assays.

Briefly, NCL388-3 was not hemolytic, did not induce complement activation or platelet aggregation, and did not affect collagen-mediated platelet aggregation at the tested concentrations. The formulation did exhibit prolongation of plasma coagulation times in both the thrombin and activated partial thromboplastin time assays. This finding is consistent with the known effects of iohexol on blood coagulation [4]. The formulation also induced chemokines (IL-8, MIP-1α, MIP-1β, and RANTES) in human PBMC cultures in vitro; the observed reverse concentration-response relationship suggests potential toxicity of particles to the cells under in vitro conditions.

The lack of pro-inflammatory cytokines such as (TNFα, IL-1β, IL-6, IL-10) in the NCL388-12-treated cultures is in agreement with an earlier clinical study of ioxehol [5]. Likewise, the induction of chemokine gene expression by iodine-containing compounds has been reported in thyroid follicular cells [4] and PBMCs [7]. The named chemokines are produced by activated myeloid cells such as monocytes and dendritic cells, and by T-cells. The role of these chemokines in the safety of NCL388 is unknown at the moment. Since chemokine function is to recruit immune cells to the site of inflammation, we hypothesize that the observed chemokine response to NCL388 may provide beneficial immunomodulation that could improve the efficacy of cancer immunotherapies (e.g., immune checkpoint inhibitors anti-CTLA4 and anti-PD-1) by promoting lymphocyte infiltration of a tumor. Since IL-8 is also known as one of the pyrogenic markers [8, 9], an elevation of body temperature in sensitive individuals may be observed after administration of NCL388. However, the risk of pyrogenicity does not appear high because more prominent pyrogenic cytokines (TNFα, IL-1β and IL-6) [10-14] were not induced by NCL388. Verification of these hypotheses, understanding of the role of these chemokines in the NCL388 safety profile, along with confirmation of the in vitro-in vivo correlation of these findings require additional investigation.

Selection of Concentrations

For in vitro immunology experiments described in this Section, NCL388-3 was tested at concentrations ranging from 0.28-5 mg/mL. These concentrations were selected based on the in vivo dosing information provided by the sponsor as described below.

As reported by the sponsor, the efficacious dose in a mouse model was 7000 mg/kg. NCL in vitro immunology assays, however, are based on human blood. Therefore, the mouse dose was used to calculate a human equivalent dose (HED) of 569 mg/kg, as shown in Section I [2].

Approximate adult body weights (70 kg) and blood volumes (5.6 L; 8% of body weight) were used to estimate a theoretical plasma concentration of 7.1 mg/mL blood. This calculation assumes that all of the HED injected dose remains in circulation.

HED x avg. adult body weight ÷ avg. adult blood volume = theoretical plasma concentration

569 mg/kg x 70 kg = 39830 mg in 5.6 L of blood = 7.1 mg/mL blood

The in vitro assays ideally test the theoretical plasma concentration (7.1 mg/mL), a 10X concentration, as well as two serial 5-fold dilutions of the theoretical plasma concentration. However, due to limitations in the stock concentration and/or required sample volumes/dilutions for assays, 7.1 mg/mL was not attainable. The highest feasible test concentration was 5 mg/mL. The two serial 5-fold dilutions tested were 1.4 mg/mL and 0.28 mg/mL. A 10X test concentration was not feasible.

We emphasize that the plasma concentrations discussed above are theoretical. They are derived from mathematical calculations and assumptions and may differ from the actual concentrations in the blood under in vivo conditions.

Hemolysis (ITA-1)

Design and Methods

The objective of this experiment was to evaluate the nanoparticle's effect on the integrity of human red blood cells in vitro following NCL protocol ITA-1, Analysis of Hemolytic Properties of Nanoparticles (https://ncl.cancer.gov/resources/assay-cascade-protocols). In brief, freshly drawn human blood anticoagulated with lithium heparin was diluted in PBS to a concentration of 10 mg/mL total blood hemoglobin. The diluted whole blood was then incubated with NCL388-3 at three concentrations (0.28, 1.4, and 5.0 mg/mL of iodine) for 3 hours at 37°C. Following incubation, cell free supernatants were prepared and analyzed for the presence of plasma free hemoglobin by converting hemoglobin and its metabolites into cyanmethemoglobin (CMH) using Drabkin's reagent. CMH was then quantified against a hemoglobin standard by measuring the absorbance of the samples at 540 nm.

Results and Discussion

No hemolysis was detected for NCL388-3 under the tested in vitro assay conditions.

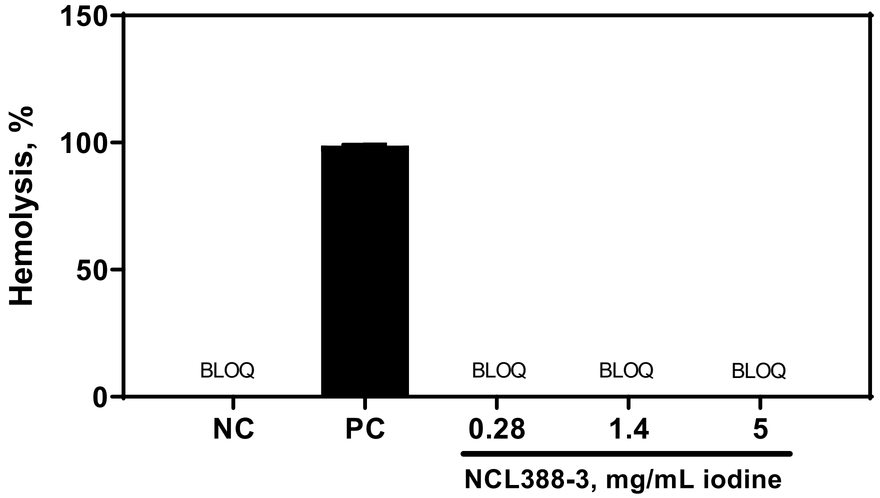

Figure IV-1. In Vitro Hemolysis. NCL388-3 was evaluated for potential particle effects on the integrity of red blood cells. Three independent samples were prepared for each nanoparticle concentration and analyzed in duplicate (%CV < 20). Shown is mean (n = 3) ± SD. Triton X-100 was used as a positive control (PC). PBS was used as the negative control (NC). BLOQ = below lower limit of quantification.

Platelet Aggregation (ITA-2.1)

Design and Methods

The objective of this experiment was to evaluate the effect of NCL388-3 on human platelets *in vitro* following NCL protocol ITA-2.1, Analysis of Platelet Aggregation by Cell Counting (https://ncl.cancer.gov/resources/assay-cascade-protocols). In brief, platelet rich plasma (PRP) and platelet poor plasma (PPP) were prepared from freshly drawn human blood anticoagulated with Sodium Citrate (Na-Cit). Plasma from three donors was pooled. PPP was used as the background control. PRP was incubated with NCL388-3 at three concentrations (0.28, 1.4, and 5.0 mg/mL of iodine), and the number of single platelets was counted using a Beckman Coulter Z2 analyzer. Platelet poor plasma combined with NCL388-3 was used to monitor potential particle aggregation/agglomeration in order to rule out false-negative results. Percent platelet aggregation was calculated by comparing the number of single (unaggregated) platelets in the negative control group with that in the test sample.

Results and Discussion

NCL388-3 did not induce platelet aggregation under the tested in vitro conditions (Figure IV-2A). Furthermore, NCL388-3 did not affect collagen-induced platelet aggregation (Figure IV-2B).

A. Effects on Platelet Aggregation

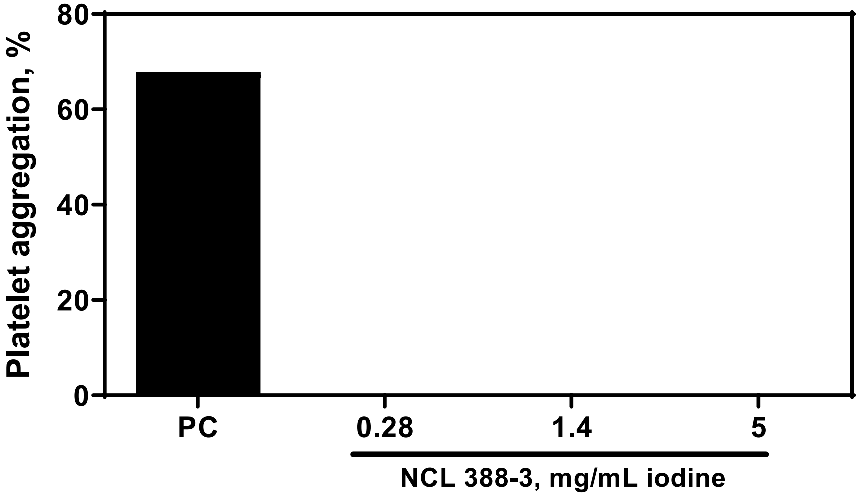

B. Effects on Collagen-Induced Platelet Aggregation

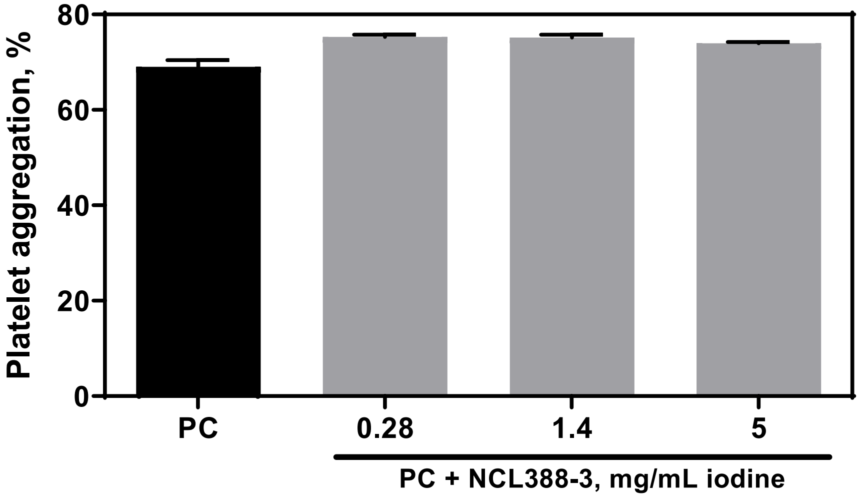

Figure IV-2. In Vitro Platelet Aggregation. NCL388-3 was used to evaluate potential particle effects on platelet aggregation. The sample was tested at concentrations 0.28-5 mg/mL of iodine. Collagen was used as the positive control (PC). Each nanoparticle concentration was tested in triplicate. Shown is the mean ± SD. **B.** Nanoparticles were added to plasma prior to addition of assay's positive control. Platelet aggregation induced by collagen alone (black bar) is compared to platelet aggregation induced by collagen in the plasma containing nanoparticles.

Plasma Coagulation Times (ITA-12)

Design and Methods

The objective of this experiment was to evaluate the effect of nanoparticle treatment on human plasma coagulation *in vitro* following NCL protocol ITA-12, Coagulation Assay (https://ncl.cancer.gov/resources/assay-cascade-protocols). Three plasma coagulation tests, prothrombin time (PT), activated partial thromboplastin time (APTT) and thrombin time (TT), that correspond to the extrinsic, intrinsic and common pathways, respectively, were performed. Freshly drawn human blood from three donors was used to prepare pooled plasma. The pooled plasma was then incubated with NCL388-3 nanoparticles at three concentrations (0.25, 1.4, 5.0 mg/mL iodine) for 30 min at 37°C. Three individual samples were prepared at each concentration and analyzed in duplicate using the STArt4 coagulometer (Diagnostica Stago).

Results and Discussion

NCL388-3 was evaluated for potential effects on the biochemical component of the blood coagulation cascade, i.e., prothrombin time, thrombin time, and activated partial thromboplastin time. NCL388-3 did not affect plasma coagulation in the PT assay. However, NCL388-3 showed prolongation of coagulation time in the APTT assay and concentration-dependent prolongation of coagulation time in the TT assay. This data suggests a heparin-like activity of NCL388-3 and is consistent with known effects of iohexol on blood coagulation (Figure IV-3) [4].

A. Prothrombin Time

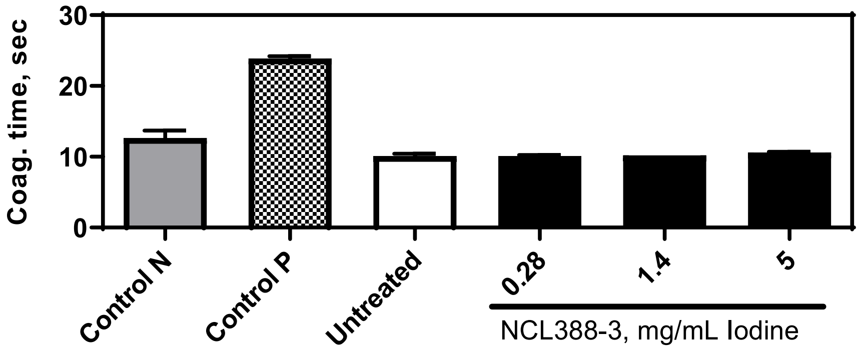

B. Thrombin Time

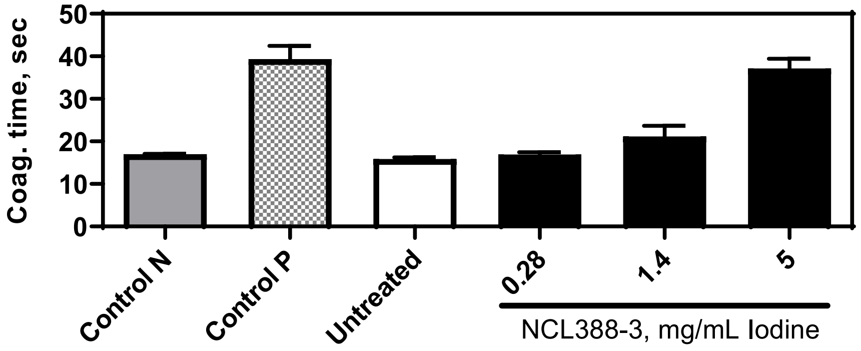

C. Activated Partial Thromboplastin Time

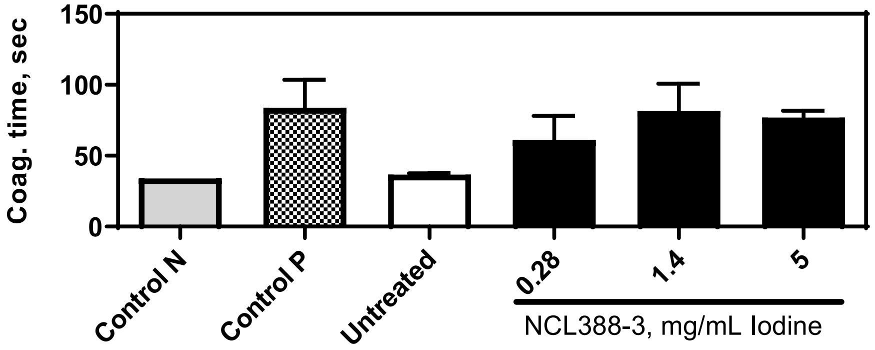

Figure IV-3. In Vitro Plasma Coagulation Times. NCL388-3 was used to evaluate potential particle effects on the biochemical component of the blood coagulation cascade (prothrombin time, thrombin time, and activated partial thromboplastin time). For each nanoparticle concentration, three independent samples were prepared and analyzed in duplicate (%CV < 5). Each bar represents the mean (N=3) ± SD. Normal plasma standard (Control N) and abnormal plasma standard (Control P) were used for instrument controls. Plasma pooled from at least three donors was either untreated (Untreated) or treated with nanoparticles at shown concentrations.

Complement Activation (ITA-5.2)

Design and Methods

The objective of this experiment was to evaluate the effect of NCL388-3 on the human complement system. The procedure described in ITA-5.2, Analysis of Complement Activation by EIA (https://ncl.cancer.gov/resources/assay-cascade-protocols) was followed. Briefly, plasma was prepared from freshly drawn human blood anticoagulated with K_2EDTA. Plasma from three donors was pooled and incubated with test samples (0.28, 1.4, and 5.0 mg/ml iodine) and veronal buffer for 30 min at 37°C. Following incubation, the samples were analyzed for the presence of the iC3b component of complement using a commercial enzyme immunoassay kit. Doxil, which is known to cause complement-mediated toxicity in patients, was included as an additional control and was tested at 0.66 mg/mL.

Results and Discussion

NCL388-3 did not activate complement *in vitro* at any of the tested concentrations (Figure IV-4).

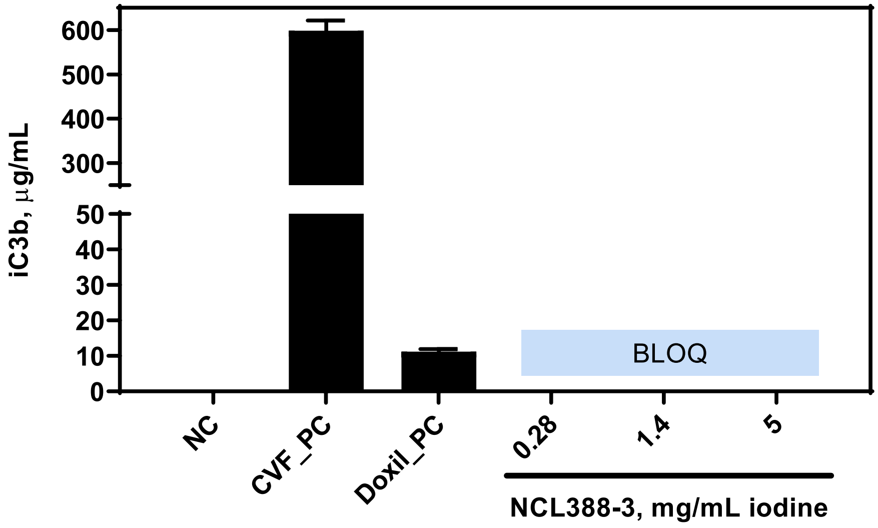

Figure IV-4. In Vitro Complement Activation. NCL388-3 was tested at concentrations ranging 0.28-5 mg/mL iodine. PBS was used as the negative control (NC). Cobra venom factor (CVF) was used as the positive control. Three independent samples were prepared for each concentration and analyzed in duplicate (%CV<20). Shown is the mean response (N=3) ± SD. BLOQ = below limit of quantification.

Cytokines (ITA-10)

Design and Methods

The objective of this experiment was to evaluate the effect of nanoparticles on induction of pro-inflammatory cytokines in whole blood cultures of healthy donor volunteers. Experiments were performed according to NCL protocol ITA-10 (https://ncl.cancer.gov/resources/assay-cascade-protocols). Briefly, whole blood anticoagulated with lithium heparin was used to purify PBMC. PBMCs were incubated with controls and nanoparticles for 24 h. At the end of incubation, the samples were centrifuged for 5 min at 18,000x*g* and supernatants were analyzed for the presence of presence of cytokines and interferons using 15-plex multiplex ELISA kits (Quansys, Logan, UT).

Results and Discussion

The multiplex panel used in this study included the following cytokines: IFNγ, IL-1α, IL1β, IL-6, IL-8, IL-10, IL-12, IL-21, IP-10, MCP-1, MCP-2, MIP-1α, MIP-1β, RANTES and TNFα). No elevation in cytokine levels was observed for IFNγ, IL-1α, IL1β, IL-6, IL-10, IL-12, IL-21, IP-10, MCP-1, MCP-2, and TNFα (Figure IV-5). This data is in agreement with an earlier clinical study demonstrating the lack of proinflammatory cytokine response after ioxehol injection [5].

NCL388-12 induced chemokines IL-8, MIP-1α, MIP-1β, and RANTES in a reverse concentration-response manner (Figure IV-5 and IV-6). The observed reverse concentration-response relationship suggests potential toxicity of particles to the cells under in vitro conditions. The induction of chemokine gene expression by iodine-containing compounds has been reported in thyroid follicular cells [6] and PBMCs [7]. The named chemokines are produced by activated myeloid cells such as monocytes and dendritic cells, and by T-cells. The role of these chemokines in the safety of NCL388 is unknown at the moment. Since chemokines' function is to recruit immune cells to the site of inflammation, we hypothesize that the observed chemokine response to NCL388 may provide beneficial immunomodulation that could improve the efficacy of cancer immunotherapies (e.g., immune checkpoint inhibitors anti-CTLA4 and anti-PD-1) by promoting lymphocyte infiltration of a tumor. Since IL-8 is also known as a pyrogenic marker [8, 9], an elevation of body temperature in sensitive individuals may be observed after the administration of NCL388. However, the risk of pyrogenicity does not appear high because more prominent pyrogenic cytokines (TNFα, IL-1β and IL-6) [10-14] were not induced by NCL388. Verification of these hypotheses, understanding of the role of these chemokines in the NCL388 safety profile, along with confirmation of the in vitro-in vivo correlation of these findings, require additional investigation.

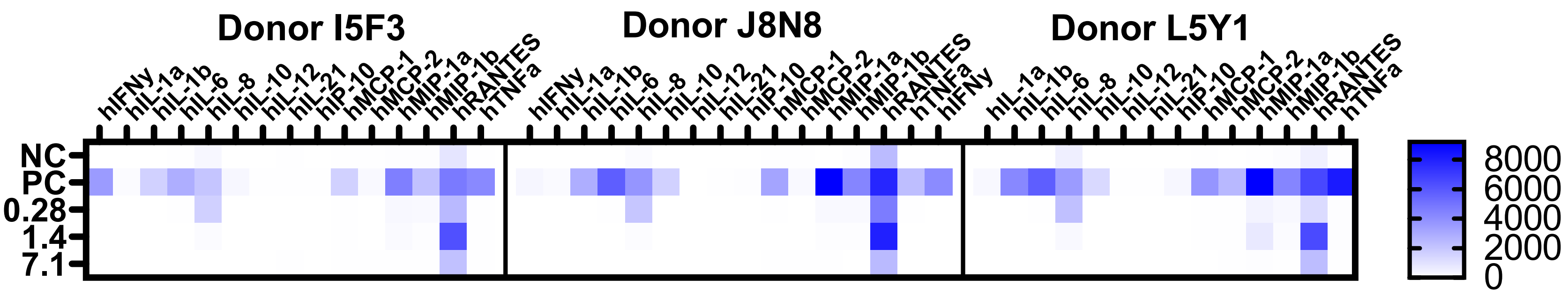

Figure IV-5. Induction of cytokines by NCL388-12 in PBMC cultures in vitro. Human PBMC isolated from fresh blood of healthy donor volunteers (I5F3, J8N8 and L5Y1) were exposed to various concentrations of NCL388-12 (0.28, 1.4 and 7.1 mg/mL of iodine), negative control (NC) or positive control (PC) for 24 hours. Following incubation, the supernatants were analyzed for the presence of cytokines. PBS was used as the NC; PHA-M and LPS were used as the PC. Each square on the heatmap represents a mean response (N=3). The scale on the right side of the heatmap shows the concentration of cytokines in pg/mL. The darker the color, the higher the level of cytokines. Note, low levels of some cytokines produced by less abundant cell populations are not easy to visualize on the heatmap in the presence of other cytokines that are present at the very high levels. Bar graphs of individual cytokines induced by NCL388-12 are shown in Figure IV-6. Cytokines which were not induced by NCL388-12 are not included in Figure IV-6.

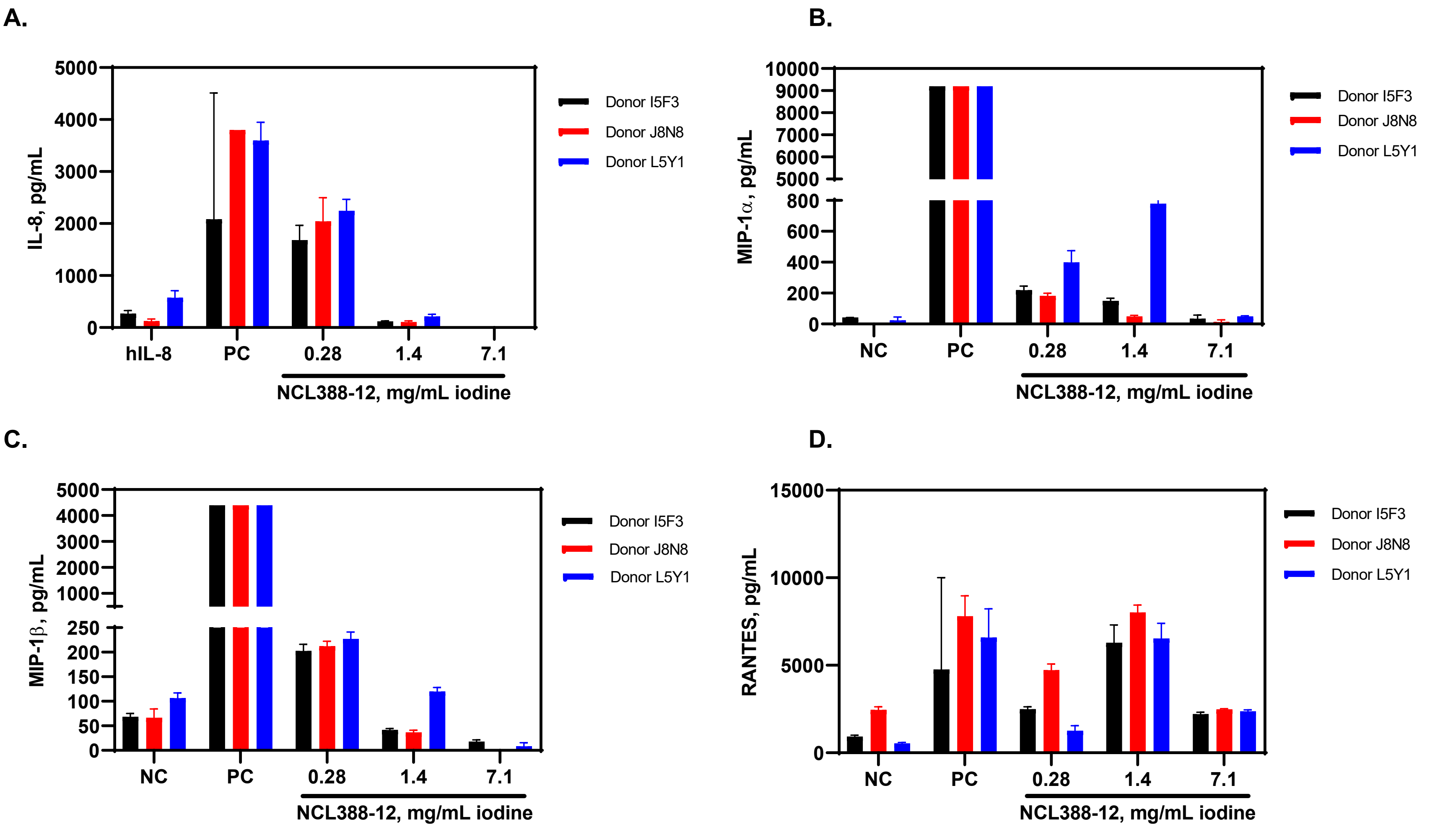

Figure IV-6. Induction of chemokine response by NCL388-12. Human PBMC isolated from fresh blood of healthy donor volunteers (I5F3, J8N8 and L5Y1) were exposed to various concentrations of NCL388-12 (0.28, 1.4 and 7.1 mg/mL of iodine), negative control (NC) or positive control (PC) for 24 hours. Following incubation, the supernatants were analyzed for the presence of cytokines. PBS was used as the NC; PHA-M and LPS were used as the PC. Each bar is the mean response and standard deviation (N=3). Only cytokines, which levels were at least 2-fold above the baseline for at least one concentration of NCL388-12 are shown in this figure and includes chemokines IL-8 (A), MIP-1α (B), MIP-1β (C), and RANTES (D). Cytokines which were not induced by NCL388-12 are not shown in this figure. The same data used in Figure IV-5 were used to create this figure.

V. Multidose In Vivo Toxicology Study in CD-1 Mice (ADME Tox 193)

Section Summary

The purpose of this study was to evaluate the multidose (qdx4) toxicity of NCL388 in CD-1 mice at two different dose levels, 858 and 1,715 mg I/kg total dose. In addition to the main study group that was terminated on study day 5, one day post the final dose, a recovery arm was also included that was terminated 14 days following the last dose, in order to evaluate recovery from potential toxicities observed in the main study group. The inclusion of a recovery arm is standard practice in toxicology study design.

The dose levels used in this study were based on discussions with Nanoprobes and their prior experience with the drug. Study parameters included daily observation and body weights, and necropsy at study termination. Necropsy included organ weights, gross organ description, hematology, clinical chemistry, gross pathology, and comprehensive histopathology of tissues. Tissues were selected following histopathology analysis for targeted immunohistochemistry and electron microscopy evaluation of findings. Urine and feces were also collected during the study period for analysis of iodine excretion using ICP-MS methods. One animal in the low-dose group of the main study arm died post-injection following the second treatment. Due to a lack of observable effects on other animals in the treatment groups, this instance was likely not related to treatment. Similar body weight gain was observed for low dose treatment and control groups. Statistically significant weight gain was noted in the high dose treatment groups on study days 4 and 5 in the high dose main group. This significant weight increase is consistent among animals in the group and likely related to treatment. Although there was a similar trend toward increased body weight in the high dose recovery arm, this did not reach statistical significance.

Statistically significant increases in leukocytes and neutrophils in treated animals suggest a mild leukocytosis with neutrophilia, with recovery groups being more severely affected than main study groups. The remaining statistically significant hematology and clinical chemistry findings were not considered biologically significant because they were modest and not dose-dependent. BUN, glucose and MPV were above historical averages, and platelets, RDW, and lymphocyte values were below historical averages for all groups, including the saline control (https://www.criver.com/sites/default/files/resources/CD-1IGSMouseModelInformationSheet.pdf).

Statistically significant, dose-dependent increases in liver and spleen weights were observed in both the main and recovery treatment groups in comparison to saline control; these increases are considered treatment-related. Statistically significant differences in brain weight and kidney weight were variable in presentation and likely not treatment related.

Upon necropsy, enlarged liver and spleen were observed most frequently in high-dose groups, consistent with the significantly increased organ weights described above. Other gross lesions identified were inconsistent and typical background of this model/strain, and not considered treatment related.

Treated mice in both the main and recovery groups developed similar lesions characterized by multisystemic histiocytosis, with recovery groups being more severely affected. The histiocytic infiltrate ranges from mild to severe, was present in all organs, and was dose-dependent. The presence of this histiocytosis was statistically significant across evaluated organs when compared to respective controls. *NB*: The histiocytosis noted is most likely the result of iodine polymer accumulation in tissue macrophage, as has been shown for other biopersistent

polymers such as PEG with PEGylated therapeutics [15], and in this study was not associated with other histopathological changes, nor associated with alterations in hematological or clinical chemistry parameters indicative of toxicity. The remaining histological lesions were interpreted as background changes commonly seen in this animal model/strain.

Anti-F4/80 immunohistochemistry was performed on liver sections from saline control, high dose main, and high dose recovery groups to confirm the diagnosis of histiocytosis. Anti-F4/80+ cells, corresponding with cells classified as histiocytes, were significantly increased in high dose groups compared to the saline control, and in the recovery group compared to the main group in high-dose animals. Electron microscopy examination of liver from treated mice in both the low and high dose recovery groups showed the presence of intracytoplasmic vacuoles of varying size and density within Kupffer and sinusoidal cells consistent with uptake of the iodine polymer.

Design and Methods

Test Article Preparation
The multidose toxicity study utilized NCL388-12, which was a pooled lot comprised of batches NCL388-4, NCL388-5, NCL388-6, NCL388-9, NCL388-10, and NCL388-11. The NCL388-12 concentration was 68.6 mg iodine/mL (measured by ICP-MS at NCL). The saline control (saline for injection) was dosed at 25 mL/kg. The high dose level was administered at stock concentration; stock NCL388-12 was diluted in PBS for the low dose level groups.

Experimental Methods
The animal model utilized was 7-week-old male CD-1 mice, eight animals per treatment group (Charles River Laboratories, Inc., Wilmington, MA). The study included two dose levels of NCL388, as well as a saline control group, administered i.v. by tail vein every day for four consecutive doses (qd x 4) (Table V-1), and consisted of both a main study arm that was terminated one day following the last dose, and a recovery arm that was terminated 14 days following the last dose. The inclusion of a recovery arm is standard practice in toxicology study design, in order to evaluate recovery from potential toxicities observed in the main study group. All treatments were administered at 25 mL/kg body weight. This study also included 8-hr urine and feces collections from all animals 24 hours after receiving the final dose, as well as from recovery animals on day 18 (14 days after receiving the final dose). Urine and feces collections were pooled by group, with 2 samples per group per timepoint, and analyzed using ICP-MS methods for iodine quantification. Animals were randomized to treatment groups by weight using StudyLog software (StudyLog Systems Inc., CA). For the randomization report, see Appendix C; for further study protocol details see Appendix D.

Animals were monitored daily for mortality and signs of pharmacologic or toxicologic effects. Body weights were measured every alternate weekday (M, W, F). Moribund animals (sustained >20% loss in body weight) were euthanized. At study termination, 1 day following the last dose for the main study (study day 5) and 14 days after the last dose for the recovery study group (study day 18), animals were euthanized by CO_2 asphyxiation. Necropsy included organ weights and gross organ description. Blood was removed by thoracic cutdown for hematology (see parameters list below) and clinical chemistry (see parameters list below). All tissues predetermined (see tissue list below) and those presenting with gross lesions were fixed, embedded, and underwent further histopathological evaluation and grading by board certified veterinary pathologists. Anti-F4/80 immunohistochemistry for macrophage identification was performed on a subset of animal tissues selected by the pathologist, to include both control and treated subjects. Several liver samples were also selected by the pathologist and submitted for electron microscopy (EM) analysis.

Table V-1. Toxicity Study Dose Groups. There were two arms to this study, a main study arm that was terminated one day following the last dose, and a recovery study arm that was terminated 14 days following the last dose.

Treatment Group	NCL388-12 (mg I/kg) qdx4	Mice (n)	Dose Volume (mL/kg)
Saline Vehicle	*not applicable*	8	25
NCL388-12	214<br>(855 total dose)	8	25
NCL388-12	429<br>(1715 total dose)	8	25

<u>Tissues Evaluated by Histopathology</u>
Tissues evaluated by histopathology include adrenal, brain, cecum, colon, duodenum, esophagus, eye, femur, Harderian gland, heart, ileum, jejunum, kidney, liver, lung, mammary gland, mandibular lymph node, mesenteric lymph node, nasal sections, ovary, pancreas, parathyroid, pituitary, rectum, salivary gland, skin/subcutis, spinal cord, spleen, stomach, tail, thymus, thyroid, tongue, trachea, urinary bladder, uterus, vertebra, and any additional tissue with gross findings at necropsy.

<u>Hematology Parameters</u>
Hematology parameters analyzed include differential leukocyte count (BA, EO, LY, MO, and NE), erythrocyte count (RBC), hematocrit (HCT), hemoglobin (Hb), mean corpuscular hemoglobin (MCH), mean corpuscular hemoglobin concentration (MCHC), mean corpuscular volume (MCV), mean platelet volume (MPV), platelet count (PLT), red blood cell distribution width (RDW), and total leukocyte count (WBC).

<u>Clinical Chemistry Parameters</u>
Clinical chemistry parameters analyzed include alanine aminotransferase (ALT), albumin (A), albumin/globulin ratio (A/G), alkaline phosphatase (ALP), amylase, blood urea nitrogen (BUN), creatinine, calcium, globulin (G), glucose, phosphate, potassium, sodium, total bilirubin, and total protein.

<u>Husbandry</u>
Animals were acclimated to the study environment for one week prior to study initiation. Animal rooms were kept at 30-70% relative humidity, 70-75˚F with 12 hr light/dark cycles. Mice were housed by treatment group in metabolic cages for urine and feces collection, with four animals/cage (2 cages/treatment group). Animals were allowed *ad libitum* access to Purina 5L79 rodent diet and chlorinated tap water.

The Frederick National Laboratory for Cancer Research is accredited by AAALAC International and follows the Public Health Service *Policy for the Care and Use of Laboratory Animals* (Health Research Extension Act of 1985, Public Law 99-158, 1986). Animal care was provided in accordance with the procedures outlined in the *Guide for Care and Use of Laboratory Animals* (National Research Council, 1996; National Academy Press, Washington, D.C.). All animal protocols were approved by the FNLCR institutional Animal Care and Use Committee. The experiments outlined herein are scientifically justified and do not represent an unnecessary duplication of previous work by the sponsor.

<u>Statistical Methods</u>
Statistical analyses were conducted using the software TIBCO Statistica, version 13.6 (TIBCO Software Inc. (2018). Statistica (data analysis software system), version 13. http://tibco.com.) Statistical differences for parametric data were determined by ANOVA, with post-hoc comparisons by Dunnett's T Test or Tukey's multiple range test. Nonparametric data was analyzed by the Kruskal-Wallis ANOVA with multiple comparisons test.

In-Life Results

Clinical Observations

No abnormal clinical observations were noted for any of the animals in any of the treatment groups. One animal in the low-dose group of the main arm died post-injection following the second treatment. Due to a lack of observable effects in other animals in the treatment groups, this instance is likely not related to treatment.

Absolute Body Weight

Group means and statistics resulting from the initial body weight randomization can be found in Appendix C. Similar body weight gain was observed for low dose treatment and control groups. Statistically significant weight gain was noted in high dose treatment groups on study days 4 and 5, as well as upon termination for the high dose main group (Table V-2a). This significant weight increase is consistent among the group and likely related to treatment. No statistically significant differences in body weight were observed in the recovery arm (Table V-2b). Individual absolute body weight values can be found in Appendix B.

(Significant differences between treatment and saline control groups are highlighted in yellow on each Table, $p<0.05$ by ANOVA with Dunnett's T test.)

Table V-2a. Main Study Body Weight. Body weight for each study day is displayed by treatment group. Study day 1 is the first day of injections. Data is presented as the mean ± SD (n = 8). Significant differences between treatment and saline control groups are highlighted in yellow, $p<0.05$ by ANOVA with Dunnett's T test.

Day	Saline			NCL388-12 855 mg I/kg			NCL388-12 1715 mg I/kg		
1	35.19	+	2.45	34.56	+	2.81	34.63	+	1.81
3	34.86	+	2.26	34.76	+	1.97	35.14	+	1.70
4	34.99	+	2.33	35.57	+	2.05	37.98	+	1.67*
5	34.93	+	2.32	35.43	+	2.06	37.81	+	1.65*

Table V-2b. Recovery Study Body Weight. Body weight for each study day is displayed by treatment group. Data is presented as the mean ± SD (n = 8).

Day	Saline			NCL388-12 855 mg I/kg			NCL388-12 1715 mg I/kg		
1	35.48	+	1.65	34.71	+	1.77	34.40	+	2.88
3	35.30	+	1.59	35.15	+	2.33	36.19	+	3.74
4	35.39	+	1.41	35.86	+	2.34	36.89	+	3.60
5	35.28	+	1.49	35.73	+	2.34	36.60	+	3.54
10	36.70	+	1.26	37.58	+	2.05	37.61	+	4.92
12	37.11	+	1.31	38.14	+	2.26	39.06	+	4.27
15	37.33	+	1.05	38.49	+	2.63	39.75	+	4.42
17	37.74	+	1.30	39.24	+	2.54	40.29	+	4.35
18	37.54	+	1.44	38.96	+	2.46	39.99	+	4.38

Necropsy Results

Clinical Chemistry Parameters
Statistically significant findings, including differences in sodium, glucose, amylase, and alkaline phosphatase, were inconsistent in presentation in the main and recovery study groups and do not appear to be treatment related (Table V-3a and V-3b). Albumin and total protein decreases in the main study group are dose-dependent and appear treatment related, but are modest and within historical ranges (https://www.criver.com/sites/default/files/resources/CD-1IGSMouseModelInformationSheet.pdf) and unlikely to have biological significance. BUN and glucose values for all groups were above historical averages for all groups, including the saline control. Individual clinical chemistry values can be found in Appendix B.

Table V-3a. Main Group Clinical Chemistry Parameters. Clinical chemistry parameters are displayed by treatment group. Data is presented as the mean + SD.

Treatment		Saline			NCL388-12 855 mg I/kg			NCL388-12 1715 mg I/kg		
Number of Animals		8			7			8		
**Electrolyte balance**										
Calcium	mg/dL	12.74	+	0.72	12.23	+	0.35	11.75	+	0.92
Phosphate	mg/dL	12.43	+	0.54 *	12.44	+	1.54	11.49	+	1.71
Potassium	mmol/L	10.48	+	1.07	10.06	+	0.91	9.30	+	1.43
Sodium	mmol/L	156.38	+	0.74	154.43	+	2.82	151.00	+	2.39*
**Carbohydrate Metabolism**										
Glucose	mg/dL	352.88	+	45.41	343.00	+	43.83	275.88	+	44.51*
**Pancreatic Function**										
Amylase	u/L	1475.25	+	436.66	1029.71	+	80.90*	1029.00	+	162.92
**Liver Function: A) Hepatobiliary**										
Total Bilirubin	mg/dL	0.28	+	0.05	0.30	+	0.00	0.30	+	0.00
**Liver Function: B) Hepatocellular**										
Alanine Aminotransferase	u/L	81.00	+	102.92	42.43	+	23.64	104.14	+	117.23
**Kidney Function**										
Creatinine	mg/dL	0.25	+	0.18	0.21	+	0.04	0.23	+	0.05
Urea Nitrogen	mg/dL	26.25	+	4.33	22.14	+	2.19	24.00	+	2.93
**Others**										
Albumin (A)	g/dL	4.28	+	0.34	3.33	+	0.30*	3.18	+	0.14*
Alkaline Phosphatase	u/L	83.00	+	14.76	58.14	+	5.49*	59.20	+	11.71
Globulin (G)	g/dL	1.91	+	0.19	1.93	+	0.28	1.84	+	0.21
A/G Ratio		2.26	+	0.33	1.76	+	0.29*	1.75	+	0.22*
Total Protein	g/dL	6.20	+	0.21	5.24	+	0.35*	5.01	+	0.23*

*Significantly different from saline control, p<0.05 (ANOVA with Dunnett's T test).

Table V-3b. Recovery Group Clinical Chemistry Parameters. Clinical chemistry parameters are displayed by treatment group. Data is presented as the mean + SD.

Treatment		Saline			NCL388-12 855 mg I/kg			NCL388-12 1715 mg I/kg		
Number of Animals		8			8			8		
**Electrolyte Balance**										
Calcium	mg/dL	12.00	+	0.91	12.33	+	0.32	12.34	+	0.52
Phosphate	mg/dL	10.93	+	2.06	12.64	+	1.67	12.99	+	1.97
Potassium	mmol/L	8.99	+	1.08	9.84	+	1.20	9.71	+	1.25
Sodium	mmol/L	155.63	+	1.51	155.75	+	1.91	155.13	+	1.46
**Carbohydrate Metabolism**										
Glucose	mg/dL	327.63	+	52.13	347.13	+	62.62	298.38	+	68.75
**Pancreatic Function**										
Amylase	u/L	1274.88	+	96.32	1253.25	+	132.11	1353.50	+	133.75
**Liver Function: A) Hepatobiliary**										
Total Bilirubin	mg/dL	0.26	+	0.05	0.29	+	0.06	0.29	+	0.06
**Liver Function: B) Hepatocellular**										
Alanine Aminotransferase	u/L	50.63	+	19.27	286.75	+	651.07	90.38	+	47.96
**Kidney Function**										
Creatinine	mg/dL	0.30	+	0.12	0.31	+	0.08	0.28	+	0.12
Urea Nitrogen	mg/dL	26.00	+	3.42	24.50	+	2.33	25.38	+	3.07
**Others**										
Albumin (A)	g/dL	3.98	+	0.38	3.76	+	0.19	3.79	+	0.25
Alkaline Phosphatase	u/L	78.88	+	11.15	58.63	+	10.76*	56.38	+	14.27*
Globulin (G)	g/dL	2.06	+	0.24	2.24	+	0.29	2.36	+	0.37
A/G Ratio		1.95	+	0.26	1.71	+	0.25	1.65	+	0.38
Total Protein	g/dL	5.99	+	0.42	6.00	+	0.32	6.15	+	0.28

*Significantly different from saline control, $p<0.05$ (ANOVA with Dunnett's T test).

Hematology Parameters

Statistically significant findings, including differences in MCHC, RBC, hemoglobin, hematocrit, MCH, RDW, and MPV, were inconsistently observed in main and recovery treated animals in comparison to saline, and unlikely to be treatment related (Table V-4a and V-4b). Increases in neutrophils and overall leukocytes suggest a mild leukocytosis with neutrophilia across treated groups, with recovery groups being more severely affected than main groups. A consistent decrease in monocytes in treated animals in the main group is also noted. Several parameters are outside of reported historical ranges in the high dose treated animals: RBC, hemoglobin, and hematocrit are slightly lower, with MPV being slightly higher than historical data. Platelets, RDW and lymphocyte values for all treatment groups, including saline control, were lower than historical averages (https://www.criver.com/sites/default/files/resources/CD-1IGSMouseModelInformationSheet.pdf). Individual hematology values can be found in Appendix B.

Table V-4a. Hematology Parameters. Hematology parameters are displayed by treatment group for main study. Data is presented as the mean ± SD.

Treatment groups		Saline - Main			NCL388 855 mg I/kg - Main			NCL388 1715 mg I/kg - Main		
Number of Animals		8			7			8		
**Leukocytes**										
WBC	K/uL	5.12	+	1.83	6.85	+	2.46	6.50	+	2.74
NE	K/uL	1.96	+	0.75	4.48	+	2.26*	3.64	+	1.73
LY	K/uL	1.99	+	0.90	1.78	+	0.39	2.16	+	1.48
MO	K/uL	0.79	+	0.32	0.44	+	0.14*	0.51	+	0.20*
EO	K/uL	0.33	+	0.36	0.10	+	0.08	0.12	+	0.14
BA	K/uL	0.05	+	0.03	0.06	+	0.05	0.06	+	0.07
NE	%	37.69	+	7.80	62.39	+	11.46*	56.54	+	12.74*
LY	%	39.17	+	12.41	28.39	+	9.13	32.73	+	11.39
MO	%	15.89	+	4.65	6.73	+	2.03*	8.28	+	1.83*
EO	%	6.35	+	6.42	1.64	+	1.23	1.71	+	1.29
BA	%	0.91	+	0.59	0.85	+	0.73	0.74	+	0.70
**Erythrocytes**										
RBC	M/uL	8.86	+	1.57	8.30	+	0.54	7.14	+	1.42*
Hb	g/dL	13.50	+	2.66	12.99	+	1.22	10.94	+	1.59*
HCT	%	45.11	+	8.65	40.63	+	6.95	34.35	+	5.55*
MCV	fL	51.00	+	3.54	48.83	+	6.27	48.56	+	2.94
MCH	pg	15.20	+	0.67	15.63	+	0.75	15.54	+	1.28
MCHC	g/dL	29.90	+	1.97	32.27	+	2.48*	31.91	+	0.78
RDW	%	11.88	+	0.29	12.00	+	0.36	13.51	+	3.29
**Thrombocytes**										
PLT	K/mL	744.63	+	304.76	610.14	+	222.35	521.00	+	228.45
MPV	fL	6.71	+	0.23	6.96	+	0.20	7.34	+	0.17*

*Significantly different from saline control, $p<0.05$ (ANOVA with Dunnett's T test).

Table V-4b. Hematology Parameters. Hematology parameters are displayed by treatment group for recovery study. Data is presented as the mean ± SD (n = 8).

Treatment groups		Saline - Recovery			NCL388, 855 mg I/kg - Recovery			NCL388, 1715 mg I/kg - Recovery		
Number of Animals		8			8			8		
**Leukocytes**										
WBC	K/uL	7.65	±	2.30	11.69	±	2.65*	9.37	±	1.72
NE	K/uL	2.77	±	0.81	5.87	±	1.64*	5.43	±	0.94*
LY	K/uL	3.37	±	1.50	4.42	±	1.36	2.45	±	0.83
MO	K/uL	0.86	±	0.26	0.84	±	0.28	0.63	±	0.25
EO	K/uL	0.55	±	0.29	0.40	±	0.22	0.55	±	0.46
BA	K/uL	0.10	±	0.08	0.16	±	0.11	0.32	±	0.33
NE	%	36.32	±	5.06	50.12	±	6.47*	58.32	±	6.46*
LY	%	43.02	±	8.20	37.75	±	7.00	25.92	±	5.50*
MO	%	11.48	±	2.20	7.05	±	1.02*	6.74	±	2.29*
EO	%	8.01	±	5.21	3.60	±	2.50	5.76	±	4.50
BA	%	1.18	±	0.76	1.49	±	1.32	3.26	±	3.24
**Erythrocytes**										
RBC	M/uL	8.91	±	0.56	8.40	±	0.30*	8.29	±	0.32*
Hb	g/dL	14.23	±	1.08	12.70	±	0.83*	12.15	±	0.94*
HCT	%	43.19	±	6.31	40.99	±	5.68	37.25	±	4.08
MCV	fL	48.33	±	4.27	48.71	±	6.12	44.96	±	4.76
MCH	pg	15.98	±	0.60	15.13	±	0.87	14.65	±	0.88*
MCHC	g/dL	33.20	±	2.15	31.28	±	2.61	32.73	±	1.68
RDW	%	12.58	±	0.74	13.41	±	1.32	15.38	±	2.16*
**Thrombocytes**										
PLT	K/mL	855.00	±	198.24	918.75	±	107.70	1083.88	±	248.50
MPV	fL	6.63	±	0.15	6.73	±	0.20	7.03	±	0.16*

*Significantly different from saline control, $p<0.05$ (ANOVA with Dunnett's T test).

Organ Weights

Statistically significant findings, including differences in brain weight (absolute and as a percent of body weight) and kidney weight (as percent of brain weight), were variable in presentation and likely not treatment related (Table V-5a and V-5b). Consistent, statistically significant changes in liver and spleen weight values were observed in both main and recovery groups in comparison to saline control groups. In the main study arm, liver and spleen weights were elevated, but not significant, in the low-dose group compared to the control group; liver weights in the high dose group were significantly elevated, suggesting this increase is treatment related and dose-dependent. In the recovery arm, both treatment groups displayed statistically significant increases in liver weight. The high dose recovery group also displayed a statistically significant increase in spleen weight compared to the saline control group. Individual organ weight values can be found in Appendix B.

Table V-5a. Main Study Organ Weights. The organ weights are displayed as absolute values, % body weight, and % brain weight by treatment group. Data is presented as the mean ± SD.

Treatment		Saline			NCL388-12 855 mg I/kg			NCL388-12 1715 mg I/kg		
Number of Animals		8			7*			8		
**Body Weight**										
Absolute Weight	Gram	34.27	±	2.71	35.55	±	1.63	37.72	±	1.96*
**Brain**										
Absolute Weight	Gram	0.48	±	0.03	0.49	±	0.02	0.51	±	0.01*
Per body weight	%	1.40	±	0.09	1.37	±	0.06	1.34	±	0.07
**Heart**										
Absolute Weight	Gram	0.19	±	0.02	0.19	±	0.02	0.20	±	0.02
Per body weight	%	0.56	±	0.05	0.53	±	0.06	0.53	±	0.06
Per brain weight	%	40.10	±	4.75	38.54	±	3.98	39.42	±	3.63
**Kidney**										
Absolute Weight	Gram	0.57	±	0.08	0.56	±	0.04	0.57	±	0.08
Per body weight	%	1.66	±	0.22	1.59	±	0.14	1.51	±	0.20
Per brain weight	%	118.44	±	13.83	116.12	±	9.85	112.32	±	14.95
**Liver**										
Absolute Weight	Gram	2.12	±	0.26	2.33	±	0.12	2.61	±	0.35*
Per body weight	%	6.20	±	0.51	6.55	±	0.36	6.91	±	0.62*
Per brain weight	%	444.88	±	50.09	477.57	±	16.24	516.80	±	62.91*
**Lung**										
Absolute Weight	Gram	0.27	±	0.08	0.30	±	0.09	0.26	±	0.05
Per body weight	%	0.80	±	0.22	0.84	±	0.26	0.69	±	0.12
Per brain weight	%	57.37	±	16.79	60.83	±	17.47	51.41	±	10.01
**Spleen**										
Absolute Weight	Gram	0.09	±	0.02	0.10	±	0.01	0.15	±	0.05*
Per body weight	%	0.27	±	0.07	0.29	±	0.03	0.40	±	0.17
Per brain weight	%	19.54	±	4.92	20.96	±	1.99	29.42	±	10.82*

*Significantly different from saline control, p≤0.05 (ANOVA with Dunnett's T test).

Table V-5b. Recovery Study Organ Weights. The organ weights are displayed as absolute values, % body weight, and % brain weight by treatment group. Data is presented as the mean ± SD.

Treatment		Saline			NCL388-12 855 mg I/kg			NCL388-12 1715 mg I/kg		
Number of Animals		8			7*			8		
**Body Weight**										
Absolute Weight	Gram	36.38	±	1.57	38.06	±	2.58	39.09	±	4.37
Brain										
Absolute Weight	Gram	0.51	±	0.02	0.49	±	0.02	0.49	±	0.02
Per body weight	%	1.41	±	0.07	1.30	±	0.09	1.26	±	0.12*
Heart										
Absolute Weight	Gram	0.20	±	0.02	0.19	±	0.01	0.20	±	0.02
Per body weight	%	0.54	±	0.06	0.49	±	0.03	0.51	±	0.05
Per brain weight	%	38.20	±	4.12	37.90	±	2.16	40.30	±	3.65
Kidney										
Absolute Weight	Gram	0.59	±	0.05	0.59	±	0.05	0.56	±	0.08
Per body weight	%	1.64	±	0.14	1.55	±	0.11	1.43	±	0.11
Per brain weight	%	116.25	±	7.11	119.23	±	10.86	114.37	±	15.31*
Liver										
Absolute Weight	Gram	2.18	±	0.26	2.61	±	0.27*	3.20	±	0.51*
Per body weight	%	5.97	±	0.50	6.88	±	0.73*	8.16	±	0.78*
Per brain weight	%	425.56	±	44.54	531.16	±	66.59*	651.38	±	93.11*
Lung										
Absolute Weight	Gram	0.26	±	0.06	0.24	±	0.03	0.27	±	0.05
Per body weight	%	0.82	±	0.32	0.63	±	0.05	0.70	±	0.12
Per brain weight	%	58.02	±	20.20	48.69	±	5.99	55.56	±	10.20
Spleen										
Absolute Weight	Gram	0.10	±	0.02	0.17	±	0.07	0.27	±	0.12*
Per body weight	%	0.28	±	0.04	0.47	±	0.23	0.71	±	0.32*
Per brain weight	%	19.77	±	3.32	35.76	±	16.63	55.59	±	24.37*

*Significantly different from saline control, p≤0.05 (ANOVA with Dunnett's T test).

<u>Gross Pathology</u>

The gross lesions observed in the main and recovery animal groups are summarized in Table V-6. The findings of enlarged liver and spleen were most frequently observed in the high-dose groups, consistent with the with statistically significant increases in these organ weights displayed previously. Other gross lesions identified in each group were incidental and typical of this animal model. These lesions were observed inconsistently in both the treated and control animals and were not considered treatment related.

Table V-6a. Main Group Gross Pathology. Findings are displayed by treatment group.

ORGAN/TISSUE	Lesions	Treatment Group (Main)		
		Saline Vehicle	NCL388-12 855 mg l/kg*	NCL388-12 1715 mg l/kg
**Adrenal**	**# of Organs**	**8**	**7**	**8**
	Discoloration, pale, both	1/8		2/8
**Kidney**	**# of Organs**	**8**	**8**	**8**
	Absent, Right			1/8
	Discolored, Both			2/8
	Discolored, Left			1/8
	Enlarged, Left			1/8
**Liver**	**# of Organs**	**8**	**7**	**8**
	Discoloration, Pale, All lobes			1/8
	Enlarged, All lobes	4/8	5/7	7/8
**LN, mesenteric**	**# of Organs**	**8**	**7**	**8**
	Enlarged	3/8	5/7	5/8
**Lung**	**# of Organs**	**8**	**7**	**8**
	Discolored, Mottled, All lobes	5/8	6/7	5/8
	Enlarged, all lobes	1/8		
	Foci, Diaphragmatic lobe	1/8		
	Focus, Left lobe		1/7	
**Lymph Node**	**# of Organs**	**0**	**0**	**2**
	Enlarged, Axillary			2/2
**Pancreas**	**# of Organs**	**8**	**7**	**8**
	Discoloration, Pale	3/8	3/7	4/8
**Pinna (Ear)**	**# of Organs**	**0**	**1**	**0**
	Crust, Right		1/1	
**Small Intestine**	**# of Organs**	**8**		**8**
	Enlarged, GALTs	3/8	3/7	3/8
**Spleen**	**# of Organs**	**8**	**8**	**8**
	Discolored, Mottled			1/8
	Discolored, Pale	2/8		1/8
	Enlarged	2/8	1/7	7/8
**Urinary Bladder**	**# of Organs**	**8**	**7**	**8**
	Foreign body, Mucosa	1/8	4/7	1/8

Table V-6b. Recovery Group Gross Pathology. Findings are displayed by treatment group.

ORGAN/TISSUE	Lesions	Treatment Group (Recovery)		
		Saline Vehicle	NCL388-12 855 mg I/kg	NCL388-12 1715 mg I/kg
Liver	# of Organs	8	8	8
	Enlarged, All lobes			2/8
LN, mandibular	# of Organs	8	8	8
	Enlarged	3/8	2/8	1/8
LN, mesenteric	# of Organs	8	8	8
	Enlarged	3/8	6/8	7/8
Lung	# of Organs	8	8	8
	Discolored, Mottled, All lobes	3/8	2/8	3/8
	Focus, Left lobe			1/8
	Foci, Left lobe	1/8		
	Focus, Azygous lobe	1/8		
	Focus, Diaphragmatic lobe	1/8		
Lymph Node	# of Organs	8	8	8
	Enlarged, Axillary		4/8	2/8
	Enlarged, Cervical		1/8	
	Enlarged, Iliac		1/8	
Pancreas	# of Organs	8	8	8
	Discoloration, Pale	2/8	1/8	3/8
Small Intestine	# of Organs	8	8	
	Enlarged, GALTs	2/8	4/8	3/8
Spleen	# of Organs	8	8	8
	Enlarged		2/8	6/8
Urinary Bladder	# of Organs	8	8	8
	Foreign body, Mucosa		1/8	2/8

Histopathology Summary
Table V-7 summarizes the graded histopathological findings observed in the main and recovery groups. A comprehensive set of tissues were evaluated for histopathological analysis in addition to tissues that showed gross lesions identified during necropsy by a board-certified veterinary pathologist. Figure V-1 shows representative H&E images of histological findings for each group.

All treated mice in both main and recovery groups developed similar changes which were characterized by multisystemic histiocytosis. The histiocytic infiltrate ranges from mild to severe, is present in all organs, and is dose-dependent. The presence of this histiocytosis was statistically significant across evaluated organs when compared to respective saline controls. *NB*: The histiocytosis noted is most likely the result of iodine polymer accumulation in tissue macrophage, as has been shown for other biopersistent polymers such as PEG with PEGylated therapeutics [15], and in this study was not associated with other histopathological changes, nor associated with alterations in hematological or clinical chemistry parameters indicative of toxicity.

Multiple mice in both control and treated groups developed lung inflammation and vasculitis. These lung lesions are related to presence of foreign materials (e.g., hair shaft) which may be caused by repeated injections via tail vein and were not statistically significant between treatment groups. Benign proliferative adrenal lesions were present in two mice in the main saline control group and main high-dose group. These findings were not statistically significant and are considered as CD-1 background lesions. The remaining histological lesions identified were of mild severity and interpreted as background changes commonly seen in mice of this strain. A copy of the pathologist’s narrative is provided in Appendix A of this report.

Table V-7a. Main Group Histopathology Findings. Findings are displayed by treatment group.

*Significantly different from respective saline control, p<0.05 (Kruskal-Wallis nonparametric ANOVA with multiple comparisons post hoc).

			Treatment Group (Main)		
ORGAN/TISSUE	Lesions	Grade	Saline Vehicle	NCL388-12 855 mg l/kg	NCL388-12 1715 mg l/kg
Adrenal	# of Organs		8	8	8
	Angiectasis	++			2/8
	Histiocytosis	+		1/8	3/8*
		++		1/8	4/8*
	Hyperplasia, subscapular cell	+	1/8	1/8	
Brain	# of Organs		8	8	8
	Histiocytes, choroid plexus	+		6/8	2/8*
		++		1/8	6/8*
Cecum	# of Organs		8	8	8
	Histiocytosis	+		6/8*	3/8*
		++		1/8*	5/8*
Coagulating Gland	# of Organs		8	8	8
	Histiocytosis	+		5/8	3/8*
		++			5/8*
Colon	# of Organs		8	8	8
	Histiocytosis	+		5/8	
		++		2/8	8/8*
		+++			
	Hyperplasia, GALT	++	1/8		
Duodenum	# of Organs		8	8	8
	Histiocytosis	+		7/8*	4/8*
		++			4/8*
	Hyperplasia, GALT	++			
Epididymis	# of Organs		8	8	8

	Histiocytosis	+		6/8	
		++		1/8	8/8*
		+++			
Esophagus	# of Organs		8	8	8
	Cellulitis with edema	++		1/8	1/8
	Histiocytosis	+		7/8*	6/8*
		++			2/8*
Eye	# of Organs		8	8	7
	Histiocytosis	+		7/8	2/7*
		++			5/7*
Femur	# of Organs		8	8	8
Femur, Marrow	# of Organs		8	8	8
	Histiocytosis	+		2/8	
		++			3/8
		+++			
	Hyperplasia, granulocytic	++		1/8	
Gall Bladder	# of Organs		8	8	8
	Histiocytosis	+		6/8	2/8*
		++		1/8	6/8*
Harderian Gland	# of Organs		8	8	8
	Dilation	++			
	Histiocytosis	+			1/8
		++			1/8
	Pigment	+	2/8	5/8	1/8
		++	6/8	3/8	7/8
		+++			
Heart	# of Organs		8	8	8
	Histiocytosis	+		1/8*	
		++		6/8*	3/8*
		+++			4/8*
Ileum	# of Organs		8	8	8
	Histiocytosis	+		7/8*	5/8*
		++			3/8*

	Hyperplasia, GALT	++			
Jejunum	# of Organs		8	8	8
	Histiocytosis	+		7/8*	5/8*
		++			3/8*
	Hyperplasia, GALT	++			
	Lymphoplasmacytic infiltrate	++			1/8
Kidney	# of Organs		7	8	8
	Degeneration, tubule	+			1/8
	Dilatation, renal pelvis	++			
	Dilatation, tubule	++	1/7		
	Histiocytosis	+			4/8
		++			
	Inflammation, subacute	+	1/7		
Liver	# of Organs		8	8	8
	Degeneration	+			1/8
	Extramedullary hematopoiesis	+	1/8		
	Histiocytosis	++		7/8	3/8*
		+++			5/8*
		++++			
	Inflammation, active chronic	+			1/8
		++			
		+++			
	Inflammation, granulomatous	+++			
	Inflammation, histiocytic	+			
	Necrosis, coagulative	++			
	Vacuolation, centrilobular	+		1/8	
	Vacuolation, hepatocellular, increased	++	1/8		
LN, mandibular	# of Organs		8	8	8
	Hemorrhage	+		1/8	
	Histiocytosis	+	3/8	1/8	
		++		3/8	5/8
		+++	1/8	2/8	2/8

		++++		1/8	
	Hyperplasia, lymphoid	+	3/8	1/8	2/8*
		++		1/8	5/8*
	Inflammation, acute	++			
LN, mesenteric	# of Organs		8	8	8
	Histiocytosis	+			
		++		4/8*	1/8*
		+++		3/8*	7/8*
		++++			
	Hyperplasia, lymphoid	+	2/8	1/8	1/8
		++	6/8	6/8	6/8
Lung	# of Organs		8	8	8
	Foreign material, bronchi(ole) lumen	+		1/8	
		++		1/8	
	Foreign material, vascular lumen	++	2/8		
		+++	1/8	1/8	
	Hemorrhage	+	1/8		
		++	1/8		
	Histiocytosis, alveolar	+	1/8	3/8*	3/8*
		++		4/8*	3/8*
		+++			1/8*
	Inflammation, chronic active	++	1/8	2/8	1/8
		+++	1/8		1/8
	Lymphocytic infiltrate	+	1/8		1/8
	Vasculitis	+	1/8		
		++	2/8		
		+++	2/8		1/8
Lymph Node	# of Organs		0	0	1
	Histiocytosis	+++			1/1
Mesentery	# of Organs		0	0	0
	Intravascular foamy histiocytes	++++			
Muscle	# of Organs		0	8	9
	Histiocytosis	+		6/8*	3/9*

		++		2/8*	6/9*
	Inflammation, chronic active	++		1/8	
**Nasal Sections**	**# of Organs**		8	8	8
**Pancreas**	**# of Organs**		8	8	8
	**Histiocytosis**	+		6/8	
		++		1/8	8/8*
		+++			
	**Inflammation, subacute**	+++			1/8
	**Necrosis**	++++			1/8
**Parathyroid**	**# of Organs**		8	8	5
	**Histiocytosis**	+			3/5
		++			
**Pinna (Ear)**	**# of Organs**		0	1	0
	**Histiocytosis**	+++		1/1	
	**Inflammation, acute**	++++		1/1	
**Pituitary**	**# of Organs**		8	8	8
	**Cyst**	+			
	**Histiocytosis**	+			5/8
		++		1/8	
**Prostate**	**# of Organs**		8	8	8
	**Histiocytosis**	+		6/8*	3/8*
		++		1/8*	5/8*
**Rectum**	**# of Organs**		8	8	8
	**Histiocytosis**	+		7/8*	5/8*
		++			3/8*
**Salivary Gland**	**# of Organs**		8	8	8
	**Histiocytes**	+		6/8	
		++			3/8
	**Histiocytosis**	++		1/8	5/8
		+++			
	**Lymphocytic infiltrate**	+			1/8
**Seminal Vesicle**	**# of Organs**		8	8	8
	**Histiocytosis**	+		3/8	5/8*

		++			3/8*
	Hyperplasia	+		2/8	
	Hyperplasia	++		1/8	1/8
**Skin/Subcutis**	**# of Organs**		8	9	8
Skin/Subcutis	Histiocytosis	+		1/9	
Skin/Subcutis	Histiocytosis	++		6/9	3/8*
Skin/Subcutis	Histiocytosis	+++			4/8*
Skin/Subcutis	Histiocytosis	++++			1/8*
**Small Intestine**	**# of Organs**		0	0	0
**Spinal Cord**	**# of Organs**		8	8	8
Spinal Cord	Hemorrhage	++		1/8	
**Spleen**	**# of Organs**		8	8	8
Spleen	Atrophy. white pulp	+			1/8
Spleen	Extramedullary hematopoiesis	++		1/8	1/8
Spleen	Extramedullary hematopoiesis	+++			1/8
Spleen	Histiocytosis	+		6/8	
Spleen	Histiocytosis	++		1/8	
Spleen	Histiocytosis	+++			8/8*
Spleen	Histiocytosis	++++			
Spleen	Hyperplasia, lymphoid	+	2/8		
Spleen	Hyperplasia, lymphoid	++			
**Stomach, glandular**	**# of Organs**		8	8	8
Stomach, glandular	Bacteria	+	1/8		
Stomach, glandular	Histiocytosis	+		7/8	2/8*
Stomach, glandular	Histiocytosis	++			6/8*
**Stomach, nonglandular**	**# of Organs**		8	8	8
Stomach, nonglandular	Histiocytosis	+		7/8	2/8*
Stomach, nonglandular	Histiocytosis	++			6/8*
**Testis**	**# of Organs**		8	8	8
Testis	Histiocytosis	+		5/8	
Testis	Histiocytosis	++		1/8	3/8*
Testis	Histiocytosis	+++		1/8	5/8*
**Thymus**	**# of Organs**		8	8	8

	Histiocytosis	+		1/8	
		++		5/8	3/8*
		+++			5/8*
Thyroid	# of Organs		8	8	8
	Dilation, follicle	+		2/8	
		++		1/8	2/8
	Histiocytosis	+		6/8	2/8*
		++		1/8	6/8*
		+++			
Tongue	# of Organs		8	8	8
	Histiocytosis	++		4/8*	5/8*
		+++		3/8*	3/8*
Trachea	# of Organs		8	8	8
	Cellulitis with histiocytic inflammation	++			2/8
	Histiocytosis	+		6/8*	6/8*
		++			1/8*
		+++			
Urinary Bladder	# of Organs		8	8	8
	Histiocytosis	+		7/8	3/8*
		++			5/8*
	Proteinaceous plugs	++	1/8		
		+++	1/8		
Vertebra	# of Organs		8	8	8

Table V-7b. Recovery Group Histopathology Findings. Findings are displayed by treatment group.
*Significantly different from respective saline control, $p<0.05$ (Kruskal-Wallis nonparametric ANOVA with multiple comparisons post hoc).

			Treatment Group (Recovery)		
ORGAN/TISSUE	Lesions	Grade	Saline Vehicle	NCL388-12 855 mg l/kg	NCL388-12 1715 mg l/kg
Adrenal	# of Organs		7	8	8
	Angiectasis	++			1/8
	Histiocytosis	+		7/8*	4/8*
		++		1/8*	3/8*
	Hyperplasia, subscapular cell	+			
Brain	# of Organs		8	8	8
	Histiocytes, choroid plexus	+		4/8*	2/8*
		++		4/8*	6/8*
Cecum	# of Organs		8	8	8
	Histiocytosis	+		2/8*	
		++		6/8*	8/8*
Coagulating Gland	# of Organs		8	8	8
	Histiocytosis	+		4/8*	1/8*
		++		3/8*	7/8*
Colon	# of Organs		8	8	8
	Histiocytosis	+		1/8*	
		++		6/8*	8/8*
		+++		1/8*	
	Hyperplasia, GALT	++			
Duodenum	# of Organs		8	8	8
	Histiocytosis	+		2/8*	
		++		6/8*	8/8*
	Hyperplasia, GALT	++	1/8		
Epididymis	# of Organs		8	8	8
	Histiocytosis	+		1/8*	

		++		7/8*	5/8*
		+++			3/8*
**Esophagus**	**# of Organs**		8	8	8
	**Cellulitis with edema**	++			
	**Histiocytosis**	+		3/8	
		++		3/8	8/8*
**Eye**	**# of Organs**		8	8	8
	**Histiocytosis**	+		2/8	4/8*
		++		1/8	4/8*
**Femur**	**# of Organs**		8	8	8
**Femur, Marrow**	**# of Organs**		8	8	8
	**Histiocytosis**	+		2/8	
		++		2/8	4/8*
		+++			4/8*
	**Hyperplasia, granulocytic**	++			
**Gall Bladder**	**# of Organs**		8	6	7
	**Histiocytosis**	+		5/6	
		++		1/6	7/7*
**Harderian Gland**	**# of Organs**		8	8	8
	**Dilation**	++			1/8
	**Histiocytosis**	+		3/8	2/8*
		++		1/8	6/8*
	**Pigment**	+	1/8		
		++	5/8	6/8	7/8
		+++			1/8
**Heart**	**# of Organs**		8	8	8
	**Histiocytosis**	+			
		++		6/8*	
		+++		2/8*	8/8*
**Ileum**	**# of Organs**		8	8	8
	**Histiocytosis**	+		2/8*	
		++		6/8*	8/8*
	**Hyperplasia, GALT**	++			1/8

**Jejunum**	**# of Organs**		8	8	8
	**Histiocytosis**	**+**		2/8*	
		**++**		6/8*	8/8*
	**Hyperplasia, GALT**	**++**			1/8
	**Lymphoplasmacytic infiltrate**	**++**			
**Kidney**	**# of Organs**		8	8	8
	**Degeneration, tubule**	**+**			
	**Dilatation, renal pelvis**	**++**	1/8		
	**Dilatation, tubule**	**++**			
	**Histiocytosis**	**+**		2/8	2/8*
		**++**			5/8*
	**Inflammation, subacute**	**+**			
**Liver**	**# of Organs**		8	8	8
	**Degeneration**	**+**			
	**Extramedullary hematopoiesis**	**+**			
	**Histiocytosis**	**++**			
		**+++**		8/8*	7/8*
		**++++**			1/8*
	**Inflammation, active chronic**	**+**			
		**++**		3/8	3/8
		**+++**		2/8	2/8
	**Inflammation, granulomatous**	**+++**			1/8
	**Inflammation, histiocytic**	**+**	1/8		
	**Necrosis, coagulative**	**++**			2/8
	**Vacuolation, centrilobular**	**+**			
	**Vacuolation, hepatocellular, increased**	**++**			
**LN, mandibular**	**# of Organs**		8	8	8
	**Hemorrhage**	**+**			
	**Histiocytosis**	**+**	4/8		
		**++**	1/8	4/8*	3/8
		**+++**	1/8	3/8*	4/8
		**++++**		1/8*	

	Hyperplasia, lymphoid	+	2/8		
		++	2/8		1/8
	Inflammation, acute	++			1/8
LN, mesenteric	# of Organs		8	8	8
	Histiocytosis	+	4/8		
		++	2/8	1/8	
		+++		4/8	2/8*
		++++		3/8	6/8*
	Hyperplasia, lymphoid	+			
		++	8/8	2/8*	2/8*
Lung	# of Organs		8	8	8
	Foreign material, bronchi(ole) lumen	+			
		++			
	Foreign material, vascular lumen	++	1/8		
		+++			
	Hemorrhage	+	2/8		
		++	2/8	1/8	2/8
	Histiocytosis, alveolar	+		3/8	1/8*
		++		3/8	6/8*
		+++			1/8*
	Inflammation, chronic active	++	1/8		
		+++			
	Lymphocytic infiltrate	+			
	Vasculitis	+			
		++	1/8		
		+++	1/8		
Lymph Node	# of Organs		0	0	0
	Histiocytosis	+++			
Mesentery	# of Organs		0	0	2
	Intravascular foamy histiocytes	++++			2/2
Muscle	# of Organs		0	6	8
	Histiocytosis	+		2/6	1/8*
		++		3/6	7/8*

	Inflammation, chronic active	++			
Nasal Sections	# of Organs		8	8	7
Pancreas	# of Organs		8	8	8
	Histiocytosis	+			
		++		5/8*	2/8*
		+++		3/8*	6/8*
	Inflammation, subacute	+++			
	Necrosis	++++			
Parathyroid	# of Organs		5	4	5
	Histiocytosis	+		3/4	4/5*
		++			1/5*
Pinna (Ear)	# of Organs		0	0	0
	Histiocytosis	+++			
	Inflammation, acute	++++			
Pituitary	# of Organs		8	8	8
	Cyst	+	1/8		
	Histiocytosis	+		5/8*	4/8*
		++		2/8*	4/8*
Prostate	# of Organs		8	7	6
	Histiocytosis	+		2/7*	1/6*
		++		5/7*	5/6*
Rectum	# of Organs		8	8	8
	Histiocytosis	+		2/8*	
		++		6/8*	8/8*
Salivary Gland	# of Organs		8	8	8
	Histiocytes	+			
		++		1/8	
	Histiocytosis	++		5/8	
		+++		2/8	8/8*
	Lymphocytic infiltrate	+			
Seminal Vesicle	# of Organs		8	8	8
	Histiocytosis	+		7/8*	2/8*
		++		1/8*	6/8*

	Hyperplasia	+			
		++			
Skin/Subcutis	# of Organs		8	8	8
	Histiocytosis	+			
		++		7/8	
		+++		1/8	5/8*
		++++			3/8*
Small Intestine	# of Organs		2	0	0
Spinal Cord	# of Organs		8	8	8
	Hemorrhage	++			
Spleen	# of Organs		8	8	8
	Atrophy. white pulp	+			
	Extramedullary hematopoiesis	++			
		+++			
	Histiocytosis	+			
		++			
		+++		6/8*	2/8*
		++++		2/8*	6/8*
	Hyperplasia, lymphoid	+			
		++	1/8		
Stomach, glandular	# of Organs		8	8	8
	Bacteria	+			
	Histiocytosis	+		2/8*	
		++		6/8*	8/8*
Stomach, nonglandular	# of Organs		8	8	8
	Histiocytosis	+		3/8*	
		++		5/8*	8/8*
Testis	# of Organs		8	8	8
	Histiocytosis	+			
		++		3/8*	2/8*
		+++		5/8*	6/8*
Thymus	# of Organs		8	8	8
	Histiocytosis	+			

		++		1/8*	1/8*
		+++		7/8*	7/8*
Thyroid	# of Organs		8	8	8
	Dilation, follicle	+			
		++			1/8
	Histiocytosis	+	1/8		
		++		7/8*	3/8*
		+++		1/8*	5/8*
Tongue	# of Organs		8	8	8
	Histiocytosis	++		6/8*	3/8*
		+++		1/8*	5/8*
Trachea	# of Organs		8	8	8
	Cellulitis with histiocytic inflammation	++			
	Histiocytosis	+		4/8	
		++		3/8	7/8*
		+++			1/8*
Urinary Bladder	# of Organs		8	8	8
	Histiocytosis	+		3/8*	
		++		5/8*	8/8*
	Proteinaceous plugs	++		1/8	
		+++	1/8		
Vertebra	# of Organs		8	8	8

Figure V-1A. Saline Control Liver Histological Images. Representative H&E sections of liver are shown from saline control group animals. No foamy histiocytes were noted.

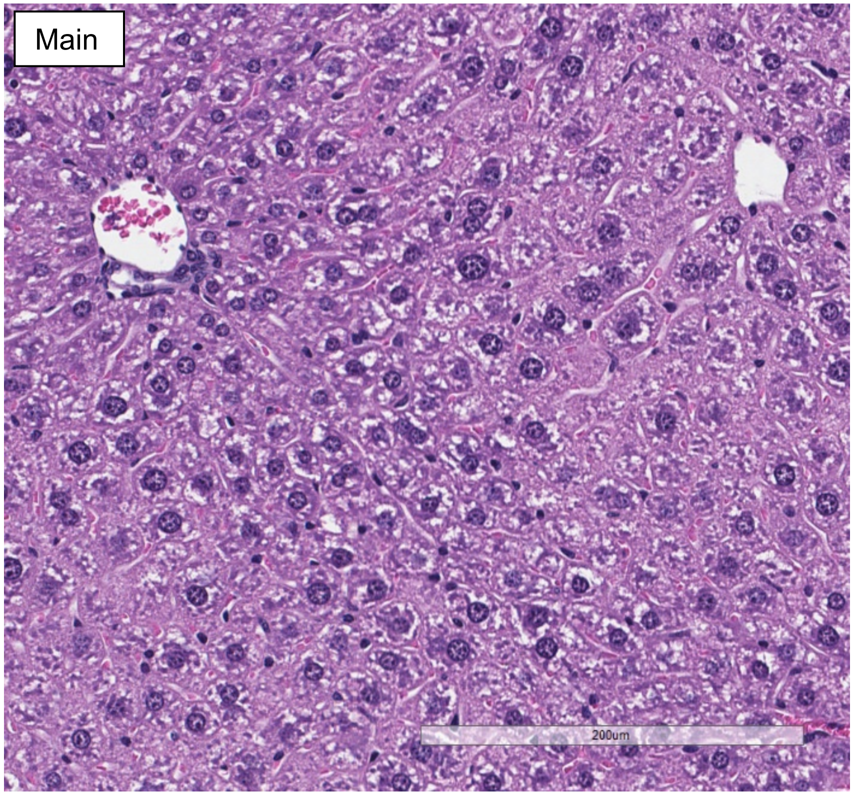

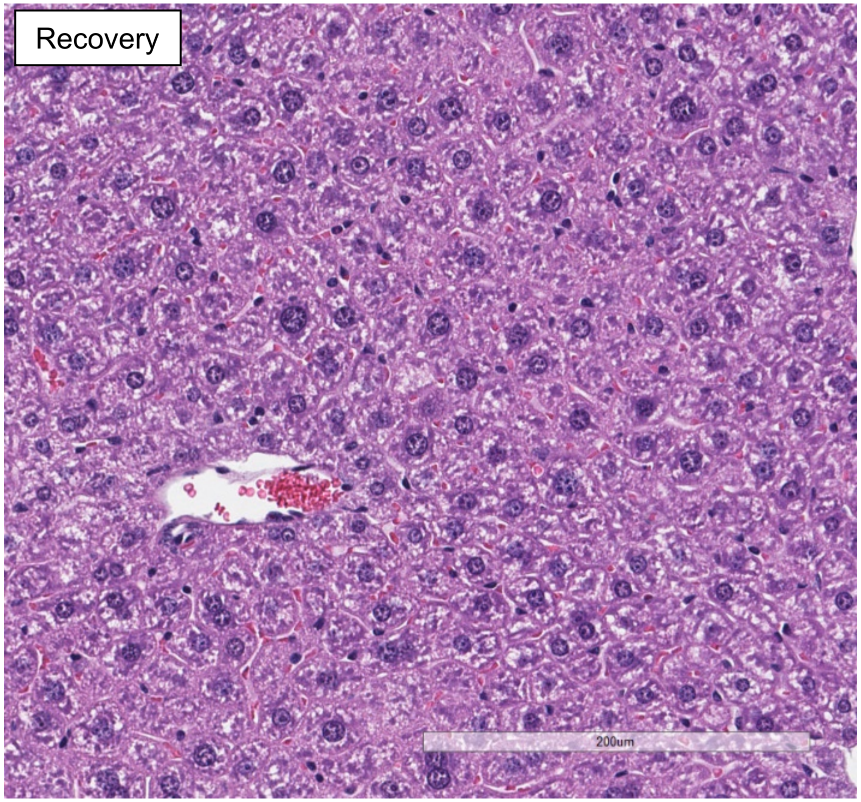

Figure V-1B. Low-Dose Liver Histological Images. Representative H&E sections of liver are shown from low-dose groups. Foamy histiocytes were noted in both groups; the presentation in the main group is mild, while the recovery group is moderate to severe.

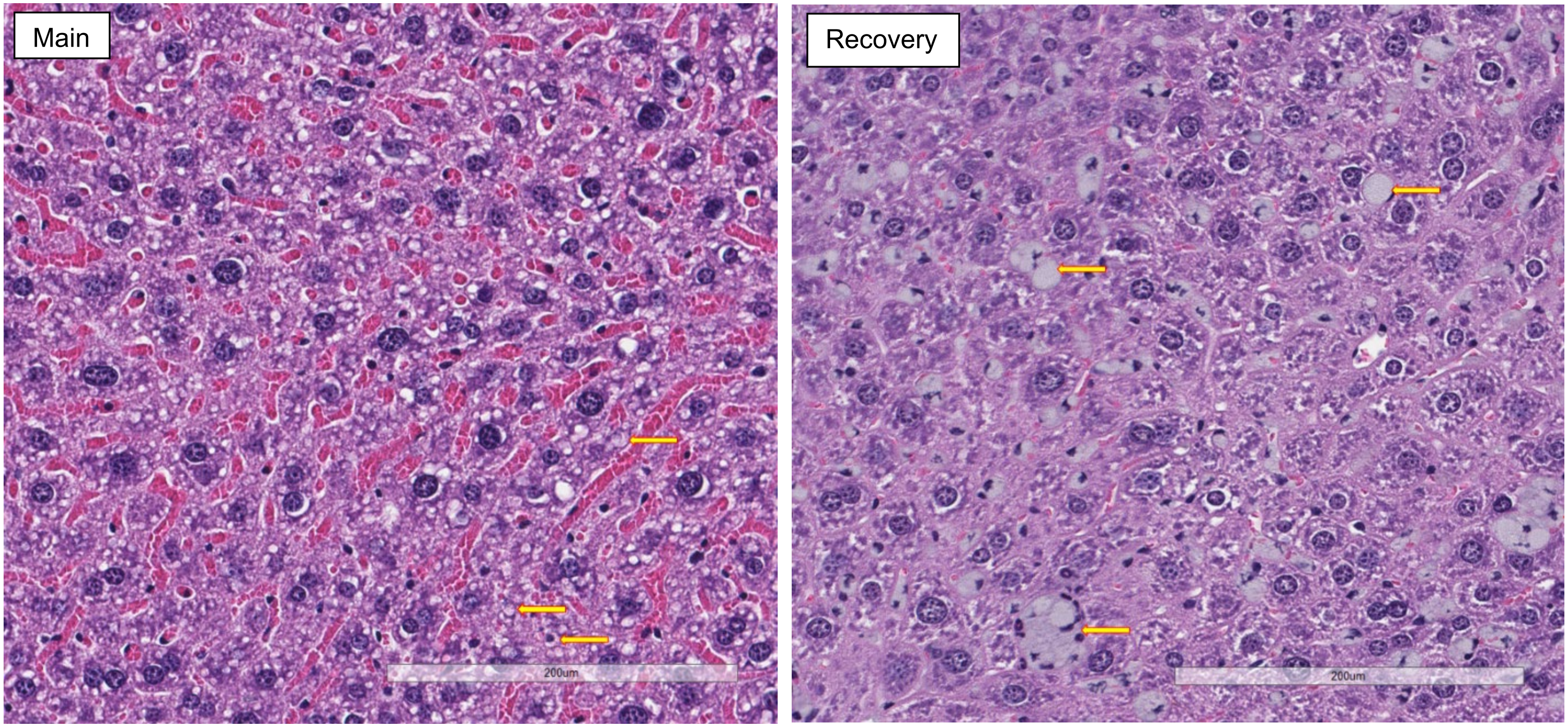

Figure V-1C. High-Dose Liver Histological Images. Representative H&E sections of liver are shown from high-dose groups. Foamy histiocytes were noted in both groups; main groups contain moderate levels, while recovery groups are more severely affected.

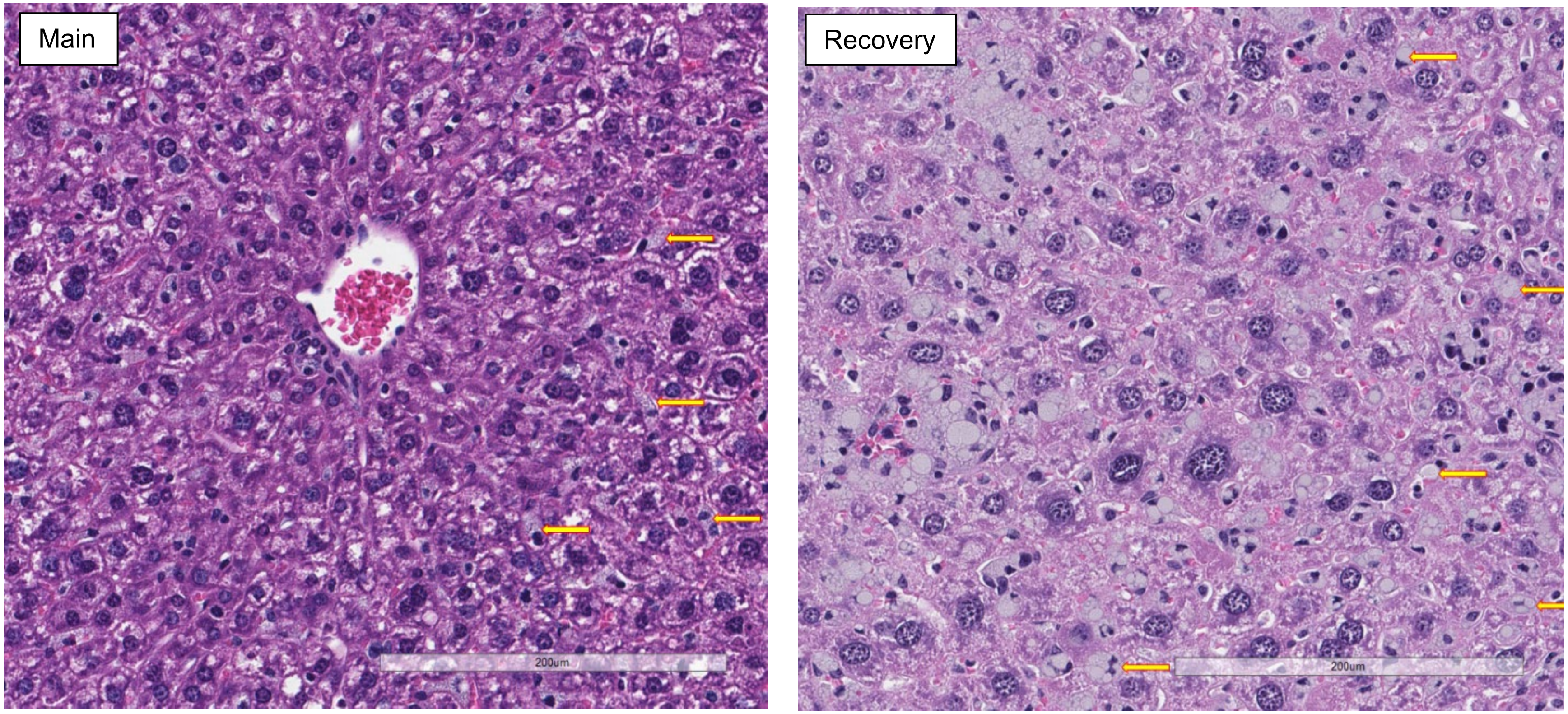

Molecular Pathology Summary

Immunohistochemistry analysis for the macrophage marker Anti-F4/80 was performed on liver sections from saline control, high dose main, and high dose recovery groups to confirm the diagnosis of histiocytosis. Slides were scanned at 200x in a single z-plane with an Aperio ScanScope XT (Leica) and analyzed using a HALO cytonuclear algorithm.

Figure V-2 shows representative immunohistochemistry images of the anti-F4/80 immunoassay from both main and recovery high dose groups, along with the saline control. The number of anti-F4/80+ cells was significantly increased in both high dose groups, compared to the saline control. The total number of anti-F4/80+ cells was also significantly increased in the recovery group, compared to the main group in high-dose animals. Individual IHC parameters can be found in Appendix B.

Figure V-2A. Saline Control Immunohistochemistry Images. Representative F4/80 immunohistochemistry images of liver are shown at 200X magnification from saline control (untreated) animals. Normal quantity/morphology of macrophages is present.

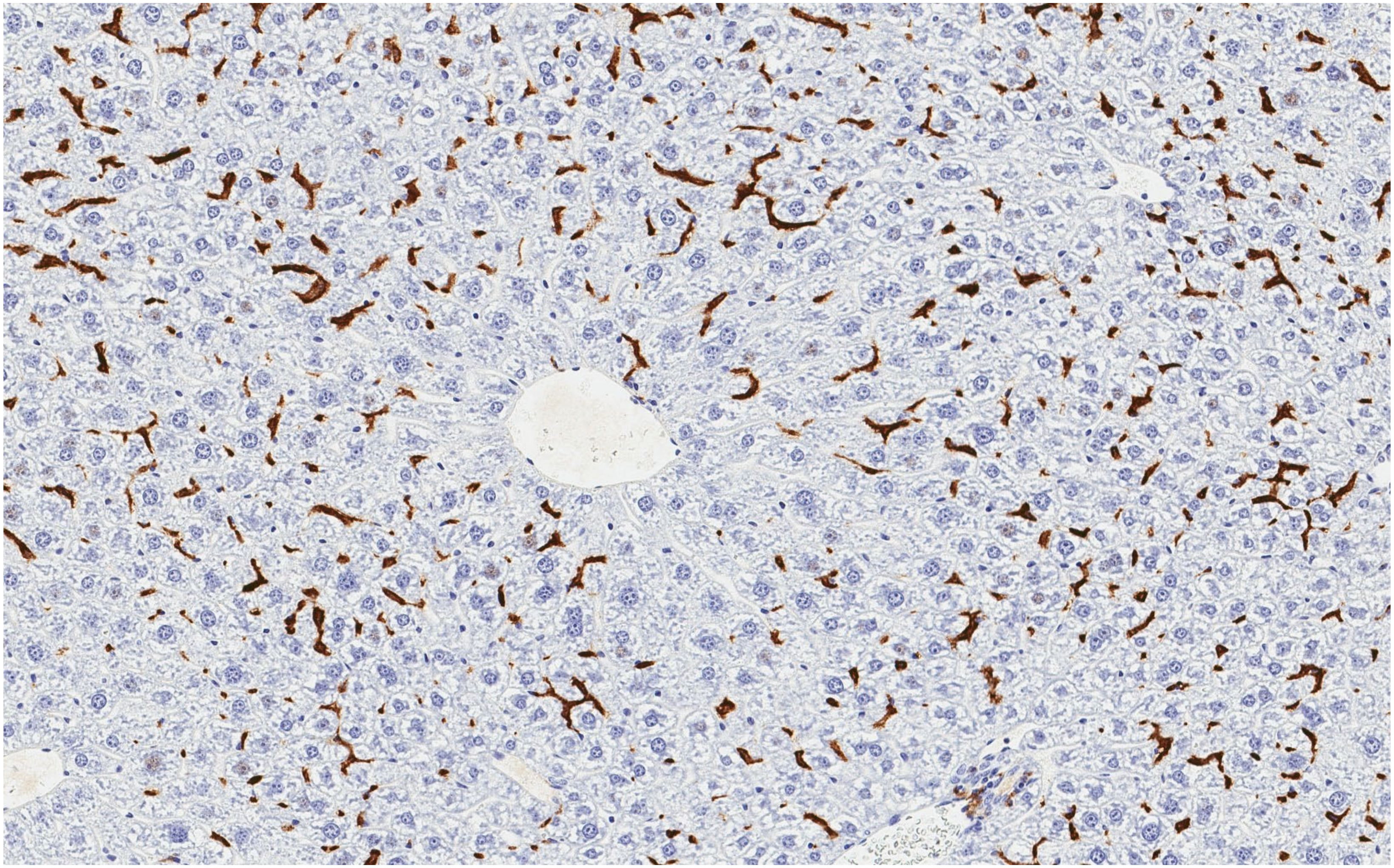

Figure V-2B. High-Dose Main Group Liver Immunohistochemistry Images. Representative images of F40/80 immunohistochemistry of liver are shown at 200X magnification from the high-dose main group. An increased number of foamy anti-F4/80-positive cells are present.

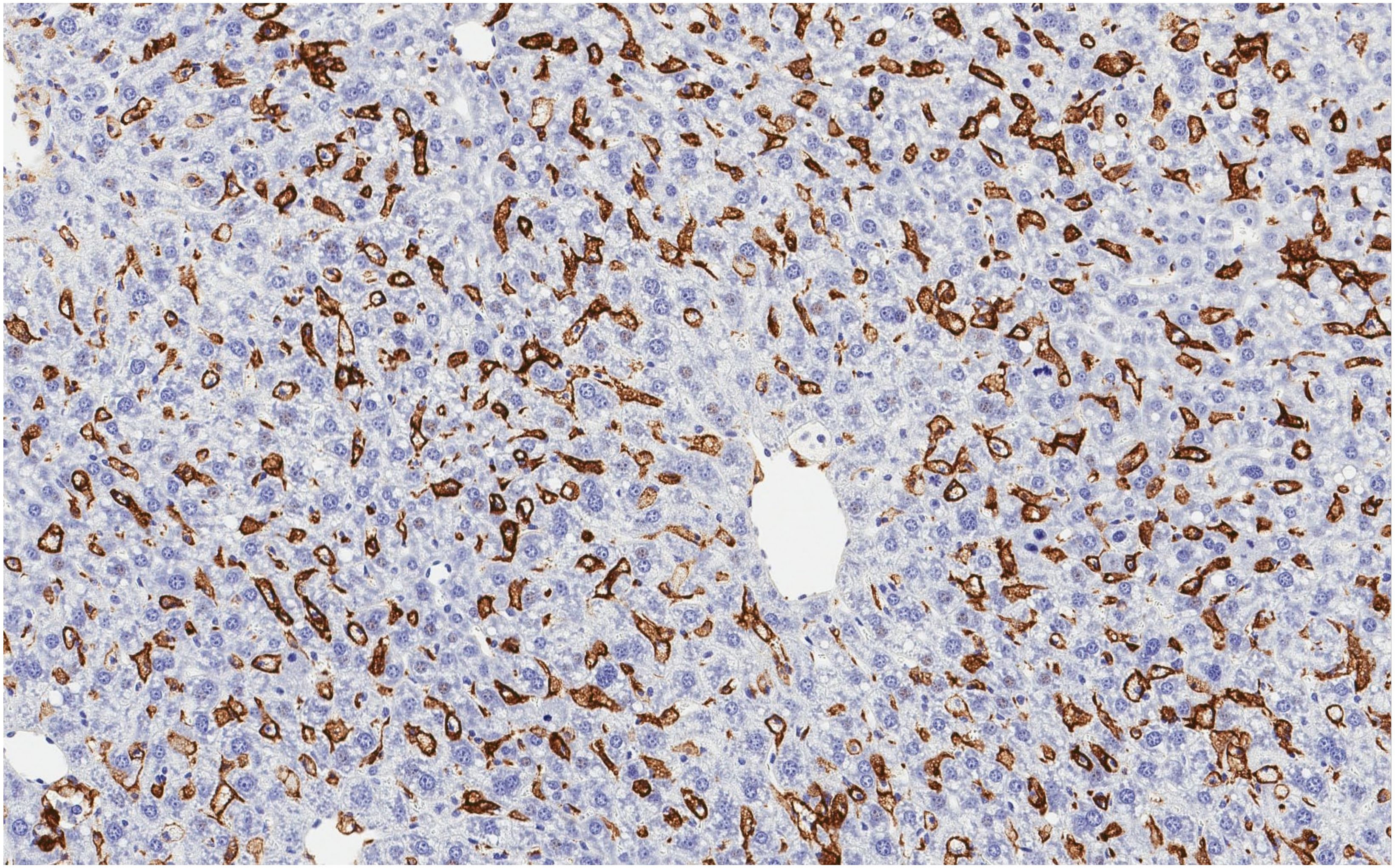

Figure V-2C. High-Dose Recovery Liver Immunohistochemistry Images. Representative F4/80 immunohistochemistry images of liver are shown at 200X magnification from the high-dose recovery group. An increased number of foamy anti-F4/80-positive cells are present.

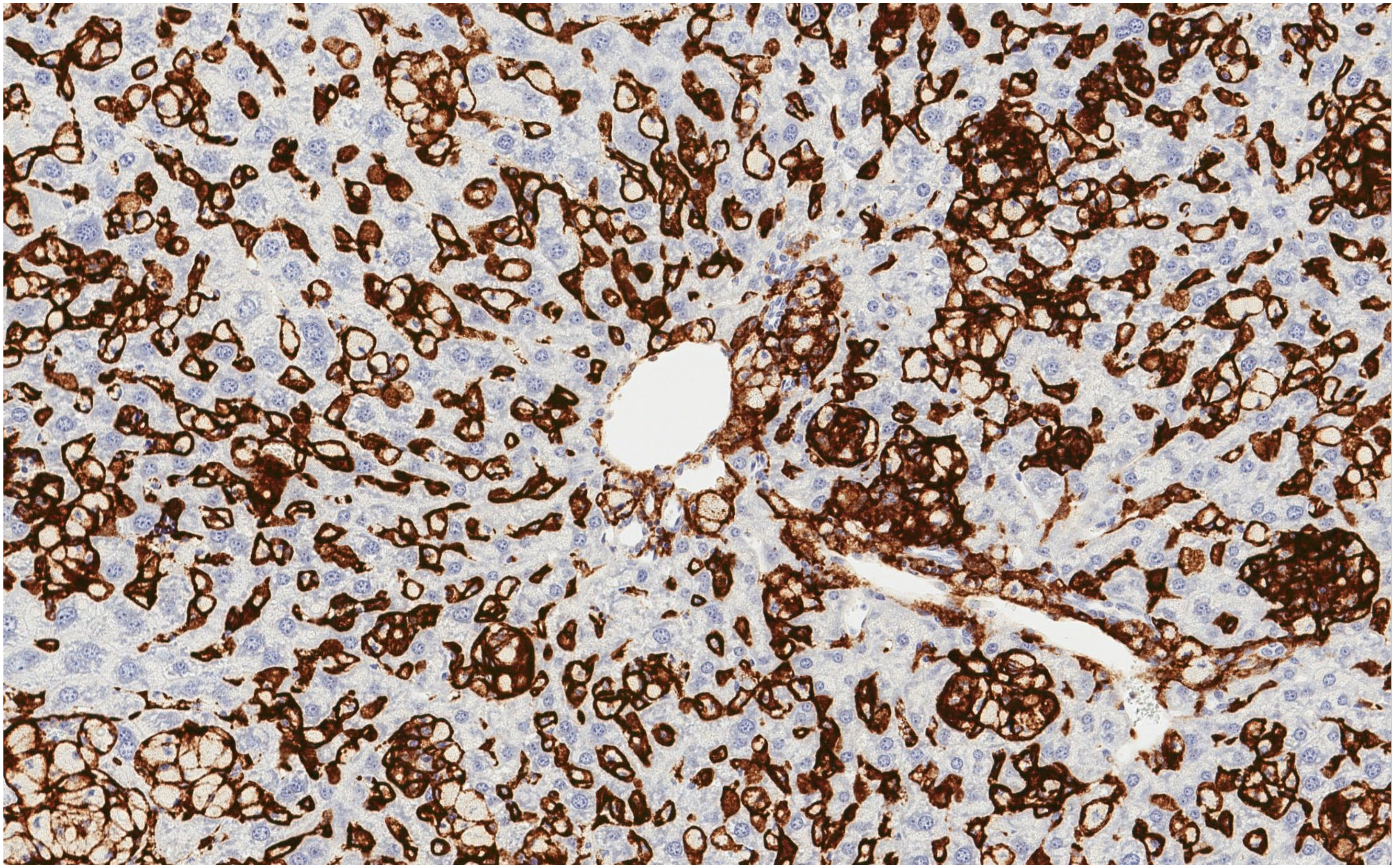

ICP-MS Analysis of Feces and Urine

The results of the ICP-MS analysis of 8-hr feces and urine samples from the main and recovery animal groups are summarized in Table V-8. The overall percentage of excreted iodine was calculated for each matrix by cage and treatment group. Total percentages for iodine excreted in urine or feces did not exceed 1% in any sample.

Table V-8. Mouse Urine/Feces Iodine Quantification. Findings are displayed by treatment group, per cage.

Treatment		NCL388-12 855 mg I/kg Main	NCL388-12 855 mg I/kg Recovery	NCL388-12 1715 mg I/kg Main	NCL388-12 1715 mg I/kg Recovery	NCL388-12 855 mg I/kg Recovery	NCL388-12 1715 mg I/kg Recovery
Number of Animals		8*	8	8	8	8	8
		DAY 5				DAY 18	
**8 hr Feces**							
Cage 1							
ICP-MS Total I	mg	1.40	1.24	4.29	2.53	0.09	0.27
I Administered	mg	467.51	493.85	1007.91	995.04	493.85	995.04
% Excreted	%	**0.30**	**0.25**	**0.43**	**0.25**	**0.02**	**0.03**
Cage 2							
ICP-MS Total I	mg	2.58	2.76	2.18	1.32	0.13	0.27
I Administered	mg	397.32	466.74	945.31	951.48	466.74	951.48
% Excreted	%	**0.65**	**0.59**	**0.23**	**0.14**	**0.03**	**0.03**
**8 hr Urine**							
Cage 1							
ICP-MS Total I	mg	2.20	4.50	9.79	6.61	0.17	0.39
I Administered	mg	467.51	493.85	1007.91	995.04	493.85	995.04
% Excreted	%	**0.47**	**0.91**	**0.97**	**0.66**	**0.03**	**0.04**
Cage 2							
ICP-MS Total I	mg	1.27	2.04	4.38	5.32	0.22	0.32
I Administered	mg	397.32	466.74	945.31	951.48	466.74	951.48
% Excreted	%	**0.32**	**0.44**	**0.46**	**0.56**	**0.05**	**0.03**

*One animal died post-dose after receiving the 2nd dose, in the low-dose main group (cage 2).

Electron Microscopy Analysis of Liver

Representative annotated images from electron microscopy analysis of liver are shown in Figure V-3. One sample per recovery treatment group was included in analysis.

Most hepatocytes from saline control sample appear to be normal and contain many glycogen granules, mitochondria, and rERs. There are also a few depleted glycogen granules and odd-shaped RBCs. Most Kupffer cells are normal, while some contain large lipid vacuoles. Liver samples from recovery treated animals (M04 and M06) contained hepatocytes that appeared normal and were comparable to the saline control animal. However, most endothelial and Kupffer cells in M04 and M06 samples contain medium to large vacuoles, filled with material tentatively identified as iodine polymer.

Figure V-3A. Saline Control Liver Electron Microscopy. A representative image of liver displaying hepatocytes and sinusoidal endothelial cells from the saline control group is shown.

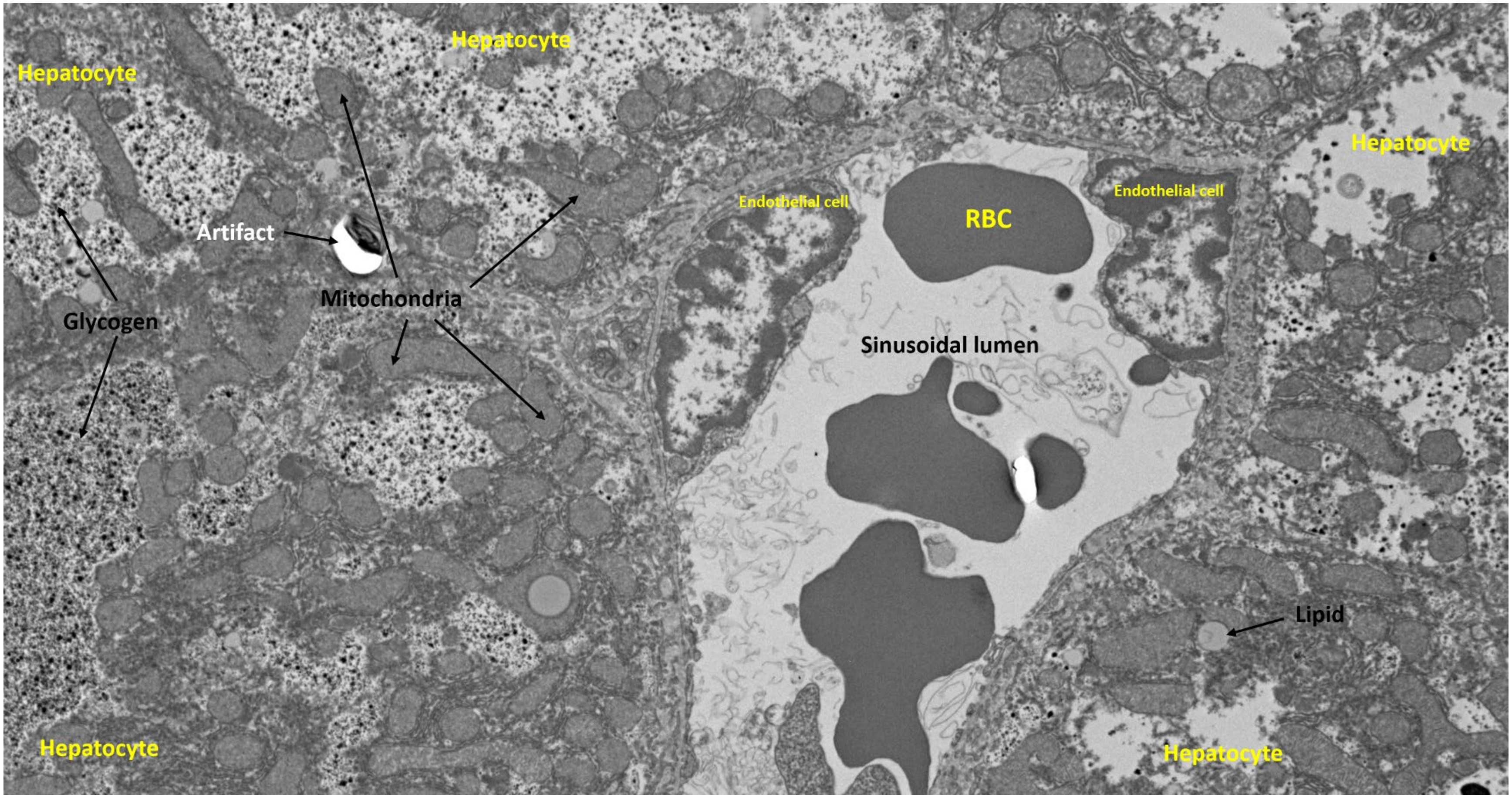

Figure V-3B. Recovery Low-Dose Liver Electron Microscopy. A representative image of liver displaying hepatocytes and sinusoidal endothelial cells from the low dose recovery group is shown. Note the presence of vacuoles.

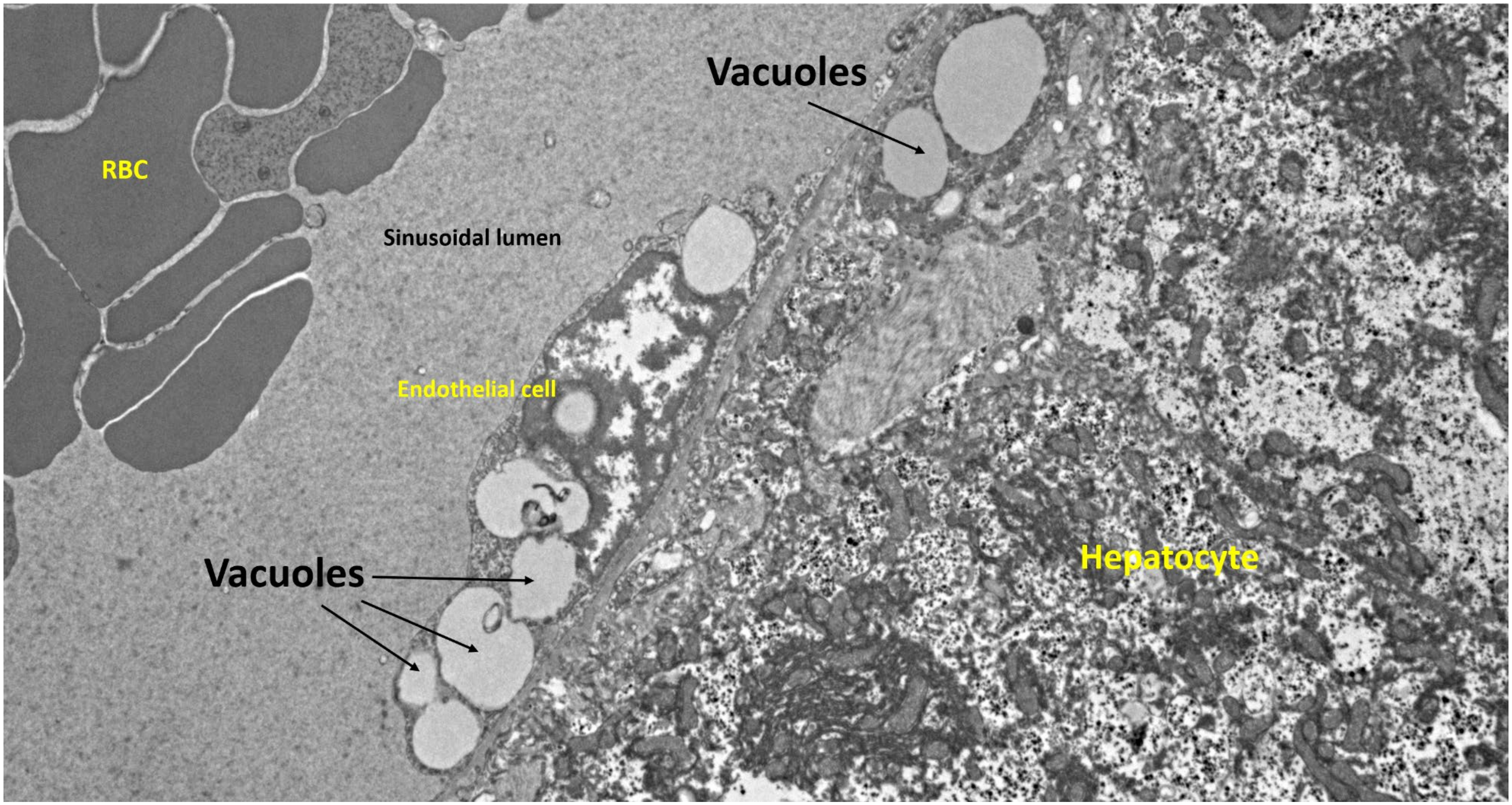

Figure V-3C. Recovery High-Dose Liver Electron Microscopy. A representative image of liver displaying hepatocytes (left) and sinusoidal endothelial cells (right) from the recovery high-dose group is shown. Note the presence of vacuoles.

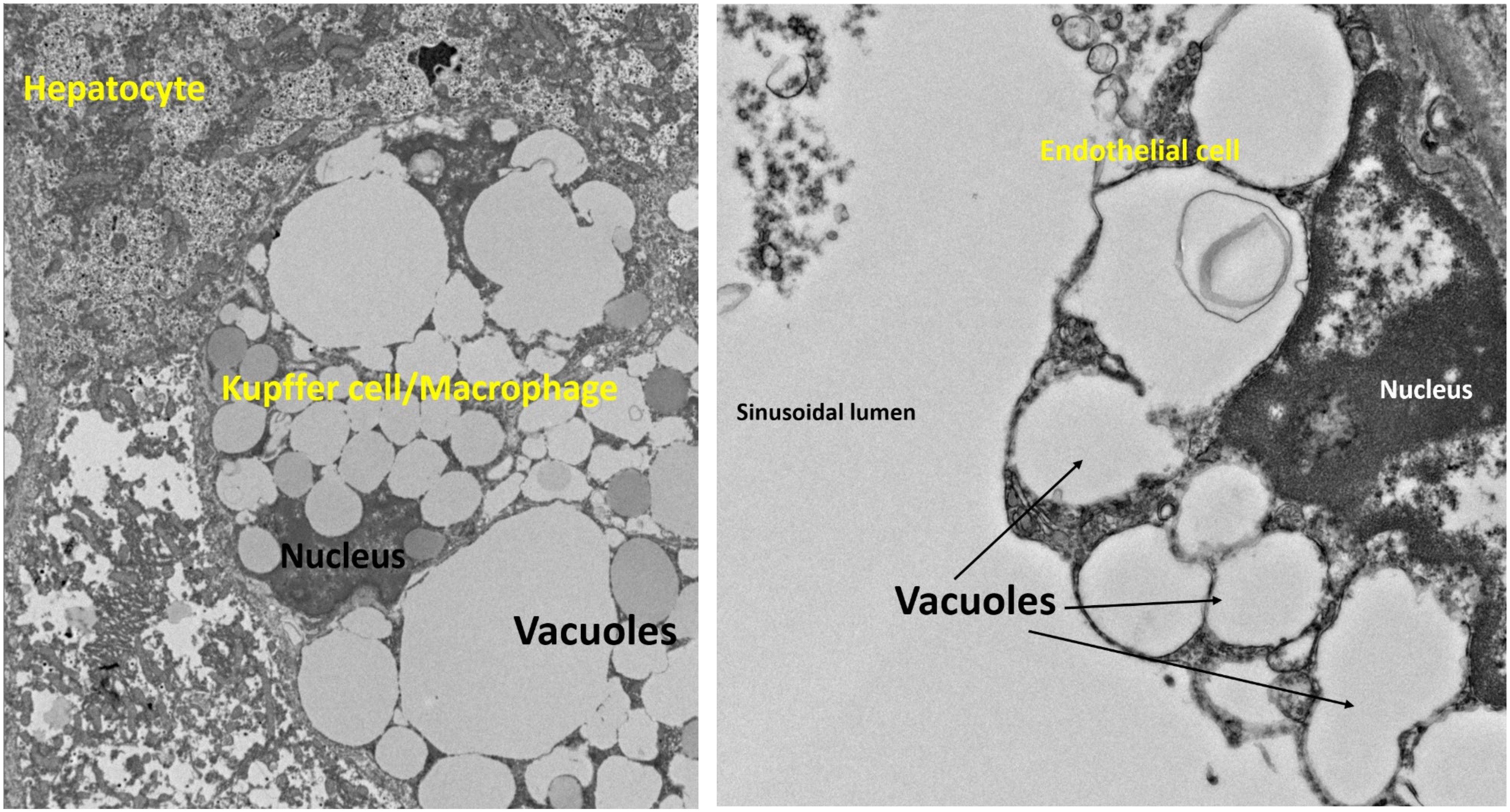

APPENDIX

Appendix A. Molecular Histopathology Laboratory Pathology Narrative (ADME-Tox 193)

Investigator: Dr. Stern
Zap Sty: NCL ADME TOX 193 IND Multi-Dose Toxicity Study in CD-1 Mice

METHODS
CD-1 mice acclimated for a week prior to study initiation. Animals were randomized based on body weight. Treatment (i.v. tail vein, qd x 4; 4 total injections) was initiated by intravenous tail vein injection. Animal behavior was monitored daily and body weights were measured every alternate day (M, W, and F). Urine and feces were collected at 24 hr interval following the final dose for both main and recovery groups. Animals in the main groups were terminated on study at day 5, 24 hr post last dose. Animals in the recovery groups were terminated on study at day 18, 14 days post last dose. All animals were submitted to MH for euthanasia by CO_2 asphyxiation and pathology evaluation. Comprehensive necropsies including gross evaluation of organ systems were performed. Blood was taken for complete blood counts (CBC), blood smear preparation, and serum clinical chemistry.

RESULTS
All the mice, except one mouse in group M05, survive for the duration of the study. Animals euthanized by CO_2 asphyxiation and sent to MHL for evaluation.

Heart, kidney, liver, brain, lung, and spleen were weighed for each mouse. There is a slight increase in body weight, liver weight, and spleen weight in treated recovery groups (group 04 and 06).

There is a mild leukocytosis with neutrophilia in treated recovery groups (M04 and M06) and less severe neutrophilia in the main groups (M03 and M05). Decreases in total protein and albumin are observed in main treated groups (M03 and M05). ALP also decreases in all treated mice compared to controls, which may suggest mild liver dysfunction. No other significant changes on serum chemistry are observed among groups.

All treated mice in all four groups developed similar changes which characterized by multisystemic histiocytosis. The histiocytic infiltrate ranges from mild to severe and are present in all organs and dose dependent. F4/80 IHC was performed on heart and kidney to confirm the diagnosis of histiocytosis. Immunohistochemistry (Anti-F4/80) also performed on liver sections from saline, M03, and M04 groups. IHC slides were scanned analyzed using HALO cytonuclear algorithm. Multiple mice in control and treated groups developed lung inflammation and vasculitis. The lung lesions are related to presence of foreign materials (hair shaft) which may be caused by repeated injections via tail vein. Benign proliferative adrenal lesions were also present in two mice in M01 and M03 group which is considered CD-1 background lesions.

Electron-microscopic examination of liver saline treated mouse reveals presence of normal hepatocytes, macrophages, and endothelial cells. Macrophages and endothelial cells lining sinusoidal spaces in M04 treated mice show presence of intracytoplasmic variably sized round vacuoles that are mostly electron-lucent.

SUMMARY

This is an interesting study. All the treated mice developed multisystemic histiocytosis. H&E and IHC examination revealed that the high dose treated mice in recovery group (M04) have more histocyte infiltrate than the other groups. Electron-microscopic examination shows presence of variable electron-density vacuoles in macrophages and endothelial cells lining sinusoidal spaces. Mild neutrophilia was also observed in treated mice. The data show that there is an increase in the liver and spleen weight in treated recovery groups which may be related to severe histiocytosis, in the absence of other lesions. Mild liver dysfunction was noticed only in treated main groups.

____

Baktiar O. Karim, DVM, PhD
Senior Pathologist

____

Albert Jeon, DVM, PhD
Pathology Fellow

Pathologist Report, Figure 1. Body and Organ Weights. Mean body weight and the percent of body weight of liver, kidney, lungs, heart, spleen and brain by treatment group in CD-1 mice (n = 48).

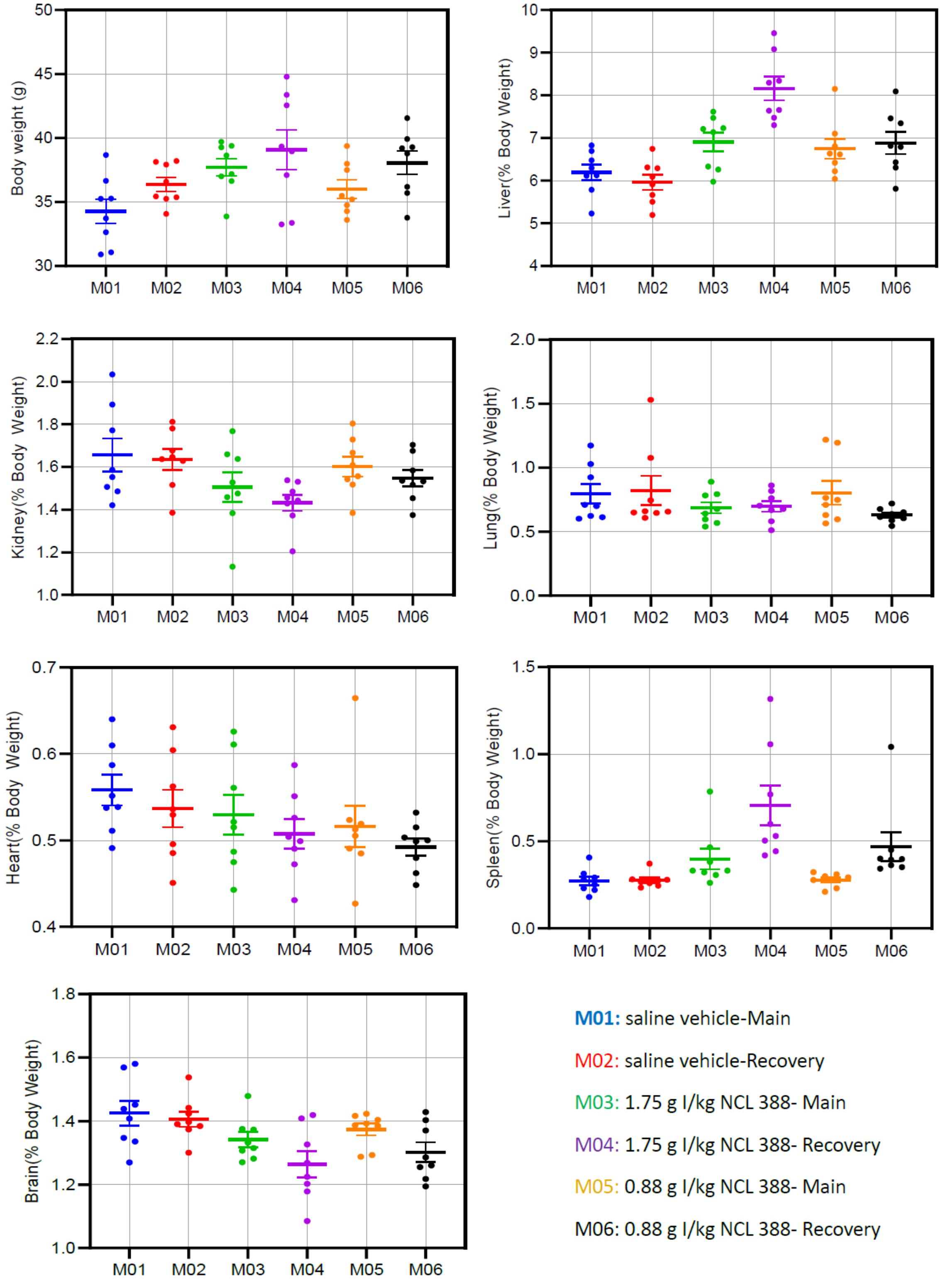

Pathologist Report, Figure 2. Hematology. Complete blood count. Bars represent the means and standard errors of the means within treatment groups.

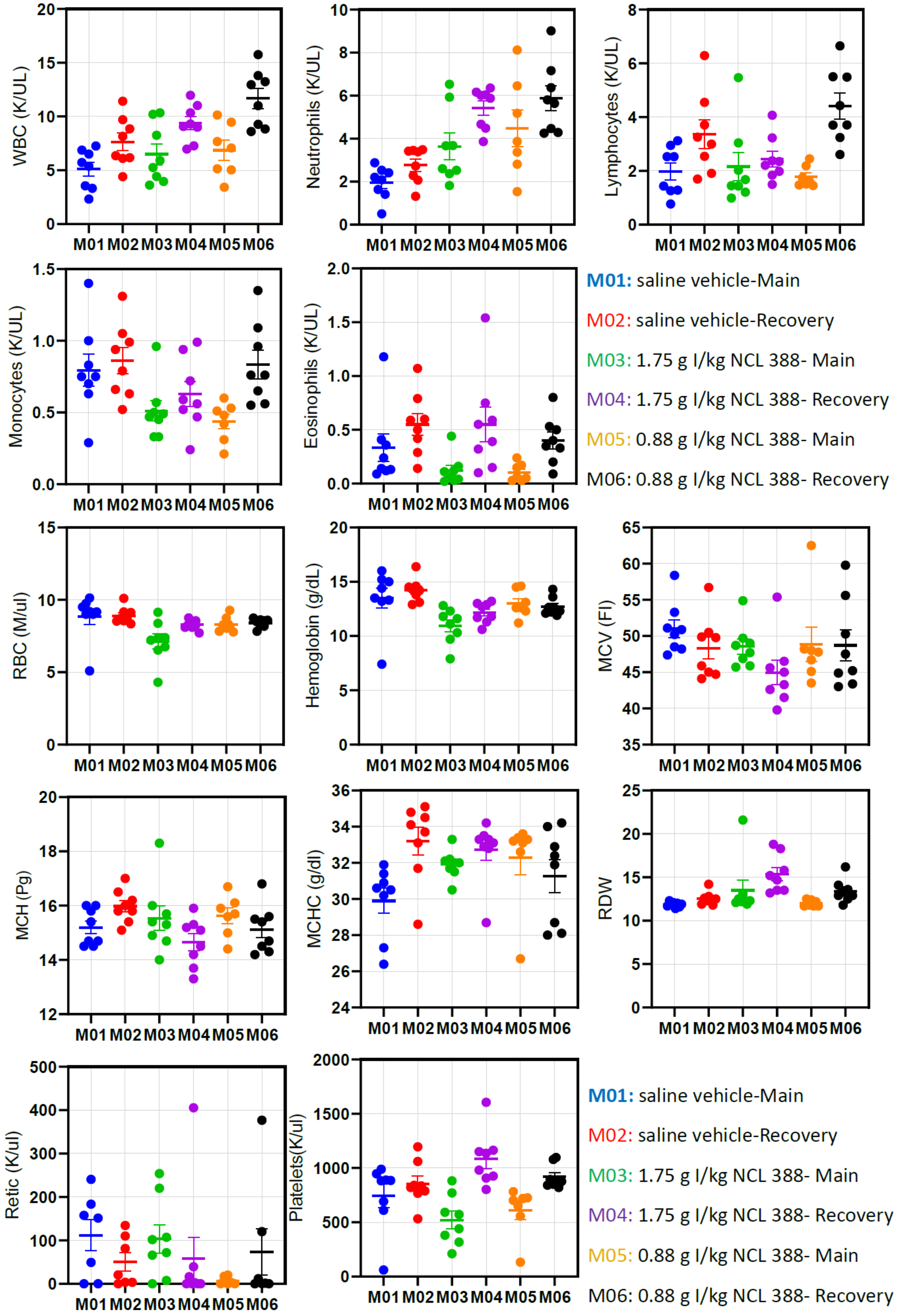

Pathologist Report, Figure 3. Serum Chemistry. Serum chemistry result. Bars represent the means and standard errors of the means within treatment groups.

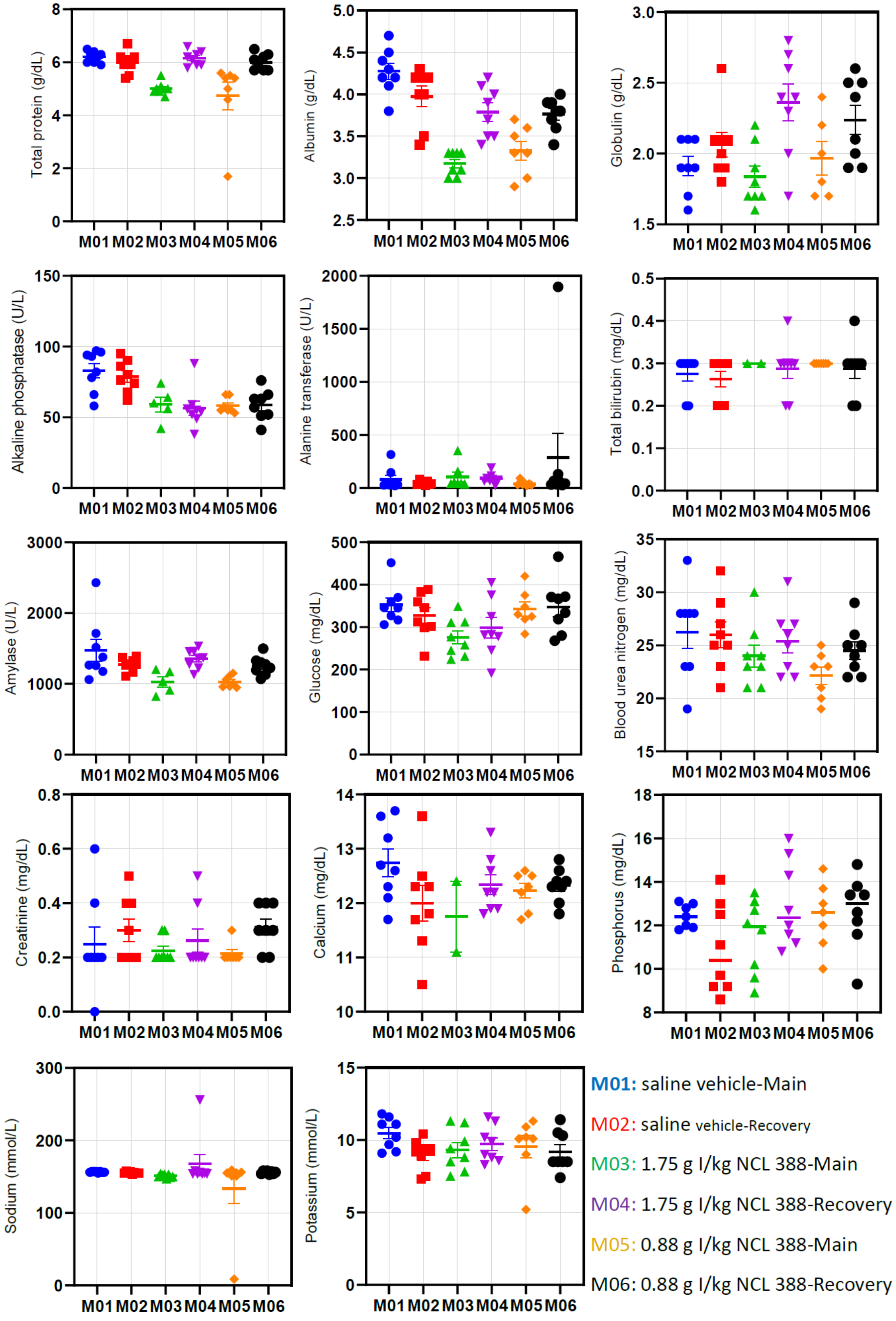

Pathologist Report, Figure 4. Histopathological Results. Representative images of H&E liver sections showing presence of large number of macrophages in treated mice (arrows).

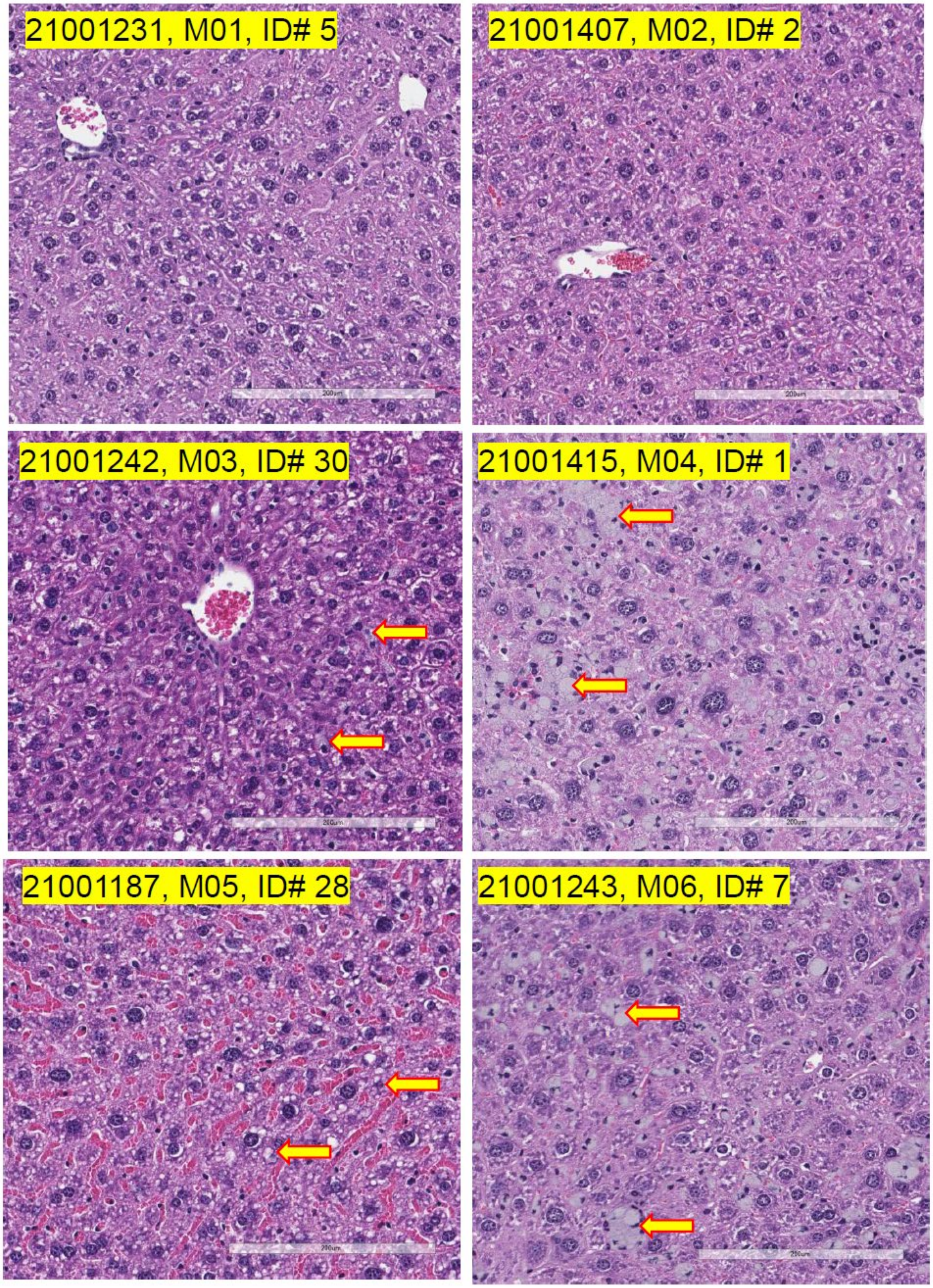

Pathologist Report, Figure 5. Molecular Pathology. Top: Severity of histiocytic infiltrate in saline, main (M03), and recovery (M04) group. Bottom: Representative image from saline (top) and recovery group, M04 (top).

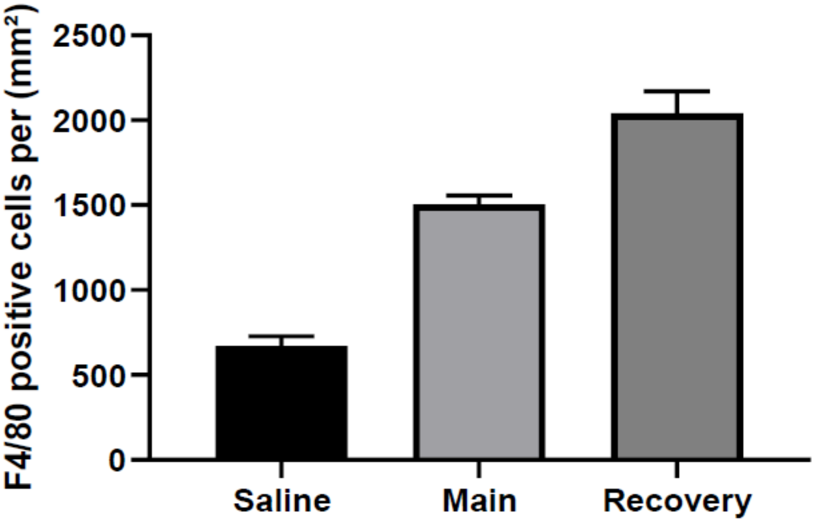

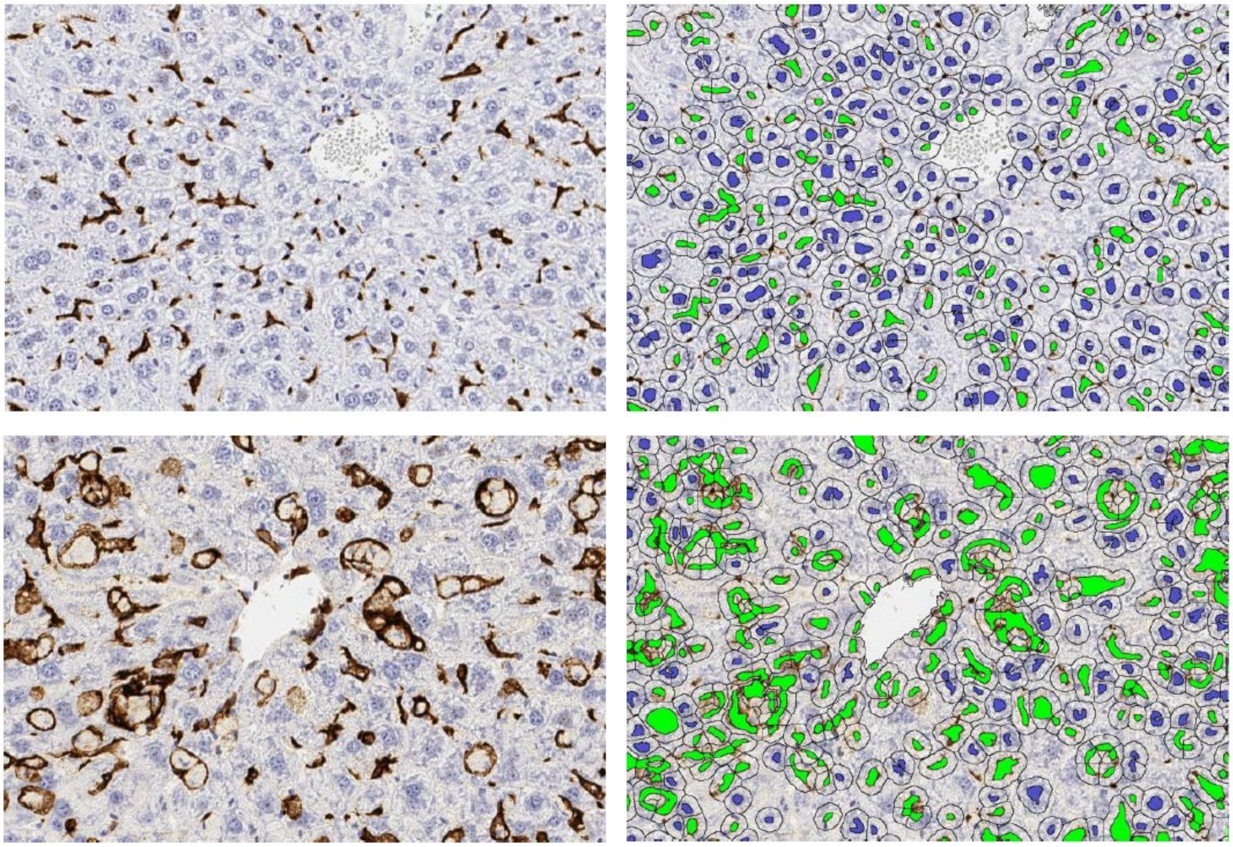

Pathologist Report, Figure 6. Molecular Pathology. Sections of mouse liver from saline and treated recovery (M04). Note presence of variable electron-density vacuoles in macrophages and endothelial cells.

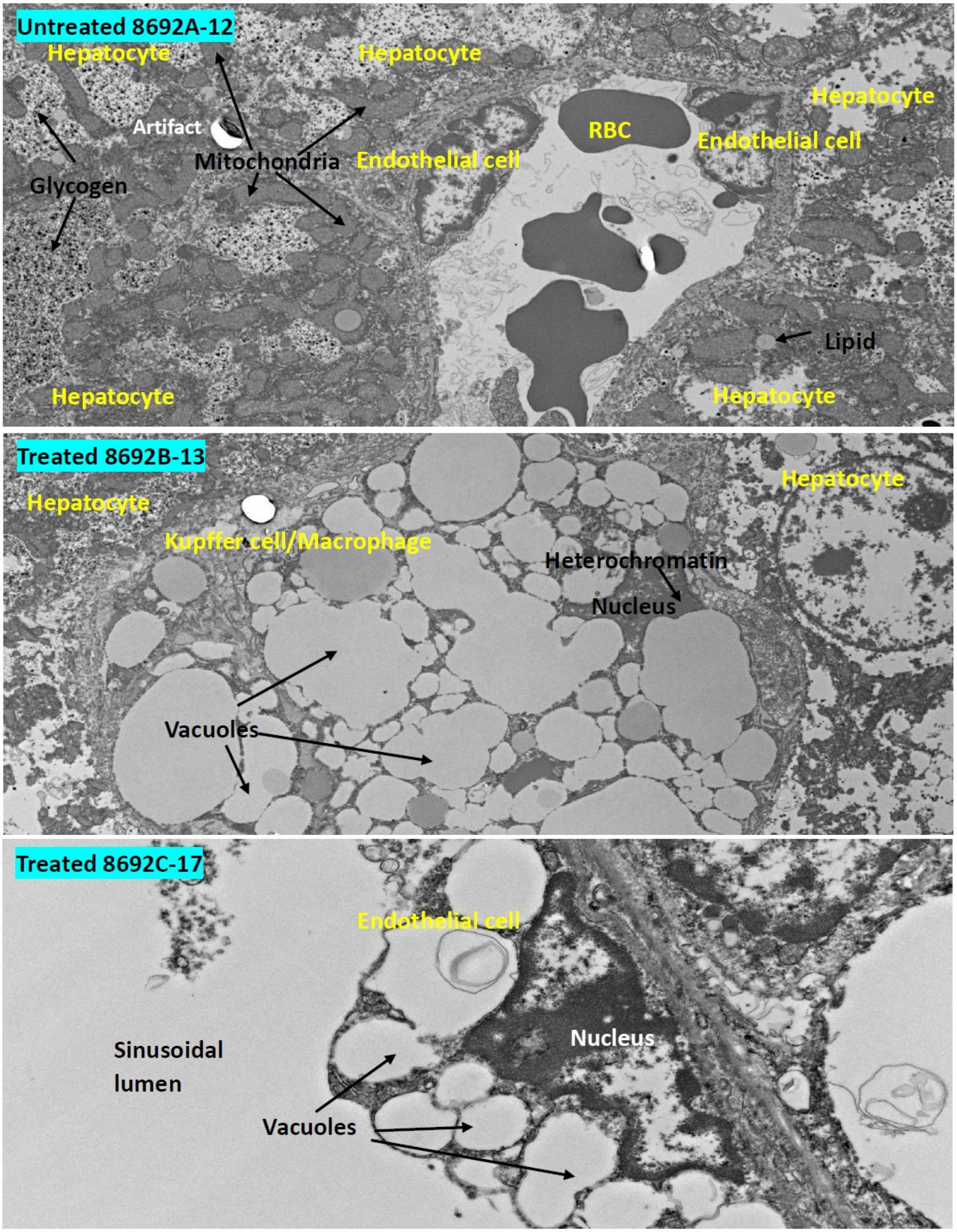

Pathologist Report, Figure 7. Individual Fate Data Report

Group	PHL No.	Animal No.	Age	TOT	Fate	Organ	HN	Neo
**M01**	**Saline-Main**							
	21001231	5	59	4	SS	Euthanasia		
	21001232	8	59	4	SS	Euthanasia		
	21001233	10	59	4	SS	Euthanasia		1
	21001234	18	59	4	SS	Euthanasia		
	21001235	27	59	4	SS	Euthanasia		
	21001236	38	59	4	SS	Euthanasia		
	21001237	40	59	4	SS	Euthanasia		
	21001238	41	59	4	SS	Euthanasia		
	**Mice: 8**	**Average:**	**59**	**4**				
**M02**	**Saline-Recovery**							
	21001407	2	72	17	SS	Euthanasia		
	21001408	3	72	17	SS	Euthanasia		
	21001409	15	72	17	SS	Euthanasia		
	21001410	16	72	17	SS	Euthanasia		
	21001411	19	72	17	SS	Euthanasia		
	21001412	20	72	17	SS	Euthanasia		
	21001413	24	72	17	SS	Euthanasia		
	21001414	32	72	17	SS	Euthanasia		
	**Mice: 8**	**Average:**	**72**	**17**				
**M03**	**545 mg I/kg - Main**							
	21001239	4	59	4	SS	Euthanasia		
	21001240	12	59	4	SS	Euthanasia		
	21001241	13	59	4	SS	Euthanasia		
	21001242	30	59	4	SS	Euthanasia		
	21001243	31	59	4	SS	Euthanasia		
	21001244	35	59	4	SS	Euthanasia		1
	21001245	36	59	4	SS	Euthanasia		
	21001246	42	59	4	SS	Euthanasia		
	**Mice: 8**	**Average:**	**59**	**4**				
**M04**	**545 mg I/kg - Recovery**							
	21001415	1	72	17	SS	Euthanasia		
	21001416	14	72	17	SS	Euthanasia		
	21001417	21	72	17	SS	Euthanasia		
	21001418	44	72	17	SS	Euthanasia		

	21001419	46	72	17	SS	Euthanasia		
	21001420	49	72	17	SS	Euthanasia		
	21001421	51	72	17	SS	Euthanasia		
	21001422	52	72	17	SS	Euthanasia		
	**Mice: 8**	**Average:**	**72**	**17**				
**M05**	**272.5 mg I/kg - Main**							
	21001187	28	56	1	FD	DO NOT USE		
	21001247	6	59	4	SS	Euthanasia		
	21001248	11	59	4	SS	Euthanasia		
	21001249	25	59	4	SS	Euthanasia		
	21001250	26	59	4	SS	Euthanasia		
	21001251	43	59	4	SS	Euthanasia		
	21001252	47	59	4	SS	Euthanasia		
	21001253	50	59	4	SS	Euthanasia		
	**Mice: 8**	**Average:**	**59**	**4**				
**M06**	**272.5 mg I/kg - Recovery**							
	21001423	7	72	17	SS	Euthanasia		
	21001424	17	72	17	SS	Euthanasia		
	21001425	22	72	17	SS	Euthanasia		
	21001426	23	72	17	SS	Euthanasia		
	21001427	33	72	17	SS	Euthanasia		
	21001428	37	72	17	SS	Euthanasia		
	21001429	39	72	17	SS	Euthanasia		
	21001430	45	72	17	SS	Euthanasia		
	**Mice: 8**	**Average:**	**72**	**17**				

**Code**	**Fate**
CS	Clinical Sacrifice
ES	End of Study
FD	Found Dead
IS	Interim Sacrifice
SR	Slides Received
SS	Scheduled Sacrifice
WSS	Wet Tiss Rec - Sched Sac
WTR	Wet Tissues Received

Selection
Groups: M01, M02, M03, M04, M05, M06

Appendix B. Individual Animal Data (ADME-Tox 193)

Table B-1a. Group 1 Hematology Parameters.

Treatment		Saline - Main									
N		8									
Animal ID		5	8	10	18	27	38	40	41	**AVG**	**STDEV**
**WBC**	(K/µL)	6.50	7.26	6.88	3.56	5.38	2.32	5.72	3.32	**5.12**	**1.83**
NE	(K/µL)	2.41	2.88	2.53	1.41	2.20	0.50	2.09	1.64	**1.96**	**0.75**
LY	(K/µL)	2.53	3.12	2.95	1.26	1.28	1.44	2.53	0.77	**1.99**	**0.90**
MO	(K/µL)	1.40	0.75	1.00	0.75	0.63	0.29	0.83	0.70	**0.79**	**0.32**
EO	(K/µL)	0.13	0.41	0.36	0.12	1.18	0.09	0.24	0.14	**0.33**	**0.36**
BA	(K/µL)	0.02	0.10	0.04	0.02	0.08	0.01	0.04	0.06	**0.05**	**0.03**
NE	%	37.06	39.65	36.77	39.67	40.90	21.41	36.53	49.53	**37.69**	**7.80**
LY	%	38.94	42.95	42.87	35.33	23.88	61.98	44.19	23.18	**39.17**	**12.41**
MO	%	21.61	10.32	14.48	20.98	11.72	12.34	14.50	21.14	**15.89**	**4.65**
EO	%	2.04	5.67	5.23	3.50	22.01	3.94	4.12	4.29	**6.35**	**6.42**
BA	%	0.35	1.42	0.64	0.52	1.49	0.34	0.65	1.86	**0.91**	**0.59**
**Erythrocytes:**											
RBC	(M/uL)	9.75	9.17	9.01	10.13	9.20	5.09	9.00	9.50	**8.86**	**1.57**
Hb	(g/dL)	15.00	13.30	14.40	16.00	13.50	7.40	13.20	15.20	**13.50**	**2.66**
HCT	%	56.90	43.50	45.20	51.80	44.30	27.10	43.70	48.40	**45.11**	**8.65**
MCV	(fL)	58.40	47.40	50.20	51.10	48.20	53.30	48.50	50.90	**51.00**	**3.54**
MCH	(pg)	15.40	14.50	16.00	15.80	14.70	14.50	14.70	16.00	**15.20**	**0.67**
MCHC	(g/dL)	26.40	30.60	31.90	30.90	30.50	27.30	30.20	31.40	**29.90**	**1.97**
RDW	%	12.00	11.90	11.60	12.00	11.40	11.70	12.30	12.10	**11.88**	**0.29**
**Thrombocytes:**											
PLT	(K/mL)	610.00	693.00	889.00	886.00	883.00	60.00	989.00	947.00	**744.63**	**304.76**
MPV	(fL)	6.60	6.60	6.80	6.80	6.50	7.20	6.50	6.70	**6.71**	**0.23**

Table B-1b. Group 2 Hematology Parameters.

Treatment		Saline - Recovery									
Number of Animals		8									
Animal ID		2	3	15	16	19	20	24	32	**AVG**	**STDEV**
**WBC**	(K/µL)	6.18	8.86	11.42	6.10	9.70	8.16	6.36	4.40	**7.65**	**2.30**
NE	(K/µL)	2.07	3.49	3.42	2.29	3.39	3.45	2.73	1.32	**2.77**	**0.81**
LY	(K/µL)	3.00	3.71	6.29	2.54	4.55	3.26	1.70	1.91	**3.37**	**1.50**
MO	(K/µL)	0.94	0.66	1.31	0.63	1.05	0.99	0.79	0.52	**0.86**	**0.26**
EO	(K/µL)	0.14	0.79	0.29	0.60	0.50	0.42	1.07	0.59	**0.55**	**0.29**
BA	(K/µL)	0.03	0.22	0.11	0.03	0.21	0.05	0.07	0.05	**0.10**	**0.08**
NE	%	33.52	39.40	29.95	37.58	34.92	42.24	42.90	30.03	**36.32**	**5.06**
LY	%	48.51	41.84	55.07	41.71	46.94	39.96	26.68	43.43	**43.02**	**8.20**
MO	%	15.23	7.40	11.49	10.37	10.84	12.12	12.47	11.91	**11.48**	**2.20**
EO	%	2.32	8.93	2.56	9.82	5.12	5.09	16.80	13.44	**8.01**	**5.21**
BA	%	0.42	2.44	0.93	0.51	2.17	0.59	1.15	1.19	**1.18**	**0.76**
**Erythrocytes:**											
RBC	(M/uL)	9.13	8.71	8.68	8.56	8.35	10.11	8.53	9.17	**8.91**	**0.56**
Hb	(g/dL)	14.60	13.80	14.30	12.90	14.20	16.40	13.10	14.50	**14.23**	**1.08**
HCT	%	46.10	40.00	43.20	38.30	41.70	57.30	37.60	41.30	**43.19**	**6.31**
MCV	(fL)	50.50	45.90	49.80	44.70	49.90	56.70	44.10	45.00	**48.33**	**4.27**
MCH	(pg)	16.00	15.80	16.50	15.10	17.00	16.20	15.40	15.80	**15.98**	**0.60**
MCHC	(g/dL)	31.70	34.50	33.10	33.70	34.10	28.60	34.80	35.10	**33.20**	**2.15**
RDW	%	11.80	12.50	11.90	12.50	12.30	14.20	12.60	12.80	**12.58**	**0.74**
**Thrombocytes:**											
PLT	(K/mL)	840.00	768.00	1060.00	835.00	789.00	1195.00	533.00	820.00	**855.00**	**198.24**
MPV	(fL)	6.70	6.80	6.80	6.40	6.60	6.70	6.50	6.50	**6.63**	**0.15**

Table B-1c. Group 3 Hematology Parameters.

Treatment		NCL388-12, 1715 mg I/kg - Main									
Number of Animals		8									
Animal ID		4	12	13	30	31	35	36	42	**AVG**	**STDEV**
**WBC**	(K/µL)	5.90	10.20	3.96	3.62	5.26	10.34	4.42	8.26	**6.50**	**2.74**
NE	(K/µL)	3.68	5.93	2.53	1.82	2.60	3.67	2.37	6.53	**3.64**	**1.73**
LY	(K/µL)	1.68	3.04	0.99	1.45	2.02	5.47	1.44	1.21	**2.16**	**1.48**
MO	(K/µL)	0.49	0.57	0.33	0.33	0.50	0.96	0.47	0.45	**0.51**	**0.20**
EO	(K/µL)	0.03	0.44	0.06	0.02	0.12	0.16	0.11	0.04	**0.12**	**0.14**
BA	(K/µL)	0.02	0.23	0.05	0.00	0.03	0.08	0.03	0.02	**0.06**	**0.07**
NE	%	62.35	58.10	63.86	50.27	49.50	35.54	53.59	79.09	**56.54**	**12.74**
LY	%	28.53	29.78	24.98	40.10	38.37	52.94	32.49	14.64	**32.73**	**11.39**
MO	%	8.30	5.61	8.40	9.06	9.45	9.24	10.67	5.49	**8.28**	**1.83**
EO	%	0.51	4.28	1.59	0.56	2.19	1.55	2.50	0.53	**1.71**	**1.29**
BA	%	0.30	2.23	1.17	0.00	0.49	0.74	0.75	0.26	**0.74**	**0.70**
**Erythrocytes:**											
RBC	(M/uL)	9.14	7.22	7.37	7.39	4.31	6.75	8.38	6.53	**7.14**	**1.42**
Hb	(g/dL)	12.80	11.10	11.80	11.60	7.90	10.30	12.30	9.70	**10.94**	**1.59**
HCT	%	42.00	35.20	36.60	36.20	23.70	32.20	38.30	30.60	**34.35**	**5.55**
MCV	(fL)	45.90	48.70	49.70	49.00	54.90	47.70	45.70	46.90	**48.56**	**2.94**
MCH	(pg)	14.00	15.40	16.00	15.70	18.30	15.30	14.70	14.90	**15.54**	**1.28**
MCHC	(g/dL)	30.50	31.50	32.20	32.00	33.30	32.00	32.10	31.70	**31.91**	**0.78**
RDW	%	11.90	12.30	12.40	12.50	21.60	12.10	12.20	13.10	**13.51**	**3.29**
**Thrombocytes:**											
PLT	(K/mL)	883.00	317.00	380.00	441.00	771.00	211.00	593.00	572.00	**521.00**	**228.45**
MPV	(fL)	7.10	7.50	7.50	7.30	7.30	7.50	7.10	7.40	**7.34**	**0.17**

Table B-1d. Group 4 Hematology Parameters.

Treatment		NCL388-12, 1715 mg I/kg - Recovery									
Number of Animals		8									
Animal ID		1	14	21	44	46	49	51	52	**AVG**	**STDEV**
**WBC**	(K/µL)	9.30	9.00	10.34	7.28	6.98	11.98	11.02	9.08	**9.37**	**1.72**
NE	(K/µL)	5.88	4.49	6.01	4.67	3.86	6.04	6.35	6.16	**5.43**	**0.94**
LY	(K/µL)	2.38	2.35	1.50	1.98	1.84	4.07	3.23	2.21	**2.45**	**0.83**
MO	(K/µL)	0.56	0.94	0.24	0.47	0.52	0.99	0.72	0.59	**0.63**	**0.25**
EO	(K/µL)	0.32	0.75	1.54	0.15	0.55	0.59	0.39	0.10	**0.55**	**0.46**
BA	(K/µL)	0.16	0.47	1.05	0.01	0.22	0.28	0.34	0.02	**0.32**	**0.33**
NE	%	63.22	49.88	58.11	64.14	55.28	50.40	57.66	67.84	**58.32**	**6.46**
LY	%	25.63	26.06	14.50	27.23	26.33	34.01	29.28	24.33	**25.92**	**5.50**
MO	%	5.99	10.43	2.32	6.45	7.46	8.29	6.50	6.50	**6.74**	**2.29**
EO	%	3.43	8.35	14.90	2.00	7.82	4.93	3.52	1.10	**5.76**	**4.50**
BA	%	1.72	5.27	10.16	0.19	3.11	2.36	3.04	0.23	**3.26**	**3.24**
**Erythrocytes:**											
RBC	(M/uL)	7.72	8.31	8.11	8.11	8.52	8.76	8.25	8.56	**8.29**	**0.32**
Hb	(g/dL)	10.60	12.70	11.80	12.90	11.30	13.20	11.70	13.00	**12.15**	**0.94**
HCT	%	32.00	38.60	34.50	44.90	33.90	39.40	35.70	39.00	**37.25**	**4.08**
MCV	(fL)	41.50	46.50	42.60	55.40	39.80	45.00	43.30	45.60	**44.96**	**4.76**
MCH	(pg)	13.70	15.30	14.50	15.90	13.30	15.10	14.20	15.20	**14.65**	**0.88**
MCHC	(g/dL)	33.10	32.90	34.20	28.70	33.30	33.50	32.80	33.30	**32.73**	**1.68**
RDW	%	18.80	14.70	13.50	15.20	18.30	13.20	15.80	13.50	**15.38**	**2.16**
**Thrombocytes:**											
PLT	(K/mL)	907.00	1164.00	1135.00	981.00	1606.00	802.00	1150.00	926.00	**1083.88**	**248.50**
MPV	(fL)	7.10	7.10	7.00	6.70	6.90	7.20	7.10	7.10	**7.03**	**0.16**

Table B-1e. Group 5 Hematology Parameters.

Treatment		NCL388-12, 855 mg I/kg - Main								
Number of Animals		8								
Animal ID		6	11	25	26	43	47	50	**AVG**	**STDEV**
**WBC**	(K/µL)	7.08	5.14	9.48	3.42	5.04	7.68	10.14	**6.85**	**2.46**
NE	(K/µL)	3.87	3.37	6.45	1.54	2.81	5.18	8.12	**4.48**	**2.26**
LY	(K/µL)	2.19	1.48	2.45	1.51	1.61	1.74	1.46	**1.78**	**0.39**
MO	(K/µL)	0.60	0.21	0.51	0.31	0.42	0.53	0.48	**0.44**	**0.14**
EO	(K/µL)	0.24	0.05	0.03	0.03	0.15	0.17	0.06	**0.10**	**0.08**
BA	(K/µL)	0.17	0.02	0.04	0.03	0.05	0.06	0.02	**0.06**	**0.05**
NE	%	54.72	65.62	68.09	45.03	55.69	67.46	80.10	**62.39**	**11.46**
LY	%	30.97	28.86	25.81	44.05	31.98	22.63	14.41	**28.39**	**9.13**
MO	%	8.48	4.09	5.39	9.18	8.38	6.89	4.71	**6.73**	**2.03**
EO	%	3.45	1.05	0.29	0.93	2.95	2.21	0.61	**1.64**	**1.23**
BA	%	2.37	0.38	0.42	0.81	0.98	0.81	0.18	**0.85**	**0.73**
**Erythrocytes:**										
RBC	(M/uL)	8.04	8.74	7.83	7.78	8.23	8.20	9.28	**8.30**	**0.54**
Hb	(g/dL)	12.60	14.60	12.60	11.20	13.10	12.30	14.50	**12.99**	**1.22**
HCT	%	37.50	54.60	37.70	33.80	39.30	37.00	44.50	**40.63**	**6.95**
MCV	(fL)	46.70	62.50	48.20	43.50	47.80	45.10	48.00	**48.83**	**6.27**
MCH	(pg)	15.70	16.70	16.10	14.40	15.90	15.00	15.60	**15.63**	**0.75**
MCHC	(g/dL)	33.60	26.70	33.40	33.10	33.30	33.20	32.60	**32.27**	**2.48**
RDW	%	12.40	11.70	11.70	11.70	11.80	12.20	12.50	**12.00**	**0.36**
**Thrombocytes:**										
PLT	(K/mL)	719.00	132.00	703.00	557.00	652.00	782.00	726.00	**610.14**	**222.35**
MPV	(fL)	6.70	6.90	7.10	6.80	7.30	6.90	7.00	**6.96**	**0.20**

Table B-1f. Group 6 Hematology Parameters.

Treatment		NCL388-12, 855 mg I/kg - Recovery									
Number of Animals		8									
Animal ID		7	17	22	23	33	37	39	45	**AVG**	**STDEV**
**WBC**	(K/µL)	8.60	12.96	8.84	13.24	9.28	13.80	15.76	11.00	**11.69**	**2.65**
NE	(K/µL)	4.25	5.80	4.29	6.37	4.47	9.01	7.16	5.64	**5.87**	**1.64**
LY	(K/µL)	2.61	5.50	3.70	5.49	3.71	3.24	6.65	4.43	**4.42**	**1.36**
MO	(K/µL)	0.55	1.09	0.56	0.96	0.65	0.76	1.35	0.76	**0.84**	**0.28**
EO	(K/µL)	0.80	0.40	0.20	0.35	0.33	0.53	0.50	0.09	**0.40**	**0.22**
BA	(K/µL)	0.39	0.17	0.08	0.06	0.13	0.26	0.10	0.08	**0.16**	**0.11**
NE	%	49.39	44.77	48.51	48.14	48.18	65.30	45.45	51.25	**50.12**	**6.47**
LY	%	30.30	42.44	41.89	41.48	39.95	23.47	42.18	40.26	**37.75**	**7.00**
MO	%	6.42	8.38	6.34	7.23	6.97	5.54	8.58	6.92	**7.05**	**1.02**
EO	%	9.35	3.07	2.30	2.67	3.53	3.84	3.16	0.84	**3.60**	**2.50**
BA	%	4.55	1.34	0.95	0.47	1.38	1.85	0.62	0.73	**1.49**	**1.32**
**Erythrocytes:**											
RBC	(M/uL)	8.74	8.59	8.18	8.49	7.83	8.23	8.55	8.62	**8.40**	**0.30**
Hb	(g/dL)	13.60	12.30	12.60	14.30	12.10	11.90	12.60	12.20	**12.70**	**0.83**
HCT	%	48.60	38.60	38.90	50.80	35.40	41.40	37.10	37.10	**40.99**	**5.68**
MCV	(fL)	55.60	44.90	47.50	59.80	45.20	50.30	43.40	43.00	**48.71**	**6.12**
MCH	(pg)	15.60	14.30	15.40	16.80	15.50	14.50	14.70	14.20	**15.13**	**0.87**
MCHC	(g/dL)	28.00	31.90	32.40	28.10	34.20	28.70	34.00	32.90	**31.28**	**2.61**
RDW	%	13.30	12.90	12.50	14.10	13.60	16.20	12.90	11.80	**13.41**	**1.32**
**Thrombocytes:**											
PLT	(K/mL)	894.00	1079.00	877.00	888.00	1098.00	851.00	843.00	820.00	**918.75**	**107.70**
MPV	(fL)	6.70	6.80	6.50	6.80	6.80	7.10	6.60	6.50	**6.73**	**0.20**

Table B-2a. Group 1 Clinical Chemistry Parameters.

Treatment		Saline - Main									
Number of Animals		8									
Animal ID		5	8	10	18	27	38	40	41	**AVG**	**STDEV**
**Electrolyte balance**											
Calcium	mg/dL	12.6	13.2	12.7	13.6	12.3	11.7	12.1	13.7	**12.7**	**0.7**
Phosphate	mg/dL	11.8	12.4	11.9	n/a	13.0	12.0	12.8	13.1	**12.4**	**0.5**
Potassium	mmol/L	11.1	11.6	10.2	11.8	9.7	9.1	9.2	11.1	**10.5**	**1.1**
Sodium	mmol/L	157.0	157.0	155.0	157.0	156.0	156.0	157.0	156.0	**156.4**	**0.7**
**Carbohydrate metabolism**											
Glucose	mg/dL	360.0	452.0	345.0	370.0	346.0	306.0	317.0	327.0	**352.9**	**45.4**
**Pancreatic function**											
Amylase	u/L	1713.0	1267.0	1516.0	1175.0	1259.0	1379.0	1061.0	2432.0	**1475.3**	**436.7**
**Liver function: A) hepatobiliary**											
Total Bilirubin	mg/dL	0.3	0.2	0.3	0.3	0.3	0.3	0.3	0.2	**0.3**	**0.0**
**Liver function: B) hepatocellular**											
Alanine Aminotransferase	u/L	29.0	315.0	42.0	35.0	27.0	146.0	33.0	21.0	**81.0**	**102.9**
**Kidney function**											
Creatinine	mg/dL	0.6	0.2	0.2	0.2	0.4	0.0	0.2	0.2	**0.3**	**0.2**
Urea Nitrogen	mg/dL	23.0	28.0	33.0	28.0	23.0	19.0	28.0	28.0	**26.3**	**4.3**
**Others**											
Albumin (A)	g/dL	4.1	4.3	4.4	4.7	3.8	4.2	4.2	4.5	**4.3**	**0.3**
Alkaline Phosphatase	u/L	82.0	66.0	78.0	97.0	93.0	94.0	96.0	58.0	**83.0**	**14.8**
Globulin (G)	g/dL	1.9	2.1	1.9	1.6	2.1	1.7	1.9	2.1	**1.9**	**0.2**
A/G Ratio		2.2	2.0	2.3	2.9	1.8	2.5	2.2	2.1	**2.3**	**0.3**
Total Protein	g/dL	6.0	6.4	6.3	6.3	5.9	6.0	6.2	6.5	**6.2**	**0.2**

Table B-2b. Group 2 Clinical Chemistry Parameters.

Treatment		Saline - Recovery									
Number of Animals		8									
Animal ID		0	0	0	0	0	0	0	0	**AVG**	**STDEV**
**Electrolyte balance**											
Calcium	mg/dL	11.8	12.5	11.7	10.5	12.3	13.6	11.3	12.3	**12.0**	**0.9**
Phosphate	mg/dL	9.7	9.2	9.2	8.6	13.0	14.1	12.5	11.1	**10.9**	**2.1**
Potassium	mmol/L	9.8	9.2	7.3	7.5	8.9	9.4	10.4	9.4	**9.0**	**1.1**
Sodium	mmol/L	155.0	158.0	157.0	156.0	155.0	156.0	155.0	153.0	**155.6**	**1.5**
**Carbohydrate metabolism**											
Glucose	mg/dL	360.0	388.0	298.0	232.0	346.0	383.0	302.0	312.0	**327.6**	**52.1**
**Pancreatic function**											
Amylase	u/L	1375.0	1272.0	1326.0	1395.0	1267.0	1112.0	1283.0	1169.0	**1274.9**	**96.3**
**Liver function: A) hepatobiliary**											
Total Bilirubin	mg/dL	0.2	0.2	0.3	0.3	0.3	0.2	0.3	0.3	**0.3**	**0.1**
**Liver function: B) hepatocellular**											
Alanine Aminotransferase	u/L	34.0	41.0	26.0	37.0	65.0	82.0	54.0	66.0	**50.6**	**19.3**
**Kidney function**											
Creatinine	mg/dL	0.5	0.2	0.2	0.4	0.2	0.4	0.2	0.3	**0.3**	**0.1**
Urea Nitrogen	mg/dL	25.0	32.0	23.0	21.0	29.0	25.0	27.0	26.0	**26.0**	**3.4**
**Others**											
Albumin (A)	g/dL	4.0	4.3	4.2	3.5	4.0	4.2	3.4	4.2	**4.0**	**0.4**
Alkaline Phosphatase	u/L	62.0	68.0	95.0	76.0	80.0	86.0	90.0	74.0	**78.9**	**11.2**
Globulin (G)	g/dL	2.1	1.9	1.8	1.9	2.0	2.6	2.1	2.1	**2.1**	**0.2**
A/G Ratio		1.9	2.3	2.3	1.8	2.0	1.6	1.6	2.0	**1.9**	**0.3**
Total Protein	g/dL	6.1	6.2	5.9	5.4	5.9	6.7	5.5	6.2	**6.0**	**0.4**

Table B-2c. Group 3 Clinical Chemistry Parameters.

Treatment		NCL388-12, 1715 mg I/kg - Main									
Number of Animals		8									
Animal ID		4	12	13	30	31	35	36	42	**AVG**	**STDEV**
**Electrolyte balance**											
Calcium	mg/dL	12.4	LIP	LIP	LIP	LIP	LIP	LIP	11.1	**11.8**	**0.9**
Phosphate	mg/dL	13.1	13.5	12.1	11.8	8.9	12.7	9.6	10.2	**11.5**	**1.7**
Potassium	mmol/L	11.2	11.3	9.9	9.4	8.8	8.5	7.5	7.8	**9.3**	**1.4**
Sodium	mmol/L	153.0	150.0	150.0	154.0	149.0	147.0	153.0	152.0	**151.0**	**2.4**
**Carbohydrate metabolism**											
Glucose	mg/dL	258.0	349.0	231.0	245.0	312.0	311.0	277.0	224.0	**275.9**	**44.5**
**Pancreatic function**											
Amylase	u/L	1203.0	LIP	1170.0	911.0	824.0	LIP	LIP	1037.0	**1029.0**	**162.9**
**Liver function: A) hepatobiliary**											
Total Bilirubin	mg/dL	0.3	LIP	LIP	LIP	LIP	LIP	LIP	0.3	**0.3**	**0.0**
**Liver function: B) hepatocellular**											
Alanine Aminotransferase	u/L	39.0	157.0	41.0	44.0	n/a	352.0	54.0	42.0	**104.1**	**117.2**
**Kidney function**											
Creatinine	mg/dL	0.3	0.2	0.2	0.2	0.2	0.3	0.2	0.2	**0.2**	**0.0**
Urea Nitrogen	mg/dL	23.0	21.0	30.0	21.0	23.0	24.0	26.0	24.0	**24.0**	**2.9**
**Others**											
Albumin (A)	g/dL	3.3	3.1	3.0	3.3	3.3	3.1	3.3	3.0	**3.2**	**0.1**
Alkaline Phosphatase	u/L	64.0	LIP	74.0	56.0	42.0	LIP	LIP	60.0	**59.2**	**11.7**
Globulin (G)	g/dL	2.2	1.8	1.7	1.7	1.7	1.9	1.6	2.1	**1.8**	**0.2**
A/G Ratio		1.5	1.7	1.8	1.9	1.9	1.6	2.1	1.4	**1.7**	**0.2**
Total Protein	g/dL	5.5	4.9	4.7	5.0	5.0	5.0	4.9	5.1	**5.0**	**0.2**

Table B-2d. Group 4 Clinical Chemistry Parameters.

Treatment		NCL388-12, 1715 mg I/kg - Recovery									
Number of Animals		8									
Animal ID		1	14	21	44	46	49	51	52	**AVG**	**STDEV**
**Electrolyte balance**											
Calcium	mg/dL	11.9	13.3	11.8	12.2	12.8	11.9	12.6	12.2	**12.3**	**0.5**
Phosphate	mg/dL	12.0	16.0	11.2	10.8	15.3	12.7	14.3	11.6	**13.0**	**2.0**
Potassium	mmol/L	8.8	11.6	8.6	8.3	11.3	9.9	10.2	9.0	**9.7**	**1.2**
Sodium	mmol/L	156.0	154.0	155.0	156.0	154.0	154.0	158.0	154.0	**155.1**	**1.5**
**Carbohydrate metabolism**											
Glucose	mg/dL	192.0	405.0	246.0	278.0	376.0	283.0	282.0	325.0	**298.4**	**68.7**
**Pancreatic function**											
Amylase	u/L	1372.0	1456.0	1134.0	1278.0	1462.0	1534.0	1365.0	1227.0	**1353.5**	**133.7**
**Liver function: A) hepatobiliary**											
Total Bilirubin	mg/dL	0.3	0.4	0.2	0.3	0.3	0.3	0.2	0.3	**0.3**	**0.1**
**Liver function: B) hepatocellular**											
Alanine Aminotransferase	u/L	191.0	71.0	84.0	26.0	67.0	92.0	76.0	116.0	**90.4**	**48.0**
**Kidney function**											
Creatinine	mg/dL	0.2	0.5	0.3	0.2	0.4	0.2	0.2	0.2	**0.3**	**0.1**
Urea Nitrogen	mg/dL	23.0	25.0	22.0	31.0	27.0	22.0	27.0	26.0	**25.4**	**3.1**
**Others**											
Albumin (A)	g/dL	3.5	4.2	4.1	3.9	3.7	4.0	3.5	3.4	**3.8**	**0.3**
Alkaline Phosphatase	u/L	38.0	59.0	55.0	52.0	54.0	88.0	49.0	56.0	**56.4**	**14.3**
Globulin (G)	g/dL	2.8	2.4	2.3	2.0	2.7	1.7	2.6	2.4	**2.4**	**0.4**
A/G Ratio		1.3	1.8	1.8	2.0	1.4	2.4	1.3	1.4	**1.7**	**0.4**
Total Protein	g/dL	6.2	6.6	6.4	5.9	6.3	5.8	6.1	5.9	**6.2**	**0.3**

Table B-2e. Group 5 Clinical Chemistry Parameters.

Treatment		NCL388-12, 855 mg I/kg - Main								
Number of Animals		8								
Animal ID		6	11	25	26	43	47	50	**AVG**	**STDEV**
**Electrolyte balance**										
Calcium	mg/dL	11.8	11.7	12.3	12.5	12.2	12.6	12.5	**12.2**	**0.4**
Phosphate	mg/dL	13.0	14.6	12.0	13.7	12.6	10.0	11.2	**12.4**	**1.5**
Potassium	mmol/L	8.8	10.2	9.0	11.3	10.9	10.1	10.1	**10.1**	**0.9**
Sodium	mmol/L	154.0	151.0	155.0	151.0	155.0	156.0	159.0	**154.4**	**2.8**
**Carbohydrate metabolism**										
Glucose	mg/dL	348.0	325.0	375.0	319.0	330.0	420.0	284.0	**343.0**	**43.8**
**Pancreatic function**										
Amylase	u/L	964.0	949.0	1064.0	959.0	1115.0	1150.0	1007.0	**1029.7**	**80.9**
**Liver function: A) hepatobiliary**										
Total Bilirubin	mg/dL	0.3	0.3	0.3	0.3	0.3	0.3	0.3	**0.3**	**0.0**
**Liver function: B) hepatocellular**										
Alanine Aminotransferase	u/L	41.0	92.0	20.0	48.0	32.0	36.0	28.0	**42.4**	**23.6**
**Kidney function**										
Creatinine	mg/dL	0.2	0.2	0.2	0.2	0.2	0.2	0.3	**0.2**	**0.0**
Urea Nitrogen	mg/dL	25.0	23.0	20.0	24.0	21.0	19.0	23.0	**22.1**	**2.2**
**Others**										
Albumin (A)	g/dL	3.5	3.3	2.9	3.7	3.6	3.0	3.3	**3.3**	**0.3**
Alkaline Phosphatase	u/L	66.0	55.0	57.0	55.0	55.0	66.0	53.0	**58.1**	**5.5**
Globulin (G)	g/dL	1.7	1.7	1.7	2.0	1.8	2.4	2.2	**1.9**	**0.3**
A/G Ratio		2.1	1.9	1.7	1.9	2.0	1.3	1.5	**1.8**	**0.3**
Total Protein	g/dL	5.2	5.0	4.6	5.6	5.4	5.4	5.5	**5.2**	**0.3**

Table B-2f. Group 6 Clinical Chemistry Parameters.

Treatment		NCL388-12, 855 mg I/kg - Recovery									
Number of Animals		8									
Animal ID		7	17	22	23	33	37	39	45	**AVG**	**STDEV**
**Electrolyte balance**											
Calcium	mg/dL	12.3	12.3	12.4	12.0	12.4	12.6	12.8	11.8	**12.3**	**0.3**
Phosphate	mg/dL	13.8	14.8	13.4	11.6	13.4	12.2	12.6	9.3	**12.6**	**1.7**
Potassium	mmol/L	10.5	11.4	10.5	7.4	9.4	10.4	9.3	9.8	**9.8**	**1.2**
Sodium	mmol/L	153.0	158.0	154.0	157.0	156.0	156.0	154.0	158.0	**155.8**	**1.9**
**Carbohydrate metabolism**											
Glucose	mg/dL	319.0	466.0	372.0	268.0	367.0	334.0	371.0	280.0	**347.1**	**62.6**
**Pancreatic function**											
Amylase	u/L	1499.0	1328.0	1130.0	1193.0	1226.0	1303.0	1277.0	1070.0	**1253.3**	**132.1**
**Liver function: A) hepatobiliary**											
Total Bilirubin	mg/dL	0.4	0.3	0.3	0.3	0.3	0.2	0.2	0.3	**0.3**	**0.1**
**Liver function: B) hepatocellular**											
Alanine Aminotransferase	u/L	62.0	1896.0	33.0	27.0	43.0	130.0	35.0	68.0	**286.8**	**651.1**
**Kidney function**											
Creatinine	mg/dL	0.3	0.2	0.3	0.4	0.3	0.4	0.2	0.4	**0.3**	**0.1**
Urea Nitrogen	mg/dL	26.0	29.0	22.0	22.0	25.0	23.0	25.0	24.0	**24.5**	**2.3**
**Others**											
Albumin (A)	g/dL	3.9	4.0	3.6	3.9	3.7	3.4	3.8	3.8	**3.8**	**0.2**
Alkaline Phosphatase	u/L	57.0	76.0	66.0	63.0	41.0	51.0	63.0	52.0	**58.6**	**10.8**
Globulin (G)	g/dL	2.4	2.5	2.1	1.9	2.0	2.6	2.5	1.9	**2.2**	**0.3**
A/G Ratio		1.6	1.6	1.7	2.1	1.9	1.3	1.5	2.0	**1.7**	**0.3**
Total Protein	g/dL	6.2	6.5	5.7	5.8	5.7	6.1	6.3	5.7	**6.0**	**0.3**

Table B-3a. Main Study Absolute Body Weight (grams).
(See key following Table B-3b.)

Group	Animal ID	Sex	Fate		10/5/2020	10/7/2020	10/8/2020	10/9/2020
			Code	Day	1	3	4	5
Group 01 Saline Vehicle - Main	05	M	MHL	5	32.00	31.90	32.00	32.10
Group 01 Saline Vehicle - Main	08	M	MHL	5	32.20	32.00	32.00	31.80
Group 01 Saline Vehicle - Main	10	M	MHL	5	34.40	34.60	35.00	35.10
Group 01 Saline Vehicle - Main	18	M	MHL	5	35.60	34.90	35.50	35.30
Group 01 Saline Vehicle - Main	27	M	MHL	5	39.10	38.30	38.00	38.10
Group 01 Saline Vehicle - Main	38	M	MHL	5	34.60	34.10	33.60	33.50
Group 01 Saline Vehicle - Main	40	M	MHL	5	37.60	37.10	37.80	37.40
Group 01 Saline Vehicle - Main	41	M	MHL	5	36.00	36.00	36.00	36.10
Group 03 1715 mg l/kg NCL 388 - Main	04	M	MHL	5	36.80	37.00	39.90	39.60
Group 03 1715 mg l/kg NCL 388 - Main	12	M	MHL	5	35.40	35.50	37.80	37.50
Group 03 1715 mg l/kg NCL 388 - Main	13	M	MHL	5	35.70	35.50	38.70	38.50
Group 03 1715 mg l/kg NCL 388 - Main	30	M	MHL	5	36.00	36.50	39.00	39.00
Group 03 1715 mg l/kg NCL 388 - Main	31	M	MHL	5	31.30	31.60	34.60	34.50
Group 03 1715 mg l/kg NCL 388 - Main	35	M	MHL	5	35.00	36.00	39.30	39.20
Group 03 1715 mg l/kg NCL 388 - Main	36	M	MHL	5	33.80	34.00	37.50	37.20
Group 03 1715 mg l/kg NCL 388 - Main	42	M	MHL	5	33.00	35.00	37.00	37.00
Group 05 855 mg l/kg NCL 388 - Main	06	M	MHL	5	38.60	37.10	38.00	38.00
Group 05 855 mg l/kg NCL 388 - Main	11	M	MHL	5	32.00	33.40	34.00	33.70
Group 05 855 mg l/kg NCL 388 - Main	25	M	MHL	5	30.70	31.40	32.00	31.90
Group 05 855 mg l/kg NCL 388 - Main	26	M	MHL	5	33.80	34.80	35.90	35.70
Group 05 855 mg l/kg NCL 388 - Main	28	M	PPRO	2	38.60			
Group 05 855 mg l/kg NCL 388 - Main	43	M	MHL	5	33.80	35.40	36.00	36.10
Group 05 855 mg l/kg NCL 388 - Main	47	M	MHL	5	34.20	34.40	35.60	35.50
Group 05 855 mg l/kg NCL 388 - Main	50	M	MHL	5	34.80	36.80	37.50	37.10

Table B-3b. Recovery Study Absolute Body Weight (grams).

(See key following Table.)

Group	Animal ID	Sex	Fate		10/5/2020	10/7/2020	10/8/2020	10/9/2020	10/14/2020	10/16/2020	10/19/2020	10/21/2020	10/22/2020
			Code	Day	1	3	4	5	10	12	15	17	18
Group 02 Saline Vehicle - Recovery	02	M	MHL	18	34.10	33.00	33.50	33.20	36.30	35.80	36.30	36.90	36.80
Group 02 Saline Vehicle - Recovery	03	M	MHL	18	36.40	36.50	36.00	35.70	37.70	37.90	38.50	38.10	38.00
Group 02 Saline Vehicle - Recovery	15	M	MHL	18	35.50	35.00	35.00	35.10	36.70	36.60	36.70	37.70	37.30
Group 02 Saline Vehicle - Recovery	16	M	MHL	18	35.30	35.40	35.50	35.10	36.90	37.10	37.40	37.90	37.60
Group 02 Saline Vehicle - Recovery	19	M	MHL	18	36.40	36.20	36.00	36.10	37.40	37.60	37.40	38.30	38.20
Group 02 Saline Vehicle - Recovery	20	M	MHL	18	38.50	38.00	38.10	38.20	38.60	39.60	39.20	40.10	40.20
Group 02 Saline Vehicle - Recovery	24	M	MHL	18	34.40	34.30	35.00	34.70	35.10	35.40	36.30	35.50	35.10
Group 02 Saline Vehicle - Recovery	32	M	MHL	18	33.20	34.00	34.00	34.10	34.90	36.90	36.80	37.40	37.10
Group 04 1715 mg I/kg NCL 388 - Recovery	01	M	MHL	18	35.70	37.00	37.10	37.00	32.10	39.80	40.70	40.70	40.30
Group 04 1715 mg I/kg NCL 388 - Recovery	14	M	MHL	18	36.20	39.60	39.50	38.10	41.50	42.50	43.30	44.00	43.70
Group 04 1715 mg I/kg NCL 388 - Recovery	21	M	MHL	18	29.80	31.20	31.80	31.50	31.90	32.90	33.30	34.10	33.70
Group 04 1715 mg I/kg NCL 388 - Recovery	44	M	MHL	18	38.40	41.80	42.00	42.00	43.70	44.50	45.50	46.30	46.00
Group 04 1715 mg I/kg NCL 388 - Recovery	46	M	MHL	18	34.80	35.70	37.20	37.00	39.10	40.10	41.20	41.60	41.30
Group 04 1715 mg I/kg NCL 388 - Recovery	49	M	MHL	18	31.20	31.80	32.00	31.90	32.50	33.20	33.80	34.70	34.30
Group 04 1715 mg I/kg NCL 388 - Recovery	51	M	MHL	18	36.20	38.40	39.50	39.30	42.70	42.10	42.40	42.80	42.60
Group 04 1715 mg I/kg NCL 388 - Recovery	52	M	MHL	18	32.90	34.00	36.00	36.00	37.40	37.40	37.80	38.10	38.00
Group 06 855 mg I/kg NCL 388 - Recovery	07	M	MHL	18	34.40	35.80	36.20	36.10	38.00	38.80	38.90	39.40	39.00
Group 06 855 mg I/kg NCL 388 - Recovery	17	M	MHL	18	35.40	35.80	36.50	36.10	38.50	39.50	39.70	40.30	40.10
Group 06 855 mg I/kg NCL 388 - Recovery	22	M	MHL	18	35.70	35.70	36.10	36.20	37.10	37.80	39.60	39.90	39.50
Group 06 855 mg I/kg NCL 388 - Recovery	23	M	MHL	18	36.50	38.40	39.10	39.00	40.40	41.40	42.20	43.30	43.00
Group 06 855 mg I/kg NCL 388 - Recovery	33	M	MHL	18	36.20	37.50	38.20	38.10	39.70	39.80	40.20	40.80	40.40
Group 06 855 mg I/kg NCL 388 - Recovery	37	M	MHL	18	30.90	31.40	31.60	31.50	34.00	34.10	33.70	34.80	34.70
Group 06 855 mg I/kg NCL 388 - Recovery	39	M	MHL	18	34.50	33.40	34.20	34.10	36.00	36.50	36.40	37.50	37.30
Group 06 855 mg I/kg NCL 388 - Recovery	45	M	MHL	18	34.10	33.20	35.00	34.70	36.90	37.20	37.20	37.90	37.70

Key:

MHL- Submitted to MHL for Analysis
PPRO- Post-Procedure Death

Main and Recovery Groups

Group	Type	Article	Vehicle	Dose Level	Unit	Route	Frequency	Vehicle	Dose Rule
Group 01	Control	Vehicle	PBS	0	mg I/kg	Intravenous	qd x 4; 4 total injections	PBS	By Individual BW (25 µl/g)
Group 02	Control	Vehicle	PBS	0	mg I/kg	Intravenous	qd x 4; 4 total injections	PBS	By Individual BW (25 µl/g)
Group 03	Test	NCL 388	PBS	1715	mg i/kg	Intravenous	qd x 4; 4 total injections	PBS	By Individual BW (25 µl/g)
Group 04	Test	NCL 388	PBS	1715	mg I/kg	Intravenous	qd x 4; 4 total injections	PBS	By Individual BW (25 µl/g)
Group 05	Test	NCL 388	PBS	855	mg I/kg	Intravenous	qd x 4; 4 total injections	PBS	By Individual BW (25 µl/g)
Group 06	Test	NCL 388	PBS	855	mg I/kg	Intravenous	qd x 4; 4 total injections	PBS	By Individual BW (25 µl/g)

Table B-4a. Group 1 Organ Weight Parameters.

Treatment		Saline							
Number of Animals		8							
Animal #		5	8	10	18	27	38	40	41
**Body Weight**									
Absolute Weight	Gram	33.72	31.07	30.91	35.27	38.67	32.65	36.66	35.26
**Brain**									
Absolute Weight	Gram	0.49	0.49	0.42	0.48	0.49	0.47	0.52	0.47
Per body weight	%	1.44	1.58	1.36	1.35	1.27	1.45	1.41	1.34
**Heart**									
Absolute Weight	Gram	0.19	0.17	0.16	0.19	0.23	0.21	0.18	0.22
Per body weight	%	0.55	0.54	0.51	0.54	0.59	0.64	0.49	0.61
Per brain weight	%	38.35	34.01	37.62	40.00	46.23	44.09	34.88	45.65
**Kidney**									
Absolute Weight	Gram	0.54	0.59	0.44	0.52	0.69	0.66	0.57	0.53
Per body weight	%	1.59	1.89	1.42	1.49	1.77	2.03	1.55	1.51
Per brain weight	%	110.31	119.76	104.52	110.32	139.51	140.08	110.27	112.74
**Liver**									
Absolute Weight	Gram	1.76	2.01	1.95	2.04	2.64	2.19	2.25	2.16
Per body weight	%	5.23	6.48	6.30	5.79	6.83	6.70	6.13	6.12
Per brain weight	%	363.71	409.78	463.33	429.89	537.88	461.39	435.27	457.75
**Lung**									
Absolute Weight	Gram	0.31	0.22	0.19	0.41	0.23	0.23	0.23	0.36
Per body weight	%	0.93	0.71	0.61	1.17	0.60	0.70	0.62	1.03
Per brain weight	%	64.33	45.01	45.24	87.16	47.45	48.31	44.38	77.07
**Spleen**									
Absolute Weight	Gram	0.09	0.06	0.10	0.10	0.09	0.13	0.11	0.08
Per body weight	%	0.25	0.18	0.31	0.28	0.23	0.41	0.29	0.22
Per brain weight	%	17.53	11.41	23.10	20.84	18.13	28.06	20.74	16.56

Table B-4b. Group 2 Organ Weight Parameters.

Treatment		Saline - Recovery							
Number of Animals		8							
Animal #		2	3	15	16	19	20	24	32
**Body Weight**									
Absolute Weight	Gram	35.25	38.15	36.59	35.38	37.93	38.20	34.08	35.44
**Brain**									
Absolute Weight	Gram	0.49	0.50	0.52	0.51	0.53	0.53	0.52	0.49
Per body weight	%	1.38	1.30	1.42	1.44	1.40	1.39	1.54	1.37
**Heart**									
Absolute Weight	Gram	0.19	0.20	0.17	0.20	0.19	0.24	0.21	0.17
Per body weight	%	0.54	0.53	0.45	0.56	0.50	0.63	0.60	0.49
Per brain weight	%	38.73	40.73	31.67	39.02	35.47	45.39	39.31	35.32
**Kidney**									
Absolute Weight	Gram	0.58	0.58	0.60	0.64	0.64	0.63	0.61	0.49
Per body weight	%	1.64	1.52	1.63	1.81	1.68	1.65	1.78	1.39
Per brain weight	%	118.24	116.53	114.40	125.69	120.00	118.46	115.84	100.82
**Liver**									
Absolute Weight	Gram	2.00	2.26	2.31	2.16	2.39	2.58	1.77	1.95
Per body weight	%	5.67	5.91	6.31	6.09	6.29	6.75	5.20	5.51
Per brain weight	%	409.22	454.64	443.38	422.55	450.19	485.50	338.17	400.82
**Lung**									
Absolute Weight	Gram	0.22	0.25	0.24	0.23	0.28	0.25	0.52	0.38
Per body weight	%	0.61	0.66	0.65	0.65	0.75	0.66	1.53	1.08
Per brain weight	%	44.06	50.81	45.49	45.10	53.40	47.27	99.62	78.44
**Spleen**									
Absolute Weight	Gram	0.09	0.10	0.10	0.09	0.11	0.14	0.08	0.10
Per body weight	%	0.27	0.26	0.28	0.25	0.28	0.37	0.23	0.28
Per brain weight	%	19.26	19.96	19.39	17.06	20.19	26.74	15.27	20.33

Table B-4c. Group 3 Organ Weight Parameters.

Treatment		NCL388-12, 1715 mg I/kg - Main							
Number of Animals		8							
Animal #		4	12	13	30	31	35	36	42
**Body Weight**									
Absolute Weight	Gram	39.40	37.19	39.28	38.64	33.88	39.71	36.65	37.00
**Brain**									
Absolute Weight	Gram	0.53	0.49	0.50	0.51	0.50	0.51	0.50	0.51
Per body weight	%	1.33	1.31	1.27	1.31	1.48	1.28	1.38	1.37
**Heart**									
Absolute Weight	Gram	0.22	0.18	0.17	0.20	0.21	0.21	0.17	0.23
Per body weight	%	0.56	0.49	0.44	0.52	0.63	0.52	0.47	0.61
Per brain weight	%	42.10	37.24	34.87	39.17	42.32	40.67	34.52	44.49
**Kidney**									
Absolute Weight	Gram	0.65	0.57	0.45	0.68	0.49	0.59	0.51	0.61
Per body weight	%	1.64	1.53	1.13	1.77	1.46	1.48	1.38	1.66
Per brain weight	%	122.86	116.87	89.18	134.45	98.60	115.13	100.60	120.87
**Liver**									
Absolute Weight	Gram	2.94	2.33	2.84	2.79	2.15	3.02	2.19	2.64
Per body weight	%	7.47	6.26	7.23	7.21	6.33	7.62	5.98	7.14
Per brain weight	%	560.76	479.01	569.34	548.43	428.34	594.11	434.52	519.88
**Lung**									
Absolute Weight	Gram	0.35	0.25	0.31	0.21	0.19	0.24	0.29	0.24
Per body weight	%	0.89	0.68	0.78	0.54	0.57	0.60	0.79	0.64
Per brain weight	%	66.86	51.85	61.72	41.14	38.52	46.56	57.74	46.85
**Spleen**									
Absolute Weight	Gram	0.10	0.14	0.18	0.13	0.27	0.12	0.12	0.12
Per body weight	%	0.26	0.38	0.47	0.33	0.79	0.31	0.32	0.33
Per brain weight	%	19.62	29.22	36.67	25.20	53.09	23.97	23.41	24.21

Table B-4d. Group 4 Organ Weight Parameters.

Treatment		NCL388-12, 1715 mg I/kg - Recovery							
Number of Animals		8							
Animal #		1	14	21	44	46	49	51	52
**Body Weight**									
Absolute Weight	Gram	38.96	43.37	33.26	44.79	39.34	33.38	42.56	37.10
**Brain**									
Absolute Weight	Gram	0.49	0.51	0.47	0.49	0.47	0.47	0.52	0.49
Per body weight	%	1.27	1.18	1.42	1.09	1.20	1.41	1.22	1.33
**Heart**									
Absolute Weight	Gram	0.19	0.24	0.17	0.19	0.21	0.20	0.20	0.19
Per body weight	%	0.49	0.55	0.50	0.43	0.53	0.59	0.47	0.50
Per brain weight	%	38.66	46.77	35.17	39.71	43.76	41.70	38.58	38.01
**Kidney**									
Absolute Weight	Gram	0.60	0.64	0.48	0.64	0.60	0.46	0.61	0.45
Per body weight	%	1.54	1.48	1.46	1.43	1.53	1.37	1.44	1.20
Per brain weight	%	121.26	126.03	102.54	131.69	127.27	97.45	117.85	90.85
**Liver**									
Absolute Weight	Gram	3.69	3.60	2.54	3.35	3.57	2.44	3.55	2.84
Per body weight	%	9.46	8.30	7.64	7.48	9.08	7.30	8.34	7.66
Per brain weight	%	745.95	704.31	538.56	689.09	755.39	518.72	681.57	577.44
**Lung**									
Absolute Weight	Gram	0.23	0.22	0.29	0.37	0.26	0.23	0.30	0.28
Per body weight	%	0.58	0.51	0.86	0.82	0.67	0.68	0.70	0.76
Per brain weight	%	45.95	43.44	60.81	75.51	55.60	48.09	57.58	57.52
**Spleen**									
Absolute Weight	Gram	0.51	0.19	0.26	0.23	0.21	0.14	0.26	0.39
Per body weight	%	1.32	0.44	0.77	0.50	0.53	0.42	0.60	1.06
Per brain weight	%	103.85	37.57	54.24	46.50	44.19	29.79	48.94	79.67

Table B-4e. Group 5 Organ Weight Parameters.

Treatment		NCL388-12, 855 mg I/kg - Main							
Number of Animals		8							
Animal #		6	11	25	26	28	43	47	50
**Body Weight**									
Absolute Weight	Gram	37.99	34.30	33.61	35.21	39.37	35.49	34.76	37.50
**Brain**									
Absolute Weight	Gram	0.53	0.48	0.48	0.50	0.55	0.46	0.49	0.49
Per body weight	%	1.39	1.39	1.42	1.42	1.39	1.29	1.40	1.29
**Heart**									
Absolute Weight	Gram	0.19	0.18	0.16	0.23	0.17	0.18	0.18	0.18
Per body weight	%	0.51	0.52	0.48	0.66	0.43	0.51	0.52	0.49
Per brain weight	%	36.29	37.47	34.24	46.71	30.77	39.82	37.30	37.94
**Kidney**									
Absolute Weight	Gram	0.53	0.53	0.52	0.64	0.66	0.57	0.60	0.57
Per body weight	%	1.38	1.54	1.56	1.80	1.67	1.61	1.73	1.52
Per brain weight	%	99.43	111.37	109.87	126.75	120.15	124.95	123.16	117.32
**Liver**									
Absolute Weight	Gram	2.52	2.33	2.39	2.26	3.21	2.15	2.30	2.33
Per body weight	%	6.64	6.81	7.10	6.42	8.15	6.04	6.62	6.22
Per brain weight	%	476.75	491.37	501.68	451.30	587.73	469.37	471.31	481.24
**Lung**									
Absolute Weight	Gram	0.29	0.42	0.25	0.42	0.22	0.22	0.25	0.22
Per body weight	%	0.77	1.22	0.75	1.20	0.57	0.63	0.71	0.60
Per brain weight	%	55.01	88.00	52.94	84.03	40.84	49.02	50.61	46.19
**Spleen**									
Absolute Weight	Gram	0.12	0.11	0.09	0.10	0.08	0.08	0.10	0.11
Per body weight	%	0.31	0.32	0.28	0.28	0.21	0.23	0.30	0.29
Per brain weight	%	22.31	23.37	19.54	19.56	15.20	17.94	21.31	22.68

Table B-4f. Group 6 Organ Weight Parameters.

Treatment		NCL388-12, 855 mg I/kg - Recovery							
Number of Animals		8							
Animal #		7	17	22	23	33	37	39	45
**Body Weight**									
Absolute Weight	Gram	38.80	39.20	39.29	41.56	39.93	33.78	35.71	36.19
**Brain**									
Absolute Weight	Gram	0.49	0.49	0.51	0.51	0.48	0.46	0.50	0.52
Per body weight	%	1.26	1.25	1.29	1.22	1.19	1.37	1.40	1.43
**Heart**									
Absolute Weight	Gram	0.17	0.19	0.20	0.19	0.20	0.17	0.19	0.18
Per body weight	%	0.45	0.48	0.50	0.46	0.50	0.52	0.53	0.50
Per brain weight	%	35.58	38.21	38.81	37.94	42.14	37.58	37.92	35.01
**Kidney**									
Absolute Weight	Gram	0.66	0.60	0.62	0.57	0.61	0.57	0.52	0.55
Per body weight	%	1.70	1.54	1.58	1.37	1.53	1.68	1.45	1.52
Per brain weight	%	135.17	122.36	123.17	112.85	128.30	122.25	103.59	106.19
**Liver**									
Absolute Weight	Gram	2.90	2.88	2.48	2.67	2.72	2.73	2.43	2.10
Per body weight	%	7.46	7.35	6.31	6.43	6.82	8.09	6.79	5.81
Per brain weight	%	592.02	585.57	490.89	528.46	570.86	590.50	484.03	406.96
**Lung**									
Absolute Weight	Gram	0.25	0.21	0.24	0.27	0.29	0.20	0.22	0.25
Per body weight	%	0.64	0.55	0.61	0.65	0.72	0.59	0.62	0.68
Per brain weight	%	50.72	43.50	47.13	53.56	60.38	42.98	43.91	47.39
**Spleen**									
Absolute Weight	Gram	0.15	0.14	0.14	0.15	0.18	0.35	0.14	0.14
Per body weight	%	0.39	0.35	0.34	0.36	0.45	1.04	0.40	0.40
Per brain weight	%	31.29	28.05	26.73	29.84	37.74	76.03	28.54	27.85

Table B-5a. Saline Immunohistochemistry Parameters.

Treatment	Saline					
Number of Animals	4					
Animal ID	2	3	15	16	**AVG**	**STDEV**
**F4/80**						
Positive Cells (per mm^2)	802	725	595	566	**672.10**	**111.00**

Table B-5b. Main Immunohistochemistry Parameters.

Treatment	NCL388 1715 mg I/kg (Main)					
Number of Animals	4					
Animal ID	30	31	35	36	**AVG**	**STDEV**
**F4/80**						
Positive Cells (per mm^2)	1427	1571	1407	1619	**1505.87**	**104.89**

Table B-5c. Recovery Immunohistochemistry Parameters.

Treatment	NCL388-12, 1715 mg I/kg (Recovery)					
Number of Animals	4					
Animal ID	1	14	21	49	**AVG**	**STDEV**
**F4/80**						
Positive Cells (per mm^2)	1815	2408	2031	1908	**2040.57**	**260.48**

Appendix C. Randomization Report (ADME-Tox 193)

Study:	19-274 ADME TOX 193
Date of Grouping:	10/5/2020
Time of Grouping:	8:54 AM
Authorized by:	
No. of Assigned Animals:	48
No. of Groups:	6
Randomization Method:	Stratified
Key of Randomization:	Body Weight (g)

Group	Animal ID	Temp ID	Weight (g)
Group 01 Saline Vehicle - Main	05	05	32
Group 01 Saline Vehicle - Main	08	08	32.2
Group 01 Saline Vehicle - Main	10	10	34.4
Group 01 Saline Vehicle - Main	18	18	35.6
Group 01 Saline Vehicle - Main	27	27	39.1
Group 01 Saline Vehicle - Main	38	38	34.6
Group 01 Saline Vehicle - Main	40	40	37.6
Group 01 Saline Vehicle - Main	41	41	36
Group 02 Saline Vehicle - Recovery	02	02	34.1
Group 02 Saline Vehicle - Recovery	03	03	36.4
Group 02 Saline Vehicle - Recovery	15	15	35.5
Group 02 Saline Vehicle - Recovery	16	16	35.3
Group 02 Saline Vehicle - Recovery	19	19	36.4
Group 02 Saline Vehicle - Recovery	20	20	38.5
Group 02 Saline Vehicle - Recovery	24	24	34.4
Group 02 Saline Vehicle - Recovery	32	32	33.2
Group 03 1715 mg I/kg NCL 388 - Main	04	04	37.5
Group 03 1715 mg I/kg NCL 388 - Main	12	12	36
Group 03 1715 mg I/kg NCL 388 - Main	13	13	36.1
Group 03 1715 mg I/kg NCL 388 - Main	30	30	37
Group 03 1715 mg I/kg NCL 388 - Main	31	31	32
Group 03 1715 mg I/kg NCL 388 - Main	35	35	35.6
Group 03 1715 mg I/kg NCL 388 - Main	36	36	34.4
Group 03 1715 mg I/kg NCL 388 - Main	42	42	33.6
Group 04 1715 mg I/kg NCL 388 - Recovery	01	01	35.7
Group 04 1715 mg I/kg NCL 388 - Recovery	14	14	37.5
Group 04 1715 mg I/kg NCL 388 - Recovery	21	21	30.6
Group 04 1715 mg I/kg NCL 388 - Recovery	44	44	39.4
Group 04 1715 mg I/kg NCL 388 - Recovery	46	46	34.8
Group 04 1715 mg I/kg NCL 388 - Recovery	49	49	31.2
Group 04 1715 mg I/kg NCL 388 - Recovery	51	51	36.2
Group 04 1715 mg I/kg NCL 388 - Recovery	52	52	33.5
Group 05 855 mg I/kg NCL 388 - Main	06	06	38.6
Group 05 855 mg I/kg NCL 388 - Main	11	11	32.7
Group 05 855 mg I/kg NCL 388 - Main	25	25	31.5
Group 05 855 mg I/kg NCL 388 - Main	26	26	34.7
Group 05 855 mg I/kg NCL 388 - Main	28	28	39
Group 05 855 mg I/kg NCL 388 - Main	43	43	35.4
Group 05 855 mg I/kg NCL 388 - Main	47	47	34.7
Group 05 855 mg I/kg NCL 388 - Main	50	50	36
Group 06 855 mg I/kg NCL 388 - Recovery	07	07	34.4
Group 06 855 mg I/kg NCL 388 - Recovery	17	17	35.6
Group 06 855 mg I/kg NCL 388 - Recovery	22	22	36.4
Group 06 855 mg I/kg NCL 388 - Recovery	23	23	36.7
Group 06 855 mg I/kg NCL 388 - Recovery	33	33	36.6
Group 06 855 mg I/kg NCL 388 - Recovery	37	37	32
Group 06 855 mg I/kg NCL 388 - Recovery	39	39	34.5
Group 06 855 mg I/kg NCL 388 - Recovery	45	45	34.2

Randomization on 10/5/2020
Randomized by Body Weight (g)

Body Weight (g)

Group	F	M	T	Mean	Std Dev	Min	Max
Group 01 Saline Vehicle - Main	0	8	8	35.19	2.45	32.00	39.10
Group 02 Saline Vehicle - Recovery	0	8	8	35.48	1.65	33.20	38.50
Group 03 1715 mg I/kg NCL 388 - Main	0	8	8	35.28	1.83	32.00	37.50
Group 04 1715 mg I/kg NCL 388 - Recovery	0	8	8	34.86	3.01	30.60	39.40
Group 05 855 mg I/kg NCL 388 - Main	0	8	8	35.33	2.59	31.50	39.00
Group 06 855 mg I/kg NCL 388 - Recovery	0	8	8	35.05	1.60	32.00	36.70
Brown-Forsythe:		W =		0.8920	P =	0.4952	
ANOVA:		F =		0.0736	P =	0.9959	

List of Temp ID

Temp ID	Group	Animal ID	BW (g)
01	Group 04 1715 mg I/kg NCL 388 - Recovery	01	35.70
02	Group 02 Saline Vehicle - Recovery	02	34.10
03	Group 02 Saline Vehicle - Recovery	03	36.40
04	Group 03 1715 mg I/kg NCL 388 - Main	04	37.50
05	Group 01 Saline Vehicle - Main	05	32.00
06	Group 05 855 mg I/kg NCL 388 - Main	06	38.60
07	Group 06 855 mg I/kg NCL 388 - Recovery	07	34.40
08	Group 01 Saline Vehicle - Main	08	32.20
10	Group 01 Saline Vehicle - Main	10	34.40
11	Group 05 855 mg I/kg NCL 388 - Main	11	32.70
12	Group 03 1715 mg I/kg NCL 388 - Main	12	36.00
13	Group 03 1715 mg I/kg NCL 388 - Main	13	36.10
14	Group 04 1715 mg I/kg NCL 388 - Recovery	14	37.50
15	Group 02 Saline Vehicle - Recovery	15	35.50
16	Group 02 Saline Vehicle - Recovery	16	35.30
17	Group 06 855 mg I/kg NCL 388 - Recovery	17	35.60
18	Group 01 Saline Vehicle - Main	18	35.60
19	Group 02 Saline Vehicle - Recovery	19	36.40
20	Group 02 Saline Vehicle - Recovery	20	38.50
21	Group 04 1715 mg I/kg NCL 388 - Recovery	21	30.60
22	Group 06 855 mg I/kg NCL 388 - Recovery	22	36.40
23	Group 06 855 mg I/kg NCL 388 - Recovery	23	36.70
24	Group 02 Saline Vehicle - Recovery	24	34.40
25	Group 05 855 mg I/kg NCL 388 - Main	25	31.50
26	Group 05 855 mg I/kg NCL 388 - Main	26	34.70
27	Group 01 Saline Vehicle - Main	27	39.10
28	Group 05 855 mg I/kg NCL 388 - Main	28	39.00
30	Group 03 1715 mg I/kg NCL 388 - Main	30	37.00
31	Group 03 1715 mg I/kg NCL 388 - Main	31	32.00
32	Group 02 Saline Vehicle - Recovery	32	33.20
33	Group 06 855 mg I/kg NCL 388 - Recovery	33	36.60
35	Group 03 1715 mg I/kg NCL 388 - Main	35	35.60
36	Group 03 1715 mg I/kg NCL 388 - Main	36	34.40
37	Group 06 855 mg I/kg NCL 388 - Recovery	37	32.00
38	Group 01 Saline Vehicle - Main	38	34.60
39	Group 06 855 mg I/kg NCL 388 - Recovery	39	34.50
40	Group 01 Saline Vehicle - Main	40	37.60
41	Group 01 Saline Vehicle - Main	41	36.00
42	Group 03 1715 mg I/kg NCL 388 - Main	42	33.60
43	Group 05 855 mg I/kg NCL 388 - Main	43	35.40
44	Group 04 1715 mg I/kg NCL 388 - Recovery	44	39.40
45	Group 06 855 mg I/kg NCL 388 - Recovery	45	34.20
46	Group 04 1715 mg I/kg NCL 388 - Recovery	46	34.80
47	Group 05 855 mg I/kg NCL 388 - Main	47	34.70
49	Group 04 1715 mg I/kg NCL 388 - Recovery	49	31.20
50	Group 05 855 mg I/kg NCL 388 - Main	50	36.00
51	Group 04 1715 mg I/kg NCL 388 - Recovery	51	36.20
52	Group 04 1715 mg I/kg NCL 388 - Recovery	52	33.50

Animal Distribution

Group 01 Saline Vehicle - Main: Vehicle

Animal ID	Temp ID	BW g	BW Date	BW Day
05	05	32.00	10/5/2020	1
08	08	32.20	10/5/2020	1
10	10	34.40	10/5/2020	1
18	18	35.60	10/5/2020	1
27	27	39.10	10/5/2020	1
38	38	34.60	10/5/2020	1
40	40	37.60	10/5/2020	1
41	41	36.00	10/5/2020	1

Group 02 Saline Vehicle - Recovery: Vehicle

Animal ID	Temp ID	BW g	BW Date	BW Day
02	02	34.10	10/5/2020	1
03	03	36.40	10/5/2020	1
15	15	35.50	10/5/2020	1
16	16	35.30	10/5/2020	1
19	19	36.40	10/5/2020	1
20	20	38.50	10/5/2020	1
24	24	34.40	10/5/2020	1
32	32	33.20	10/5/2020	1

Group 03 1715 mg I/kg NCL 388 - Main: 1715mg I/kg NCL 388

Animal ID	Temp ID	BW g	BW Date	BW Day
04	04	37.50	10/5/2020	1
12	12	36.00	10/5/2020	1
13	13	36.10	10/5/2020	1
30	30	37.00	10/5/2020	1
31	31	32.00	10/5/2020	1
35	35	35.60	10/5/2020	1
36	36	34.40	10/5/2020	1
42	42	33.60	10/5/2020	1

Group 04 1715 mg I/kg NCL 388 - Recovery: 1715mg I/kg NCL 388

Animal ID	Temp ID	BW g	BW Date	BW Day
01	01	35.70	10/5/2020	1
14	14	37.50	10/5/2020	1
21	21	30.60	10/5/2020	1
44	44	39.40	10/5/2020	1
46	46	34.80	10/5/2020	1
49	49	31.20	10/5/2020	1
51	51	36.20	10/5/2020	1
52	52	33.50	10/5/2020	1

Group 05 855 mg I/kg NCL 388 - Main: 855mg I/kg NCL 388

Animal ID	Temp ID	BW g	BW Date	BW Day
06	06	38.60	10/5/2020	1
11	11	32.70	10/5/2020	1
25	25	31.50	10/5/2020	1
26	26	34.70	10/5/2020	1
28	28	39.00	10/5/2020	1
43	43	35.40	10/5/2020	1
47	47	34.70	10/5/2020	1
50	50	36.00	10/5/2020	1

Group 06 855 mg I/kg NCL 388 - Recovery: 855mg I/kg NCL 388

Animal ID	Temp ID	BW g	BW Date	BW Day
07	07	34.40	10/5/2020	1
17	17	35.60	10/5/2020	1
22	22	36.40	10/5/2020	1
23	23	36.70	10/5/2020	1
33	33	36.60	10/5/2020	1
37	37	32.00	10/5/2020	1
39	39	34.50	10/5/2020	1
45	45	34.20	10/5/2020	1

Appendix D. Study Protocol (ADME-Tox 193)

ASP#: 19-274 **IBC#: 2016-60/13-03** ☐**BSL1** ☐ **BSL2** ☐ **ABSL1** ☐ **ABSL2** **X** **Respirator required/Nano Precautions** ☐**IC Suite**

Investigator/PI: Stern **Facility/Room:** 539-1CC/Rm-53 **LASP Study Monitor(s)**: Christina Robinson/Lai Thang

Study ID: ADME TOX 193 **Project ID: 600.300.41.01.035.001.0001** **NAS#: CS027663**

Study Title: Multidose Nanoprobes Toxicity Study in Mice

Study Initiation Date: 10/5/20 **Anticipated End Date:** 10/22/20

Animal Information: Total # of animals: 53 Animal IDs: 1-53

Age at study start: 7 weeks DOB: _____ Sex: Male Source: Charles River Species: Mice Strain name: CD-1

Comments : Total mice needed= (8/Tx x 3 Tx x 2 (main and recovery groups) = 48 + 5 extra: 53 mice

Projected Delivery Date: _____

Special Housing Consideration:

☒ Water Type: Automatic RO Start/End: 9/30/20

☒ Feed Type: Purina 5L79 Start/End: 9/30/20

☐ Special water Type: _____ Start/End: _____

☐ Special Feed Type: _____ Start/End: _____

☐ Full micro-isolator technique for cage changes

Treatment:

Randomization criteria: ☐ Tumor volume ☒ Body weight ☐ Imaging data: _____ ☐ Other: _____

NOTE: Randomize animals into two **metabolic** cages per group (4 mice in each cage).

Enroll 48 mice (order 53 animals for randomization)

GROUP ID	# OF ANIMALS	ANIMAL #S	SEX (M/F)	CAGE #S	TREATMENT	Dose Level	VEHICLE	ROUTE	Dosing schedule	Dosing Dates	EU Dates
1	8	5, 8, 10,18, 27, 38, 40, 41	M	TBD	saline vehicle-Main	0.25 mL/10g of BW	PBS	i.v. tail vein	qd x 4; 4 total injections	10/5-10/8	10/9/20
2	8	2, 3, 15, 16, 19, 20, 24, 32	M	TBD	saline vehicle-Recovery	0.25 mL/10g of BW	PBS	i.v. tail vein	qd x 4; 4 total injections	10/5-10/8	10/22/20

3	8	4, 12, 13, 30, 31, 35, 36, 42	M	TBD	1715 mg I/kg NCL 388-Main	0.25 mL/10g of BW	PBS	i.v. tail vein	qd x 4; 4 total injections	10/5-10/8	10/9/20
4	8	1, 14, 21, 44, 46, 49, 51, 52	M	TBD	1715 mg I/kg NCL 388-Recovery	0.25 mL/10g of BW	PBS	i.v. tail vein	qd x 4; 4 total injections	10/5-10/8	10/22/20
5	8	6, 11, 25, 26, 28, 43, 47, 50	M	TBD	855 mg I/kg NCL 388-Main	0.25 mL/10g of BW	PBS	i.v. tail vein	qd x 4; 4 total injections	10/5-10/8	10/9/20
6	8	7, 17, 22, 23, 33, 37, 39, 45	M	TBD	855 mg I/kg NCL 388-Recovery	0.25 mL/10g of BW	PBS	i.v. tail vein	qd x 4; 4 total injections	10/5-10/8	10/22/20

Tech Support Requested:

☐ Tumor Measurements (Frequency)
Equation to calculate volume: ☐ L*W*H* π /6 [3D] ☐ (L x W^2) / 2 [2D]
☒ Body Weights (Frequency: M, W, F)
☐ Bleeds (Frequency or Dates/Route/Volume: _____)
☐ K_3EDTA ☐ K_2EDTA ☐ Heparinized ☐ uncoated tubes
☐ Serum ☐ Plasma ☐ Whole blood
Spin down instructions & storage if applicable:______)
☐ Food Consumption: Frequency: _____)
☒ Fecal sample collection: Frequency/Dates: once daily at 24 hr intervals post final dose; also on study day 17 for recovery groups). Store fecal samples at -20°C
☒ Urine sample collection: Frequency/Dates: once daily at 24 hr intervals post final dose; also on study day 17 for recovery groups). Store urine samples at -20°C

Endpoints:

Clinical signs expected/evaluated**:** none
☐ Scheduled Date (s): _____
☒ Survival based on following endpoints: ☒ >20% Body weight loss ☐ Tumor >2 cm in diameter ☐ Tumor volume exceeding _____mm^3 ☐ Other

Necropsy performed by: ☒ MHL

MHL Zap Study# (if required): MHL Contact: Elijah Edmondson MHL instructions (e.g. H&E, etc.): Animals will be euthanized by CO2 asphyxiation and will be coordinated with MHL (necropsy will be performed by MHL staff). All standard tissues (including inguinal lymph node) will be fixed/block/slide for gross description, weights and histopathology analysis per general necropsy protocol. Blood is collected for hematology and clinical chemistry, according to necropsy protocol.

Disposition of found dead animals: ☐ send to NIH ☐ discard ☒ send to MHL ☐ refrigerate ☐ freeze
Ship to Laboratory if applicable (date): _____

Additional Notes/Comments: Animals will be acclimated for a week prior to study initiation. Animals will be randomized based on body weight. Treatment (i.v. tail vein, qd x 4; 4 total injections) is initiated by intravenous tail vein injection. Animal behavior is monitored daily and body weights are measured every alternate day (M, W, and F). Urine and feces are to be collected at 24 hr interval following the final dose for both main and recovery groups, and from recovery groups on study day 17 (13 days post the final dose). Fecal and urine samples will be collected from each cage totaling 2 samples per group, per collection (please collect entire sample). Animals in the main groups are terminated on study day 5, 24h post last dose. Animals in the recovery groups are terminated on study 18, 14 days post last dose. All animals go to necropsy at MHL. Animal is euthanized and necropsy is performed on animals showing signs morbidity or ≥20% body weight loss due to treatment.

Chemical Preparation:

**Chemical**	Dose (mg/kg)	Dose Volume (0.1ml/10g BW, 0.05ml/10gBW or Fixed volume)	Vehicle Including Percentages of Total Volume	Prepare Fresh at each Dose	Prepare Fresh Daily	Prepare Weekly	Storage at -80C	Storage at -20C	Storage at 4C	Storage at Room Temperature
Saline Vehicle	NA	25 mL/kg	PBS	☐	☐	☐	☐	☐	☒	☐
NCL 388 iodine polymer	855 and 1715 mg/ kg	25 mL/kg	PBS	☐	☒	☐	☐	☐	☒	☐

Special Instructions for Formulation: ____

Left over Drug from Dosing ☐ discard after each dose ☒ store for analysis ☒ Other Store at 4C

Left over Compounds ☐ discard at end of study ☒ return to PI ☐ Other _____

Contact Information (specific) for dead/moribund animals:

Name: Christina Robinson Office Phone #: 301-846-5500 Cell Phone #: 240-626-4593

Name: Lai Thang Office Phone #: 301-228-4431 Cell Phone #: 240-315-3570

Name: Stephan Stern Office Phone #: 301-846-6198 Cell Phone #: 301-676-0531

Signatures:

Principal Investigator: Stephan Stern Date: ______ Office Phone #: 301-846-6198 Cell Phone #: 301-676-0531

Appendix E. EDX Analysis of Tissue Samples

Liver tissue from the Multidose In Vivo Toxicology Study in CD-1 Mice (ADME Tox 193) was analyzed by high resolution Energy-Dispersive X-ray spectroscopy (EDX) mapping for determination of iodine nanopolymer subcellular hepatic distribution. NCI's NCL and Frederick National Laboratory for Cancer Research (FNLCR) performed the animal studies, electron microscopy tissue sample preparation and analysis, while the EDX iodine mapping was performed in collaboration with the Hitachi Maryland Applications Lab.

Materials and Methods

Materials and instruments were obtained from the following vendors: Formaldehyde and glutaraldehyde (Tousimis, Rockville, MD); Sodium cacodylate, Osmium, Embed-812 epoxy resin, and uranyl acetate (Electron Microscope Sciences, Fort Washington, PA); Ultra-microtome, lead citrate (Leica, Bannockburn, IL); Diamond knife (Diatome, Fort Washington, PA); Vacuum evaporator (Cressington, UK); Electron microscope (H7650 and S5000, Hitachi, Tokyo, Japan); EDX (Oxford, High Wycombe, UK); CCD Camera, (AMT, Danvers, MA).

Electron Microscopy (EM)

Iodine nanoparticle treated mouse liver was surgically removed and immediately fixed in the EM fixative containing 4% formaldehyde and 2% glutaraldehyde in 0.1M sodium cacodylate buffer (pH 7.4). The tissue sample was cut into 5 mm cubes with a clean single edge razor blade. The processing of the tissue samples for the thin-sectioned EM analysis was previously described [16]. Briefly, the tissues were washed in cacodylate buffer three times, 10 min each, and fixed in osmium tetroxide solution (1% v/v) in cacodylate buffer for 1 hr at room temperature in dark. The tissues were washed thoroughly in distilled water, and in sodium acetate buffer (0.1M pH 4.5). *En block* staining was carried out in 0.5% (w/v) uranyl acetate in sodium acetate buffer for 1 hr in dark. The tissue samples were dehydrated in a series of graduated ethanol solution (e.g., 35%, 50%, 75%, 95%, 100% v/v) and 100% propylene oxide. The tissue samples were infiltrated in equal mixture of propylene oxide and epoxy resin overnight on a rotating table. The tissue samples were embedded in pure epoxy resin filled Beem capsule mold and cured in an oven at 55°C for 48 hr. The cured epoxy blocks were removed from the mold and trimmed into less than 0.5 mm^2 with a clean single edge razor blade for thin sectioning. About 70 nm thin sections were made with an ultramicrotome equipped with a diamond knife. The thin sections were mounted on naked copper grids (150 mesh grids) and counter stained in aqueous uranyl acetate (0.5% w/v) and Reynorld's lead citrate. The thin sections were stabilized by carbon evaporation in a vacuum evaporator and examined in the EM and images captured with a digital camera.

Energy Dispersive X-Ray Analysis (EDX)

The thin tissue sections on the copper mesh grid, described above, were placed in a scanning transmission electron microscope (STEM) folder and examined in a high-resolution scanning electron microscope (SEM) equipped with EDX. The element specific X-ray signals were collected from three different areas of liver tissue for comparison and the spectrograph displayed (Figure E-1).

EM and SEM/EDX Results

The thin tissue sections of mouse liver samples were examined by EM, and normal Kupffer cells were observed in untreated mouse liver, while round and oval shaped vacuole inclusions were present in Kupffer cells from the iodine nanoparticle treated mice. The inclusion bodies were either slightly opaque or clear in appearance. However, direct visual observation by EM was unable to specifically relate the inclusions to the iodine nanoparticles. To positively identify the iodine nanoparticle content of these vacuoles in the Kupffer cells, a high-resolution scanning electron microscope (SEM) equipped with EDX was used to measure iodine concentrations in the same thin tissue section. The iodine X-ray signal was strongly detected in opaque bodies (area 1), while low level of iodine X-ray signal was detected in less opaque bodies (area 2), and no iodine signals were observed in cell nucleus (area 3) and other subcellular organelles, e.g., mitochondria (data not shown).

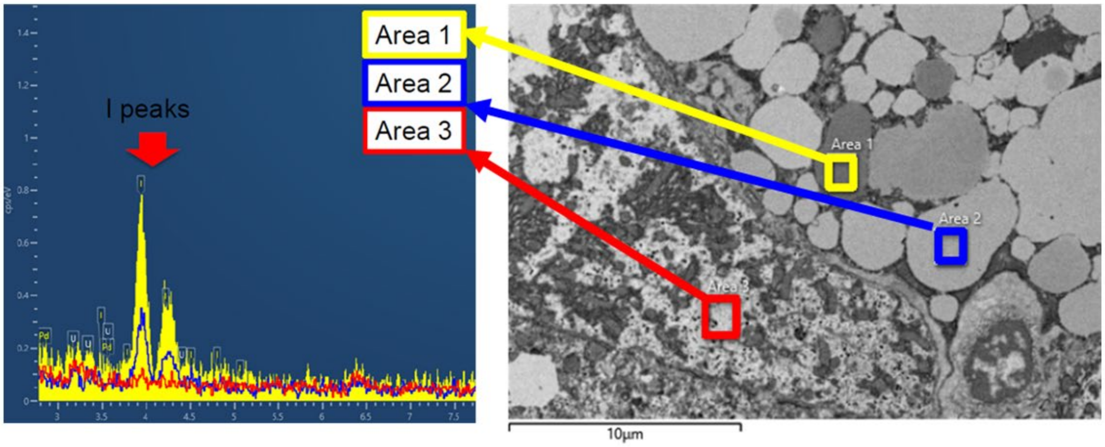

Figure E-1. Liver EDX. The image on this page displays high resolution Energy-dispersive X-ray spectroscopy (EDX) mapping of iodine nanopolymer subcellular hepatic distribution. Area 1 and Area 2 are hepatic Kupffer cell vacuoles, while area 3 is hepatic Kupffer cell nucleus (negative control area). Iodine in the Kupffer vacuoles was found to correlate with electron density.

Appendix F. ICP-MS Analysis of Tissue Samples

Nanoprobes provided several tissue samples for ICP-MS analysis of iodine concentrations. Initially, two non-tumor-bearing brain samples were provided to gauge the brain uptake of the particles. Next, Nanoprobes provided tissues from U87 tumor-bearing mice, three brains with smaller tumors, and three with larger tumors. In addition, they provided the corresponding liver and spleen samples from the tumor-bearing mice. Note, animal studies were conducted by Nanoprobes. NCL only performed ICP-MS analysis of specified tissues for iodine content.

The iodine concentration in the tissue samples was determined using a Perkin-Elmer NexION 2000B equipped with a micro-mist nebulizer, standard sample introduction system, and integrated auto-sampler, operated in standard mode was used. Tuning of the instrument was performed daily prior to sample testing.

Non-tumor bearing mice *(Animal study conducted by Nanoprobes)*
Two mice were intravenously administered NCL388 at 3.5 g iodine/kg. Brain tissues for both mice were provided to NCL for ICP-MS analysis of iodine content. Mouse one was sacrificed 1 day following administration. Mouse 2 was sacrificed three days following administration. The entire brain sample was homogenized prior to analysis.

Tumor bearing mice *(Animal study conducted by Nanoprobes)*
Six mice bearing U87 tumors were intravenously administered NCL388 at 3.5 g iodine/kg. Three mice had “small” tumors (as gauged by IVIS signals: 1.0, 1.2, 1.3 x 10e8 when injected, all 1.5 x 10e8 at 20 hours, and 2.0, 2.8, and 3.4 x 10e8 at 72 hours), and three mice had larger tumors. Mice were sacrificed 72 hours following administration. Brain, liver and spleen tissues were provided for all six mice for ICP-MS analysis of iodine content.

The three mice with “small” tumors were denoted as: RL, NO, and R
The three mice with “large” tumors were denoted as: L, RR, and LL

Brains were sectioned (at NCL) into right and left hemispheres for analysis. Livers and spleens were single homogenates of the entire organ.

Sample preparation
An empty homogenization vial was initially weighed, tissue sample was added and the vial was re-weighed. Next, approx. 3-4 mL water was added to vial and weighed. The samples were then homogenized using a Precellys homogenizer, 5 times at 6500 rpm, 30 sec each, with 2 min chill in -80 freezer between runs.

Empty 15 mL vials were weighed. 500 µL of tissue homogenate was added to a vial. Next, 500 µL of 25% TMAH and 4.5 mL of dd H_2O was added. After approx. 10 min, the vials were capped, weighed, and transferred to microwave vessels. The samples were microwaved according to the program outlined in Table F-1 below.

Following microwave digestion, samples were diluted using 1% TMAH to fall within the calibration range. NaI (Sigma Aldrich) was used as the calibrant, and was diluted to final concentrations of 0, 1, 2, 5, 10, and 25 ng/g using 1% TMAH (Sigma Aldrich). ^{130}Te was used as an internal standard and was added using a sample T.

Table F-1. Microwave digestion program. Tissue samples were microwave-digested prior to ICP-MS analysis according to the following protocol.

Step	Power (W)	Power Setting (%)	Hold Time (min)
1	400	100	05:00
2	800	100	03:00
3	400	100	05:00

Table F-2. LOD and LOQ values. The LOD and LOQ values in each matrix are provided.

Tissue	LOD (ppt)	LOQ (ppt)
Brain	6.0	20
Liver	9.1	30
Spleen	8.0	27

Table F-3. NCL388-1 Recovery. NCL388-1 was spiked into the three tissue matrices and recovery was assessed by ICP-MS. Good sample recovery was achieved in all three matrices. The theoretical I concentration in NCL388 was 65 mg/mL.

Sample	AVG mg/ml
NCL388-1 in Brain	65 ± 4
NCL388-1 in Liver	63 ± 3
NCL388-1 in Spleen	61 ± 3

Table F-4. Summary of Results. A summary of the iodine concentrations for all tissues is provided.

	Non-Tumor Bearing Mice		Small Tumor-Bearing Mice		Large Tumor-Bearing Mice	
Tissue	Average mg I/ gram tissue	Average total mg I recovered	Average mg I/ gram tissue	Average total mg I recovered	Average mg I/ gram tissue	Average total g I recovered
Whole Brain, 20 hr (n = 1)	0.014 ± 0.001	0.0069 ± 0.0005	-	-	-	-
Whole Brain, 64 hr (n = 1)	0.0336 ± 0.0005	0.0175 ± 0.0002	-	-	-	-
Brain, Left Hemisphere (Tumor) (n = 3)	-	-	0.053 ± 0.005	0.014 ± 0.002	0.25 ± 0.02*<br>0.21 ± 0.06**	0.068 ± 0.002*<br>0.060 ± 0.020**
Brain, Right Hemisphere (n = 3)	-	-	0.034 ± 0.002	0.0087 ± 0.0005	0.044 ± 0.004	0.011 ± 0.001
Liver (n = 3)	-	-	2.2 ± 0.4	1.3 ± 0.2	2.2 ± 0.2*<br>1.7 ± 0.7**	1.1 ± 0.2*<br>0.9 ± 0.3**
Spleen (n = 3)	-	-	0.8 ± 0.1	0.14 ± 0.02	1.1 ± 0.2*<br>2 ± 1**	0.189 ± 0.003*<br>0.3 ± 0.2**

- Sample not tested
* Excludes outlier (Brain RR, Spleen L, Liver RR)
** Includes outlier (Brain RR, Spleen L, Liver RR)

Individual data, as well as calibration curves are provided on the pages that follow.

Quantitation of I in Mouse Brain Matrix, proof of concept study

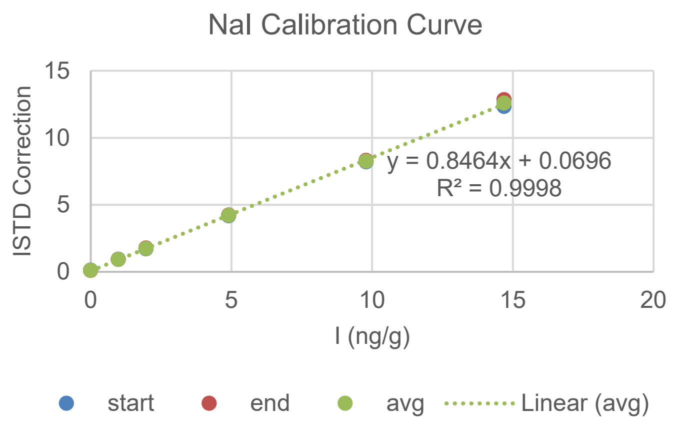

Sample Conc. (ng/g)	Signal at Start	Signal at End	Average
0	0.110625	0.090925	0.100775
0.982213	0.923656	0.921517	0.922586
1.967988	1.706254	1.763985	1.735119
4.905464	4.166827	4.232704	4.199765
9.788187	8.192955	8.297112	8.245034
14.69215	12.3301	12.83805	12.58407

Sample	CPS Ratio, ($^{127}I/^{130}Te$)	ng/g = ppb	% difference from Theoretical ppb	Dilution Corrected (mg/mL)	Average I conc. (mg/mL)
NCL388-1/Brain A – Run 1	7.97	9.34	-6.2	60.38	**60.1 ± 0.5**
NCL388-1/Brain A – Run 2	7.86	9.20		59.50	
NCL388-1/Brain A – Run 3	7.96	9.32		60.30	
NCL388-1/Brain B – Run 1	7.91	9.27	-2.6	61.89	**62.4 ± 0.4**
NCL388-1/Brain B – Run 2	7.99	9.36		62.50	
NCL388-1/Brain B – Run 3	8.02	9.39		62.70	
NCL388-1/Brain C – Run 1	9.64	11.30	+8.4	69.52	**69.4 ± 0.7**
NCL388-1/Brain C – Run 2	9.70	11.38		69.99	
NCL388-1/Brain C – Run 3	9.51	11.15		68.59	
NCL388-1/Brain D – Run 1	9.23	10.83	+4.0	66.25	**66.6 ± 0.6**
NCL388-1/Brain D – Run 2	9.23	10.82		66.19	
NCL388-1/Brain D – Run 3	9.37	10.99		67.21	

Non-Tumor-Bearing Mouse Brains

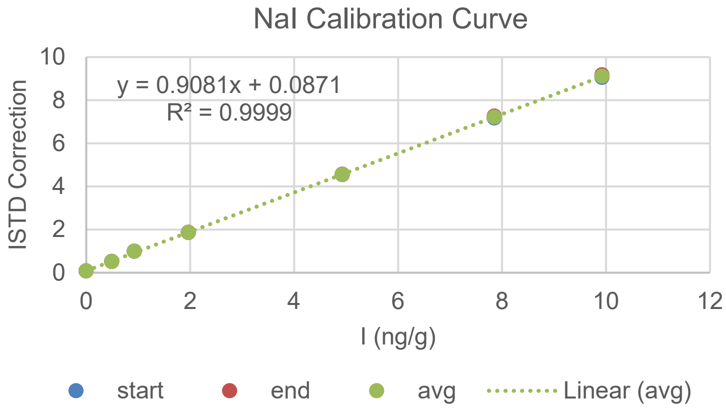

Sample Conc. (ng/g)	Signal at Start	Signal at End	Average
0	0.077697	0.06984	0.073768
0.492026	0.513793	0.507019	0.510406
0.925868	0.987493	0.989824	0.988659
1.965435	1.849723	1.866544	1.858133
4.926857	4.53751	4.558583	4.548046
7.853338	7.16932	7.254982	7.212151
9.924664	9.051237	9.169893	9.110565

Sample	CPS Ratio, ($^{127}I/^{130}Te$)	ng/g = ppb	µg I/g	g tissue	Total µg I recovered	Average µg I recovered
Mouse 1, 20 hr – Run 1	2.22	2.34	14.95	0.50578	7.56	6.9 ± 0.5
Run 2	1.97	2.07	13.23		6.69	
Run 3	1.94	2.04	13.04		6.60	
Mouse 2, 64 hr – Run 1	5.02	5.43	34.17	0.52119	17.81	17.5 ± 0.2
Run 2	4.90	5.30	33.32		17.36	
Run 3	4.91	5.31	33.42		17.42	

Calibration Curves for Tumor-Bearing Mouse Brains

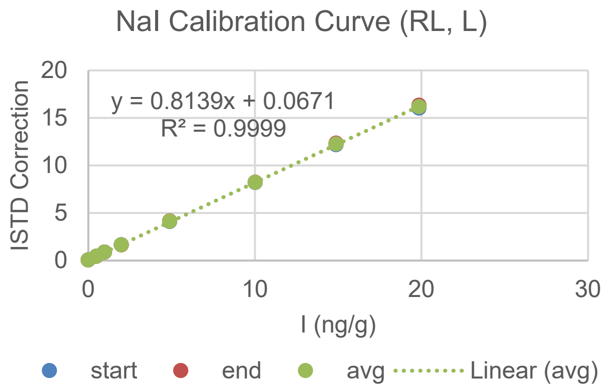

Sample Conc. (ng/g)	Signal at Start	Signal at End	Average
0	0.045019	0.040507	0.042763
0.485796	0.435607	0.443746	0.439677
0.975275	0.869219	0.877941	0.87358
1.986563	1.655604	1.626008	1.640806
4.892659	4.091696	4.162596	4.127146
10.02411	8.201826	8.234277	8.218052
14.87672	12.16145	12.33359	12.24752
19.86138	16.01718	16.31544	16.16631

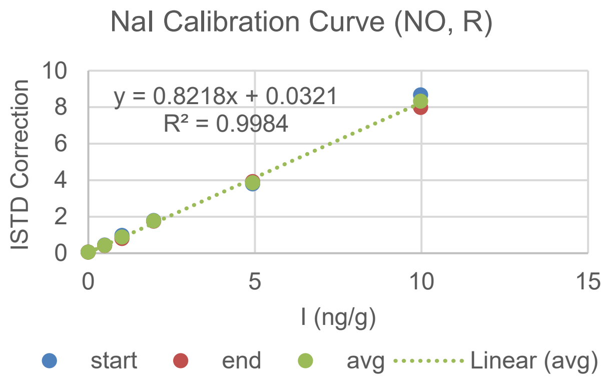

Sample Conc. (ng/g)	Signal at Start	Signal at End	Average
0	0.05119	0.049923	0.050556
0.492486	0.436412	0.410242	0.423327
1.01211	0.970281	0.795934	0.883108
1.965515	1.785348	1.74515	1.765249
4.935365	3.791574	3.919826	3.8557
9.978361	8.665184	7.980642	8.322913

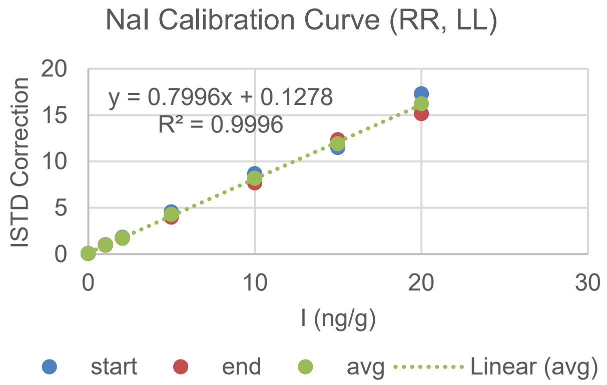

Sample Conc. (ng/g)	Signal at Start	Signal at End	Average
0	0.058522	0.046786	0.052654
1.037581	1.003739	0.954051	0.978895
2.059095	1.791312	1.704621	1.747967
4.989693	4.545314	3.972106	4.25871
9.995735	8.672604	7.663852	8.168228
14.98457	11.47682	12.32784	11.90233
20.00152	17.2895	15.14603	16.21777

Calibration Curves for Tumor-Bearing Mouse Spleens and Livers

Spleen

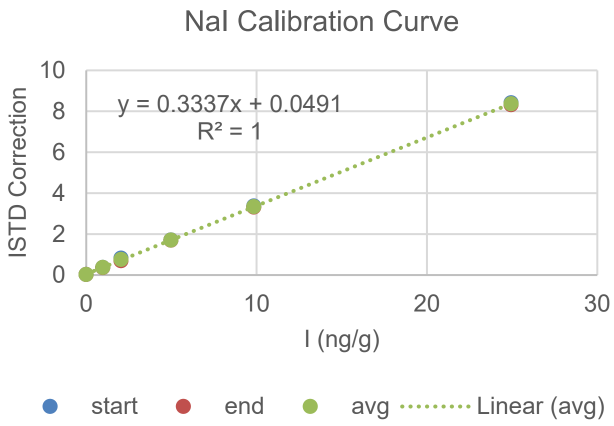

Sample Conc. (ng/g)	Signal at Start	Signal at End	Average
0	0.029532	0.029273	0.029403
0.979955	0.370198	0.364692	0.367445
2.049745	0.822507	0.698419	0.760463
4.974778	1.71801	1.696387	1.707198
9.844647	3.368558	3.315257	3.341908
24.94641	8.41512	8.323865	8.369492

Liver

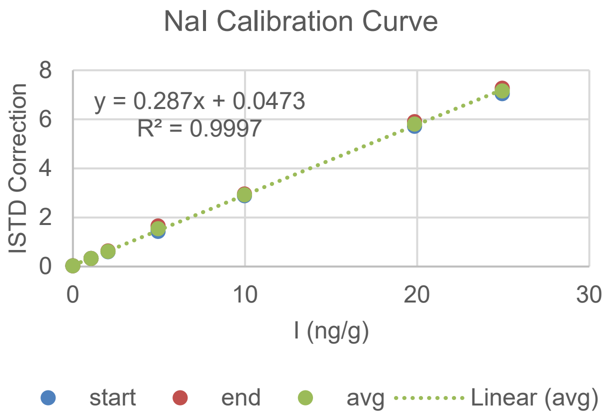

Sample Conc. (ng/g)	Signal at Start	Signal at End	Average
0	0.024135	0.023647	0.023891
1.059231	0.321501	0.325728	0.323614
2.045726	0.598754	0.628518	0.613636
4.960484	1.428281	1.646825	1.537553
9.977302	2.881342	2.954796	2.918069
19.85877	5.707784	5.902559	5.805171
24.95223	7.041182	7.25984	7.150511

Small Tumor-Bearing Mouse Brains

Sample	CPS Ratio, (^{127}I/^{130}Te)	ng/g = ppb	µg I/g	g brain	Total µg I recovered	Average (µg I)
**RL Right**– Run 1	1.38	1.61	32.81	0.25878	8.49	8.48 ± 0.02
Run 2	1.37	1.60	32.68		8.46	
Run 3	1.38	1.61	32.81		8.49	
**RL Left** – Run 1	2.23	2.66	53.77	0.27236	14.64	15 ± 1
Run 2	2.53	3.03	61.32		16.70	
Run 3	2.28	2.72	55.16		15.02	
**NO Right** – Run 1	1.60	1.91	37.69	0.25374	9.56	9.0 ± 0.5
Run 2	1.48	1.76	34.95		8.87	
Run 3	1.46	1.73	34.30		8.70	
**NO Left** – Run 1	2.21	2.64	57.22	0.25320	14.49	13.8 ± 0.9
Run 2	2.16	2.59	55.99		14.18	
Run 3	1.95	2.33	50.43		12.77	
**R Right** – Run 1	1.36	1.62	32.61	0.26545	8.66	8.7 ± 0.7
Run 2	1.27	1.51	30.46		8.09	
Run 3	1.47	1.75	35.37		9.39	
**R Left** – Run 1	1.90	2.27	49.03	0.24930	12.22	11.8 ± 0.6
Run 2	1.73	2.07	44.61		11.12	
Run 3	1.88	2.24	48.41		12.07	

Small Tumor-Bearing Mouse Spleens

Sample	CPS Ratio, ($^{127}I/^{130}Te$)	ng/g = ppb	mg I/g	g spleen	Total mg I recovered	Average (mg I)
RL Spleen– Run 1	1.66	4.82	0.90	0.13946	0.13	0.133 ± 0.007
Run 2	1.85	5.40	1.01		0.14	
Run 3	1.76	5.14	0.96		0.13	
NO Spleen – Run 1	1.91	5.58	0.79	0.19111	0.15	0.152 ± 0.002
Run 2	1.90	5.56	0.79		0.15	
Run 3	1.95	5.69	0.81		0.15	
R Spleen – Run 1	1.54	4.46	0.67	0.17635	0.12	0.1191 ± 0.0003
Run 2	1.55	4.48	0.68		0.12	
Run 3	1.54	4.48	0.68		0.12	

Small Tumor-Bearing Mouse Livers

Sample	CPS Ratio, ($^{127}I/^{130}Te$)	ng/g = ppb	mg I/g	g liver	Total mg I recovered	Average (mg I)
RL Liver – Run 1	4.43	15.25	2.57	0.55493	1.43	1.44 ± 0.02
Run 2	4.52	15.60	2.63		1.46	
Run 3	4.47	15.41	2.60		1.44	
NO Liver– Run 1	4.21	14.52	2.31	0.58055	1.34	1.35 ± 0.02
Run 2	4.22	14.52	2.31		1.34	
Run 3	4.31	14.87	2.37		1.38	
R Liver – Run 1	3.18	10.93	1.71	0.59903	1.02	1.030 ± 0.009
Run 2	3.24	11.11	1.74		1.04	
Run 3	3.19	10.95	1.71		1.03	

Large Tumor-Bearing Mouse Brains

Sample	CPS Ratio, ($^{127}I/^{130}Te$)	ng/g = ppb	µg I/g	g brain	Total µg I recovered	Average (µg I)
L Right – Run 1	1.85	2.19	48.14	0.25929	12.48	12.59 ± 0.10
Run 2	1.88	2.23	48.88		12.67	
Run 3	1.87	2.21	48.61		12.60	
L Left – Run 1	9.84	12.01	231.87	0.29491	68.38	69 ± 2
Run 2	9.77	11.92	230.18		67.88	
Run 3	10.31	12.58	242.95		71.65	
RR Right – Run 1	1.62	1.87	39.20	0.24977	9.79	9.9 ± 0.2
Run 2	1.61	1.86	38.92		9.72	
Run 3	1.67	1.93	40.34		10.08	
RR Left – Run 1	6.37	7.81	146.41	0.27593	40.40	37 ± 3
Run 2	5.39	6.58	123.45		34.06	
Run 3	5.91	7.23	135.52		37.39	
LL Right – Run 1	1.56	1.80	42.16	0.23341	9.84	10.0 ± 0.2
Run 2	1.57	1.81	42.39		9.89	
Run 3	1.62	1.87	43.96		10.26	
LL Left – Run 1	9.50	11.72	264.64	0.25213	66.72	66 ± 2
Run 2	9.15	11.29	254.95		64.28	
Run 3	9.71	11.99	270.75		68.26	
**Re-runs of Brain RR , Left half**						
RR Left A – Run 1	6.37	7.81	146.41	0.27593	40.40	37 ± 3
Run 2	5.39	6.58	123.45		34.06	
Run 3	5.91	7.23	135.52		37.39	
RR Left B – Run 1	6.18	6.87	137.18	0.27593	37.85	37.6 ± 0.4
Run 2	6.17	6.85	136.89		37.77	
Run 3	6.05	6.72	134.32		37.06	

Large Tumor-Bearing Mouse Spleens

Sample	CPS Ratio, ($^{127}I/^{130}Te$)	ng/g = ppb	mg I/g	g spleen	Total mg I recovered	Average (mg I)
L Spleen – Run 1	7.38	21.98	3.34	0.16162	0.54	0.542 ± 0.007
Run 2	7.33	21.83	3.32		0.54	
Run 3	7.52	22.37	3.40		0.55	
RR Spleen – Run 1	2.33	6.85	1.26	0.14684	0.19	0.187 ± 0.002
Run 2	2.39	7.00	1.29		0.19	
Run 3	2.35	6.90	1.27		0.19	
LL Spleen – Run 1	2.45	7.19	0.97	0.19666	0.19	0.1914 ± 0.0006
Run 2	2.46	7.23	0.98		0.19	
Run 3	2.46	7.21	0.97		0.19	
**Re-runs of L Spleen**						
L Spleen A – Run 1	7.38	21.98	3.34	0.16162	0.54	0.542 ± 0.007
Run 2	7.33	21.83	3.32		0.54	
Run 3	7.52	22.37	3.40		0.55	
L Spleen B – Run 1	5.27	20.24	3.38	0.16162	0.55	0.56 ± 0.05
Run 2	5.88	22.59	3.77		0.61	
Run 3	4.97	19.06	3.18		0.51	

Large Tumor-Bearing Mouse Livers

Sample	CPS Ratio, ($^{127}I/^{130}Te$)	ng/g = ppb	mg I/g	g liver	Total mg I recovered	Average (mg I)
L Liver – Run 1	2.96	10.15	1.99	0.46442	0.92	0.922 ± 0.005
Run 2	2.98	10.20	2.00		0.93	
Run 3	2.95	10.10	1.98		0.92	
RR Liver– Run 1	1.50	5.05	0.86	0.52656	0.46	0.460 ± 0.004
Run 2	1.52	5.13	0.88		0.46	
Run 3	1.52	5.13	0.88		0.46	
LL Liver – Run 1	4.01	13.81	2.38	0.51367	1.22	1.21 ± 0.02
Run 2	3.91	13.48	2.32		1.19	
Run 3	3.98	13.71	2.36		1.21	
**Re-runs of RR Liver**						
RR Liver A – Run 1	1.50	5.05	0.86	0.52656	0.46	0.460 ± 0.004
Run 2	1.52	5.13	0.88		0.46	
Run 3	1.52	5.13	0.88		0.46	
RR Liver B – Run 1	2.05	7.27	1.15	0.52656	0.60	0.57 ± 0.03
Run 2	1.84	6.50	1.03		0.54	
Run 3	1.88	6.64	1.05		0.55	

Appendix G. ICP-MS Analysis of Tissue Samples

Nanoprobes provided a second set of tissue samples for ICP-MS analysis of iodine concentrations. Again, Nanoprobes conducted the animal study, and NCL only completed the ICP-MS analysis of these samples. Brain samples were provided in a bulk solution of formalin. Liver, spleen and kidney were also provided in formalin, with all three clearance organs stored in the same formalin tube. Samples were stored at 4°C upon receipt.

A table summarizing the received samples is below. For each sample, 1 vial containing brain tissue in formalin and 1 vial containing the liver, spleen and kidney clearance organs in formalin were received. Mice were dosed at 1.75g/kg iohexol nanoparticles. Samples NO and RR were blanks not receiving iodine. Samples were received in January 2019.

Sample Nomenclature
Mouse 1 R
Mouse 1 L
Mouse 1 LR
Mouse 1 NO
Mouse 1 RR
Mouse 2 R
Mouse 2 L
Mouse 2 LR
Mouse 2 NO
Mouse 2 RR

Brains, as well as the formalin used for brain storage, were analyzed for iodine content. Additionally, livers, spleens and kidneys for each sample were analyzed as single homogenates of the combined clearance organs. The formalin used to store the clearance organs was also analyzed for iodine content.

The iodine concentration in the samples was determined using either an Agilent 7500cx ICP-MS or a Perkin-Elmer NexION 2000B. Tuning of the instrument was performed daily prior to sample testing.

<u>Sample Preparation, Brains</u>

Brains were removed from the formalin solution and placed on a disposable weigh boat. Excess liquid was removed by wicking using a kimwipe. The brains were allowed to sit at room temperature in open air for 30 minutes to allow to remaining excess moisture to dry. The brains were then transferred to a pre-weighed disposable weigh boat and the total brain weight was recorded. The brain was transferred to PFA (55 mL, CEM corporation) microwave digestion vessels and 10 mL of tetramethylammonium hydroxide (TMAH, Sigma Aldrich, 25% by weight) was added to the vessels. Additionally, two control blanks containing only the TMAH solution were prepared along with two samples of spiked NaI solutions. For the spike solutions, 1 and 0.1 mL of a 0.99898 µg/g solution of iodide (NaI 99.999% trace metal basis, Sigma Aldrich) was added to a PFA vessel and diluted with 10 mL of TMAH. The samples were microwave digested using the method in Table G-1.

Table G-1. Microwave digestion program. Tissue samples were microwave-digested prior to ICP-MS analysis according to the following protocol.

Step	Power	Ramp Time	Temp	Hold Time
1	800W (100%)	25 min	120°C	20 min
2	400W (0%)	0 min	0°C	30 min
3	800W (100%)	15 min	195°C	20 min
4	400W (0%)	0 min	0°C	25 min

After the samples were microwaved, the brains were completely dissolved. The digested material was transferred to a pre-weighed 60 mL LDPE sample bottle and the weight of the digest was recorded. The samples were diluted to a total of 50 mL using a 1% TMAH solution and the total weight was recorded. This solution will be subsequently referred to as Dilution 1. For iodine quantitation, the samples were diluted an additional time by adding 1 mL of Dilution 1 to a pre-weighed 18 mL LDPE sample vial. The weight of the solution was recorded, and was diluted to 10 mL using a 1% TMAH solution. The total weight was recorded.

Calibration samples: Calibration standards were prepared from NaI (NaI 99.999% trace metal basis, Sigma Aldrich) and serial dilution with 1% TMAH to achieve the final concentrations necessary for iodine quantitation. During the final dilution step, the samples were matrix matched using 1 mL of either the Mouse 1 No or Mouse 2 No Dilution 1 samples. Calibration standards had concentrations of 0, 0.498, .997, 9.91738, 19.984, 29.980, 40.086, and 50.061 ng/g.

ICP-MS Method: An Agilent 7500cx ICP-MS instrument was used to conduct the iodine quantitation. Iodine was quantified using an external calibration constructed using the standards noted above. Three points per mass were measured with an integration time of 0.06 seconds. Ten replicants per measurement were averaged to give the final iodine counts. A 20 ppb solution of Indium in 1% TMAH was used as an internal standard and added via a sample T to analyte solution. However, the variance of the internal standard counts compared with the analyte counts suggested that the injection of the internal standard was not consistent, so the raw counts were used to construct the calibration curves. Software: Chemstation. Masses Analyzed: I – 126, In – 115.

Sample Preparation, Brain Formalin

The total amount of formalin (that each brain was stored in) was determined by transferring the total contents of the remaining formalin to a pre-weighed 15 mL Falcon tube. The total weight was recorded. For iodine determination, 1 mL of the formalin was transferred to a pre-weighed 15 mL Falcon tube. The weight was recorded, and the formalin was diluted to 10 mL using a 1% TMAH solution. The final weight of the solution was recorded.

Calibration samples: Calibration standards were prepared from NaI (NaI 99.999% trace metal basis, Sigma Aldrich) and serial dilution with 1% TMAH to achieve the final concentrations necessary for iodine quantitation. During the final dilution step, the samples were matrix matched using 1 mL of the Mouse 1 No. Calibration standards had concentrations of 0, 0.993, 1.987, 3.975, 5.975, and 7.691 ng/g.

ICP-MS Method: A NexION 200B ICP-MS (Perkin Elmer, Waltham, MA) was used to conduct iodine quantitation. Iodine was quantified using an external calibration constructed using the standards as noted above. Three scans per mass were measured with an integration time of 0.06 seconds. Ten replicants per measurement were averaged to give the final iodine counts. Software: Syngistix, Masses Analyzed: I – 126.

Sample Preparation, Clearance Organs

Organs were removed from the formalin solution and placed on a disposable weigh boat. The organs were digested and analyzed as a single sample due to leaching and cross-contamination of iodine as a result of the storage conditions. Excess liquid was removed by wicking using a kimwipe. The organs were allowed to sit at room temperature in open air for 30 minutes to allow to remaining excess moisture to dry. The organs were then transferred to a pre-weighed disposable weigh boat and the total brain weight was recorded. The brain was transferred to PFA (55 mL, CEM corporation) microwave digestion vessels and 10 mL of tetramethylammonium hydroxide (TMAH, Sigma Aldrich, 25% by weight) was added to the vessels. Additionally, two control blanks containing only the TMAH solution were prepared along with two samples of spiked NaI solutions. For the spike solutions, 1 and 0.5 mL of a 50.04 µg/g solution of iodide (NaI 99.999% trace metal basis, Sigma Aldrich) was added to a PFA vessel and diluted with 10 mL of TMAH. The samples were microwave digested using the method in Table B-1.

After the samples were microwaved, the organs were completely dissolved. The digested material was transferred to a pre-weighed 60 mL LDPE sample bottle and the weight of the digest was recorded. The samples were diluted to a total of 50 mL using a 1% TMAH solution and the total weight was recorded. This solution will be subsequently referred to as Dilution 1. For iodine quantitation, the samples were diluted an additional time by adding 50 µL of Dilution 1 to a pre-weighed 50 mL LDPE sample vial. The weight of the solution was recorded, and was diluted to 50 mL using a 1% TMAH solution. The total weight was recorded. The spiked samples were diluted similarly, however, 500 µL of Dilution 1 were used instead of 50 µL. The spiked samples were matrix matched using 50 µL of Mouse 1 No Dilution 1.

Calibration samples: Calibration standards were prepared from NaI (NaI 99.999% trace metal basis, Sigma Aldrich) and serial dilution with 1% TMAH to achieve the final concentrations necessary for iodine quantitation. During the final dilution step, the samples were matrix matched using 50 µL of either the Mouse 1 Dilution 1 samples. Calibration standards had concentrations of 0, 1.009, 4.970, 10.022, 20.040, 30.176, and 39.991 ng/g.

ICP-MS Method: A Perkin Elmer NexION 200B ICP-MS instrument was used to conduct the iodine quantitation. Iodine was quantified using an external calibration constructed using the standards as noted above. Three points per mass were measured with an integration time of 0.06 seconds. Ten replicants per measurement were averaged to give the final iodine counts. A 20 ppb solution of indium in 1% TMAH was used as an internal standard and added via a sample T to analyte solution. Calibration curves were constructed using internal standard corrected counts. Internal standard corrected counts were calculated by dividing the iodine signal by the Indium signal for each measurement.

Sample Preparation, Clearance Organs Formalin

The total amount of formalin (that the three clearance organs were stored in) was determined by transferring the total contents of the remaining formalin to a pre-weighed 50 mL Falcon tube. The total weight was recorded. For iodine determination, 0.5 mL of the formalin was transferred

to a pre-weighed 15 mL Falcon tube. The weight was recorded, and the formalin was diluted to 10 mL using a 1% TMAH solution. The final weight of the solution was recorded.

Calibration samples: Calibration standards were prepared from NaI (NaI 99.999% trace metal basis, Sigma Aldrich) and serial dilution with 1% TMAH to achieve the final concentrations necessary for iodine quantitation. During the final dilution step, the samples were matrix matched using 1 mL of the Mouse 1 No. Calibration standards had concentrations of 0, 9.739, 19.717, 36.680, 59.878, and 79.832 ng/g.

ICP-MS Method: A NexION 200B ICP-MS (Perkin Elmer, Waltham, MA) was used to conduct iodine quantitation. Iodine was quantified using an external calibration constructed using the standards as noted above. Three scans per mass were measured with an integration time of 0.06 seconds. Ten replicants per measurement were averaged to give the final iodine counts. Software: Syngistix, Masses Analyzed: I – 126.

Quantitation of Iodine in Brain Tissue

Calibration Curve

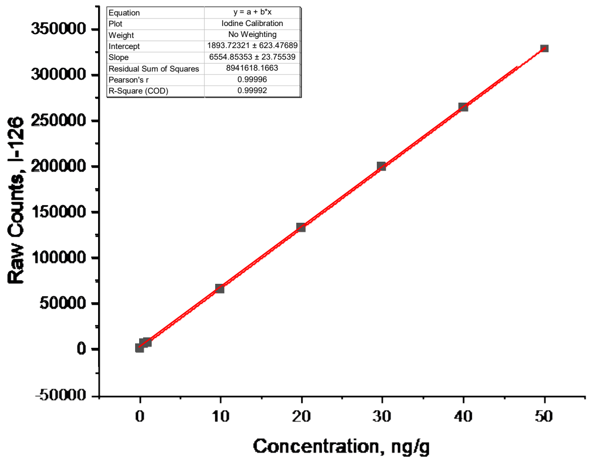

Concentration of Iodine, µg/g of tissue

Sample ID	[I], µg/g	Error, µg/g
Mouse 1 R	20.5	2.7
Mouse 1L	28.0	1.0
Mouse 1 LR	24.0	0.9
Mouse 1 NO	-0.2	0.3
Mouse 1 RR	-0.2	0.3
Mouse 2 R	49.2	2.6
Mouse 2 L	27.9	1.1
Mouse 2 LR	26.6	1.1
Mouse 2 NO	-0.2	0.3
Mouse 2 RR	-0.2	0.3
1 mL of 1 ppm spike*	1.0	0.1

* Actual value of the iodine spike is 0.999 µg/g, giving a % recovery of 102.5%

Total Iodine, µg

Sample ID	[I], µg	Error, µg
Mouse 1 R	9.3	1.2
Mouse 1L	12.8	0.5
Mouse 1 LR	11.9	0.4
Mouse 1 NO	-0.1	0.1
Mouse 1 RR	-0.1	0.1
Mouse 2 R	24.7	1.3
Mouse 2 L	10.8	0.4
Mouse 2 LR	11.4	0.5
Mouse 2 NO	-0.1	0.1
Mouse 2 RR	-0.1	0.1

Quantitation of Iodine in Brain Formalin

Calibration Curve

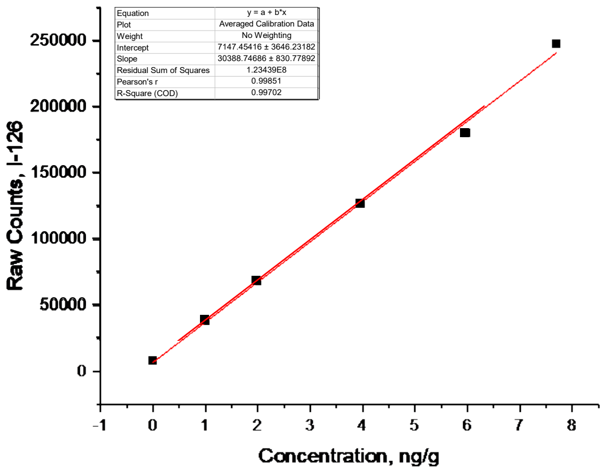

Concentration of Iodine, μg/g of tissue

Sample ID	[I], μg/g	Error, μg/g
Mouse 1 R	0.015	0.003
Mouse 1L	0.026	0.003
Mouse 1 LR	0.023	0.003
Mouse 1 NO	0.000	0.003
Mouse 1 RR	0.000	0.003
Mouse 2 R	0.057	0.005
Mouse 2 L	0.022	0.003
Mouse 2 LR	0.019	0.003
Mouse 2 NO	0.001	0.003
Mouse 2 RR	0.000	0.003

Total Iodine, μg

Sample ID	[I], μg	Error, μg
Mouse 1 R	0.20	0.04
Mouse 1L	0.34	0.04
Mouse 1 LR	0.30	0.04
Mouse 1 NO	0.00	0.04
Mouse 1 RR	0.00	0.04
Mouse 2 R	0.76	0.07
Mouse 2 L	0.29	0.04
Mouse 2 LR	0.26	0.04
Mouse 2 NO	0.01	0.04
Mouse 2 RR	0.00	0.04

Quantitation of Iodine in Clearance Tissues

Calibration Curve

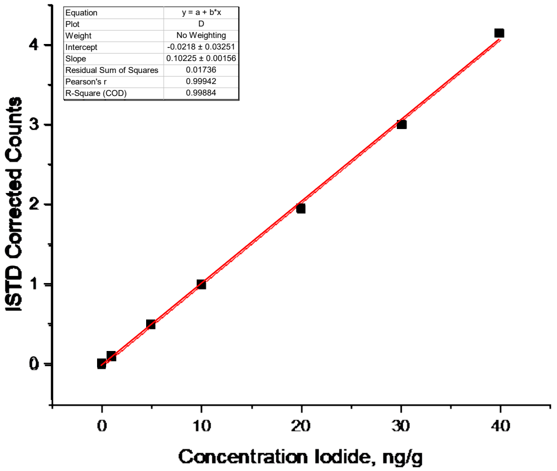

Concentration of Iodine, µg/g of tissue

Sample ID	[I], µg/g	Error, µg/g
Mouse 1 R	606.4	48.4
Mouse 1L	1196.4	106.6
Mouse 1 LR	946.1	105.5
Mouse 1 NO	9.5	29.7
Mouse 1 RR	6.5	20.4
Mouse 2 R	884.7	80.3
Mouse 2 L	1029.6	99.0
Mouse 2 LR	567.0	65.1
Mouse 2 NO	10.0	31.3
Mouse 2 RR	7.5	23.7
Spike #13*	50.7	8.6
Spike #14*	49.6	5.9

Total Iodine, µg

Sample ID	[I], µg	Error, µg
Mouse 1 R	808.9	64.5
Mouse 1L	2129.4	189.7
Mouse 1 LR	932.2	103.9
Mouse 1 NO	12.4	39.1
Mouse 1 RR	12.8	40.2
Mouse 2 R	1332.8	121.0
Mouse 2 L	1149.8	110.5
Mouse 2 LR	762.7	87.6
Mouse 2 NO	12.4	38.9
Mouse 2 RR	12.9	41.0

*Actual value of the iodine spike is 50.0 µg/g, giving a % recovery of 101.4% and 99.1% for Spike #13 and Spike #14, respectively.

Quantitation of Iodine in Clearance Tissue Formalin

Calibration Curve

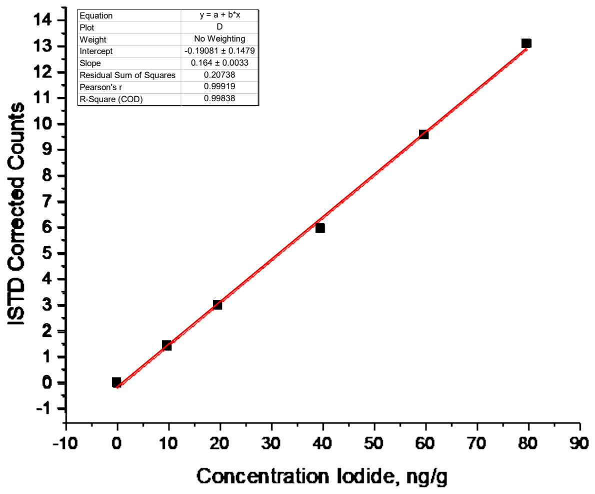

Concentration of Iodine, µg/g of tissue

Sample ID	[I], µg/g	Error, µg/g
Mouse 1 R	0.68	0.05
Mouse 1L	1.1	0.1
Mouse 1 LR	0.74	0.06
Mouse 1 NO	0.03	0.04
Mouse 1 RR	0.03	0.04
Mouse 2 R	0.84	0.07
Mouse 2 L	1.18	0.09
Mouse 2 LR	0.9	0.1
Mouse 2 NO	0.03	0.04
Mouse 2 RR	0.03	0.04

Total Iodine, µg

Sample ID	[I], µg/g	Error, µg/g
Mouse 1 R	36.4	3.6
Mouse 1L	57.0	6.7
Mouse 1 LR	40.3	4.3
Mouse 1 NO	1.5	2.7
Mouse 1 RR	1.8	2.7
Mouse 2 R	45.6	5.0
Mouse 2 L	64.5	6.1
Mouse 2 LR	47.2	7.4
Mouse 2 NO	1.6	2.7
Mouse 2 RR	1.5	2.7

Summary, Iodine Concentration (µg/ g of tissue or formalin)

	Brains		Clearance Organs	
Sample ID	Tissue [I], µg/g	Formalin [I], µg/g	Tissue [I], µg/g	Formalin [I], µg/g
Mouse 1 R	20.5 + 2.7	0.015 + 0.003	606.4 + 48.4	0.68 + 0.05
Mouse 1L	28.0 + 1.0	0.026 + 0.003	1196.4 + 106.6	1.1 + 0.1
Mouse 1 LR	24.0 + 0.9	0.023 + 0.003	946.1 + 105.5	0.74 + 0.06
Mouse 1 NO	-0.2 + 0.3	0.000 + 0.003	9.5 + 29.7	0.03 + 0.04
Mouse 1 RR	-0.2 + 0.3	0.000 + 0.003	6.5 + 20.4	0.03 + 0.04
Mouse 2 R	49.2 + 2.6	0.057 + 0.005	884.7 + 80.3	0.84 + 0.07
Mouse 2 L	27.9 + 1.1	0.022 + 0.003	1029.6 + 99.0	1.18 + 0.09
Mouse 2 LR	26.6 + 1.1	0.019 + 0.003	567.0 + 65.1	0.9 + 0.1
Mouse 2 NO	-0.2 + 0.3	0.001 + 0.003	10.0 + 31.3	0.03 + 0.04
Mouse 2 RR	-0.2 + 0.3	0.000 + 0.003	7.5 + 23.7	0.03 + 0.04

Summary, Iodine Concentration (µg)

	Brains		Clearance Organs	
Sample ID	Tissue [I], µg	Formalin [I], µg	Tissue [I], µg	Formalin [I], µg
Mouse 1 R	9.3 + 1.2	0.20 + 0.04	808.9 + 64.5	36.4 + 4.9
Mouse 1L	12.8 + 0.5	0.34 + 0.04	2129.4 + 189.7	57.0 + 9.3
Mouse 1 LR	11.9 + 0.4	0.30 + 0.04	932.2+ 103.9	40.3 + 6.0
Mouse 1 NO	-0.1 + 0.1	0.00 + 0.04	12.4 + 39.1	1.5 + 3.8
Mouse 1 RR	-0.1 + 0.1	0.00 + 0.04	12.8 + 40.2	1.8 + 3.7
Mouse 2 R	24.7 + 1.3	0.76 + 0.07	1332.8 + 121.0	45.6+ 7.0
Mouse 2 L	10.8 + 0.4	0.29 + 0.04	1149.8 + 110.5	64.5 + 8.5
Mouse 2 LR	11.4 + 0.5	0.26 + 0.04	762.7 + 87.6	47.2+ 10.3
Mouse 2 NO	-0.1 + 0.1	0.01 + 0.04	12.4 + 38.9	1.6 + 3.7
Mouse 2 RR	-0.1 + 0.1	0.00 + 0.04	12.9 + 41.0	1.5 + 3.7

1. United States Pharmacopeia and National Formulary, <85> Bacterial Endotoxins Test, **2011**, Rockville, MD.
2. FDA Guidance for Industry Estimating the maximum safe starting dose in initial clinical trials for therapeutics in adult healthy volunteers. **July 2005**, CDER.
3. Stern ST, Adiseshaiah PP, Crist RM. Autophagy and lysosomal dysfunction as emerging mechanisms of nanomaterial toxicity. *Part Fibre Toxicol,* **2012**, 9, 20. DOI: 10.1186/1743-8977-9-20. PMID: 22697169.
4. Krause W, Press WR. Influence of Contrast Media on Blood Coagulation. *Investigative Radiology*, **1997**, 32(5), 249-259. DOI: 10.1097/00004424-199705000-00001. PMID: 9140744.
5. Chuang FR, Chen TC, Wang IK, Chuang CH, Chang HW, Ting-Yu Chiou T, Cheng YF, Lee WC, Chen WC, Yang KD, Lee CH. Comparison of iodixanol and iohexol in patients undergoing intravenous pyelography: a prospective controlled study. *Ren Fail.* **2009**, 31(3), 181-188. doi: 10.1080/08860220802669636. PMID: 19288321.
6. Yamazaki K, Tanigawa K, Suzuki K, Yamada E, Yamada T, Takano K, Obara T, Sato K. Iodide-induced chemokines and genes related to immunological function in cultured human thyroid follicles in the presence of thyrotropin. *Thyroid*. **2010**, 20(1), 67-76. doi: 10.1089/thy.2009.0242. PMID: 20025541.
7. Bilal MY, Dambaeva S, Kwak-Kim J, Gilman-Sachs A, Beaman KD. A Role for Iodide and Thyroglobulin in Modulating the Function of Human Immune Cells. *Front Immunol*. **2017**, 8, 1573. doi: 10.3389/fimmu.2017.01573. PMID: 29187856.
8. Zampronio AR, Souza GE, Silva CA, Cunha FQ, Ferreira SH. Interleukin-8 induces fever by a prostaglandin-independent mechanism. *Am J Physiol*. **1994**, 266(5 Pt 2), R1670-1674. doi: 10.1152/ajpregu.1994.266.5.R1670. PMID: 8203649.
9. Hirao Y, MD Kanda T, Aso Y, Mitsuhashi M, Kobayashi I. Interleukin-8—An Early Marker for Bacterial Infection. *Laboratory Medicine,* **2000**, 31(1), 39-44.
10. Dinarello CA. The history of fever, leukocytic pyrogen and interleukin-1. *Temperature (Austin)*. **2015**, 2(1), 8-16. doi:10.1080/23328940.2015.1017086. PMID: 27226996.
11. Dinarello CA. Infection, fever, and exogenous and endogenous pyrogens: some concepts have changed. *J Endotoxin Res*. **2004**, 10(4), 201-222. doi: 10.1179/096805104225006129. PMID: 15373964.
12. Valentini S, Santoro G, Baffetta F, Franceschi S, Paludi M, Brandini E, Gherardini L, Serruto D, Capecchi B. Monocyte-activation test to reliably measure the pyrogenic content of a vaccine: An in vitro pyrogen test to overcome in vivo limitations. *Vaccine*. **2019**, 37(29), 3754-3760. doi: 10.1016/j.vaccine.2018.10.082. PMID: 30448065.
13. Casey LC, Balk RA, Bone RC. Plasma cytokine and endotoxin levels correlate with survival in patients with the sepsis syndrome. *Ann Intern Med*. **1993**, 119(8), 771-778. doi: 10.7326/0003-4819-119-8-199310150-00001. PMID: 8379598.
14. Engel A, Kern WV, Mürdter G, Kern P. Kinetics and correlation with body temperature of circulating interleukin-6, interleukin-8, tumor necrosis factor alpha and interleukin-1 beta in patients with fever and neutropenia. *Infection*. **1994**, 22(3), 160-164. doi: 10.1007/BF01716695. PMID: 7927810.
15. Irizarry Rovira AR, Bennet BM, Bolon B, Braendli-Baiocco A, Chandra S, Fleurance R, Garman R, Hutto D, Lane J, Romeike A, Sargeant A, Zimmerman B. Scientific and Regulatory Policy Committee Points to Consider: Histopathologic Evaluation in Safety Assessment Studies for PEGylated Pharmaceutical Products. *Pharmaceutical Products. Toxicol Pathol.* **2018**, 46(6), 616-635. doi: 10.1177/0192623318791801. PMID: 30092727

16. Nagashima K, Zheng J, Parmiter D, Patri AK. Biological tissue and cell culture specimen preparation for TEM nanoparticle characterization. Methods Mol Biol. 2011;697:83-91. doi: 10.1007/978-1-60327-198-1_8. PMID:21116956

NCL protocols used throughout this project can be found here:
https://ncl.cancer.gov/resources/assay-cascade-protocols

ABBREVIATIONS

A	albumin
A/G	albumin/globulin ratio
AAALAC	Association for Assessment and Accreditation of Laboratory Animal Care
AF4	asymmetric-flow field-flow fractionation
ALT	alanine aminotransferase
ALP	alkaline phosphatase
API	active pharmaceutical ingredient
APTT	activated partial thromboplastin time
BA	basophil
BLOQ	below limit of quantification
BSA	bovine serum albumin
BUN	blood urea nitrogen
CBC	complete blood counts
CFU	colony forming units
CMH	cyanmethemoglobin
CV	coefficient of variation
CVF	cobra venom factor
d.nm	diameter, in nanometers
DLS	dynamic light scattering
EDX	energy dispersive x-ray spectroscopy
EL	endotoxin limit
EM	electron microscopy
EO	eosinophil
EU	endotoxin unit
FBS	fetal bovine serum
FDA	Food and Drug Administration
G	globulin
GTA	general toxicity
H&E	haematoxylin-eosin
Hb	hemoglobin
HCT	hematocrit
HED	human equivalent dose
Hep G2	human hepatocarcinoma cells
ICP-MS	inductively coupled plasma mass spectrometry
IHC	immunohistochemistry
ITA	immunotoxicity assay
LAL	Limulus Amebocyte Lysate
LDH	lactate dehydrogenase
LDPE	low density polyethylene
LLC-PK1	porcine renal proximal tubule cell line
LOD	limit of detection
LOQ	limit of quantitation
LY	lymphocytes
MALS	multi-angle light scattering
MCH	mean corpuscular hemoglobin
MCHC	mean corpuscular hemoglobin concentration
MCV	mean corpuscular volume
MO	monocytes

MPV	mean platelet volume
MTT	3-(4,5-dimethylthiazol-2-yl)-2,5-diphenyltetrazolium bromide
MVD	maximum valid dilution
NC	negative control
NCL	Nanotechnology Characterization Laboratory
ND	not detected
NE	neutrophil
NIST	National Institute of Standards and Technology
NT	not tested
PBS	phosphate buffered saline
PC	positive control
PCC	physicochemical characterization
PdI	polydispersity index
PEG	polyethylene glycol
PLT	platelet count
PPP	platelet poor plasma
PRP	platelet rich plasma
PT	prothrombin time
RBC	erythrocyte count
RDW	red blood cell distribution width
RI	refractive index
SD	standard deviation
SE	standard error
SEC	size exclusion chromatography
STE	sterility
TGA	thermogravimetric analysis
TMAH	tetramethylammonium hydroxide
TS	tryptic soy
TT	thrombin time
USP	United States Pharmacopeia
WBC	total leukocyte count

CONTRIBUTORS & ACKNOWLEDGEMENTS

Nanotechnology Characterization Laboratory

Stephan T. Stern
Marina A. Dobrovolskaia
Jeffrey D. Clogston
Rachael M. Crist
Yingwen Hu
Siva Dasa
Matthew Hansen
Tim Potter
Barry Neun
Sarah Skoczen
Edward Cedrone
Kelsie Snapp
Alison Vermilya
Teagan Ware
Jie Xu
Cassandra Mankus

This project has been funded in whole or in part with Federal funds from the Frederick National Laboratory for Cancer Research, National Institutes of Health, under contract 75N91019D00024.. The content of this publication does not necessarily reflect the views or policies of the Department of Health and Human Services, nor does mention of trade names, commercial products or organizations imply endorsement by the US Government.
